# Herbal mixtures in traditional medicine in Northern Peru

**DOI:** 10.1186/1746-4269-6-10

**Published:** 2010-03-14

**Authors:** Rainer W Bussmann, Ashley Glenn, Karen Meyer, Alyse Kuhlman, Andrew Townesmith

**Affiliations:** 1 William L Brown Center, Missouri Botanical Garden, PO Box 299, St Louis, MO 63166-0299, USA

## Abstract

The investigation of plant mixtures used in traditional medicine in Northern Peru yielded a total of 974 herbal preparations used to treat 164 different afflictions. Psychosomatic disorders were, with almost 30% of all recipes applied, the most important afflictions treated. In most cases, healers used only one or two mixtures to treat an illness. However, up to 49 different preparations were used to treat the same disease. This indicates a high degree of experimentation. Altogether 330 plant species, representing almost 65% of the medicinal flora used in the region were applied in mixtures. The overwhelming number of plant mixtures contained 2-7 different plant species, although in the most extreme case 27 distinct species were included. The cluster analysis confirmed that mixtures used for applications like inflammations, infections and blood purification, as well as cough, cold, bronchitis or other respiratory disorders, or urinary infection and kidney problems had similar floristic compositions. Mixtures used for nervous system disorders, anxiety and heart problems often had a similar composition

## Introduction

Traditional Medicine, defined by the WHO as " medical knowledge systems that developed over generations within various societies before the era of modern medicine, including the health practices, approaches, knowledge and beliefs incorporating plant, animal and mineral-based medicines, spiritual therapies, manual techniques and exercises, applied singularly or in combination to treat, diagnose and prevent illnesses or maintain well-being." [1] is used globally and has rapidly growing economic importance. In developing countries, Traditional Medicine is often the only accessible and affordable treatment available. In Latin America, the WHO Regional Office for the Americas (AMRO/PAHO) reports that 71% of the population in Chile and 40% of the population in Colombia have used Traditional Medicine. In many Asian countries Traditional Medicine is widely used, even though Western medicine is often readily available. In Japan, 60-70% of allopathic doctors prescribe traditional medicines for their patients. In the US the number of visits to providers of Complementary Alternative Medicine (CAM, codified herbal medicine) now exceeds by far the number of visits to all primary care physicians [1-3].

Complementary Alternative Medicine is also becoming more and more popular in many developed countries. Forty-two percent of the population in the US have used Complementary Alternative Medicine at least once [4], and a national survey reported the use of at least one of 16 alternative therapies increased from 34% in 1990 to 42% in 1997 [5].

The expense for the use of Traditional and Complementary Alternative Medicine is exponentially growing in many parts of the world. The 1997 out-of-pocket Complementary Alternative Medicine expenditure was estimated at US$ 2.7 billion in the USA. The world market for herbal medicines based on traditional knowledge is now estimated at US$ 60 billion [6].

Northern Peru is believed to be the center of the Central Andean Health Axis [7], and traditional medicinal practices in this region are still an important component of everyday life [8-16]. Traditional Medicine is also gaining more and more respect by national governments and health providers. Peru's National Program in Complementary Medicine and the Pan American Health Organization recently compared Complementary Medicine to allopathic medicine in clinics and hospitals operating within the Peruvian Social Security System [17].

According to WHO [3], the sustainable cultivation and harvesting of medicinal species is one of the most important challenges for the next few years.

Many traditional healers rely on herbal preparations, often consisting of complex ingredients and with very specific preparations, to treat their patients' illnesses, rather than just employing single plant extracts. However, studies documenting these preparations and analyzing the composition of the mixtures are almost non-existent. Most ethnobotanical studies to date document the "use" of single species, without asking the important question if the plants in question are really employed alone, or if they are in fact part of a more complex preparation. Cano & Volpato [18] and Carmona et al. [19] were amongst the first authors to respond to this challenge, and reported on plant mixtures employed in Cuba and the Middle East, and Vandebroek et al. [20] demonstrated the great complexity of plant preparations in the Dominican Republic. No information however was available for the very species rich Andean pharmacopoeia.

The present publication attempts to give a detailed overview on the herbal mixtures employed by traditional practitioners in Northern Peru and the specific applications they are used for, in order to provide a baseline for more in-depth studies on efficacy and safety of these preparations, as well as the possible applications in the public health system.

## Materials and methods

### Plant Collections

**Figure 1:**
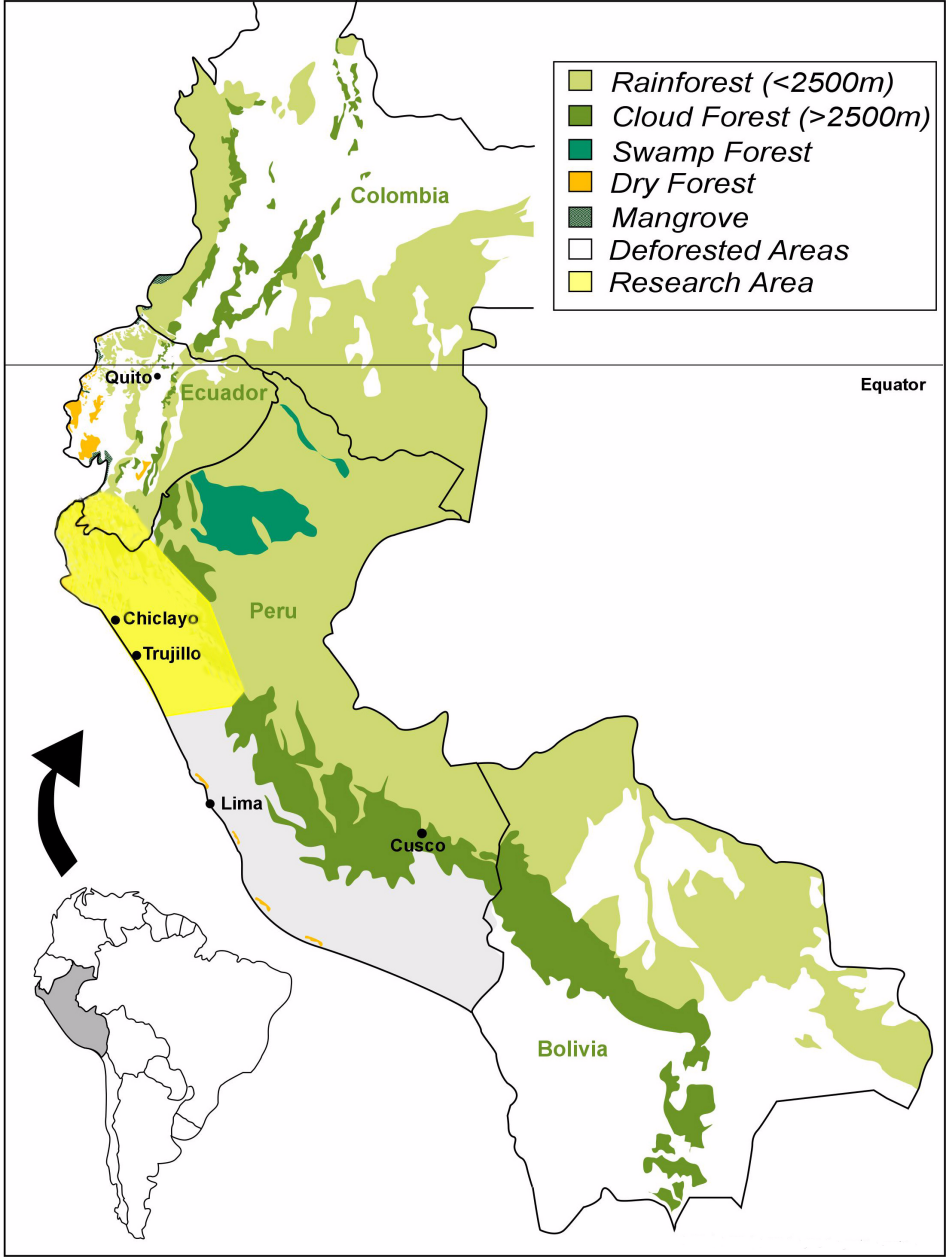
**Research area**.

Plants in the research area in Northern Peru (Fig. 1) were collected in the field, in markets, and at the homes of traditional healers (*curanderos*) visited in August-September 2001, July-August 2002, July-August 2003, June-August 2004, July-August 2005, July-August 2006, June-August 2007, June-August 2008, March-April 2009 and June-August 2009. The specimens are registered under the collection series "RBU/PL," "ISA," "GER," "JULS," "EHCHL," "VFCHL," "TRUBH," "ACR," "KMM," "ACT," and "TRUVANERICA," depending on the year of fieldwork and collection location. Surveys were conducted in Spanish by fluent speakers. Semi-structured interviews were conducted with *curanderos*. All were asked to participate, but due to expected resistance information could not collected from everyone.

Vouchers of all specimens were deposited at the Herbario Truxillensis (HUT, Universidad Nacional de Trujillo), and Herbario Antenor Orrego (HAO, Universidad Privada Antenor Orrego Trujillo). In order to recognize Peru's rights under the Convention on Biological Diversity, most notably with regard to the conservation of genetic resources in the framework of a study treating medicinal plants, the identification of the plant material was conducted entirely in Peru. No plant material was exported in any form whatsoever.

#### Nomenclature

The nomenclature of plant families, genera, and species follows the *Catalogue of the Flowering Plants and Gymnosperms of Peru *[21] and the *Catalogue of Vascular Plants of Ecuador *[22]. The nomenclature was compared to the TROPICOS database. Species were identified using the available volumes of the *Flora of Peru *[23], as well as [24-26], and the available volumes of the *Flora of Ecuador *[27], and reference material in the herbaria HUT, HAO, QCA, LOJA and QCNE.

#### Cluster analysis of plant records

The goal of cluster analysis is to group objects together that are similar. Data in the literature and market collections were organized in an Excel spreadsheet that contained species as rows and sources as columns. Individual cells contained qualitative presence/absence data, represented by numerical values "1" or "0." The Excel spreadsheet was imported into NCSS (version 2007) and a (dis)similarity matrix was produced using the Simple Matching Coefficient that measures the degree of similarity/dissimilarity between all pairs of mixtures. Next, a dendrogram (tree) was generated. Since a cluster analysis will always yield clusters, it is necessary to demonstrate how well the analysis represents the original (dis)similarity matrix. To this end, the tree matrix is transformed into a matrix of ultrametric distances and the latter matrix is statistically compared with the original (dis)similarity matrix. The resulting correlation coefficient "r" between both matrices (normalized Mantel statistic Z) can be used as a measure of goodness of fit for cluster analysis. This analysis provided an in-depth comparison of the species composition of all mixtures.

## Results and Discussion

The investigation of plant mixtures used in traditional medicine in Northern Peru yielded a total of 974 herbal preparations used to treat 164 different afflictions (Table 1). The classification of diseases followed the curandero's terminology. To allow a better overview the different disease concepts were grouped in more inclusive disease categories, according to their similarity. Psychosomatic disorders were the most outstanding afflictions treated with traditional herbal mixtures, with almost 30% of all recipes applied, followed by respiratory illnesses, female issues, kidney problems and heart problems (Table 2). *Susto *(fright), problems of the nervous system, general systemic inflammation and bronchitis together accounted for almost 25% of all remedies used. In many cases, healers used only one or two common mixtures to treat an illness (Table 3). This degree of consensus between different healers shows great sophistication in the diagnosis and treatment of specific disorders. On the contrary, when it came to the treatment of unspecific disease categories like "inflammation" or "bronchitis", every healer seemed to use her/his own specific mixture to treat the problem. This was particularly obvious in the treatment of neurological and psychosomatic problems, for which the majority of plants and mixtures was employed. Up to 49 different preparations were used to treat the same disease. This seems to indicate a high degree of experimentation that is still ongoing in order to find a working cure for unspecific symptoms, and that there is very little consent amongst the individual healers as which cure to employ. This low consensus, especially where spiritual and nervous system/psychosomatic aspects are involved, might also indicate that the individual healers are reluctant to exchange knowledge about their dedicated, specific and guarded treatment methodology in these areas, while the knowledge about "simple" treatments is much more widespread.

**Table 1:** Mixtures used in individual disease categories (letter in parenthesis refers to combined category in table 2)

Application	# of mixtures	%	Application	# of mixtures	%	Application	# of mixtures	%
Abortion (L)	1	0.1	Dysentery (G)	1	0.1	Pharyngitis (B)	1	0.1
After Birth (L)	1	0.1	Epilepsy (R)	8	0.82	Pimples (S)	1	0.1
AIDS (Q)	1	0.1	Fertility (L)	6	0.62	Pneumonia (O)	2	0.21
Allergies (J)	3	0.31	Fever (E)	10	1.03	Promoting Lactation (L)	1	0.1
Amoeba Infection (G)	1	0.1	Fibroids (L)	1	0.1	Prostate (C)	16	1.64
Anemia (L)	2	0.21	Food Coloring	1	0.1	Protection (R)	4	0.41
Anger/Moodiness (R)	2	0.21	Forgetting (R)	3	0.31	Pulmonary Disease (O)	2	0.21
Angina Pectoris (K)	1	0.1	Fractures (P)	2	0.21	Rashes (S)	2	0.21
Animal Bites (B)	2	0.21	Fragrance (C)	1	0.1	Recovering (R)	1	0.1
Antibiotic (B)	2	0.21	Fungus (S)	4	0.41	Rehabilitation of Drug Addicts (R)	1	0.1
Antiseptic (B)	1	0.1	Gallbladder (D)	12	1.24	Relaxation (R)	3	0.31
Anxiety (R)	13	1.33	Gases (A)	9	0.92	Renal Bleeding (M)	1	0.1
Aphrodisiac (C)	1	0.1	Gastritis (A)	5	0.51	Renal Disease (M)	4	0.41
Arthritis (J)	16	1.64	Good Business, Health (R)	1	0.1	Rheumatism (J)	8	0.82
Asthma (J)	16	1.64	Hair loss (C)	4	0.42	Scars (S)	1	0.1
Bad Air - Mal Aire (R)	22	2.25	Hallucinogen (R)	4	0.42	Sexual Potency (C)	5	0.52
Bad Breath (C)	2	0.21	Hangover (R)	2	0.21	Sharp Pain (internal) (I)	1	0.1
Baldness(C)	1	0.1	Headache (I)	4	0.41	Sinusitis (O)	5	0.52
Bladder (M)	7	0.72	Heart (K)	29	2.98	Skin (S)	6	0.62
Blood (K)	23	2.36	Hemorrhages (A)	9	0.92	Skin Marks (S)	1	0.1
Blood Pressure (high) (K)	4	0.41	Hemorrhoids (A)	1	0.1	Sleep Aid (R)	1	0.1
Blood Pressure (Low) (K)	4	0.41	Hepatitis (D)	6	0.62	Snake Bite	1	0.1
Blood purification (K)	4	0.41	Hyperactivity (R)	1	0.1	Sorcery (R)	11	1.13
Boils (S)	1	0.1	Indigestion (A)	2	0.21	Sores (S)	1	0.1
Bone and muscular pain (I)	15	1.54	Infection (B)	6	0.62	Speech Impediment (R)	1	0.1
Bones (P)	2	0.21	Inflammation (N)	41	4.21	Sterilization for Women (L)	2	0.21
Brain (R)	2	0.21	Inflammation of the Bladder (M)	1	0.1	Stomach (A)	11	1.13
Bronchitis (O)	41	4.21	Inflammation of the Kidneys (M)	23	2.36	Stomach Pain (I)	3	0.31
Bruises (I)	3	0.31	Inflammation of the Liver (D)	6	0.62	Stress (R)	1	0.1
Bumps (I)	1	0.1	Inflammation of the Lungs (O)	1	0.1	Susto (R)	51	5.23
Burn Fat (K)	2	0.21	Inflammation of the Ovaries (L)	8	0.82	Swelling (I)	1	0.1
Cancer (H)	7	0.72	Inflammation of the Stomach (A)	2	0.21	Tachycardia (K)	2	0.21
Cancerous wounds (H)	1	0.1	Inflammation of the Tonsils (B)	1	0.1	Tapeworm (G)	1	0.1
Chills (E)	2	0.21	Inflammation of the Womb (L)	1	0.1	Tension (R)	1	0.1
Cholera (Q)	2	0.21	Inflammation of urinary tract (M)	2	0.21	Throat (B)	2	0.21
Cholesterol (K)	2	0.21	Inflammation of Uterus (L)	8	0.82	Tranquility (R)	1	0.1
Cleansing (R)	2	0.21	Insomnia (R)	18	1.85	Tuberculosis (B)	2	0.21
Cold (high mucus) (O)	1	0.1	Internal Bleeding (A)	1	0.1	Tumors (H)	4	0.41
Cold Sores (Q)	1	0.1	Intestine (A)	3	0.31	Twisted Bones (R)	1	0.1
Colds (O)	25	2.57	Judgment (R)	1	0.1	Typhoid (B)	1	0.1
Colic (A)	12	1.23	Kidneys (M)	24	2.46	Ulcers (A)	6	0.62
Colic of the stomach (A)	2	0.21	Laxative (A)	2	0.21	Urinary Infections (M)	2	0.21
Concussions (I)	2	0.21	Liver (D)	30	3.01	Urinary Problems (M)	7	0.72
Congestion (A)	1	0.1	Luck (R)	15	1.54	Uterus (L)	3	0.31
Contraceptive (L)	4	0.41	Lungs (O)	1	0.1	Uterus (cancer) (H)	2	0.21
Cough (O)	22	2.26	Mal Aire (R)	12	1.23	Vaginal cleansing (L)	4	0.41
Cysts (L)	5	0.51	Malaria (E)	2	0.21	Vaginal discharge (L)	2	0.21
Daño (R)	20	2.05	Menstrual regulation (L)	13	1.33	Vomiting (G)	1	0.1
Dengue (Q)	1	0.1	Nausea (G)	1	0.1	Warts (S)	1	0.1
Depression (R)	10	1.03	Nerves (R)	49	5.03	Wounds (B)	20	2.05
Detoxification (R)	1	0.1	Nervousness (R)	2	0.21	Yellow Fever (Q)	3	0.31
Detoxification of alcohol drugs (R)	1	0.1	Nostalgic Anxiety/Emotional Trauma (R)	2	0.21			
Diabetes (F)	11	1.12	Ovaries (L)	2	0.21			
Diarrhea (G)	9	0.92	Pain (I)	2	0.21			
Dizziness (R)	1	0.1	Pain of Love (R)	6	0.62			
Domination (R)	1	0.1	Parasites (G)	2	0.21			

**Table 2:** Number of mixtures per disease category (letter in parenthesis refers to individual category in table 1)

Application	Number of mixtures used	%
Colic/Intestinal problems (A)	66	6.68
Wounds/external infections (B)	38	3.90
Prostate and other male issues (C)	30	3.08
Gall and Liver ailments (D)	64	6.57
Malaria and Fever (E)	14	1.44
Diabetes (F)	11	1.13
Diarrhea (including parasites) (G)	16	1.64
Cancer (H)	14	1.44
Pain relief (I)	32	3.29
Arthritis, Rheumatism, Asthma, auto-immune (J)	43	4.41
Heart (K)	71	7.29
Female issues (L)	64	6.57
Kidney and urinary tract (M)	76	7.80
Imflammation (N)	41	4.21
Respiratory tract (O)	100	10.27
Bones (P)	4	0.42
Viral infections (HIV, Dengue, Yellow Fever etc.) (Q)	8	0.81
Psychosomatic problems (R)	263	27.00
Skin problems	18	1.85

**Table 3:** Number of mixtures per application

# of mixtures	# of applications	%
1	56	34.15
2	36	21.95
3	8	4.88
4	12	7.31
5	4	2.44
6	7	4.23
7	3	1.83
8	4	2.44
9	3	1.83
10	2	1.22
11	3	2.44
12	3	1.83
13	2	1.22
14	0	0
15	2	1.22
16	3	1.83
17	0	0
18	1	0.61
19	0	0
20	2	1.22
21	0	0
22	2	1.22
23	2	1.22
24	1	0.61
25	1	0.61
26	0	0
27	0	0
28	0	0
29	1	0.61
30	1	0.61
31	0	0
32	0	0
33	0	0
34	0	0
35	0	0
36	0	0
37	0	0
38	0	0
39	0	0
40	0	0
41	2	1.22
42	0	0
43	0	0
44	0	0
45	0	0
46	0	0
47	0	0
48	0	0
49	1	0.61
50	0	0
51	1	0.61

Altogether 330 plant species, representing almost 65% of the medicinal flora used in the region (Bussmann & Sharon 2006) were applied in mixtures. Of these, 64 species (19.39%) were introductions, which falls within the range of introduced species as percentage of the whole medicinally used flora. Amongst the plants employed, Asteraceae expectedly stood out, and the number of species of this family used was comparable to the percentage of Asteraceae in the medicinal flora of the region (Bussmann & Sharon 2006, Table 4). The overwhelming number of plant mixtures contained 2-7 different plant species, although in the most extreme case 27 distinct species were included (Table 5). A large number of species appeared in various mixtures. For the most important representatives. A complete overview on all plant mixtures used for all illness categories is given in Additional file 1. The plant species for each mixture are listed in the order given by the *curanderos *in order to express the importance of the individual species, rather than providing an alphabetical listing. For a detailed overview on quantities and parts of each plant use see [8]. A complete taxonomic overview can be found in Additional file 2.

**Table 4:** Number of species per family

Family	# of species	%	Family	# of species	%
Asteraceae	48	14.16	Aizoaceae	1	0.295
Lamiaceae	22	6.49	Amaryllidaceae	1	0.295
Fabaceae	17	5.01	Annonaceae	1	0.295
Solanaceae	15	4.44	Aquifoliaceae	1	0.295
Piperaceae	10	2.95	Araliaceae	1	0.295
Apiaceae	9	2.65	Arecaceae	1	0.295
Rosaceae	8	2.36	Aristolochiaceae	1	0.295
Euphorbiaceae	8	2.36	Asclepiadaceae	1	0.295
Amaranthaceae	6	1.77	Balanophoraceae	1	0.295
Lycopodiaceae	6	1.77	Berberidaceae	1	0.295
Poaceae	6	1.77	Bixaceae	1	0.295
Rutaceae	6	1.77	Burseraceae	1	0.295
Orchidaceae	5	1.47	Capparidaceae	1	0.295
Plantaginaceae	5	1.47	Chenopodiaceae	1	0.295
Verbenaceae	5	1.17	Chloranthaceae	1	0.295
Anacardiaceae	4	1.18	Chrysobalanaceae	1	0.295
Boraginaceae	4	1.18	Clethraceae	1	0.295
Bromeliaceae	4	1.18	Crassulaceae	1	0.295
Cucurbitaceae	4	1.18	Dipsacaceae	1	0.295
Ericaceae	4	1.18	Elaeocarpaceae	1	0.295
Gentianaceae	4	1.18	Ephedraceae	1	0.295
Geraniaceae	4	1.18	Erythroxylaceae	1	0.295
Lauraceae	4	1.18	Grossulariaceae	1	0.295
Myrtaceae	4	1.18	Illiciaceae	1	0.295
Polypodiaceae	4	1.18	Isoetaceae	1	0.295
Valerianaceae	4	1.18	Juglandaceae	1	0.295
Apocynaceae	3	0.85	Lythraceae	1	0.295
Caryophyllaceae	3	0.85	Malesherbiaceae	1	0.295
Convolvulaceae	3	0.85	Melastomataceae	1	0.295
Lobeliaceae	3	0.85	Meliaceae	1	0.295
Malvaceae	3	0.85	Moraceae	1	0.295
Onagraceae	3	0.85	Myricaceae	1	0.295
Portulacaceae	3	0.85	Myristicaceae	1	0.295
Rubiaceae	3	0.85	Oxalidaceae	1	0.295
Urticaceae	3	0.85	Papaveraceae	1	0.295
Cyperaceae	3	0.85	Phytolaccaceae	1	0.295
Brassicaceae	2	0.59	Polygalaceae	1	0.295
Bignoniaceae	2	0.59	Polygonaceae	1	0.295
Caprifoliaceae	2	0.59	Proteaceae	1	0.295
Clusiaceae	2	0.59	Punicaceae	1	0.295
Dioscoreaceae	2	0.59	Ranunculaceae	1	0.295
Equisetaceae	2	0.59	Santalaceae	1	0.295
Liliaceae	2	0.59	Smilacaceae	1	0.295
Linaceae	2	0.59	Thelypteridaceae	1	0.295
Loganiaceae	2	0.59	Thymeleaceae	1	0.295
Loranthaceae	2	0.59	Tiliaceae	1	0.295
Monimiaceae	2	0.59	Tropaeolaceae	1	0.295
Nyctaginaceae	2	0.59	Typhaceae	1	0.295
Olacaceae	2	0.59	Ulmaceae	1	0.295
Passifloraceae	2	0.59	Violaceae	1	0.295
Polemoniaceae	2	0.59	Xyridaceae	1	0.295
Salicaceae	2	0.59	Zingiberaceae	1	0.295
Scrophulariaceae	2	0.59	Zygophyllaceae	1	0.295
Acanthaceae	1	0.295			
Adiantaceae	1	0.295			

**Table 5:** Number of mixtures w/number of plants

# plants per mixture	# of mixtures	%
2	81	8.38
3	113	11.69
4	153	15.82
5	118	12.20
6	126	13.03
7	99	10.24
8	77	7.96
9	68	7.03
10	25	2.28
11	24	2.48
12	17	1.76
13	7	0.72
14	15	1.55
15	2	0.21
16	23	2.31
17	2	0.21
18	2	0.21
19	1	0.1
20	3	0.31
21	7	0.72
22	2	0.21
23	1	0.1
24	0	0
25	0	0
26	0	0
27	1	0.1

The cluster analysis confirmed that mixtures used for applications like inflammations, infections and blood purification, as well as cough, cold, bronchitis or other respiratory disorders, or urinary infection and kidney problems had similar floristic compositions. However, a few interesting clusters stood out: Mixtures used for nervous system disorders, anxiety and heart problems often had a similar composition for example, as did mixtures for prostate and bladder problems; kidney problems, gallbladder disorders, diabetes and cholesterol were treated with the same preparations; as were rheumatic illnesses and asthma. Our research suggests that this indicates that the local healers have a very detailed understanding of disease concepts, and are choosing their remedies very carefully, based on what underlying cause they diagnose, i.e. heart problems get treated differently if they are caused by stress, versus a physical agent, kidney infections are treated differently from kidney problems linked to diabetes and/or obesity. A complete dendrogram is given in Additional file 3, and a complete overview on the number of applications for all species is given in Additional file 4.

## Conclusions

The floristic composition as well as the complex phytochemistry of traditional herbal mixtures remain woefully understudied. This is the more surprising as traditional one-plant one single-compound based drug discovery efforts have yielded very little results in the last decades, and might in fact be an explanation as to why so many plant species that have been documented for a certain use, are "inefficient" or "toxic" when introduced to clinical trials.

Our research indicates that a large number of plants used in traditional healing in Northern Peru are employed in often sophisticated mixtures, rather than as individual plants. Peruvian *curanderos *appear to employ very specific guidelines in the preparation of these cocktails, and seem to have a clear understanding of disease concepts when they diagnose a patient, which in turn leads them to often apply specific mixtures for specific conditions. There seems to be a widespread exchange of knowledge about mixtures for treatment of bodily diseases, while mixtures for spiritual, nervous system and psychosomatic disorders appear to be more closely guarded by the individual healers.

Traditional herbal mixtures, with their wealth of compound fragments and new compounds originating in the preparation process, could well yield new clues to the treatment of a wide variety of disease. The present paper provides detailed baseline information on composition and use of traditional mixtures in Northern Peru, and further studies to compare the compound composition of these preparations versus single plant extracts, as well as investigations comparing efficacy and toxicity of herbal preparations versus their single plant ingredients are in progress.

## Competing interests

The authors declare that they have no competing interests.

## Authors' contributions

RB collected/identified plant material under the voucher acronyms "RBU/PL," "ISA," "GER," "JULS," "EHCHL," "VFCHL," "TRUBH", and "TRUVANERICA, and conducted the statistical analysis of the data as well as writing the manuscript. AG conducted laboratory work, data analysis and manuscript composition. AK, KM and AT collected and identified the plant material under "ACR," "KMM," and "ACT," and revised the plant nomenclature of the manuscript. All authors have read and approved the final manuscript.

## Supplementary Material

Additional file 1 Disease categories and mixtures used for treatment. Click here for file

Additional file 2 Scientific plant names and collection numbers. Click here for file

Additional file 3 Dendrograms. Click here for file

Additional file 4 Most important plant species. Click here for file
