# Supplementary material for: Herbal mixtures in traditional medicine in Northern Peru
Source: J Ethnobiol Ethnomed. 2010 Mar 14;6:10. doi: 10.1186/1746-4269-6-10 (PMC2848642; doi:10.1186/1746-4269-6-10)
Supplement: Additional file 1 — Disease categories and mixtures used for treatment. [file 1746-4269-6-10-S1.PDF]

Additional file 1. Plants used in herbal mixtures (number refers to mixture number in dendrogram, see additional file 3)

Abortion

1. *Ruta graveolens* L., *Lepechinia meyenii* (Walp.) Epling, *Salvia officinalis* L., *Salvia cuspidata* Ruiz & Pav., *Salvia sagittata* Ruiz. & Pav., *Origanum vulgare* L., *Schinus molle* L., *Eucalyptus globulus* Labill., *Ambrosia peruviana* Willd., *Artemisia absinthium* L., *Adiantum concinnum* Humb. & Bonpl. Ex Willd., *Gentianella bicolor* (Wedd.) J.S. Pringle

After Birth

2. *Foeniculum vulgare* Mill., *Matricaria frigidum* (H.B.K.) Kunth, *Matricaria recutita* L., *Ambrosia peruviana* Willd., *Mentha x piperita* L., *Scutellaria scutellarioides* (Kunth) Harley, *Lippia integrifolia* (Griseb.) Hieron., *Melissa officinalis* L., *Sanguisorba minor* Scop., *Dianthus caryophyllus* L., *Borago officinalis* L.

AIDS

3. *Uncaria tomentosa* (Willd. ex Roem. & Schult.) DC., *Phyllanthus niruri* L., *Phyllanthus stipulatus* (Raf.) G.L.Webster, *Phyllanthus urinaria* L., *Linum sativum* L., *Linum usitatissimum* L., *Peumus boldus* Molina, *Cordia lutea* Lam., *Capsella bursa-pastoris* (L.) Medik.

Allergies

4. *Alternanthera porrigens* (Jacq.) Kuntze, *Schkuhria pinnata* (Lam.) Kuntze ex Thell, *Polygala paniculata* L., *Alternanthera halimifolia* (Lam.) Standl. Ex Pittier, *Iresine herbstii* Hook, *Adiantum concinnum* Humb. & Bonpl. Ex Willd., *Cuphea strigulosa* Kunth, *Smilax medica* Schltdl. & Cham., *Rubus robustus* C. Presl.
5. *Uncaria tomentosa* (Willd. ex Roem. & Schult.) DC., *Phyllanthus niruri* L., *Phyllanthus stipulatus* (Raf.) G.L.Webster, *Phyllanthus urinaria* L., *Linum sativum* L., *Linum usitatissimum* L., *Peumus boldus* Molina, *Cordia lutea* Lam., *Capsella bursa-pastoris* (L.) Medik.
6. *Bejaria aestuans* Mutis ex L., *Buddleja utilis* Kraenzl., *Dioscorea trifida* L.f., *Tiquilia paronychioides* (Phil.) A.T. Richardson., *Ilex guayusa* Loes., *Geranium ayavacense* Willd. ex Kunth, *Geranium sessiliflorum* Cav., *Monactis flaverioides* Kunth, *Malva sylvestris* L., *Alcea rosea* L., *Pelargonium odoratissimum* (L.) L'Hér., *Malva parviflora* L., *Bidens pilosa* L., *Verbena litoralis* Kunth, *Plantago linearis* Kunth, *Plantago major* L., *Equisetum bogotense* Kunth, *Equisetum giganteum* L., *Muehlenbeckia tamnifolia (Kunth) Meisn.*, *Smilax kunthii* Killip & C.V. Morton, *Oreocallis grandiflora* (Lam.) R.Br., *Cinchona officinalis* L.

Amoeba Infection

7. *Caesalpinia spinosa* (Molina) Kuntze, *Nerium oleander* L., *Myrica pubescens* Humb. & Bonpl. ex Willd., *Monactis flaverioides* Kunth *,* *Artemisia absinthium* L., *Achyrocline alata* (Kunth) DC, *Aiouea dubia* (Kunth) Mez, *Nectandra reticulata* (Ruiz & Pav.) Mez

Anemia

8. *Cuphea strigulosa* Kunth, *Peperomia inaequalifolia* Ruiz & Pav., *Dianthus caryophyllus* L., *Lonicera japonica* Thunb. Ex Murray, *Urtica magellanica* Juss. ex Poir., *Urtica urens* L., *Alternanthera halimifolia* (Lam.) Standl. Ex Pittier, *Alternanthera porrigens* (Jacq.) Kuntze, *Pilea microphylla* (L.) Liebm., *Iresine herbstii* Hook., *Erodium cicutarium* (L.) L'Hér. Ex Aiton, *Desmodium molliculum* (Kunth) DC., *Equisetum bogotense* Kunth, *Equisetum giganteum* L., *Verbena litoralis* Kunth, *Sanguisorba minor* Scop., *Buddleja utilis* Kraenzl., *Cynodon dactylon* (L.) Pers., *Bejaria aestuans* Mutis ex L., *Cenchrus echinatus* L., *Tribulus terrestris* L., *Baccharis genistelloides* (Lam.) Pers., *Bidens pilosa* L.
9. *Matricaria recutita* L., *Plantago linearis* Kunth*, Plantago major* L., *Nerium oleander* L., *Myrica pubescens* Humb. & Bonpl. ex Willd.

Anger/Moodiness

10. *Epilobium* sp., *Sanguisorba minor* Scop., *Bejaria aestuans* Mutis ex L., *Cenchrus echinatus* L., *Tribulus terrestris* L., *Baccharis genistelloides* (Lam.) Pers., *Bidens pilosa* L., *Iresine herbstii* Hook., *Alternanthera porrigens* (Jacq.) Kuntze, *Cuphea strigulosa* Kunth
11. *Cheilanthes myriophylla* Desv., *Hesperoxiphion niveum* (Ravenna) Ravenna, *Clethra castaneifolia* Meisn.

Angina Pectoris

12. *Caesalpinia spinosa* (Molina) Kuntze, *Nerium oleander* L., *Myrica pubescens* Humb. & Bonpl. ex Willd, *Monactis flaverioides* Kunth *,* *Solanum americanum* Mill., *Artemisia absinthium* L., *Achyrocline alata* (Kunth) DC, *Aiouea dubia* (Kunth) Mez, *Nectandra reticulata* (Ruiz & Pav.) Mez

Animal Bites

13. *Caesalpinia spinosa* (Molina) Kuntze, *Rosmarinus officinalis* L., *Salvia rosmarinifolia* G. Don., *Erythroxylum coca* Lam., *Croton draconoides* Müll. Arg., *Croton lechleri* Müll. Arg.
14. *Caesalpinia spinosa* (Molina) Kuntze, *Nerium oleander* L., *Myrica pubescens* Humb. & Bonpl. ex Willd., *Monactis flaverioides* Kunth, *Solanum americanum* Mill., *Plantago sericea* Ruiz & Pav., *Artemisia absinthium* L., *Achyrocline alata* (Kunth) DC, *Aiouea dubia* (Kunth) Mez., *Nectandra reticulata* (Ruiz & Pav.) Mez

Antibiotic

15. *Caesalpinia spinosa* (Molina) Kuntze, *Rosmarinus officinalis* L., *Salvia rosmarinifolia* G. Don., *Erythroxylum coca* Lam., *Croton draconoides* Müll. Arg., *Croton lechleri* Müll. Arg.

16. *Caesalpinia spinosa* (Molina) Kuntze, *Nerium oleander* L., *Myrica pubescens* Humb. & Bonpl. ex Willd., *Monactis flaverioides* Kunth, *Solanum americanum* Mill., *Plantago sericea* Ruiz & Pav., *Artemisia absinthium* L., *Achyrocline alata* (Kunth) DC, *Aiouea dubia* (Kunth) Mez, *Nectandra reticulata* (Ruiz & Pav.) Mez

Antiseptic

17. *Leucaena leucocephala* (Lam.) de Wit, *Verbena litoralis* Kunth, *Cestrum auriculatum* L'Hér., *Plantago linearis* Kunth, *Plantago major* L., *Equisetum bogotense* Kunth, *Equisetum giganteum* L., *Mauria heterophylla* Kunth

Anxiety

18. *Alternanthera halimifolia* (Lam.) Standl. Ex Pittier, *Melissa officinalis* L., *Matricaria frigidum* (H.B.K.) Kunth, *Matricaria recutita* L., *Ambrosia peruviana* Willd., *Rosmarinus officinalis* L., *Salvia rosmarinifolia* G. Don., *Foeniculum vulgare* Mill., *Marrubium vulgare* L., *Cydonia oblonga* Mill.
19. *Apium graveolens* L., *Matricaria frigidum* (H.B.K.) Kunth, *Matricaria recutita* L., *Ambrosia peruviana* Willd., *Origanum majorana* L., *Adiantum concinnum* Humb. & Bonpl. Ex Willd.
20. *Borago officinalis* L., *Oritrophium peruvianum* (Lam.) Cuatrec., *Senecio canescens* (Bonpl.) Cuatrec.
21. *Tillandsia cacticola* L.B. Sm., *Matricaria frigidum* (H.B.K.) Kunth, *Matricaria recutita* L., *Ambrosia peruviana* Willd., *Bursera graveolens* (Kunth) Triana & Planch., *Lavandula angustifolia* Mill.
22. *Lathyrus odoratus* L., *Melissa officinalis* L., *Sanguisorba minor* Scop., *Origanum majorana* L., *Lippia integrifolia* (Griseb.) Hieron.
23. *Origanum majorana* L., *Alternanthera halimifolia* (Lam.) Standl. Ex Pittier, *Peperomia inaequalifolia* Ruiz & Pav., *Melissa officinalis* L., *Matricaria frigidum* (H.B.K.) Kunth, *Matricaria recutita* L., *Ambrosia peruviana* Willd., *Foeniculum vulgare* Mill., *Hyptis sidifolia* (L'Hér.) Briq., *Ocimum basilicum* L., *Mentha x piperita* L., *Scutellaria scutellarioides* (Kunth) Harley, *Lippia integrifolia* (Griseb.) Hieron.
24. *Passiflora caerulea* L., *Melissa officinalis* L., *Sanguisorba minor* Scop., *Marrubium vulgare* L., *Rosmarinus officinalis* L., *Salvia rosmarinifolia* G. Don.
25. *Peperomia fraseri* C. DC., *Huperzia* sp., *Valeriana plantaginea* Kunth, *Huperzia* cf.*columnaris* B. Øllg., *Phyllactis rigida* (Ruiz & Pav.) Pers., *Gentianella bicolor* (Wedd.) J.S. Pringle, *Senecio chionogeton* Wedd., *Solanum* sp., *Niphogeton dissecta* (Benth.) J.F. Macbr., *Werneria villosa* A.Gray, *Hypericum laricifolium* Juss.,
26. *Peperomia fraseri* C. DC., *Tillandsia cacticola* L.B. Sm., *Tillandsia multiflora* var. *decipiens* (André) L.B. Sm., *Echeveria peruviana* Meyen, *Melissa officinalis* L., *Sanguisorba minor* Scop., *Rosmarinus officinalis* L., *Salvia rosmarinifolia* G. Don., *Origanum majorana* L., *Viola tricolor* L., *Lathyrus odoratus* L.
27. *Peperomia inaequalifolia* Ruiz & Pav., *Peperomia galioides* Kunth, *Melissa officinalis* L., *Sanguisorba minor* Scop., *Origanum majorana* L., *Viola tricolor* L., *Lathyrus odoratus* L.
28. *Sanguisorba minor* Scop., *Bejaria aestuans* Mutis ex L., *Cenchrus echinatus* L., *Tribulus terrestris* L., *Baccharis genistelloides* (Lam.) Pers., *Bidens pilosa* L., *Alternanthera porrigens* (Jacq.) Kuntze, *Cuphea strigulosa* Kunth, *Melissa officinalis* L., *Peperomia inaequalifolia* Ruiz & Pav., *Dianthus carthusianorum* L., *Matricaria frigidum* (H.B.K.) Kunth, *Matricaria recutita* L., *Ambrosia peruviana* Willd., *Cestrum nocturnum* L.
29. *Citrus sinensis* (L.) Osbeck, *Melissa officinalis* L., *Dianthus caryophyllus* L., *Tagetes erecta* L., *Matricaria frigidum* (H.B.K.) Kunth, *Matricaria recutita* L., *Ambrosia peruviana* Willd., *Origanum majorana* L., *Desmodium molliculum* (Kunth) DC., *Minthostachys mollis* (Kunth) Griseb., *Rosmarinus officinalis* L., *Salvia rosmarinifolia* G. Don.
30. *Populus deltoides* W. Bartram ex Marshall, *Matricaria frigidum* (H.B.K.) Kunth, *Matricaria recutita* L., *Ambrosia peruviana* Willd., *Melissa officinalis* L., *Sanguisorba minor* Scop., *Foeniculum vulgare* Mill., *Marrubium vulgare* L., *Cydonia oblonga* Mill.

Aphrodisiac

31. *Ruta graveolens* L., *Gentianella bicolor* (Wedd.) J.S. Pringle, *Lepechinia meyenii* (Walp.) Epling, *Salvia officinalis* L., *Salvia cuspidata* Ruiz & Pav., *Salvia sagittata* Ruiz. & Pav., *Origanum vulgare* L., *Schinus molle* L., *Eucalyptus globulus* Labill., *Ambrosia peruviana* Willd., *Artemisia absinthium* L., *Adiantum concinnum* Humb. & Bonpl. Ex Willd.

Arthritis

32. *Baccharis latifolia* (Ruiz & Pav.) Pers., *Matricaria recutita* L., *Monactis flaverioides* Kunth, *Solanum americanum* Mill., *Plantago sericea* Ruiz & Pav., *Nerium oleander* L., *Myrica pubescens* Humb. & Bonpl. ex Willd.
33. *Equisetum giganteum* L., *Verbena litoralis* Kunth, *Matricaria frigidum* (H.B.K.) Kunth, *Matricaria recutita* L., *Ambrosia peruviana* Willd., *Mauria heterophylla* Kunth, *Eugenia obtusifolia* Cambess., *Zea mays* L., *Plantago sericea* subsp. *sericans* (Pilg.) Rahn, *Rorippa nasturtium-aquaticum* (L.) Hayek, *Desmodium molliculum* (Kunth) DC., *Dioscorea trifida* L.f.
34. *Gaultheria reticulata* Kunth, *Brugmansia candida* Pers., *Brugmansia arborea* (L.) Lagerh., *Brugmansia sanguinea* (Ruiz & Psv.) D. Don.,
35. *Spartium junceum* L., *Gaultheria reticulata* Kunth
36. *Juglans neotropica* Diels, *Siparuna muricata* (Ruiz & Pav.) A. DC., *Trixis cacalioides* (Kunth) D. Don, *Oreocallis grandiflora* (Lam.) R.Br., *Achyrocline alata* (Kunth) DC, *Sambucus peruviana* Kunth
37. *Allium sativum* L., *Eucalyptus globulus* Labill., *Laccopetalum giganteum* (Wedd.) Ulbr., *Heisteria acuminata* (Humb. & Bonpl.) Engl., *Zingiber officinale* Roscoe
38. *Siparuna muricata* (Ruiz & Pav.) A. DC., *Monactis flaverioides* Kunth, *Solanum americanum* Mill., *Plantago sericea* Ruiz & Pav., *Achyrocline alata* (Kunth) DC, *Aiouea dubia* (Kunth) Mez., *Nectandra reticulata* (Ruiz & Pav.) Mez, *Rosmarinus officinalis* L., *Salvia rosmarinifoila* G. Don., *Ruta graveolens* L., *Achyrocline alata* (Kunth) DC, *Escallonia pendula* (Ruiz & Pav.) Pers., *Bursera graveolens* (Kunth) Triana & Planch.
39. *Brosimum rubescens* Taub., *Heisteria acuminata* (Humb. & Bonpl.) Engl., *Cinchona* sp.
40. *Heisteria acuminata* (Humb. & Bonpl.) Engl., *Eucalyptus globulus* Labill., *Schinus molle* L., *Capparis crotonoides* Kunth
41. *Fuchsia ayavacensis* Kunth, *Sambucus peruviana* Kunth, *Juglans neotropica* Diels, *Lepechinia meyenii* (Walp.) Epling, *Salvia officinalis* L., *Salvia cuspidata* Ruiz & Pav., *Salvia sagittata* Ruiz. & Pav., *Siparuna muricata* (Ruiz & Pav.) A. DC.
42. *Muehlenbeckia tamnifolia (Kunth) Meisn.*, *Bejaria aestuans* Mutis ex L.

43. *Prunus serotina* Ehrh., *Ambrosia peruviana* Willd., *Artemisia absinthium* L., *Ruta graveolens* L., *Rosmarinus officinalis* L., *Salvia rosmarinifolia* G. Don.
44. *Rubus robustus* C. Presl., *Alternanthera porrigens* (Jacq.) Kuntze, *Alternanthera brasiliana* (L.) Kuntze, *Alternanthera villosa* Kunth, *Alternanthera halimifolia* (Lam.) Standl. Ex Pittier, *Jamesonia rotundifolia* Fée, *Alchemilla nivalis* Kunth, *Bejaria aestuans* Mutis ex L., *Typha angustifolia* L.
45. *Escallonia pendula* (Ruiz & Pav.) Pers., *Oreocallis grandiflora* (Lam.) R.Br., *Vallea stipularis* L.f., *Achyrocline alata* (Kunth) DC, *Artemisia absinthium* L.
46. *Celtis loxensis* C.C. Berg, *Brosimum rubescens* Taub., *Heisteria acuminata* (Humb. & Bonpl.) Engl., *Cinchona officinalis* L., *Corynaea crassa* Hook.f.
47. *Urtica magellanica* Juss. ex Poir., *Oritrophium peruvianum* (Lam.) Cuatrec., *Laccopetalum giganteum* (Wedd.) Ulbr., *Senecio tephrosioides* Turcz., *Alternanthera brasiliana* (L.) Kuntze, *Malesherbia ardens* J.F. Macbr., *Stachys lanata* Jacq., *Gentianella bicolor* (Wedd.) J.S. Pringle, *Baccharis genistelloides* (Lam.) Pers., *Bidens pilosa* L., *Juglans neotropica* Diels, *Schinus molle* L., *Ruta graveolens* L., *Piper aduncum* L.

Asthma

48. *Alternanthera brasiliana* (L.) Kuntze, *Rubus robustus* C. Presl., *Oritrophium peruvianum* (Lam.) Cuatrec., *Laccopetalum giganteum* (Wedd.) Ulbr., *Senecio tephrosioides* Turcz., *Clerodendrum* sp.
49. *Chuquiraga weberbaueri* Tovar, *Eucalyptus globulus* Labill., *Piper aduncum* L., *Gaultheria erecta* Vent., *Desmodium molliculum* (Kunth) DC., *Minthostachys mollis* (Kunth) Griseb., *Cordia lutea* Lam.
50. *Cronquistianthus lavandulifolius* (DC.) R.M. King & H. Rob., *Piper aduncum* L., *Rubus robustus* C. Presl., *Juglans neotropica* Diels, *Lepechinia meyenii* (Walp.) Epling, *Salvia officinalis* L., *Salvia cuspidata* Ruiz & Pav., *Salvia sagittata* Ruiz. & Pav., *Borago officinalis* L., *Salvia discolor* Kunth, *Ambrosia peruviana* Willd., *Miconia salicifolia* (Bonpl. ex Naudin) Naudin, *Oritrophium peruvianum* (Lam.) Cuatrec., *Senecio canescens* (Bonpl.) Cuatrec.
51. *Perezia multiflora* (Bonpl.) Less., *Piper aduncum* L., *Eucalyptus globulus* Labill., *Malesherbia ardens* J.F. Macbr., *Alternanthera brasiliana* (L.) Kuntze, *Jamesonia rotundifolia* Fée, *Stachys lanata* Jacq., *Oritrophium peruvianum* (Lam.) Cuatrec., *Senecio canescens* (Bonpl.) Cuatrec., *Juglans neotropica* Diels, *Laccopetalum giganteum* (Wedd.) Ulbr., *Senecio tephrosioides* Turcz., *Tilia platyphyllos* Scop., *Rubus robustus* C. Presl.
52. *Senecio canescens* (Bonpl.) Cuatrec., *Borago officinalis* L., *Eucalyptus globulus* Labill., *Perezia multiflora* (Bonpl.) Less., *Sonchus oleraceus* L., *Matricaria frigidum* (H.B.K.) Kunth, *Matricaria recutita* L., *Ambrosia peruviana* Willd., *Melissa officinalis* L., *Peperomia inaequalifolia* Ruiz & Pav., *Mentha x piperita* L., *Lippia integrifolia* (Griseb.) Hieron., *Acanthoxanthium spinosum* (L.) Fourr., *Aphelandra cirsioides* Lindau
53. *Oritrophium peruvianum* (Lam.) Cuatrec., *Laccopetalum giganteum* (Wedd.) Ulbr., *Senecio tephrosioides* Turcz., , *Malesherbia ardens* J.F. Macbr., *Alternanthera brasiliana* (L.) Kuntze, *Jamesonia rotundifolia* Fée, *Stachys lanata* Jacq., *Senecio canescens* (Bonpl.) Cuatrec., *Clerodendrum* sp.
54. *Hedyosmum racemosum* (Ruiz & Pav.) G. Don., *Heisteria acuminata* (Humb. & Bonpl.) Engl., *Cinchona officinalis* L.
55. *Gaultheria erecta* Vent., *Oritrophium peruvianum* (Lam.) Cuatrec., *Laccopetalum giganteum* (Wedd.) Ulbr., *Senecio tephrosioides* Turcz., *Perezia multiflora* (Bonpl.) Less., *Eucalyptus globulus* Labill., *Piper aduncum* L., *Malesherbia ardens* J.F. Macbr., *Alternanthera brasiliana* (L.) Kuntze, *Jamesonia rotundifolia* Fée, *Stachys lanata* Jacq.
56. *Satureja pulchella* (Kunth) Briq., *Otholobium glandulosum* (L.) J.W. Grimes, *Matricaria frigidum* (H.B.K.) Kunth, *Matricaria recutita* L., *Ambrosia peruviana* Willd., *Desmodium molliculum* (Kunth) DC., *Minthostachys mollis* (Kunth) Griseb.
57. *Stachys lanata* Jacq., *Lepechinia meyenii* (Walp.) Epling, *Salvia officinalis* L., *Salvia cuspidata* Ruiz & Pav., *Salvia sagittata* Ruiz. & Pav., *Piper aduncum* L., *Rubus robustus* C. Presl.
58. *Malesherbia ardens* J.F. Macbr., *Tilia platyphyllos* Scop., *Leucaena leucocephala* (Lam.) de Wit, *Jacaranda acutifolia* Bonpl., *Oritrophium peruvianum* (Lam.) Cuatrec., *Laccopetalum giganteum* (Wedd.) Ulbr., *Senecio tephrosioides* Turcz.,
59. *Myristica fragrans* Houtt., *Brosimum rubescens* Taub., *Celtis loxensis* C.C. Berg, *Laccopetalum giganteum* (Wedd.) Ulbr., *Corynaea crassa* Hook.f., *Eustephia coccinea* Cav.
60. *Eucalyptus globulus* Labill., *Matricaria frigidum* (H.B.K.) Kunth, *Matricaria recutita* L., *Ambrosia peruviana* Willd., *Piper aduncum* L., *Juglans neotropica* Diels, *Cordia alliodora* (Ruiz & Pav.) Oken, *Acalypha mandonii* Müll. Arg., *Baccharis latifolia* (Ruiz & Pav.) Pers.
61. *Uncaria tomentosa* (Willd. ex Roem. & Schult.) DC., *Phyllanthus niruri* L., *Phyllanthus stipulatus* (Raf.) G.L.Webster, *Phyllanthus urinaria* L., *Linum sativum* L., *Linum usitatissimum* L., *Peumus boldus* Molina, *Cordia lutea* Lam., *Capsella bursa-pastoris* (L.) Medik.
62. *Urtica magellanica* Juss. ex Poir., *Oritrophium peruvianum* (Lam.) Cuatrec., *Laccopetalum giganteum* (Wedd.) Ulbr., *Senecio tephrosioides* Turcz., *Malesherbia ardens* J.F. Macbr., *Alternanthera brasiliana* (L.) Kuntze, *Stachys lanata* Jacq., *Gentianella bicolor* (Wedd.) J.S. Pringle, *Baccharis genistelloides* (Lam.) Pers., *Bidens pilosa* L., *Juglans neotropica* Diels, *Schinus molle* L., *Ruta graveolens* L., *Piper aduncum* L.
63. *Clerodendrum* sp., *Oritrophium peruvianum* (Lam.) Cuatrec., *Laccopetalum giganteum* (Wedd.) Ulbr., *Senecio tephrosioides* Turcz., *Malesherbia ardens* J.F. Macbr., *Alternanthera brasiliana* (L.) Kuntze, *Jamesonia rotundifolia* Fée, *Stachys lanata* Jacq.

Bad Air - Mal Aire

64. *Puya weberbaueri* Mez, *Cestrum auriculatum* L'Hér.
65. *Myroxylon balsamum* (L.) Harms, *Couepia* sp., *Trichilia* sp., *Strychnos* sp., *Achyrocline alata* (Kunth) DC, *Aiouea dubia* (Kunth) Mez., *Nectandra reticulata* (Ruiz & Pav.) Mez, *Thevetia peruviana* (Pers.) K. Schum.
66. *Myroxylon balsamum* (L.) Harms, *Achyrocline alata* (Kunth) DC, *Aiouea dubia* (Kunth) Mez., *Nectandra reticulata* (Ruiz & Pav.) Mez., *Couepia* sp., *Trichillia* sp., *Strychnos* sp., *Myristica fragrans* Houtt., *Aloysia triphylla* Royle, *Nicotiana tabacum* L., *Allium sativum* L., *Myroxylon balsamum* (L.) Harms
67. *Ocimum basilicum* L., *Eucalyptus globulus* Labill., *Schinus molle* L., *Spartium junceum* L., *Porophyllum ruderale* (Jacq.) Cass., *Ruta graveolens* L., *Ambrosia peruviana* Willd.
68. *Ocimum basilicum* L., *Tilia platyphyllos* Scop., *Melissa officinalis* L., *Cymbopogon citratus* (DC.) Stapf, *Aloysia triphylla* Royle

69. *Ocimum basilicum* L., *Rosmarinus officinalis* L., *Salvia rosmarinifolia* G. Don., *Lepechinia meyenii* (Walp.) Epling, *Monactis flaverioides* Kunth, *Malva sylvestris* L., *Alcea rosea* L., *Pelargonium odoratissimum* (L.) L'Hér., *Malva parviflora* L.
70. *Salvia rosmarinifolia* G. Don., *Lepechinia meyenii* (Walp.) Epling, *Salvia officinalis* L., *Salvia cuspidata* Ruiz & Pav., *Salvia sagittata* Ruiz. & Pav., *Origanum majorana* L.
71. *Salvia rosmarinifolia* G. Don., *Bursera graveolens* (Kunth) Triana & Planch., *Eucalyptus globulus* Labill.
72. *Aiouea dubia* (Kunth) Mez., *Achyrocline alata* (Kunth) DC, *Nectandra reticulata* (Ruiz & Pav.) Mez., *Trichillia* sp., *Strychnos* sp.
73. *Aiouea dubia* (Kunth) Mez., *Couepia* sp., *Trichilia* sp., *Strychnos* sp., *Myroxylon balsamum* (L.) Harms, *Myristica fragrans* Houtt., *Thevetia peruviana* (Pers.) K. Schum.
74. *Nectandra floribunda* (Sw.) Nees, *Couepia* sp., *Trichilia* sp., *Strychnos* sp., *Myroxylon balsamum* (L.) Harms, *Myristica fragrans* Houtt., *Thevetia peruviana* (Pers.) K. Schum.
75. *Allium sativum* L., *Eucalyptus globulus* Labill., *Laccopetalum giganteum* (Wedd.) Ulbr., *Heisteria acuminata* (Humb. & Bonpl.) Engl., *Zingiber officinale* Roscoe
76. *Malva sylvestris* L., *Melissa officinalis* L., *Sanguisorba minor* Scop., *Origanum majorana* L., *Viola tricolor* L., *Lathyrus odoratus* L., *Cymbopogon citratus* (DC.) Stapf, *Aloysia triphylla* Royle
77. *Myristica fragrans* Houtt., *Brosimum rubescens* Taub., *Celtis loxensis* C.C. Berg, *Laccopetalum giganteum* (Wedd.) Ulbr., *Corynaea crassa* Hook.f., *Eustephia coccinea* Cav.
78. *Myristica fragrans* Houtt., *Couepia* sp., *Trichilia* sp., *Strychnos* sp., *Myroxylon balsamum* (L.) Harms, *Achyrocline alata* (Kunth) DC, *Nectandra reticulata* (Ruiz & Pav.) Mez, *Thevetia peruviana* (Pers.) K. Schum.
79. *Peperomia galioides* Kunth, *Pelargonium odoratissimum* (L.) L'Hér., *Tillandsia cacticola* L.B. Sm., *Tillandsia multiflora* var. *decipiens* (André) L.B. Sm., *Echeveria peruviana* Meyen, *Pilea microphylla* (L.) Liebm.
80. *Ruta graveolens* L., *Gentianella bicolor* (Wedd.) J.S. Pringle, *Lepechinia meyenii* (Walp.) Epling, *Salvia officinalis* L., *Salvia cuspidata* Ruiz & Pav., *Salvia sagittata* Ruiz. & Pav., *Origanum vulgare* L., *Schinus molle* L., *Eucalyptus globulus* Labill., *Artemisia absinthium* L., *Adiantum concinnum* Humb. & Bonpl. Ex Willd.
81. *Smilax medica* Schltdl. & Cham., *Peperomia inaequalifolia* Ruiz & Pav., *Mauria heterophylla* Kunth, *Piper aduncum* L., *Equisetum bogotense* Kunth, *Equisetum giganteum* L.
82. *Brugmansia arborea* (L.) Lagerh., *Brugmansia candida* Pers., *Gaultheria reticulata* Kunth, *Solanum mammosum* L.
83. *Brugmansia sanguinea* (Ruiz & Pav.) D. Don., *Brugmansia candida* Pers., *Brugmansia arborea* (L.) Lagerh., *Porophyllum ruderale* (Jacq.) Cass., *Gaultheria reticulata* Kunth
84. *Urtica urens* L., *Artemisia absinthium* L., *Salvia discolor* Kunth, *Ambrosia peruviana* Willd., *Miconia salicifolia* (Bonpl. ex Naudin) Naudin
85. *Viola tricolor* L., *Melissa officinalis* L.

Bad Breath

86. *Schkuhria pinnata* (Lam.) Kuntze ex Thell. , *Urtica magellanica* Juss. ex Poir., *Urtica urens* L., *Alternanthera porrigens* (Jacq.) Kuntze, *Cuphea strigulosa* Kunth, *Adiantum concinnum* Humb. & Bonpl. Ex Willd., *Satureja pulchella* (Kunth) Briq., *Bejaria aestuans* Mutis ex L., *Peumus boldus* Molina, *Rorippa nasturtium-aquaticum* (L.) Hayek, *Buddleja utilis* Kraenzl., *Schkuhria pinnata* (Lam.) Kuntze ex Thell., *Polygala paniculata* L.
87. *Tessaria integrifolia* Ruiz & Pav., *Equisetum bogotense* Kunth, *Equisetum giganteum* L., *Verbena litoralis* Kunth, *Mauria heterophylla* Kunth, *Iresine diffusa* Humb. & Bonpl. ex Willd., *Plantago sericea* subsp. *sericans* (Pilg.) Rahn, *Zea mays* L.

Baldness

88. *Baccharis genistelloides* (Lam.) Pers., *Schkuhria pinnata* (Lam.) Kuntze ex Thell., *Polygala paniculata* L., *Verbena litoralis* Kunth, *Bidens pilosa* L., *Equisetum bogotense* Kunth, *Equisetum giganteum* L., *Cuphea strigulosa* Kunth, *Ipomoea batatas* (L.) Lam.

Bladder

89. *Tiquilia paronychioides* (Phil.) A.T. Richardson, *Monactis flaverioides* Kunth, *Malva sylvestris* L., *Alcea rosea* L., *Pelargonium odoratissimum* (L.) L'Hér., *Malva parviflora* L., *Zea mays* L., *Equisetum bogotense* Kunth, *Equisetum giganteum* L., *Pilea microphylla* (L.) Liebm., *Buddleja utilis* Kraenzl., *Bejaria aestuans* Mutis ex L., *Cenchrus echinatus* L., *Tribulus terrestris* L., *Baccharis genistelloides* (Lam.) Pers., *Bidens pilosa* L., *Rorippa nasturtium-aquaticum* (L.) Hayek, *Typha angustifolia* L., *Bixa orellana* L., *Alternanthera porrigens* (Jacq.) Kuntze, *Cuphea strigulosa* Kunth, *Eustephia coccinea* Cav.
90. *Phyllanthus stipulatus* (Raf.) G.L.Webster *Equisetum bogotense* Kunth, *Equisetum giganteum* L., *Plantago linearis* Kunth *,* *Plantago major* L., *Peumus boldus* Molina, *Cordia lutea* Lam., *Lycaste gigantea* Lindl., *Buddleja utilis* Kraenzl., *Tiquilia paronychioides* (Phil.) A.T. Richardson
91. *Pilea microphylla* (L.) Liebm., *Equisetum bogotense* Kunth, *Equisetum giganteum* L., *Bixa orellana* L., *Phyllanthus niruri* L., *Phyllanthus stipulatus* (Raf.) G.L.Webster, *Phyllanthus urinaria* L.
92. *Cordia lutea* Lam., *Plantago linearis* Kunth, *Plantago major* L., *Peumus boldus* Molina
93. *Plantago linearis* Kunth, *Equisetum bogotense* Kunth, *Equisetum giganteum* L., *Mauria heterophylla* Kunth, *Eugenia obtusifolia* Cambess., *Cynodon dactylon* (L.) Pers., *Buddleja utilis* Kraenzl.
94. *Polypodium crassifolium* L., *Desmodium molliculum* (Kunth) DC., *Bidens pilosa* L., *Equisetum bogotense* Kunth, *Equisetum giganteum* L.
95. *Uncaria tomentosa* (Willd. ex Roem. & Schult.) DC., *Phyllanthus niruri* L., *Phyllanthus stipulatus* (Raf.) G.L.Webster, *Phyllanthus urinaria* L., *Linum sativum* L., *Linum usitatissimum* L., *Peumus boldus* Molina, *Cordia lutea* Lam., *Capsella bursa-pastoris* (L.) Medik.

Blood

96. *Alternanthera porrigens* (Jacq.) Kuntze, *Schkuhria pinnata* (Lam.) Kuntze ex Thell., *Polygala paniculata* L., *Alternanthera halimifolia* (Lam.) Standl. Ex Pittier, *Alternanthera brasiliana* (L.) Kuntze, *Jamesonia rotundifolia* Fée, *Iresine herbstii* Hook., *Cuphea strigulosa* Kunth, *Adiantum concinnum* Humb. & Bonpl. Ex Willd., *Smilax medica* Schltdl. & Cham., *Rubus robustus* C. Presl.

97. *Iresine diffusa* Humb. & Bonpl. ex Willd., *Scabiosa atropurpurea* L., *Alternanthera porrigens* (Jacq.) Kuntze, *Cuphea strigulosa* Kunth, *Epilobium* sp., *Brosimum rubescens* Taub.
98. *Iresine herbstii* Hook., *Pilea microphylla* (L.) Liebm.
99. *Petroselinum crispum* (Mill.) Fuss, *Melissa officinalis* L., *Sanguisorba minor* Scop., *Origanum majorana* L., *Tillandsia cacticola* L.B. Sm., *Tillandsia multiflora* var. *decipiens* (André) L.B. Sm., *Echeveria peruviana* Meyen.
100. *Baccharis genistelloides* (Lam.) Pers., *Schkuhria pinnata* (Lam.) Kuntze ex Thell., *Polygala paniculata* L., *Verbena litoralis* Kunth, *Bidens pilosa* L., *Equisetum bogotense* Kunth, *Equisetum giganteum* L., *Cuphea strigulosa* Kunth, *Ipomoea batatas* (L.) Lam.
101. *Bidens pilosa* L., *Mauria heterophylla* Kunth, *Eugenia obtusifolia* Cambess., *Tiquilia paronychioides* (Phil.) A.T. Richardson, *Zea mays* L., *Equisetum bogotense* Kunth, *Equisetum giganteum* L., *Psidium guajava* L., *Sanguisorba minor* Scop., *Cestrum nocturnum* L.
102. *Picrosia longifolia* D. Don, *Verbena litoralis* Kunth, *Schkuhria pinnata* (Lam.) Kuntze ex Thell., *Polygala paniculata* L.
103. *Borago officinalis* L., *Oritrophium peruvianum* (Lam.) Cuatrec., *Senecio canescens* (Bonpl.) Cuatrec.
104. *Sechium edule* (Jacq.) Sw., *Alternanthera halimifolia* (Lam.) Standl. Ex Pittier, *Alternanthera brasiliana* (L.) Kuntze, *Jamesonia rotundifolia* Fée, *Alternanthera porrigens* (Jacq.) Kuntze
105. *Scabiosa atropurpurea* L., *Tilia platyphyllos* Scop., *Malesherbia ardens* J.F. Macbr., *Stachys lanata* Jacq., *Cuphea strigulosa* Kunth, *Alternanthera halimifolia* (Lam.) Standl. Ex Pittier, *Alternanthera brasiliana* (L.) Kuntze, *Jamesonia rotundifolia* Fée, *Alternanthera porrigens* (Jacq.) Kuntze, *Epilobium* sp.
106. *Bejaria aestuans* Mutis ex L., *Buddleja utilis* Kraenzl., *Dioscorea trifida* L.f., *Tiquilia paronychioides* (Phil.) A.T. Richardson, *Ilex guayusa* Loes., *Geranium ayavacense* Willd. ex Kunth, *Geranium sessiliflorum* Cav., *Monactis flaverioides* Kunth, *Malva sylvestris* L., *Alcea rosea* L., *Pelargonium odoratissimum* (L.) L'Hér., *Malva parviflora* L., *Bidens pilosa* L., *Verbena litoralis* Kunth, *Plantago linearis* Kunth, *Plantago major* L., *Equisetum bogotense* Kunth, *Equisetum giganteum* L., *Muehlenbeckia tamnifolia (Kunth) Meisn.*, *Smilax kunthii* Killip & C.V. Morton, *Oreocallis grandiflora* (Lam.) R.Br., *Cinchona officinalis* L.
107. *Phyllanthus niruri* L., *Equisetum bogotense* Kunth, *Equisetum giganteum* L., *Plantago linearis* Kunth, *Plantago major* L., *Peumus boldus* Molina, *Cordia lutea* Lam., *Lycaste gigantea* Lindl., *Buddleja utilis* Kraenzl., *Tiquilia paronychioides* (Phil.) A.T. Richardson
108. *Phyllanthus stipulatus* (Raf.) G.L.Webster, *Equisetum bogotense* Kunth, *Equisetum giganteum* L., *Plantago linearis* Kunth, *Plantago major* L., *Peumus boldus* Molina, *Cordia lutea* Lam., *Lycaste gigantea* Lindl., *Buddleja utilis* Kraenzl., *Tiquilia paronychioides* (Phil.) A.T. Richardson
109. *Spartium junceum* L., *Gaultheria reticulata* Kunth
110. *Trifolium repens* L., *Alternanthera porrigens* (Jacq.) Kuntze, *Cuphea strigulosa* Kunth, *Iresine herbstii* Hook., *Smilax medica* Schltdl. & Cham.
111. *Cuphea strigulosa* Kunth*, Peperomia inaequalifolia* Ruiz & Pav., *Dianthus caryophyllus* L., *Tagetes erecta* L., *Lonicera japonica* Thunb. Ex Murray, *Urtica magellanica* Juss. ex Poir., *Urtica urens* L., *Alternanthera halimifolia* (Lam.) Standl. Ex Pittier, *Alternanthera porrigens* (Jacq.) Kuntze, *Alternanthera brasiliana* (L.) Kuntze, *Jamesonia rotundifolia* Fée, *Pilea microphylla* (L.) Liebm., *Iresine herbstii* Hook., *Erodium cicutarium* (L.) L'Hér. Ex Aiton, *Desmodium molliculum* (Kunth) DC., *Equisetum bogotense* Kunth, *Equisetum giganteum* L., *Verbena litoralis* Kunth, *Sanguisorba minor* Scop., *Buddleja utilis* Kraenzl., *Cynodon dactylon* (L.) Pers., *Bejaria aestuans* Mutis ex L., *Cenchrus echinatus* L., *Tribulus terrestris* L., *Baccharis genistelloides* (Lam.) Pers., *Bidens pilosa* L.
112. *Brosimum rubescens* Taub., *Celtis loxensis* C.C. Berg, *Scabiosa atropurpurea* L.
113. *Passiflora ligularis* Juss., *Peumus boldus* Molina, *Equisetum bogotense* Kunth, *Equisetum giganteum* L., *Mauria heterophylla* Kunth, *Bidens pilosa* L.
114. *Sanguisorba minor* Scop., *Bejaria aestuans* Mutis ex L., *Cenchrus echinatus* L., *Tribulus terrestris* L., *Baccharis genistelloides* (Lam.) Pers., *Bidens pilosa* L., *Alternanthera porrigens* (Jacq.) Kuntze, *Cuphea strigulosa* Kunth, *Melissa officinalis* L., *Peperomia inaequalifolia* Ruiz & Pav., *Dianthus caryophyllus* L., *Matricaria frigidum* (H.B.K.) Kunth, *Matricaria recutita* L., *Ambrosia peruviana* Willd., *Cestrum nocturnum* L.
115. *Uncaria tomentosa* (Willd. ex Roem. & Schult.) DC., *Phyllanthus niruri* L., *Phyllanthus stipulatus* (Raf.) G.L.Webster, *Linum usitatissimum* L, *Peumus boldus* Molina, *Cordia lutea* Lam., *Capsella bursa-pastoris* (L.) Medik.
116. *Celtis loxensis* C.C. Berg, *Brosimum rubescens* Taub., *Heisteria acuminata* (Humb. & Bonpl.) Engl., *Laccopetalum giganteum* (Wedd.) Ulbr., *Heisteria acuminata* (Humb. & Bonpl.) Engl., *Cinchona officinalis* L., *Ipomoea pauciflora* M. Martens & Galeotti, *Corynaea crassa* Hook.f.
117. *Urtica magellanica* Juss. ex Poir., *Oritrophium peruvianum* (Lam.) Cuatrec., *Laccopetalum giganteum* (Wedd.) Ulbr., *Senecio tephrosioides* Turcz., *Malesherbia ardens* J.F. Macbr., *Alternanthera brasiliana* (L.) Kuntze *Stachys lanata* Jacq., *Gentianella bicolor* (Wedd.) J.S. Pringle, *Baccharis genistelloides* (Lam.) Pers., *Bidens pilosa* L., *Juglans neotropica* Diels, *Schinus molle* L., *Ruta graveolens* L., *Piper aduncum* L.
118. *Verbena litoralis* Kunth, *Sonchus oleraceus* L., *Alternanthera halimifolia* (Lam.) Standl. Ex Pittier, *Alternanthera porrigens* (Jacq.) Kuntze, *Alternanthera brasiliana* (L.) Kuntze, *Jamesonia rotundifolia* Fée, *Portulaca oleracea* subsp. *tuberculata* Danin & H.G. Baker, *Portulaca villosa* Cham.

Blood Pressure (high)

119. *Sonchus oleraceus* L., *Iresine herbstii* Hook., *Alternanthera porrigens* (Jacq.) Kuntze, *Cuphea strigulosa* Kunth, *Pilea microphylla* (L.) Liebm.
120. *Spartium junceum* L., *Cordia lutea* Lam.
121. *Erodium cicutarium* (L.) L'Hér. Ex Aiton, *Scabiosa atropurpurea* L., *Cuphea strigulosa* Kunth, *Alternanthera halimifolia* (Lam.) Standl. Ex Pittier
122. *Cestrum auriculatum* L'Hér., *Polylepis racemosa* Ruiz & Pav., *Eucalyptus globulus* Labill., *Salvia rosmarinifolia* G. Don.

Blood Pressure (Low)

123. *Erodium cicutarium* (L.) L'Hér. Ex Aiton, *Scabiosa atropurpurea* L., *Cuphea strigulosa* Kunth, *Alternanthera halimifolia* (Lam.) Standl. Ex Pittier

124. *Ocimum basilicum* L., *Eucalyptus globulus* Labill., *Schinus molle* L., *Spartium junceum* L., *Porophyllum ruderale* (Jacq.) Cass., *Ruta graveolens* L. *Ambrosia peruviana* Willd.
125. *Ocimum basilicum* L., *Tilia platyphyllos* Scop., *Melissa officinalis* L., *Origanum majorana* L., *Cymbopogon citratus* (DC.) Stapf, *Aloysia triphylla* Royle
126. *Ocimum basilicum* L., *Rosmarinus officinalis* L., *Salvia rosmarinifolia* G. Don., *Lepechinia meyenii* (Walp.) Epling, *Cestrum auriculatum* L'Hér., *Monactis flaverioides* Kunth, *Malva sylvestris* L., *Alcea rosea* L., *Pelargonium odoratissimum* (L.) L'Hér., *Malva parviflora* L.

Blood purification

127. *Adiantum concinnum* Humb. & Bonpl. Ex Willd., *Bejaria aestuans* Mutis ex L., *Alternanthera porrigens* (Jacq.) Kuntze, *Alternanthera halimifolia* (Lam.) Standl. Ex Pittier, *Alternanthera brasiliana* (L.) Kuntze, *Jamesonia rotundifolia* Fée, *Cuphea strigulosa* Kunth, *Origanum vulgare* L.
128. *Picrosia longifolia* D. Don, *Verbena litoralis* Kunth, *Schkuhria pinnata* (Lam.) Kuntze ex Thell., *Polygala paniculata* L.
129. *Schkuhria pinnata* (Lam.) Kuntze ex Thell., *Urtica magellanica* Juss. ex Poir., *Urtica urens* L., *Adiantum concinnum* Humb. & Bonpl. Ex Willd., *Satureja pulchella* (Kunth) Briq., *Bejaria aestuans* Mutis ex L., *Peumus boldus* Molina, *Rorippa nasturtium-aquaticum* (L.) Hayek, *Buddleja utilis* Kraenzl., *Polygala paniculata* L.
130. *Cuphea strigulosa* Kunth, *Peperomia inaequalifolia* Ruiz & Pav., *Dianthus caryophyllus* L., *Tagetes erecta* L., *Lonicera japonica* Thunb. Ex Murray, *Urtica magellanica* Juss. ex Poir., *Urtica urens* L., *Alternanthera porrigens* (Jacq.) Kuntze, *Alternanthera halimifolia* (Lam.) Standl. Ex Pittier, *Alternanthera brasiliana* (L.) Kuntze, *Jamesonia rotundifolia* Fée, *Pilea microphylla* (L.) Liebm., *Iresine herbstii* Hook., *Erodium cicutarium* (L.) L'Hér. Ex Aiton, *Desmodium molliculum* (Kunth) DC., *Equisetum bogotense* Kunth, *Equisetum giganteum* L., *Verbena litoralis* Kunth, *Sanguisorba minor* Scop., *Buddleja utilis* Kraenzl., *Cynodon dactylon* (L.) Pers., *Bejaria aestuans* Mutis ex L., *Cenchrus echinatus* L., *Tribulus terrestris* L., *Baccharis genistelloides* (Lam.) Pers., *Bidens pilosa* L.

Boils

131. *Caesalpinia spinosa* (Molina) Kuntze, *Nerium oleander* L., *Myrica pubescens* Humb. & Bonpl. ex Willd., *Monactis flaverioides* Kunth, *Artemisia absinthium* L., *Achyrocline alata* (Kunth) DC., *Aiouea dubia* (Kunth) Mez., *Nectandra reticulata* (Ruiz & Pav.) Mez.

Bone and muscular pain

132. *Loxopterygium huasango* Spruce ex Engl., *Ephedra americana* Humb. & Bonpl. ex Willd., *Psittacanthus chanduyensis* Eichler, *Tristerix longebracteatus* (Desr.) Barlow & Wiens
133. *Baccharis latifolia* (Ruiz & Pav.) Pers., *Matricaria recutita* L., *Monactis flaverioides* Kunth., *Nerium oleander* L., *Myrica pubescens* Humb. & Bonpl. ex Willd.
134. *Hedyosmum racemosum* (Ruiz & Pav.) G. Don., *Heisteria acuminata* (Humb. & Bonpl.) Engl., *Cinchona officinalis* L.
135. *Gaultheria reticulata* Kunth, *Brugmansia candida* Pers., *Brugmansia arborea* (L.) Lagerh., *Brugmansia sanguinea* (Ruiz & Pav.) D. Don
136. *Spartium junceum* L., *Gaultheria reticulata* Kunth
137. *Siparuna muricata* (Ruiz & Pav.) A. DC., *Monactis flaverioides* Kunth, *Achyrocline alata* (Kunth) DC, *Aiouea dubia* (Kunth) Mez., *Nectandra reticulata* (Ruiz & Pav.) Mez., *Rosmarinus officinalis* L., *Salvia rosmarinifolia* G. Don., *Ruta graveolens* L., *Escallonia pendula* (Ruiz & Pav.) Pers., *Bursera graveolens* (Kunth) Triana & Planch.
138. *Brosimum rubescens* Taub., *Heisteria acuminata* (Humb. & Bonpl.) Engl., *Cinchona officinalis* L.
139. *Myristica fragrans* Houtt., *Brosimum rubescens* Taub., *Celtis loxensis* C.C. Berg, *Laccopetalum giganteum* (Wedd.) Ulbr., *Corynaea crassa* Hook.f., *Eustephia coccinea* Cav.
140. *Eucalyptus globulus* Labill., *Sonchus oleraceus* L., *Borago officinalis* L., *Oritrophium peruvianum* (Lam.) Cuatrec., *Senecio canescens* (Bonpl.) Cuatrec., *Matricaria recutita* L., *Rosmarinus officinalis* L., *Salvia rosmarinifolia* G. Don., *Urtica magellanica* Juss. ex Poir.
141. *Heisteria acuminata* (Humb. & Bonpl.) Engl., *Eucalyptus globulus* Labill., *Schinus molle* L., *Capparis crotonoides* Kunth
142. *Cenchrus echinatus* L., *Bidens pilosa* L., *Arctium lappa* L., *Centropogon* cf.*rufus* Wimm.
143. *Muehlenbeckia tamnifolia (Kunth) Meisn.*, *Bejaria aestuans* Mutis ex L.
144. *Prunus serotina* Ehrh., *Ambrosia peruviana* Willd., *Artemisia absinthium* L., *Ruta graveolens* L., *Rosmarinus officinalis* L., *Salvia rosmarinifolia* G. Don.
145. *Escallonia pendula* (Ruiz & Pav.) Pers., *Oreocallis grandiflora* (Lam.) R.Br., *Vallea stipularis* L.f., *Achyrocline alata* (Kunth) DC, *Artemisia absinthium* L.
146. *Celtis loxensis* C.C. Berg, *Brosimum rubescens* Taub., *Laccopetalum giganteum* (Wedd.) Ulbr., *Heisteria acuminata* (Humb. & Bonpl.) Engl., *Cinchona officinalis* L., *Corynaea crassa* Hook.f.

Bones

147. *Tristerix longebracteatus* (Desr.) Barlow & Wiens, *Uncaria tomentosa* (Willd. ex Roem. & Schult.) DC., *Mimosa nothacacia* Barneby, *Ephedra americana* Humb. & Bonpl. ex Willd.
148. *Uncaria tomentosa* (Willd. ex Roem. & Schult.) DC., *Phyllanthus niruri* L., *Phyllanthus stipulatus* (Raf.) G.L.Webster, *Phyllanthus urinaria* L., *Linum sativum* L., *Linum usitatissimum* L. *Peumus boldus* Molina, *Cordia lutea* Lam., *Capsella bursa-pastoris* (L.) Medik.

Brain

149. *Cyclanthera pedata* (L.) Schrad., *Sechium edule* (Jacq.) Sw.
150. *Phyllactis rigida* (Ruiz & Pav.) Pers., *Arnica montana* L., *Senecio pseudotites* Griseb.

Bronchitis

151. *Alternanthera brasiliana* (L.) Kuntze, *Gaultheria erecta* Vent., *Oritrophium peruvianum* (Lam.) Cuatrec., *Laccopetalum giganteum* (Wedd.) Ulbr., *Senecio tephrosioides* Turcz., *Pachyphyllum pasti* Rchb. f., *Clerodendrum* sp.
152. *Mangifera indica* L., *Schinus molle* L., *Eucalyptus globulus* Labill., *Tessaria integrifolia* Ruiz & Pav., *Citrus limon* (L.) Burm. f.
153. *Apium graveolens* L., *Matricaria frigidum* (H.B.K.) Kunth, *Matricaria recutita* L., *Origanum majorana* L., *Adiantum concinnum* Humb. & Bonpl. Ex Willd.
154. *Acanthoxanthium spinosum* (L.) Fourr., *Senna bicapsularis* (L.) Roxb., *Inga edulis* C.Martius, *Inga feuillei* DC.
155. *Ambrosia peruviana* Willd, *Piper aduncum* L., *Borago officinalis* L., *Eucalyptus globulus* Labill., *Oritrophium peruvianum* (Lam.) Cuatrec., *Senecio canescens* (Bonpl.) Cuatrec., *Clerodendrum* sp.
156. *Chuquiraga weberbaueri* Tovar, *Eucalyptus globulus* Labill., *Piper aduncum* L., *Gaultheria erecta* Vent., *Desmodium molliculum* (Kunth) DC., *Minthostachys mollis* (Kunth) Griseb., *Cordia lutea* Lam.
157. *Cronquistianthus lavandulifolius* (DC.) R.M. King & H. Rob., *Piper aduncum* L., *Rubus robustus* C. Presl., *Juglans neotropica* Diels, *Salvia officinalis* L., *Salvia cuspidata* Ruiz & Pav., *Salvia sagittata* Ruiz. & Pav., *Lepechinia meyenii* (Walp.) Epling, *Borago officinalis* L., *Oritrophium peruvianum* (Lam.) Cuatrec., *Senecio canescens* (Bonpl.) Cuatrec., *Clerodendrum* sp.
158. *Perezia multiflora* (Bonpl.) Less., *Piper aduncum* L., *Eucalyptus globulus* Labill., *Alternanthera brasiliana* (L.) Kuntze, *Jamesonia rotundifolia* Fée, *Stachys lanata* Jacq., *Oritrophium peruvianum* (Lam.) Cuatrec., *Senecio canescens* (Bonpl.) Cuatrec., *Juglans neotropica* Diels, *Laccopetalum giganteum* (Wedd.) Ulbr., *Senecio tephrosioides* Turcz.,, *Tilia platyphyllos* Scop., *Rubus robustus* C. Presl.
159. *Paranephelius uniflorus* Poepp., *Buddleja utilis* Kraenzl., *Bejaria aestuans* Mutis ex L., *Tiquilia paronychioides* (Phil.) A.T. Richardson, *Sambucus peruviana* Kunth, *Equisetum bogotense* Kunth, *Equisetum giganteum* L., *Desmodium molliculum* (Kunth) DC.
160. *Bixa orellana* L., *Uncaria tomentosa* (Willd. ex Roem. & Schult.) DC., *Mimosa nothacacia* Barneby
161. *Borago officinalis* L., *Oritrophium peruvianum* (Lam.) Cuatrec., *Senecio canescens* (Bonpl.) Cuatrec.
162. *Cordia alliodora* (Ruiz & Pav.) Oken, *Heisteria acuminata* (Humb. & Bonpl.) Engl., *Cinchona officinalis* L.
163. *Rorippa nasturtium-aquaticum* (L.) Hayek, *Monactis flaverioides* Kunth, *Malva sylvestris* L., *Alcea rosea* L., *Pelargonium odoratissimum* (L.) L'Hér., *Malva parviflora* L., *Desmodium molliculum* (Kunth) DC., *Eugenia obtusifolia* Cambess., *Bidens pilosa* L., *Mauria heterophylla* Kunth, *Iresine diffusa* Humb. & Bonpl. ex Willd., *Plantago sericea* subsp. *sericans* (Pilg.) Rahn, *Tiquilia paronychioides* (Phil.) A.T. Richardson, *Bejaria aestuans* Mutis ex L.
164. *Sambucus peruviana* Kunth, *Ricinus communis* L.
165. *Hedyosmum racemosum* (Ruiz & Pav.) G. Don., *Heisteria acuminata* (Humb. & Bonpl.) Engl., *Cinchona officinalis* L.
166. *Scabiosa atropurpurea* L., *Tilia platyphyllos* Scop., *Malesherbia ardens* J.F. Macbr., *Alternanthera brasiliana* (L.) Kuntze, *Jamesonia rotundifolia* Fée, *Stachys lanata* Jacq., *Cuphea strigulosa* Kunth, *Alternanthera halimifolia* (Lam.) Standl. Ex Pittier, *Alternanthera porrigens* (Jacq.) Kuntze, *Epilobium* sp.
167. *Gaultheria erecta* Vent., *Oritrophium peruvianum* (Lam.) Cuatrec., *Senecio canescens* (Bonpl.) Cuatrec., *Juglans neotropica* Diels, *Laccopetalum giganteum* (Wedd.) Ulbr., *Senecio tephrosioides* Turcz., *Perezia multiflora* (Bonpl.) Less., *Eucalyptus globulus* Labill., *Piper aduncum* L., *Malesherbia ardens* J.F. Macbr., *Alternanthera brasiliana* (L.) Kuntze, *Stachys lanata* Jacq.
168. *Gaultheria reticulata* Kunth, *Brugmansia candida* Pers., *Brugmansia arborea* (L.) Lagerh., *Brugmansia sanguinea* (Ruiz & Pav.) D. Don
169. *Myroxylon balsamum* (L.) Harms, *Couepia* sp., *Trichilia* sp., *Strychnos* sp., *Achyrocline alata* (Kunth) DC, *Aiouea dubia* (Kunth) Mez., *Nectandra reticulata* (Ruiz & Pav.) Mez., *Myristica fragrans* Houtt., *Thevetia peruviana* (Pers.) K. Schum.
170. *Myroxylon balsamum* (L.) Harms, *Aiouea dubia* (Kunth) Mez., *Nectandra reticulata* (Ruiz & Pav.) Mez., *Couepia* sp., *Trichilia* sp., *Strychnos* sp., *Myristica fragrans* Houtt., *Cymbopogon citratus* (DC.) Stapf, *Aloysia triphylla* Royle, *Nicotiana tabacum* L., *Allium sativum* L., *Laccopetalum giganteum* (Wedd.) Ulbr., *Eucalyptus globulus* Labill.
171. *Erodium cicutarium* (L.) L'Hér. Ex Aiton, *Scabiosa atropurpurea* L., *Cuphea strigulosa* Kunth, *Alternanthera halimifolia* (Lam.) Standl. Ex Pittier
172. *Juglans neotropica* Diels, *Piper aduncum* L., *Mikania leiostachya* Benth., *Huperzia reflexa* (Lam.) Trevis., *Borago officinalis* L.
173. *Rosmarinus officinalis* L., *Eucalyptus globulus* Labill., *Equisetum bogotense* Kunth, *Equisetum giganteum* L., *Ruta graveolens* L.
174. *Salvia officinalis* L., *Piper aduncum* L., *Juglans neotropica* Diels, *Eucalyptus globulus* Labill.
175. *Satureja pulchella* (Kunth) Briq., *Otholobium glandulosum* (L.) J.W. Grimes, *Matricaria frigidum* (H.B.K.) Kunth, *Matricaria recutita* L., *Ambrosia peruviana* Willd., *Desmodium molliculum* (Kunth) DC., *Minthostachys mollis* (Kunth) Griseb.
176. *Stachys lanata* Jacq., *Lepechinia meyenii* (Walp.) Epling, *Salvia officinalis* L., *Salvia cuspidata* Ruiz & Pav., *Salvia sagittata* Ruiz. & Pav., *Piper aduncum* L., *Gaultheria erecta* Vent.
177. *Cinnamomum verum* J. Presl., *Piper aduncum* L., *Alternanthera brasiliana* (L.) Kuntze, *Jamesonia rotundifolia* Fée, *Stachys lanata* Jacq., *Clerodendrum* sp., *Oritrophium peruvianum* (Lam.) Cuatrec., *Senecio canescens* (Bonpl.) Cuatrec.
178. *Allium sativum* L., *Piper aduncum* L., *Eucalyptus globulus* Labill., *Oritrophium peruvianum* (Lam.) Cuatrec., *Senecio canescens* (Bonpl.) Cuatrec.
179. *Malesherbia ardens* J.F. Macbr., *Tilia platyphyllos* Scop., *Leucaena leucocephala* (Lam.) de Wit, *Jacaranda acutifolia* Bonpl., *Oritrophium peruvianum* (Lam.) Cuatrec., *Laccopetalum giganteum* (Wedd.) Ulbr., *Senecio tephrosioides* Turcz.,
180. *Malva parviflora* L., *Desmodium molliculum* (Kunth) DC., *Mauria heterophylla* Kunth, *Verbena litoralis* Kunth, *Equisetum bogotense* Kunth, *Equisetum giganteum* L., *Bidens pilosa* L., *Monactis flaverioides* Kunth, *Malva sylvestris* L., *Alcea rosea* L., *Pelargonium odoratissimum* (L.) L'Hér.
181. *Brosimum rubescens* Taub., *Heisteria acuminata* (Humb. & Bonpl.) Engl., *Cinchona officinalis* L.
182. *Myristica fragrans* Houtt., *Celtis loxensis* C.C. Berg, *Laccopetalum giganteum* (Wedd.) Ulbr., *Corynaea crassa* Hook.f., *Eustephia coccinea* Cav.
183. *Eucalyptus globulus* Labill., *Matricaria frigidum* (H.B.K.) Kunth, *Matricaria recutita* L., *Ambrosia peruviana* Willd., *Piper aduncum* L., *Juglans neotropica* Diels, *Cordia alliodora* (Ruiz & Pav.) Oken, *Acalypha mandonii* Müll. Arg., *Baccharis latifolia* (Ruiz & Pav.) Pers.
184. *Piper aduncum* L., *Lepechinia meyenii* (Walp.) Epling, *Perezia multiflora* (Bonpl.) Less., *Oritrophium peruvianum* (Lam.) Cuatrec., *Senecio canescens* (Bonpl.) Cuatrec., *Borago officinalis* L., *Eupatorium gayanum* Wedd.

185. *Piper aduncum* L., *Lepechinia meyenii* (Walp.) Epling, *Eucalyptus globulus* Labill., *Nerium oleander* L., *Myrica pubescens* Humb. & Bonpl. ex Willd., *Verbena litoralis* Kunth, *Ambrosia peruviana* Willd.
186. *Piper nigrum* L., *Eupatorium gayanum* Wedd., *Borago officinalis* L., *Perezia multiflora* (Bonpl.) Less., *Gaultheria erecta* Vent., *Oritrophium peruvianum* (Lam.) Cuatrec., *Senecio canescens* (Bonpl.) Cuatrec., *Malesherbia ardens* J.F. Macbr., *Alternanthera brasiliana* (L.) Kuntze, *Jamesonia rotundifolia* Fée, *Stachys lanata* Jacq., *Cinnamomum verum* J. Presl., *Allium sativum* L.
187. *Plantago linearis* Kunth, *Piper aduncum* L., *Juglans neotropica* Diels, *Oritrophium peruvianum* (Lam.) Cuatrec., *Senecio canescens* (Bonpl.) Cuatrec., *Eucalyptus globulus* Labill.
188. *Uncaria tomentosa* (Willd. ex Roem. & Schult.) DC., *Phyllanthus niruri* L., *Phyllanthus stipulatus* (Raf.) G.L.Webster, *Linum sativum* L., *Linum usitatissimum* L., *Peumus boldus* Molina, *Cordia lutea* Lam., *Capsella bursa-pastoris* (L.) Medik.
189. *Celtis loxensis* C.C. Berg, *Brosimum rubescens* Taub., *Heisteria acuminata* (Humb. & Bonpl.) Engl., *Laccopetalum giganteum* (Wedd.) Ulbr., *Cinchona officinalis* L., *Corynaea crassa* Hook.f.
190. *Clerodendrum* sp., *Oritrophium peruvianum* (Lam.) Cuatrec., *Laccopetalum giganteum* (Wedd.) Ulbr., *Senecio tephrosioides* Turcz., , *Malesherbia ardens* J.F. Macbr., *Alternanthera brasiliana* (L.) Kuntze, *Jamesonia rotundifolia* Fée, *Stachys lanata* Jacq.
191. *Zingiber officinale* Roscoe, *Piper aduncum* L., *Juglans neotropica* Diels, *Malesherbia ardens* J.F. Macbr., *Alternanthera brasiliana* (L.) Kuntze, *Jamesonia rotundifolia* Fée, *Stachys lanata* Jacq.

Bruises

192. *Alternanthera brasiliana* (L.) Kuntze, *Gaultheria erecta* Vent., *Oritrophium peruvianum* (Lam.) Cuatrec., *Laccopetalum giganteum* (Wedd.) Ulbr., *Senecio tephrosioides* Turcz., *Clerodendrum* sp.
193. *Borago officinalis* L., *Oritrophium peruvianum* (Lam.) Cuatrec., *Senecio canescens* (Bonpl.) Cuatrec.
194. *Allium sativum* L., *Eucalyptus globulus* Labill., *Laccopetalum giganteum* (Wedd.) Ulbr., *Heisteria acuminata* (Humb. & Bonpl.) Engl., *Zingiber officinale* Roscoe

Bumps

195. *Alternanthera brasiliana* (L.) Kuntze, *Gaultheria erecta* Vent., *Oritrophium peruvianum* (Lam.) Cuatrec., *Laccopetalum giganteum* (Wedd.) Ulbr., *Senecio tephrosioides* Turcz., , *Clerodendrum* sp.

Burn Fat

196. *Borago officinalis* L., *Oritrophium peruvianum* (Lam.) Cuatrec., *Senecio canescens* (Bonpl.) Cuatrec.
197. *Heisteria acuminata* (Humb. & Bonpl.) Engl., *Eucalyptus globulus* Labill., *Schinus molle* L., *Capparis crotonoides* Kunth

Cancer

198. *Schinus molle* L., *Eucalyptus globulus* Labill., *Ruta graveolens* L., *Dodonaea viscosa* Jacq., *Tilia platyphyllos* Scop.
199. *Annona muricata* L., *Bidens pilosa* L., *Ananas comosus* (L.) Merr., *Bixa orellana* L.
200. *Mirabilis jalapa* L., *Tiquilia paronychioides* (Phil.) A.T. Richardson
201. *Muehlenbeckia tamnifolia (Kunth) Meisn.*, *Bejaria aestuans* Mutis ex L.
202. *Cinchona officinalis* L., *Muehlenbeckia tamnifolia (Kunth) Meisn.*, *Smilax medica* Schltdl. & Cham., *Bejaria aestuans* Mutis ex L.
203. *Uncaria tomentosa* (Willd. ex Roem. & Schult.) DC., *Phyllanthus niruri* L., *Phyllanthus urinaria* L., *Phyllanthus stipulatus* (Raf.) G.L.Webster, *Linum sativum* L., *Linum usitatissimum* L., *Peumus boldus* Molina, *Cordia lutea* Lam., *Capsella bursa-pastoris* (L.) Medik.
204. *Smilax kunthii* Killip & C.V. Morton, *Cinchona officinalis* L., *Bejaria aestuans* Mutis ex L.

Cancerous wounds

205. *Eustephia coccinea* Cav., *Buddleja utilis* Kraenzl., *Bejaria aestuans* Mutis ex L., *Pelargonium odoratissimum* (L.) L'Hér.

Chills

206. *Ipomoea pauciflora* M. Martens & Galeotti, *Cinchona officinalis* L., *Ephedra americana* Humb. & Bonpl. ex Willd., *Loxopterygium huasango* Spruce ex Engl.
207. *Piper aduncum* L., *Lepechinia meyenii* (Walp.) Epling, *Perezia multiflora* (Bonpl.) Less., *Oritrophium peruvianum* (Lam.) Cuatrec., *Senecio canescens* (Bonpl.) Cuatrec., *Borago officinalis* L., *Eupatorium gayanum* Wedd.

Cholera

208. *Sonchus oleraceus* L., *Iresine herbstii* Hook., *Alternanthera porrigens* (Jacq.) Kuntze, *Cuphea strigulosa* Kunth, *Pilea microphylla* (L.) Liebm.
209. *Verbena litoralis* Kunth, *Sonchus oleraceus* L., *Alternanthera halimifolia* (Lam.) Standl. Ex Pittier, *Alternanthera porrigens* (Jacq.) Kuntze, *Alternanthera brasiliana* (L.) Kuntze, *Jamesonia rotundifolia* Fée, *Portulaca oleracea* subsp. *tuberculata* Danin & H.G. Baker, *Portulaca villosa* Cham.

Cholesterol

210. *Baccharis genistelloides* (Lam.) Pers., *Schkuhria pinnata* (Lam.) Kuntze ex Thell., *Polygala paniculata* L., *Verbena litoralis* Kunth, *Bidens pilosa* L., *Equisetum bogotense* Kunth, *Equisetum giganteum* L., *Cuphea strigulosa* Kunth, *Ipomoea batatas* (L.) Lam.
211. *Rubus robustus* C. Presl., *Salvia discolor* Kunth, *Ambrosia peruviana* Willd., *Miconia salicifolia* (Bonpl. ex Naudin) Naudin

Cleansing

212. *Mauria heterophylla* Kunth, *Equisetum bogotense* Kunth, *Equisetum giganteum* L., *Verbena litoralis* Kunth, *Bidens pilosa* L.,
213. *Portulaca oleracea* subsp. *tuberculata* Danin & H.G. Baker, *Peumus boldus* Molina, *Tiquilia paronychioides* (Phil.) A.T. Richardson, *Equisetum bogotense* Kunth, *Equisetum giganteum* L.

Cold (high mucus)

214. *Acmella* cf. *ciliata* (Kunth) Cass., *Mentha spicata* L.

Cold Sores

215. *Sarcostemma clausum* (Jacq.) Schult., *Spartium junceum* L., *Polylepis racemosa* Ruiz & Pav., *Solanum tuberosum* L., *Eucalyptus globulus* Labill.

Colds

216. *Mangifera indica* L., *Schinus molle* L., *Eucalyptus globulus* Labill., *Tessaria integrifolia* Ruiz & Pav., *Citrus limon* (L.) Burm. f.
217. *Ambrosia peruviana* Willd., *Salix chilensis* Molina, *Picrosia longifolia* D. Don, *Tessaria integrifolia* Ruiz & Pav.
218. *Cronquistianthus lavandulifolius* (DC.) R.M. King & H. Rob., *Piper aduncum* L., *Rubus robustus* C. Presl., *Juglans neotropica* Diels, *Lepechinia meyenii* (Walp.) Epling, *Salvia officinalis* L., *Salvia cuspidata* Ruiz & Pav., *Salvia sagittata* Ruiz. & Pav., *Borago officinalis* L., *Salvia discolor* Kunth, *Ambrosia peruviana* Willd., *Miconia salicifolia* (Bonpl. ex Naudin) Naudin, *Oritrophium peruvianum* (Lam.) Cuatrec., *Senecio canescens* (Bonpl.) Cuatrec.
219. *Diplostephium gynoxyoides* Cuatrec., *Picrosia longifolia* D. Don
220. *Borago officinalis* L., *Oritrophium peruvianum* (Lam.) Cuatrec., *Senecio canescens* (Bonpl.) Cuatrec., *Bursera graveolens* (Kunth) Triana & Planch.
221. *Sambucus peruviana* Kunth*, Matricaria frigidum* (H.B.K.) Kunth, *Matricaria recutita* L., *Foeniculum vulgare* Mill., *Ambrosia peruviana* Willd., *Melissa officinalis* L., *Sanguisorba minor* Scop., *Dianthus caryophyllus* L., *Tagetes erecta* L.
222. *Hedyosmum racemosum* (Ruiz & Pav.) G. Don., *Heisteria acuminata* (Humb. & Bonpl.) Engl., *Cinchona officinalis* L.
223. *Ipomoea pauciflora* M. Martens & Galeotti, *Cinchona officinalis* L., *Ephedra americana* Humb. & Bonpl. ex Willd., *Loxopterygium huasango* Spruce ex Engl.
224. *Scirpus californicus* Steud., *Salix chilensis* Molina
225. *Scabiosa atropurpurea* L., *Tilia platyphyllos* Scop., *Malesherbia ardens* J.F. Macbr., *Alternanthera brasiliana* (L.) Kuntze, *Jamesonia rotundifolia* Fée, *Stachys lanata* Jacq., *Cuphea strigulosa* Kunth, *Alternanthera halimifolia* (Lam.) Standl. Ex Pittier, *Alternanthera porrigens* (Jacq.) Kuntze, *Epilobium* sp.
226. *Gaultheria reticulata* Kunth, *Brugmansia candida* Pers., *Brugmansia arborea* (L.) Lagerh., *Brugmansia sanguinea* (Ruiz & Pav.) D.Don
227. *Lavandula angustifolia* Mill., *Rosmarinus officinalis* L., *Salvia rosmarinifolia* G. Don., *Dianthus caryophyllus* L., *Tagetes erecta* L., *Foeniculum vulgare* Mill., *Melissa officinalis* L., *Matricaria recutita* L., *Matricaria frigidum* (H.B.K.) Kunth, *Ambrosia peruviana* Willd., *Sanguisorba minor* Scop.
228. *Otholobium glandulosum* (L.) J.W. Grimes, *Matricaria recutita* L., *Matricaria frigidum* (H.B.K.) Kunth, *Ambrosia peruviana* Willd., *Mentha spicata* L., *Sanguisorba minor* Scop., *Pimpinella anisum* L., *Tagetes filifolia* Lag., *Illicium verum* Hook.f.
229. *Allium sativum* L., *Piper aduncum* L., *Sonchus oleraceus* L., *Eucalyptus globulus* Labill., *Oritrophium peruvianum* (Lam.) Cuatrec., *Senecio canescens* (Bonpl.) Cuatrec.
230. *Malesherbia ardens* J.F. Macbr., *Tilia platyphyllos* Scop., *Leucaena leucocephala* (Lam.) de Wit, *Jacaranda acutifolia* Bonpl., *Oritrophium peruvianum* (Lam.) Cuatrec., *Laccopetalum giganteum* (Wedd.) Ulbr., *Senecio tephrosioides* Turcz.,
231. *Eucalyptus globulus* Labill., *Matricaria recutita* L., *Matricaria frigidum* (H.B.K.) Kunth, *Ambrosia peruviana* Willd., *Piper aduncum* L., *Juglans neotropica* Diels, *Cordia alliodora* (Ruiz & Pav.) Oken, *Acalypha mandonii* Müll. Arg., *Baccharis latifolia* (Ruiz & Pav.) Pers.
232. *Eucalyptus globulus* Labill., *Sonchus oleraceus* L., *Borago officinalis* L., *Oritrophium peruvianum* (Lam.) Cuatrec., *Senecio canescens* (Bonpl.) Cuatrec., *Matricaria recutita* L., *Rosmarinus officinalis* L., *Salvia rosmarinifolia* G. Don., *Lavandula angustifolia* Mill., *Urtica magellanica* Juss. ex Poir.
233. *Heisteria acuminata* (Humb. & Bonpl.) Engl., *Eucalyptus globulus* Labill., *Schinus molle* L., *Capparis crotonoides* Kunth
234. *Fuchsia ayavacensis* Kunth, *Sambucus peruviana* Kunth, *Juglans neotropica* Diels, *Lepechinia meyenii* (Walp.) Epling, *Salvia officinalis* L., *Salvia cuspidata* Ruiz & Pav., *Salvia sagittata* Ruiz. & Pav., *Siparuna muricata* (Ruiz & Pav.) A. DC.
235. *Piper aduncum* L., *Lepechinia meyenii* (Walp.) Epling, *Perezia multiflora* (Bonpl.) Less., *Oritrophium peruvianum* (Lam.) Cuatrec., *Senecio canescens* (Bonpl.) Cuatrec., *Borago officinalis* L., *Eupatorium gayanum* Wedd.
236. *Salix chilensis* Molina, *Prunus serotina* subsp.*capuli* (Cav.) McVaugh
237. *Tilia platyphyllos* Scop., *Sambucus peruviana* Kunth, *Matricaria recutita* L., *Matricaria frigidum* (H.B.K.) Kunth, *Foeniculum vulgare* Mill., *Ambrosia peruviana* Willd., *Melissa officinalis* L., *Sanguisorba minor* Scop., *Dianthus caryophyllus* L., *Tagetes erecta* L.
238. *Lantana scabiosiflora* Kunth, *Schkuhria pinnata* (Lam.) Kuntze ex Thell., *Polygala paniculata* L., *Adiantum concinnum* Humb. & Bonpl. Ex Willd., *Bejaria aestuans* Mutis ex L., *Satureja pulchella* (Kunth) Briq., *Lepechinia meyenii* (Walp.) Epling
239. *Verbena litoralis* Kunth, *Sonchus oleraceus* L., *Alternanthera halimifolia* (Lam.) Standl. Ex Pittier, *Alternanthera porrigens* (Jacq.) Kuntze, *Alternanthera brasiliana* (L.) Kuntze, *Jamesonia rotundifolia* Fée, *Portulaca oleracea* subsp. *tuberculata* Danin & H.G. Baker, *Portulaca villosa* Cham.
240. *Zingiber officinale* Roscoe, *Piper aduncum* L., *Juglans neotropica* Diels, *Malesherbia ardens* J.F. Macbr, *Alternanthera brasiliana* (L.) Kuntze, *Jamesonia rotundifolia* Fée, *Stachys lanata* Jacq.

Colic

241. *Apium graveolens* L., *Matricaria recutita* L., *Matricaria frigidum* (H.B.K.) Kunth, *Ambrosia peruviana* Willd., *Origanum majorana* L., *Adiantum concinnum* Humb. & Bonpl. Ex Willd.
242. *Foeniculum vulgare* Mill., *Matricaria recutita* L., *Matricaria frigidum* (H.B.K.) Kunth, *Ambrosia peruviana* Willd., *Mentha x piperita* L., *Scutellaria scutellarioides* (Kunth) Harley, *Lippia integrifolia* (Griseb.) Hieron., *Melissa officinalis* L., *Sanguisorba minor* Scop., *Dianthus caryophyllus* L., *Borago officinalis* L.
243. *Pimpinella anisum* L., *Mentha spicata* L.
244. *Mentha spicata* L., *Sanguisorba minor* Scop., *Pimpinella anisum* L., *Tagetes filifolia* Lag., *Illicium verum* Hook.f.,

245. *Origanum majorana* L., *Alternanthera halimifolia* (Lam.) Standl. Ex Pittier, *Peperomia inaequalifolia* Ruiz & Pav., *Melissa officinalis* L., *Matricaria recutita* L., *Matricaria frigidum* (H.B.K.) Kunth, *Ambrosia peruviana* Willd., *Foeniculum vulgare* Mill., *Hyptis sidifolia* (L'Hér.) Briq., *Mentha x piperita* L.
246. *Rosmarinus officinalis* L., *Eucalyptus globulus* Labill., *Equisetum bogotense* Kunth, *Equisetum giganteum* L., *Ruta graveolens* L.
247. *Cuphea strigulosa* Kunth, *Peperomia inaequalifolia* Ruiz & Pav., *Dianthus caryophyllus* L., *Lonicera japonica* Thunb. Ex Murray, *Urtica magellanica* Juss. ex Poir., *Urtica urens* L., *Alternanthera brasiliana* (L.) Kuntze, *Jamesonia rotundifolia* Fée, *Alternanthera halimifolia* (Lam.) Standl. Ex Pittier, *Alternanthera porrigens* (Jacq.) Kuntze, *Pilea microphylla* (L.) Liebm., *Iresine herbstii* Hook., *Erodium cicutarium* (L.) L'Hér. Ex Aiton, *Desmodium molliculum* (Kunth) DC., *Equisetum bogotense* Kunth, *Equisetum giganteum* L., *Verbena litoralis* Kunth, *Sanguisorba minor* Scop., *Buddleja utilis* Kraenzl., *Cynodon dactylon* (L.) Pers., *Bejaria aestuans* Mutis ex L., *Cenchrus echinatus* L., *Tribulus terrestris* L., *Baccharis genistelloides* (Lam.) Pers., *Bidens pilosa* L.
248. *Siparuna muricata* (Ruiz & Pav.) A. DC., *Monactis flaverioides* Kunth *, Aiouea dubia* (Kunth) Mez., *Nectandra reticulata* (Ruiz & Pav.) Mez, *Rosmarinus officinalis* L., *Salvia rosmarinifolia* G. Don., *Ruta graveolens* L., *Achyrocline alata* (Kunth) DC, *Escallonia pendula* (Ruiz & Pav.) Pers., *Bursera graveolens* (Kunth) Triana & Planch.
249. *Myristica fragrans* Houtt., *Brosimum rubescens* Taub., *Celtis loxensis* C.C. Berg, *Laccopetalum giganteum* (Wedd.) Ulbr., *Corynaea crassa* Hook.f., *Eustephia coccinea* Cav.
250. *Piper aduncum* L., *Lepechinia meyenii* (Walp.) Epling, *Eucalyptus globulus* Labill., *Nerium oleander* L., *Myrica pubescens* Humb. & Bonpl. ex Willd., *Verbena litoralis* Kunth, *Ambrosia peruviana* Willd.
251. *Ruta graveolens* L., *Gentianella dianthoides* (Kunth) Fabris ex J.S. Pringle, *Gentianella bicolor* (Wedd.) J.S. Pringle, *Lepechinia meyenii* (Walp.) Epling, *Salvia officinalis* L., *Salvia cuspidata* Ruiz & Pav., *Salvia sagittata* Ruiz. & Pav., *Origanum vulgare* L., *Schinus molle* L., *Eucalyptus globulus* Labill., *Ambrosia peruviana* Willd., *Artemisia absinthium* L., *Adiantum concinnum* Humb. & Bonpl. Ex Willd.
252. *Verbena litoralis* Kunth, *Sonchus oleraceus* L., *Alternanthera halimifolia* (Lam.) Standl. Ex Pittier, *Alternanthera porrigens* (Jacq.) Kuntze, *Alternanthera brasiliana* (L.) Kuntze, *Jamesonia rotundifolia* Fée, *Portulaca oleracea* subsp. *tuberculata* Danin & H.G. Baker, *Portulaca villosa* Cham.

Colic of the stomach

253. *Apium graveolens* L., *Matricaria recutita* L., *Matricaria frigidum* (H.B.K.) Kunth, *Ambrosia peruviana* Willd., *Origanum majorana* L., *Adiantum concinnum* Humb. & Bonpl. Ex Willd.
254. *Mentha spicata* L., *Pimpinella anisum* L., *Tagetes filifolia* Lag., *Illicium verum* Hook.f.

Concussions

255. *Sambucus peruviana* Kunth *,Ricinus communis* L.
256. *Matricaria recutita* L., *Nerium oleander* L., *Myrica pubescens* Humb. & Bonpl. ex Willd., *Plantago linearis* Kunth, *Plantago major* L.

Congestion

257. *Eucalyptus globulus* Labill., *Sonchus oleraceus* L., *Borago officinalis* L., *Oritrophium peruvianum* (Lam.) Cuatrec., *Senecio canescens* (Bonpl.) Cuatrec., *Matricaria recutita* L., *Rosmarinus officinalis* L., *Salvia rosmarinifolia* G. Don., *Lavandula angustifolia* Mill., *Urtica magellanica* Juss. ex Poir.

Contraceptive

258. *Persea americana* Mill., *Linum sativum* L., *Linum usitatissimum* L.
259. *Aa paleacea* (Kunth) Rchb. f., *Mimosa albida* Humb. & Bonpl. Ex Willd., *Sicana odorifera* (Vell.) Naudin
260. *Uncaria tomentosa* (Willd. ex Roem. & Schult.) DC., *Phyllanthus niruri* L., *Phyllanthus stipulatus* (Raf.) G.L.Webster, *Phyllanthus urinaria* L., *Linum usitatissimum* L., *Linum sativum* L. *Peumus boldus* Molina, *Cordia lutea* Lam., *Capsella bursa-pastoris* (L.) Medik.
261. *Thelypteris* cf. *scalaris* (Christ.) Alston, *Baccharis ciliaris* (Retz.) Koeler, *Baccharis indica* L., *Loricaria ferruginea* (Ruiz & Pav.) Wedd., *Desmodium molliculum* (Kunth) DC., *Petroselinum crispum* (Mill.) Fuss

Cough

262. *Cronquistianthus lavandulifolius* (DC.) R.M. King & H. Rob., *Piper aduncum* L., *Rubus robustus* C. Presl., *Juglans neotropica* Diels, *Lepechinia meyenii* (Walp.) Epling, *Salvia officinalis* L., *Salvia cuspidata* Ruiz & Pav., *Salvia sagittata* Ruiz. & Pav., *Borago officinalis* L., *Salvia discolor* Kunth, *Ambrosia peruviana* Willd., *Miconia salicifolia* (Bonpl. ex Naudin) Naudin, *Oritrophium peruvianum* (Lam.) Cuatrec., *Senecio canescens* (Bonpl.) Cuatrec.
263. *Perezia multiflora* (Bonpl.) Less., *Piper aduncum* L., *Eucalyptus globulus* Labill., *Malesherbia ardens* J.F. Macbr., *Alternanthera brasiliana* (L.) Kuntze, *Jamesonia rotundifolia* Fée, *Stachys lanata* Jacq., *Oritrophium peruvianum* (Lam.) Cuatrec., *Senecio canescens* (Bonpl.) Cuatrec., *Juglans neotropica* Diels, *Laccopetalum giganteum* (Wedd.) Ulbr., *Senecio tephrosioides* Turcz., , *Tilia platyphyllos* Scop., *Rubus robustus* C. Presl.
264. *Senecio canescens* (Bonpl.) Cuatrec., *Borago officinalis* L., *Eucalyptus globulus* Labill., *Perezia multiflora* (Bonpl.) Less., *Sonchus oleraceus* L., *Matricaria recutita* L., *Matricaria frigidum* (H.B.K.) Kunth, *Ambrosia peruviana* Willd., *Melissa officinalis* L., *Peperomia inaequalifolia* Ruiz & Pav., *Mentha x piperita* L., *Scutellaria scutellarioides* (Kunth) Harley, *Lippia integrifolia* (Griseb.) Hieron., *Dianthus caryophyllus* L., *Tagetes erecta* L., *Acanthoxanthium spinosum* (L.) Fourr., *Aphelandra cirsioides* Lindau
265. *Tagetes erecta* L., *Melissa officinalis* L., *Sanguisorba minor* Scop., *Mentha x piperita* L., *Scutellaria scutellarioides* (Kunth) Harley, *Lippia integrifolia* (Griseb.) Hieron., *Matricaria recutita* L., *Matricaria frigidum* (H.B.K.) Kunth, *Ambrosia peruviana* Willd.
266. *Borago officinalis* L., *Oritrophium peruvianum* (Lam.) Cuatrec., *Senecio canescens* (Bonpl.) Cuatrec.
267. *Sambucus peruviana* Kunth*, Ricinus communis* L.
268. *Sambucus peruviana* Kunth, *Matricaria recutita* L., *Matricaria frigidum* (H.B.K.) Kunth, *Foeniculum vulgare* Mill., *Ambrosia peruviana* Willd., *Melissa officinalis* L., *Sanguisorba minor* Scop., *Dianthus caryophyllus* L., *Tagetes erecta* L.

269. *Hedyosmum racemosum* (Ruiz & Pav.) G. Don., *Heisteria acuminata* (Humb. & Bonpl.) Engl., *Cinchona officinalis* L.
270. *Scabiosa atropurpurea* L., *Tilia platyphyllos* Scop., *Malesherbia ardens* J.F. Macbr., *Alternanthera brasiliana* (L.) Kuntze, *Jamesonia rotundifolia* Fée, *Stachys lanata* Jacq., *Cuphea strigulosa* Kunth, *Alternanthera halimifolia* (Lam.) Standl. Ex Pittier, *Alternanthera porrigens* (Jacq.) Kuntze, *Epilobium* sp.
271. *Salvia officinalis* L., *Piper aduncum* L., *Juglans neotropica* Diels, *Eucalyptus globulus* Labill.
272. *Allium sativum* L., *Piper aduncum* L., *Sonchus oleraceus* L., *Eucalyptus globulus* Labill., *Oritrophium peruvianum* (Lam.) Cuatrec., *Senecio canescens* (Bonpl.) Cuatrec.
273. *Malesherbia ardens* J.F. Macbr., *Tilia platyphyllos* Scop., *Leucaena leucocephala* (Lam.) de Wit, *Oritrophium peruvianum* (Lam.) Cuatrec., *Laccopetalum giganteum* (Wedd.) Ulbr., *Senecio tephrosioides* Turcz.,
274. *Malva parviflora* L., *Desmodium molliculum* (Kunth) DC., *Mauria heterophylla* Kunth, *Verbena litoralis* Kunth, *Equisetum bogotense* Kunth, *Equisetum giganteum* L., *Bidens pilosa* L., *Malva sylvestris* L., *Alcea rosea* L., *Pelargonium odoratissimum* (L.) L'Hér.
275. *Myristica fragrans* Houtt., *Brosimum rubescens* Taub., *Celtis loxensis* C.C. Berg, *Laccopetalum giganteum* (Wedd.) Ulbr., *Corynaea crassa* Hook.f., *Eustephia coccinea* Cav.
276. *Eucalyptus globulus* Labill., *Matricaria recutita* L., *Matricaria frigidum* (H.B.K.) Kunth., *Ambrosia peruviana* Willd., *Piper aduncum* L., *Juglans neotropica* Diels, *Cordia alliodora* (Ruiz & Pav.) Oken, *Acalypha mandonii* Müll. Arg., *Baccharis latifolia* (Ruiz & Pav.) Pers.
277. *Piper aduncum* L., *Lepechinia meyenii* (Walp.) Epling, *Perezia multiflora* (Bonpl.) Less., *Oritrophium peruvianum* (Lam.) Cuatrec., *Senecio tephrosioides* Turcz., , *Borago officinalis* L., *Eupatorium gayanum* Wedd.
278. *Plantago linearis* Kunth, *Piper aduncum* L., *Juglans neotropica* Diels, *Oritrophium peruvianum* (Lam.) Cuatrec., *Senecio tephrosioides* Turcz., , *Eucalyptus globulus* Labill.
279. *Rubus robustus* C. Presl., *Salvia discolor* Kunth, *Ambrosia peruviana* Willd., *Miconia salicifolia* (Bonpl. ex Naudin) Naudin
280. *Cinchona officinalis* L., *Buddleja utilis* Kraenzl., *Cynodon dactylon* (L.) Pers.
281. *Tilia platyphyllos* Scop., *Sambucus peruviana* Kunth, *Matricaria recutita* L., *Matricaria frigidum* (H.B.K.) Kunth, *Foeniculum vulgare* Mill., *Ambrosia peruviana* Willd., *Melissa officinalis* L., *Sanguisorba minor* Scop., *Dianthus caryophyllus* L., *Tagetes erecta* L.
282. *Clerodendrum* sp., *Oritrophium peruvianum* (Lam.) Cuatrec., *Laccopetalum giganteum* (Wedd.) Ulbr., *Senecio tephrosioides* Turcz., , *Malersherbia ardens* Macbr., *Alternanthera brasiliana* (L.) Kuntze, *Jamesonia rotundifolia* Fée, *Stachys lanata* Jacq.
283. *Zingiber officinale* Roscoe, *Piper aduncum* L., *Juglans neotropica* Diels, *Malersherbia ardens* Macbr., *Alternanthera brasiliana* (L.) Kuntze, *Jamesonia rotundifolia* Fée, *Stachys lanata* Jacq.

Cysts

284. *Eustephia coccinea* Cav., *Buddleja utilis* Kraenzl. *Bejaria aestuans* Mutis ex L., *Pelargonium odoratissimum* (L.) L'Hér.
285. *Dioscorea trifida* L.f., *Buddleja utilis* Kraenzl., *Bejaria aestuans* Mutis ex L., *Paranephelius uniflorus* Poepp., *Rorippa nasturtium-aquaticum* (L.) Hayek
286. *Bejaria aestuans* Mutis ex L., *Buddleja utilis* Kraenzl., *Dioscorea trifida* L.f., *Tiquilia paronychioides* (Phil.) A.T. Richardson, *Ilex guayusa* Loes., *Geranium ayavacense* Willd. ex Kunth, *Geranium sessiliflorum* Cav., *Monactis flaverioides* Kunth, *Malva sylvestris* L., *Alcea rosea* L., *Pelargonium odoratissimum* (L.) L'Hér., *Malva parviflora* L., *Bidens pilosa* L., *Verbena litoralis* Kunth, *Plantago linearis* Kunth, *Plantago major* L., *Equisetum bogotense* Kunth, *Equisetum giganteum* L., *Muehlenbeckia tamnifolia (Kunth) Meisn.*, *Smilax kunthii* Killip & C.V. Morton, *Oreocallis grandiflora* (Lam.) R.Br., *Cinchona officinalis* L.
287. *Cynodon dactylon* (L.) Pers., *Equisetum bogotense* Kunth, *Equisetum giganteum* L., *Verbena litoralis* Kunth, *Bidens pilosa* L., *Monactis flaverioides* Kunth, *Malva sylvestris* L., *Alcea rosea* L., *Pelargonium odoratissimum* (L.) L'Hér., *Malva parviflora* L., *Buddleja utilis* Kraenzl., *Bejaria aestuans* Mutis ex L., *Smilix medica* M.Martens & Galeotti, *Cuphea strigulosa* Kunth
288. *Pilea microphylla* (L.) Liebm., *Equisetum bogotense* Kunth, *Equisetum giganteum* L., *Bixa orellana* L., *Phyllanthus niruri* L., *Phyllanthus stipulatus* (Raf.) G.L.Webster, *Phyllanthus urinaria* L.

Daño

289. *Mauria heterophylla* Kunth, *Phytolacca bogotensis* Kunth, *Juglans neotropica* Diels, *Artemisia absinthium* L., *Equisetum giganteum* L.
290. *Mandevilla* cf. *trianae* Woodson, *Daucus montanus* Humb. & Bonpl. ex Spreng., *Baccharis latifolia* (Ruiz & Pav.) Pers., *Acalypha mandonii* Müll. Arg., *Trixis cacalioides* (Kunth) D. Don, *Achyrocline alata* (Kunth) DC, *Centropogon articulatus* Drake, *Fuchsia ayavacensis* Kunth, *Siphocampylus cutervensis* Zahlbr., *Monactis flaverioides* Kunth
291. *Diplostephium sagasteguii* Cuatrec., *Alternanthera brasiliana* (L.) Kuntze, *Epidendrum calanthum* Rchb.f. & Warsz., *Solanum mammosum* L., *Brugmansia candida* Pers.
292. *Ferreyranthus verbascifolius* (Kunth) H. Rob. & Brettell, *Cantua quercifolia* Juss., *Trixis cacalioides* (Kunth) D. Don, *Ruta graveolens* L. *Vallea stipularis* L.f., *Centropogon articulatus* Drake, *Fuchsia ayavacensis* Kunth, *Siphocampylus cutervensis* Zahlbr., *Salvia ayavacensis* Kunth, *Matricaria frigidum* (H.B.K.) Kunth, *Matricaria recutita* L., *Ambrosia peruviana* Willd.
293. *Mikania leiostachya* Benth., *Oreocallis grandiflora* (Lam.) R.Br., *Acalypha mandonii* Müll. Arg., *Baccharis latifolia* (Ruiz & Pav.) Pers., *Monactis flaverioides* Kunth
294. *Cordia alliodora* (Ruiz & Pav.) Oken, *Porophyllum ruderale* (Jacq.) Cass., *Rosmarinus officinalis* L., *Eucalyptus globulus* Labill., *Brugmansia arborea* (L.) Lagerh., *Brugmansia sanguinea* (Ruiz & Pav.) D. Don, *Spartium junceum* L., *Trixis cacalioides* (Kunth) D. Don
295. *Trixis cacalioides* (Kunth) D. Don, *Siparuna muricata* (Ruiz & Pav.) A. DC., *Ruta graveolens* L., *Artemisia absinthium* L. , *Achyrocline alata* (Kunth) DC, *Escallonia pendula* (Ruiz & Pav.) Pers.
296. *Salvia discolor* Kunth, *Ambrosia peruviana* Willd., *Miconia salicifolia* (Bonpl. ex Naudin) Naudin, *Cordia alliodora* (Ruiz & Pav.) Oken, *Siparuna muricata* (Ruiz & Pav.) A. DC., *Porophyllum ruderale* (Jacq.) Cass., *Trixis cacalioides* (Kunth) D. Don, *Ruta graveolens* L.
297. *Bursera graveolens* (Kunth) Triana & Planch., *Salvia rosmarinifolia* G. Don., *Rosmarinus officinalis* L.
298. *Siphocampylus cutervensis* Zahlbr., *Fuchsia ayavacensis* Kunth, *Huperzia hohenackeri* (Herter) Holub, *Vallea stipularis* L.f.

299. *Juglans neotropica* Diels, *Siparuna muricata* (Ruiz & Pav.) A. DC., *Oreocallis grandiflora* (Lam.) R.Br., *Achyrocline alata* (Kunth) DC, *Sambucus peruviana* Kunth
300. *Ocimum basilicum* L., *Eucalyptus globulus* Labill., *Schinus molle* L., *Spartium junceum* L., *Porophyllum ruderale* (Jacq.) Cass., *Ruta graveolens* L., *Ambrosia peruviana* Willd.
301. *Ocimum basilicum* L., *Tilia platyphyllos* Scop., *Melissa officinalis* L., *Origanum majorana* L., *Cymbopogon citratus* (DC.) Stapf, *Aloysia triphylla* Royle
302. *Salvia ayavacensis* Kunth, *Senecio genisianus* Cuatrec., *Phytolacca bogotensis* Kunth, *Siphocampylus cutervensis* Zahlbr., *Daucus montanus* Humb. & Bonpl. ex Spreng., *Salvia rosmarinifolia* G. Don.
303. *Siparuna muricata* (Ruiz & Pav.) A. DC., *Monactis flaverioides* Kunth, *Achyrocline alata* (Kunth) DC, *Aiouea dubia* (Kunth) Mez., *Nectandra reticulata* (Ruiz & Pav.) Mez., *Rosmarinus officinalis* L., *Salvia rosmarinifolia* G. Don., *Ruta graveolens* L., *Escallonia pendula* (Ruiz & Pav.) Pers., *Bursera graveolens* (Kunth) Triana & Planch.
304. *Fuchsia ayavacensis* Kunth, *Sambucus peruviana* Kunth, ,*Juglans neotropica* Diels, *Lepechinia meyenii* (Walp.) Epling, *Salvia officinalis* L., *Salvia cuspidata* Ruiz & Pav., *Salvia sagittata* Ruiz. & Pav., *Siparuna muricata* (Ruiz & Pav.) A. DC.
305. *Phytolacca bogotensis* Kunth, *Siparuna muricata* (Ruiz & Pav.) A. DC., *Artemisia absinthium* L., *Sambucus peruviana* Kunth, *Mauria heterophylla* Kunth, *Equisetum giganteum* L.
306. *Oreocallis grandiflora* (Lam.) R.Br., *Vallea stipularis* L.f., *Daucus montanus* Humb. & Bonpl. ex Spreng., *Siphocampylus cutervensis* Zahlbr., *Bejaria aestuans* Mutis ex L., *Trifolium repens* L., *Oxalis bulbigera* R. Knuth.
307. *Brugmansia sanguinea* (Ruiz & Pav.) D. Don., *Brugmansia candida* Pers., *Brugmansia arborea* (L.) Lagerh., *Porophyllum ruderale* (Jacq.) Cass., *Zea mays* L., *Gaultheria reticulata* Kunth
308. *Jaltomata* sp., *Diplostephium sagasteguii* Cuatrec., *Alternanthera brasiliana* (L.) Kuntze, *Epidendrum calanthum* Rchb.f. & Warsz., *Solanum mammosum* L., *Brugmansia sanguinea* (Ruiz & Pav.) D. Don., *Brugmansia candida* Pers., *Brugmansia arborea* (L.) Lagerh.

Dengue

309. *Phytolacca bogotensis* Kunth, *Siparuna muricata* (Ruiz & Pav.) A. DC., *Artemisia absinthium* L., *Sambucus peruviana* Kunth, *Mauria heterophylla* Kunth, *Equisetum giganteum* L.

Depression

310. *Borago officinalis* L., *Oritrophium peruvianum* (Lam.) Cuatrec., *Senecio canescens* (Bonpl.) Cuatrec.
311. *Tillandsia multiflora* var. *decipiens* (André) L.B. Sm., *Melissa officinalis* L., *Dianthus caryophyllus* L., *Citrus limetta* Risso
312. *Origanum majorana* L., *Alternanthera halimifolia* (Lam.) Standl. Ex Pittier, *Peperomia inaequalifolia* Ruiz & Pav., *Melissa officinalis* L., *Matricaria recutita* L., *Matricaria frigidum* (H.B.K.) Kunth, *Ambrosia peruviana* Willd., *Foeniculum vulgare* Mill., *Hyptis sidifolia* (L'Hér.) Briq., *Mentha x piperita* L.,
313. *Peperomia galioides* Kunth, *Pelargonium odoratissimum* (L.) L'Hér., *Tillandsia cacticola* L.B. Sm., *Tillandsia multiflora* var. *decipiens* (André) L.B. Sm., *Echeveria peruviana* Meyen, *Pilea microphylla* (L.) Liebm.
314. *Cydonia oblonga* Mill., *Matricaria recutita* L., *Matricaria frigidum* (H.B.K.) Kunth, *Ambrosia peruviana* Willd. *Melissa officinalis* L., *Foeniculum vulgare* Mill., *Lonicera japonica* Thunb. Ex Murray, *Rosmarinus officinalis* L., *Salvia rosmarinifolia* G. Don.
315. *Cydonia oblonga* Mill., *Matricaria recutita* L., *Matricaria frigidum* (H.B.K.) Kunth, *Ambrosia peruviana* Willd., *Melissa officinalis* L., *Sanguisorba minor* Scop., *Borago officinalis* L., *Viola tricolor* L., *Lathyrus odoratus* L., *Origanum majorana* L., *Rosmarinus officinalis* L., *Salvia rosmarinifolia* G. Don.
316. *Sanguisorba minor* Scop., *Bejaria aestuans* Mutis ex L., *Cenchrus echinatus* L., *Tribulus terrestris* L., *Baccharis genistelloides* (Lam.) Pers., *Bidens pilosa* L., *Alternanthera porrigens* (Jacq.) Kuntze, *Cuphea strigulosa* Kunth, *Melissa officinalis* L., *Peperomia inaequalifolia* Ruiz & Pav., *Dianthus caryophyllus* L., *Tagetes erecta* L., *Matricaria recutita* L., *Matricaria frigidum* (H.B.K.) Kunth, *Ambrosia peruviana* Willd., *Cestrum nocturnum* L.
317. *Citrus sinensis* (L.) Osbeck, *Melissa officinalis* L., *Dianthus caryophyllus* L., *Tagetes erecta* L., *Matricaria recutita* L., *Matricaria frigidum* (H.B.K.) Kunth, *Ambrosia peruviana* Willd., *Origanum majorana* L., *Desmodium molliculum* (Kunth) DC., *Minthostachys mollis* (Kunth) Griseb. , *Rosmarinus officinalis* L., *Salvia rosmarinifolia* G. Don.
318. *Ruta graveolens* L., *Gentianella dianthoides* (Kunth) Fabris ex J.S. Pringle, *Gentianella bicolor* (Wedd.) J.S. Pringle, *Lepechinia meyenii* (Walp.) Epling, *Salvia officinalis* L., *Salvia cuspidata* Ruiz & Pav., *Origanum vulgare* L., *Schinus molle* L., *Eucalyptus globulus* Labill., *Ambrosia peruviana* Willd., *Artemisia absinthium* L., *Adiantum concinnum* Humb. & Bonpl. Ex Willd.
319. *Aloysia triphylla* Royle, *Marrubium vulgare* L., *Desmodium molliculum* (Kunth) DC., *Minthostachys mollis* (Kunth) Griseb. , *Melissa officinalis* L., *Foeniculum vulgare* Mill., *Lonicera japonica* Thunb. Ex Murray, *Dianthus* caryophyllus L., *Tagetes erecta* L., *Viola tricolor* L., *Lathyrus odoratus* L.

Detoxification

320. *Epilobium* sp., *Sanguisorba minor* Scop., *Bejaria aestuans* Mutis ex L., *Cenchrus echinatus* L., *Tribulus terrestris* L., *Baccharis genistelloides* (Lam.) Pers., *Bidens pilosa* L., *Iresine herbstii* Hook., *Alternanthera porrigens* (Jacq.) Kuntze, *Cuphea strigulosa* Kunth

Detoxification of alcohol drugs

321. *Senna bicapsularis* (L.) Roxb., *quisetum bogotense* Kunth, *Equisetum giganteum* L., *Acanthoxanthium spinosum* (L.) Fourr., *Verbena litoralis* Kunth

Diabetes

322. *Baccharis genistelloides* (Lam.) Pers., *Schkuhria pinnata* (Lam.) Kuntze ex Thell., *Polygala paniculata* L., *Verbena litoralis* Kunth, *Bidens pilosa* L., *Equisetum bogotense* Kunth, *Equisetum giganteum* L., *Cuphea strigulosa* Kunth, *Ipomoea batatas* (L.) Lam.
323. *Bidens pilosa* L., *Mauria heterophylla* Kunth, *Eugenia obtusifolia* Cambess., *Tiquilia paronychioides* (Phil.) A.T. Richardson, *Zea mays* L., *Equisetum bogotense* Kunth, *Equisetum giganteum* L., *Psidium guajava* L., *Sanguisorba minor* Scop., *Cestrum nocturnum* L.

324. *Schkuhria pinnata* (Lam.) Kuntze ex Thell., *Urtica magellanica* Juss. ex Poir., *Urtica urens* L., *Alternanthera porrigens* (Jacq.) Kuntze, *Cuphea strigulosa* Kunth, *Adiantum concinnum* Humb. & Bonpl. Ex Willd., *Satureja pulchella* (Kunth) Briq., *Bejaria aestuans* Mutis ex L., *Peumus boldus* Molina, *Rorippa nasturtium-aquaticum* (L.) Hayek, *Buddleja utilis* Kraenzl., *Polygala paniculata* L.
325. *Bejaria aestuans* Mutis ex L., *Buddleja utilis* Kraenzl., *Dioscorea trifida* L.f., *Tiquilia paronychioides* (Phil.) A.T. Richardson, *Ilex guayusa* Loes., *Geranium ayavacense* Willd. ex Kunth, *Geranium sessiliflorum* Cav., *Monactis flaverioides* Kunth, *Malva sylvestris* L., *Alcea rosea* L., *Pelargonium odoratissimum* (L.) L'Hér., *Malva parviflora* L., *Bidens pilosa* L., *Verbena litoralis* Kunth, *Plantago linearis* Kunth, *Plantago major* L., *Equisetum bogotense* Kunth, *Equisetum giganteum* L., *Muehlenbeckia tamnifolia (Kunth) Meisn.*, *Smilax kunthii* Killip & C.V. Morton, *Oreocallis grandiflora* (Lam.) R.Br., *Cinchona officinalis* L.
326. *Spartium junceum* L., *Cordia lutea* Lam.
327. *Gentianella crassicaulis* J.S. Pringle, *Geranium ayavacense* Willd. ex Kunth, *Geranium sessiliflorum* Cav., *Taraxacum officinale* F.H.Wigg., *Gentianella dianthoides* (Kunth) Fabris ex J.S. Pringle, *Gentianella bicolor* (Wedd.) J.S. Pringle.
328. *Geranium ayavacense* Willd. ex Kunth, *Geranium sessiliflorum* Cav., *Juglans neotropica* Diels, *Otholobium glandulosum* (L.) J.W. Grimes, *Ilex guayusa* Loes., *Eucalyptus citriodora* Hook.
329. *Otholobium glandulosum* (L.) J.W. Grimes, *Matricaria recutita* L., *Matricaria frigidum* (H.B.K.) Kunth, A*mbrosia peruviana* Willd., *Mentha spicata* L., *Sanguisorba minor* Scop., *Pimpinella anisum* L., *Tagetes filifolia* Lag.
330. *Brosimum rubescens* Taub., *Celtis loxensis* C.C. Berg, *Scabiosa atropurpurea* L.
331. *Rubus robustus* C. Presl., *Salvia discolor* Kunth, *Ambrosia peruviana* Willd., *Miconia salicifolia* (Bonpl. ex Naudin) Naudin
332. *Matricaria recutita* L., *Matricaria frigidum* (H.B.K.) Kunth, *Ambrosia peruviana* Willd., *Melissa officinalis* L., *Sanguisorba minor* Scop., *Foeniculum vulgare* Mill.

Diarrhea

333. *Cocos nucifera* L., *Cymbopogon citratus* (DC.) Stapf, *Otholobium glandulosum* (L.) J.W. Grimes, *Foeniculum vulgare* Mill., *Mentha x piperita* L.
334. *Hypericum silenoides* Juss., *Otholobium glandulosum* (L.) J.W. Grimes, *Cuphea strigulosa* Kunth
335. *Desmodium molliculum* (Kunth) DC., *Mauria heterophylla* Kunth, *Bidens pilosa* L., *Verbena litoralis* Kunth
336. *Otholobium glandulosum* (L.) J.W. Grimes, *Matricaria recutita* L., *Matricaria frigidum* (H.B.K.) Kunth, A*mbrosia peruviana* Willd., *Mentha spicata* L., *Sanguisorba minor* Scop., *Pimpinella anisum* L., *Tagetes filifolia* Lag.
337. *Persea americana* Mill., *Linum sativum* L., *Linum usitatissimum* L.
338. *Cuphea strigulosa* Kunth, *Peperomia inaequalifolia* Ruiz & Pav., *Dianthus caryophyllus* L., *Lonicera japonica* Thunb. Ex Murray, *Alternanthera halimifolia* (Lam.) Standl. Ex Pittier, *Alternanthera porrigens* (Jacq.) Kuntze, *Alternanthera brasiliana* (L.) Kuntze, *Jamesonia rotundifolia* Fée, *Pilea microphylla* (L.) Liebm., *Iresine herbstii* Hook., *Erodium cicutarium* (L.) L'Hér. Ex Aiton, *Desmodium molliculum* (Kunth) DC., *Equisetum bogotense* Kunth, *Equisetum giganteum* L., *Verbena litoralis* Kunth, *Sanguisorba minor* Scop., *Buddleja utilis* Kraenzl., *Cynodon dactylon* (L.) Pers., *Bejaria aestuans* Mutis ex L., *Cenchrus echinatus* L., *Tribulus terrestris* L., *Baccharis genistelloides* (Lam.) Pers., *Bidens pilosa* L.
339. *Passiflora ligularis* Juss., *Otholobium glandulosum* (L.) J.W. Grimes, *Foeniculum vulgare* Mill., *Marrubium vulgare* L., *Desmodium molliculum* (Kunth) DC., *Minthostachys mollis* (Kunth) Griseb. .
340. *Punica granatum* L., *Foeniculum vulgare* Mill., *Persea americana* Mill.
341. *Brugmansia candida* Pers., *Gaultheria reticulata* Kunth

Dizziness

342. *Gentianella crassicaulis* J.S. Pringle, *Geranium ayavacense* Willd. ex Kunth, *Geranium sessiliflorum* Cav., *Taraxacum officinale* F.H.Wigg., *Gentianella dianthoides* (Kunth) Fabris ex J.S. Pringle, *Gentianella bicolor* (Wedd.) J.S. Pringle

Domination

343. *Cheilanthes myriophylla* Desv., *Hesperoxiphion niveum* (Ravenna) Ravenna, *Clethra castaneifolia* Meisn.

Dysentery

344. *Hypericum silenoides* Juss., *Otholobium glandulosum* (L.) J.W. Grimes, *Cuphea strigulosa* Kunth

Epilepsy

345. *Myroxylon balsamum* (L.) Harms, *Couepia* sp., *Trichilia* sp., *Strychnos* sp., *Achyrocline alata* (Kunth) DC, *Aiouea dubia* (Kunth) Mez., *Nectandra reticulata* (Ruiz & Pav.) Mez., *Myristica fragrans* Houtt., *Thevetia peruviana* (Pers.) K. Schum.
346. *Aiouea dubia* (Kunth) Mez., *Couepia* sp., *Trichilia* sp., *Strychnos* sp., *Myroxylon balsamum* (L.) Harms, *Myristica fragrans* Houtt., *Thevetia peruviana* (Pers.) K. Schum.
347. *Nectandra floribunda* (Sw.) Nees, *Couepia* sp., *Trichilia* sp., *Strychnos* sp., *Myroxylon balsamum* (L.) Harms, *Myristica fragrans* Houtt., *Thevetia peruviana* (Pers.) K. Schum.
348. *Malva sylvestris* L., *Melissa officinalis* L., *Sanguisorba minor* Scop., *Origanum majorana* L., *Viola tricolor* L., *Cymbopogon citratus* (DC.) Stapf, *Aloysia triphylla* Royle
349. *Myristica fragrans* Houtt., *Couepia* sp., *Trichilia* sp., *Strychnos* sp., *Myroxylon balsamum* (L.) Harms, *Achyrocline alata* (Kunth) DC, *Aiouea dubia* (Kunth) Mez., *Nectandra reticulata* (Ruiz & Pav.) Mez, *Thevetia peruviana* (Pers.) K. Schum.
350. *Peperomia inaequalifolia* Ruiz & Pav., *Melissa officinalis* L., *Sanguisorba minor* Scop., *Origanum majorana* L., *Viola tricolor* L., *Lathyrus odoratus* L.
351. *Laccopetalum giganteum* (Wedd.) Ulbr., *Peperomia inaequalifolia* Ruiz & Pav.
352. *Viola tricolor* L., *Melissa officinalis* L.

Fertility

353. *Ipomoea pauciflora* M. Martens & Galeotti, *Corynaea crassa* Hook.f., *Laccopetalum giganteum* (Wedd.) Ulbr., *Brosimum rubescens* Taub., *Celtis loxensis* C.C. Berg, *Heisteria acuminata* (Humb. & Bonpl.) Engl., *Cinchona officinalis* L., *Eustephia coccinea* Cav.
354. *Myristica fragrans* Houtt., *Brosimum rubescens* Taub., *Celtis loxensis* C.C. Berg, *Laccopetalum giganteum* (Wedd.) Ulbr., *Corynaea crassa* Hook.f., *Eustephia coccinea* Cav.
355. *Laccopetalum giganteum* (Wedd.) Ulbr., *Allium sativum* L., *Cordia alliodora* (Ruiz & Pav.) Oken, *Daphnopsis weberbaueri* Domke, *Corynaea crassa* Hook.f., *Brosimum rubescens* Taub.
356. *Cinchona officinalis* L., *Brosimum rubescens* Taub., *Celtis loxensis* C.C. Berg, *Inga edulis* C.Martius, *Inga feuillei* DC., *Isoetes andina* Spruce ex Hook., *Peperomia quadrifolia* (L.) Kunth, *Corynaea crassa* Hook.f.
357. *Daphnopsis weberbaueri* Domke, *Laccopetalum giganteum* (Wedd.) Ulbr., *Corynaea crassa* Hook.f., *Heisteria acuminata* (Humb. & Bonpl.) Engl., *Brosimum rubescens* Taub.
358. *Celtis loxensis* C.C. Berg, *Brosimum rubescens* Taub., *Heisteria acuminata* (Humb. & Bonpl.) Engl., *Laccopetalum giganteum* (Wedd.) Ulbr., *Cinchona officinalis* L., *Corynaea crassa* Hook.f.

Fever

359. *Tessaria integrifolia* Ruiz & Pav., *Equisetum bogotense* Kunth, *Equisetum giganteum* L., *Verbena litoralis* Kunth, *Mauria heterophylla* Kunth, *Iresine diffusa* Humb. & Bonpl. ex Willd., *Zea mays* L.
360. *Sambucus peruviana* Kunth, *Ricinus communis* L.
361. *Sambucus peruviana* Kunth *, Matricaria recutita* L., *Matricaria frigidum* (H.B.K.) Kunth, *Foeniculum vulgare* Mill., *Ambrosia peruviana* Willd., *Melissa officinalis* L., *Sanguisorba minor* Scop., *Dianthus caryophyllus* L., *Tagetes erecta* L.
362. *Scirpus californicus* subsp. *tatora* (Kunth) T. Koyama, *Salix chilensis* Molina
363. *Cuphea strigulosa* Kunth, *Peperomia inaequalifolia* Ruiz & Pav., *Dianthus caryophyllus* L., *Tagetes erecta* L., *Lonicera japonica* Thunb. Ex Murray, *Urtica magellanica* Juss. ex Poir., *Urtica urens* L., *Alternanthera halimifolia* (Lam.) Standl. Ex Pittier, *Alternanthera porrigens* (Jacq.) Kuntze, *Alternanthera brasiliana* (L.) Kuntze, *Jamesonia rotundifolia* Fée, *Pilea microphylla* (L.) Liebm., *Iresine herbstii* Hook., *Erodium cicutarium* (L.) L'Hér. Ex Aiton, *Desmodium molliculum* (Kunth) DC., *Equisetum bogotense* Kunth, *Equisetum giganteum* L., *Verbena litoralis* Kunth, *Sanguisorba minor* Scop., *Buddleja utilis* Kraenzl., *Cynodon dactylon* (L.) Pers., *Bejaria aestuans* Mutis ex L., *Cenchrus echinatus* L., *Tribulus terrestris* L., *Baccharis genistelloides* (Lam.) Pers., *Bidens pilosa* L.
364. *Muehlenbeckia tamnifolia (Kunth) Meisn.*, *Equisetum giganteum* L.
365. *Salix chilensis* Molina, *Prunus serotina* subsp. *capuli* (Cav.) McVaugh
366. *Tilia platyphyllos* Scop., *Sambucus peruviana* Kunth *, Matricaria recutita* L., *Matricaria frigidum* (H.B.K.) Kunth, *Foeniculum vulgare* Mill., Coleo, *Ambrosia peruviana* Willd., *Melissa officinalis* L., *Sanguisorba minor* Scop., *Dianthus caryophyllus* L., *Tagetes erecta* L.
367. *Urtica magellanica* Juss. ex Poir., *Oritrophium peruvianum* (Lam.) Cuatrec., *Laccopetalum giganteum* (Wedd.) Ulbr., *Senecio tephrosioides* Turcz., *Verbena litoralis* Kunth, *Gentianella bicolor* (Wedd.) J.S. Pringle, *Sonchus oleraceus* L., *Juglans neotropica* Diels, *Schinus molle* L., *Ruta graveolens* L., *Piper aduncum* L.
368. *Verbena litoralis* Kunth, *Piper aduncum* L., *Monactis flaverioides* Kunth, *Malva sylvestris* L., *Pelargonium odoratissimum* (L.) L'Hér., *Malva parviflora* L., *Plantago linearis* Kunth, *Plantago major* L., *Eustephia coccinea* Cav.

Fibroids

369. *Cynodon dactylon* (L.) Pers., *Equisetum bogotense* Kunth, *Equisetum giganteum* L., *Verbena litoralis* Kunth, *Bidens pilosa* L., *Monactis flaverioides* Kunth, *Malva sylvestris* L., *Pelargonium odoratissimum* (L.) L'Hér., *Malva parviflora* L., *Buddleja utilis* Kraenzl., *Bejaria aestuans* Mutis ex L., *Smilax medica* Schltdl. & Cham., *Cuphea strigulosa* Kunth

Food Coloring

370. *Bixa orellana* L., *Uncaria tomentosa* (Willd. ex Roem. & Schult.) DC., *Mimosa nothacacia* Barneby

Forgetting

371. *Petroselinum crispum* (Mill.) Fuss, *Melissa officinalis* L., *Sanguisorba minor* Scop., *Origanum majorana* L., *Tillandsia cacticola* L.B. Sm., *Tillandsia multiflora* var. *decipiens* (André) L.B. Sm., *Echeveria peruviana* Meyen
372. *Peperomia inaequalifolia* Ruiz & Pav., *Melissa officinalis* L., *Sanguisorba minor* Scop., *Origanum majorana* L., *Viola tricolor* L., *Lathyrus odoratus* L.
373. *Viola tricolor* L., *Melissa officinalis* L.

Fractures

374. *Alternanthera brasiliana* (L.) Kuntze, *Gaultheria erecta* Vent., *Oritrophium peruvianum* (Lam.) Cuatrec., *Laccopetalum giganteum* (Wedd.) Ulbr., *Senecio tephrosioides* Turcz., , *Clerodendrum* sp.
375. *Prunus serotina* Ehrh., *Ambrosia peruviana* Willd., *Artemisia absinthium* L., *Ruta graveolens* L., *Rosmarinus officinalis* L., *Salvia rosmarinifolia* G. Don.

Fragrance

376. *Diplostephium sagasteguii* Cuatrec., *Alternanthera brasiliana* (L.) Kuntze, *Epidendrum calanthum* Rchb.f. & Warsz., *Solanum mammosum* L., *Brugmansia candida* Pers.

Fungus

377. *Dioscorea trifida* L.f., *Piper aduncum* L., *Monactis flaverioides* Kunth, *Malva sylvestris* L., *Pelargonium odoratissimum* (L.) L'Hér., *Malva parviflora* L., *Alcea rosea* L., *Caesalpinia spinosa* (Molina) Kuntze

378. *Caesalpinia spinosa* (Molina) Kuntze, *Nerium oleander* L., *Myrica pubescens* Humb. & Bonpl. ex Willd., *Monactis flaverioides* Kunth, *Artemisia absinthium* L., *Achyrocline alata* (Kunth) DC, *Aiouea dubia* (Kunth) Mez., *Nectandra reticulata* (Ruiz & Pav.) Mez
379. *Salvia ayavacensis* Kunth, *Senecio genisianus* Cuatrec., *Phytolacca bogotensis* Kunth, *Siphocampylus cutervensis* Zahlbr., *Daucus montanus* Humb. & Bonpl. ex Spreng., *Salvia rosmarinifolia* G. Don.
380. *Verbena litoralis* Kunth, *Piper aduncum* L., *Monactis flaverioides* Kunth, *Malva sylvestris* L., *Pelargonium odoratissimum* (L.) L'Hér., *Malva parviflora* L., *Alcea rosea* L., *Plantago linearis* Kunth, *Plantago major* L., *Eustephia coccinea* Cav.

Gallbladder

381. *Arctium lappa* L., *Bejaria aestuans* Mutis ex L., *Cenchrus echinatus* L., *Tribulus terrestris* L., *Baccharis genistelloides* (Lam.) Pers., *Bidens pilosa* L., *Centropogon* cf. *rufus* E. Wimm.
382. *Baccharis genistelloides* (Lam.) Pers., *Schkuhria pinnata* (Lam.) Kuntze ex Thell., *Polygala paniculata* L., *Verbena litoralis* Kunth, *Bidens pilosa* L., *Equisetum bogotense* Kunth, *Equisetum giganteum* L., *Cuphea strigulosa* Kunth, *Ipomoea batatas* (L.) Lam.
383. *Bidens pilosa* L., *Mauria heterophylla* Kunth, *Eugenia obtusifolia* Cambess., *Tiquilia paronychioides* (Phil.) A.T. Richardson, *Zea mays* L., *Equisetum bogotense* Kunth, *Equisetum giganteum* L., *Psidium guajava* L., *Sanguisorba minor* Scop., *Cestrum nocturnum* L.
384. *Picrosia longifolia* D. Don, *Verbena litoralis* Kunth, *Schkuhria pinnata* (Lam.) Kuntze ex Thell., *Polygala paniculata* L.
385. *Schkuhria pinnata* (Lam.) Kuntze ex Thell., *Urtica magellanica* Juss. ex Poir., *Urtica urens* L., *Alternanthera porrigens* (Jacq.) Kuntze, *Cuphea strigulosa* Kunth, *Adiantum concinnum* Humb. & Bonpl. Ex Willd., *Satureja pulchella* (Kunth) Briq., *Bejaria aestuans* Mutis ex L., *Peumus boldus* Molina, *Rorippa nasturtium-aquaticum* (L.) Hayek, *Buddleja utilis* Kraenzl., *Polygala paniculata* L.
386. *Tessaria integrifolia* Ruiz & Pav., *Equisetum bogotense* Kunth, *Equisetum giganteum* L., *Verbena litoralis* Kunth, *Mauria heterophylla* Kunth, *Iresine diffusa* Humb. & Bonpl. ex Willd., *Plantago sericea* subsp. *sericans* (Pilg.) Rahn, *Zea mays* L.
387. *Tiquilia paronychioides* (Phil.) A.T. Richardson, *Malva sylvestris* L., *Pelargonium odoratissimum* (L.) L'Hér., *Malva parviflora* L., *Alcea rosea* L., *Zea mays* L., *Equisetum bogotense* Kunth, *Equisetum giganteum* L., *Pilea microphylla* (L.) Liebm., *Buddleja utilis* Kraenzl., *Bejaria aestuans* Mutis ex L., *Cenchrus echinatus* L., *Tribulus terrestris* L., *Baccharis genistelloides* (Lam.) Pers., *Bidens pilosa* L., *Rorippa nasturtium-aquaticum* (L.) Hayek, *Typha angustifolia* L., *Bixa orellana* L., *Alternanthera porrigens* (Jacq.) Kuntze, *Cuphea strigulosa* Kunth, *Chenopodium quinoa* Willd.
388. *Capsella bursa-pastoris* (L.) Medik., *Mauria heterophylla* Kunth, *Verbena litoralis* Kunth, *Zea mays* L., *Buddleja utilis* Kraenzl., *Equisetum bogotense* Kunth, *Equisetum giganteum* L., *Tiquilia paronychioides* (Phil.) A.T. Richardson, *Geranium ayavacense* Willd. ex Kunth, *Geranium sessiliflorum* Cav., *Gentianella bicolor* (Wedd.) J.S. Pringle
389. *Centropogon* cf. *rufus* Wimm., *Bejaria aestuans* Mutis ex L., *Cenchrus echinatus* L., *Tribulus terrestris* L., *Baccharis genistelloides* (Lam.) Pers., *Bidens pilosa* L., *Arctium lappa* L.
390. *Phyllanthus niruri* L., *Phyllanthus stipulatus* (Raf.) G.L.Webster, *Equisetum bogotense* Kunth, *Equisetum giganteum* L., *Plantago major* L., *Plantago linearis* Kunth, *Peumus boldus* Molina, *Cordia lutea* Lam., *Lycaste gigantea* Lindl., *Buddleja utilis* Kraenzl., *Tiquilia paronychioides* (Phil.) A.T. Richardson
391. *Linum usitatissimum* L., *Linum sativum* L., *Equisetum bogotense* Kunth, *Equisetum giganteum* L., *Phyllanthus niruri* L., *Phyllanthus stipulatus* (Raf.) G.L.Webster, *Phyllanthus urinaria* L., *Lycaste gigantea* Lindl., *Peumus boldus* Molina, *Cordia lutea* Lam.
392. *Tribulus terrestris* L., *Cenchrus echinatus* L., *Bidens pilosa* L., *Arctium lappa* L., *Centropogon* cf. *rufus* E.Wimm

Gases

393. *Apium graveolens* L., *Matricaria frigidum* (H.B.K.) Kunth, *Matricaria recutita* L., *Ambrosia peruviana* Willd., *Origanum majorana* L., *Adiantum concinnum* Humb. & Bonpl. Ex Willd.
394. *Foeniculum vulgare* Mill., *Matricaria frigidum* (H.B.K.) Kunth, *Matricaria recutita* L., *Ambrosia peruviana* Willd., *Mentha x piperita* L., *Melissa officinalis* L., *Sanguisorba minor* Scop., *Dianthus caryophyllus* L., *Tagetes erecta* L., *Borago officinalis* L.
395. *Sanguisorba minor* Scop., *Pimpinella anisum* L., *Mentha spicata* L.,
396. *Tillandsia cacticola* L.B. Sm., *Salvia rosmarinifolia* G. Don., *Rosmarinus officinalis* L., *Bursera graveolens* (Kunth) Triana & Planch., *Lavandula angustifolia* Mill.
397. *Lavandula angustifolia* Mill., *Salvia rosmarinifolia* G. Don., *Rosmarinus officinalis* L., *Dianthus caryophyllus* L., *Tagetes erecta* L., *Foeniculum vulgare* Mill., *Melissa officinalis* L., *Matricaria frigidum* (H.B.K.) Kunth, *Matricaria recutita* L., *Ambrosia peruviana* Willd., *Sanguisorba minor* Scop.
398. *Rosmarinus officinalis* L., *Eucalyptus globulus* Labill., *Equisetum bogotense* Kunth, *Equisetum giganteum* L., *Ruta graveolens* L.
399. *Cuphea strigulosa* Kunth, *Peperomia inaequalifolia* Ruiz & Pav., *Dianthus caryophyllus* L., *Tagetes erecta* L., *Lonicera japonica* Thunb. Ex Murray, *Urtica magellanica* Juss. ex Poir., *Urtica urens* L., *Alternanthera halimifolia* (Lam.) Standl. Ex Pittier, *Alternanthera porrigens* (Jacq.) Kuntze, *Pilea microphylla* (L.) Liebm., *Iresine herbstii* Hook., *Erodium cicutarium* (L.) L'Hér. Ex Aiton, *Desmodium molliculum* (Kunth) DC., *Equisetum bogotense* Kunth, *Equisetum giganteum* L., *Verbena litoralis* Kunth, *Sanguisorba minor* Scop., *Buddleja utilis* Kraenzl., *Cynodon dactylon* (L.) Pers., *Bejaria aestuans* Mutis ex L., *Cenchrus echinatus* L., *Tribulus terrestris* L., *Baccharis genistelloides* (Lam.) Pers., *Bidens pilosa* L.
400. *Siparuna muricata* (Ruiz & Pav.) A. DC., *Monactis flaverioides* Kunth , *Achyrocline alata* (Kunth) DC, *Aiouea dubia* (Kunth) Mez, *Nectandra reticulata* (Ruiz & Pav.) Mez *Rosmarinus officinalis* L., *Salvia rosmarinifolia* G. Don., *Ruta graveolens* L., *Escallonia pendula* (Ruiz & Pav.) Pers., *Bursera graveolens* (Kunth) Triana & Planch.
401. *Myristica fragrans* Houtt., *Brosimum rubescens* Taub., *Celtis loxensis* C.C. Berg, *Laccopetalum giganteum* (Wedd.) Ulbr., *Corynaea crassa* Hook.f., *Eustephia coccinea* Cav.

Gastritis

402. *Annona muricata* L., *Bidens pilosa* L., *Ananas comosus* (L.) Merr., *Bixa orellana* L.
403. *Apium graveolens* L., *Matricaria frigidum* (H.B.K.) Kunth, *Matricaria recutita* L., *Ambrosia peruviana* Willd., *Origanum majorana* L., *Adiantum concinnum* Humb. & Bonpl. Ex Willd.

404. *Mentha spicata* L., *Pimpinella anisum* L.
405. *Apium graveolens* L., *Petroselinum crispum* (Mill.) Fuss
406. *Gentianella crassicaulis* J.S. Pringle, *Geranium ayavacense* Willd. ex Kunth, *Geranium sessiliflorum* Cav., *Taraxacum officinale* F.H.Wigg., *Gentianella dianthoides* (Kunth) Fabris ex J.S. Pringle, *Gentianella bicolor* (Wedd.) J.S. Pringle

Good Business, Health

407. *Diplostephium sagasteguii* Cuatrec., *Alternanthera brasiliana* (L.) Kuntze, *Epidendrum calanthum* Rchb.f. & Warsz., *Solanum mammosum* L., *Brugmansia candida* Pers.

Hair loss

408. *Adiantum concinnum* Humb. & Bonpl. Ex Willd., *Bejaria aestuans* Mutis ex L., *Alternanthera porrigens* (Jacq.) Kuntze, *Cuphea strigulosa* Kunth, *Alternanthera halimifolia* (Lam.) Standl. Ex Pittier, *Alternanthera brasiliana* (L.) Kuntze, *Jamesonia rotundifolia* Fée, *Origanum vulgare* L.
409. *Bidens pilosa* L., *Mauria heterophylla* Kunth, *Eugenia obtusifolia* Cambess., *Tiquilia paronychioides* (Phil.) A.T. Richardson, *Zea mays* L., *Equisetum bogotense* Kunth, *Equisetum giganteum* L., *Psidium guajava* L., *Sanguisorba minor* Scop., *Cestrum nocturnum* L.
410. *Lepechinia meyenii* (Walp.) Epling, *Rosmarinus officinalis* L., *Salvia rosmarinifolia* G. Don., *Plantago linearis* Kunth, *Plantago major* L.
411. *Urtica magellanica* Juss. ex Poir., *Oritrophium peruvianum* (Lam.) Cuatrec., *Laccopetalum giganteum* (Wedd.) Ulbr., *Senecio tephrosioides* Turcz., , *Verbena litoralis* Kunth, *Gentianella bicolor* (Wedd.) J.S. Pringle, *Sonchus oleraceus* L., *Juglans neotropica* Diels, *Schinus molle* L., *Ruta graveolens* L., *Piper aduncum* L.

Hallucinogen

412. *Senecio chionogeton* Wedd., *Gentianella bicolor* (Wedd.) J.S. Pringle, *Echinopsis pachanoi* (Britton & Rose) Friedrich & G.D. Rowley
413. *Senecio chionogeton* Wedd., *Brugmansia arborea* (L.) Lagerh., *Brugmansia candida* Pers., *Brugmansia arborea* (L.) Lagerh., *Gaultheria reticulata* Kunth, *Solanum mammosum* L.
414. *Gaultheria reticulata* Kunth, *Brugmansia candida* Pers., *Brugmansia arborea* (L.) Lagerh., *Brugmansia sanguinea* (Ruiz & Pav.) D. Don, *Brugmansia sanguinea* (Ruiz & Pav.) D. Don, *Brugmansia candida* Pers.
415. *Rosmarinus officinalis* L., *Juglans neotropica* Diels, *Bidens pilosa* L., *Equisetum bogotense* Kunth, *Equisetum giganteum* L.

Hangover

416. *Salix chilensis* Molina, *Prunus serotina* subsp.*capuli* (Cav.) McVaugh
417. *Brugmansia arborea* (L.) Lagerh., *Gaultheria reticulata* Kunth, *Brugmansia sanguinea* (Ruiz & Pav.) D. Don, *Echinopsis pachanoi* (Britton & Rose) Friedrich & G.D. Rowley

Headache

418. *Cronquistianthus lavandulifolius* (DC.) R.M. King & H. Rob., *Piper aduncum* L., *Rubus robustus* C. Presl., *Juglans neotropica* Diels, *Lepechinia meyenii* (Walp.) Epling, *Salvia officinalis* L., *Salvia cuspidata* Ruiz & Pav., *Salvia sagittata* Ruiz. & Pav., *Borago officinalis* L., *Salvia discolor* Kunth, *Ambrosia peruviana* Willd., *Miconia salicifolia* (Bonpl. ex Naudin) Naudin, *Oritrophium peruvianum* (Lam.) Cuatrec., *Senecio canescens* (Bonpl.) Cuatrec.
419. *Hypericum aciculare* Kunth, *Ricinus communis* L., *Cestrum auriculatum* L'Hér.
420. *Myroxylon balsamum* (L.) Harms, *Achyrocline alata* (Kunth) DC, *Aiouea dubia* (Kunth) Mez., *Nectandra reticulata* (Ruiz & Pav.) Mez, *Couepia* sp., *Trichilia* sp., *Strychnos* sp., *Myristica fragrans* Houtt., *Cymbopogon citratus* (DC.) Stapf, *Aloysia triphylla* Royle, *Nicotiana tabacum* L., *Allium sativum* L., *Laccopetalum giganteum* (Wedd.) Ulbr., *Eucalyptus globulus* Labill.
421. *Rosmarinus officinalis* L., *Eucalyptus globulus* Labill., *Equisetum bogotense* Kunth, *Equisetum giganteum* L., *Ruta graveolens* L.

Heart

422. *Alternanthera halimifolia* (Lam.) Standl. Ex Pittier, *Melissa officinalis* L., *Matricaria frigidum* (H.B.K.) Kunth, *Matricaria recutita* L., *Ambrosia peruviana* Willd., *Rosmarinus officinalis* L., *Salvia rosmarinifolia* G. Don., *Foeniculum vulgare* Mill., *Marrubium vulgare* L., *Desmodium molliculum* (Kunth) DC., *Minthostachys mollis* (Kunth) Griseb. , *Cydonia oblonga* Mill..
423. *Iresine herbstii* Hook., *Pilea microphylla* (L.) Liebm.
424. *Apium graveolens* L., *Matricaria frigidum* (H.B.K.) Kunth, *Matricaria recutita* L., *Ambrosia peruviana* Willd., *Origanum majorana* L., *Adiantum concinnum* Humb. & Bonpl. Ex Willd.
425. *Petroselinum crispum* (Mill.) Fuss, *Melissa officinalis* L., *Sanguisorba minor* Scop., *Origanum majorana* L., *Tillandsia cacticola* L.B. Sm., *Tillandsia multiflora* var. *decipiens* (André) L.B. Sm., *Echeveria peruviana* Meyen
426. *Bidens pilosa* L., *Mauria heterophylla* Kunth, *Eugenia obtusifolia* Cambess., *Tiquilia paronychioides* (Phil.) A.T. Richardson, *Zea mays* L., *Equisetum bogotense* Kunth, *Equisetum giganteum* L., *Psidium guajava* L., *Sanguisorba minor* Scop., *Cestrum nocturnum* L.
427. *Borago officinalis* L., *Oritrophium peruvianum* (Lam.) Cuatrec., *Senecio canescens* (Bonpl.) Cuatrec.
428. *Tillandsia cacticola* L.B. Sm., *Rosmarinus officinalis* L. *Bursera graveolens* (Kunth) Triana & Planch., *Lavandula angustifolia* Mill.
429. *Tillandsia multiflora* var. *decipiens* (André) L.B. Sm., *Melissa officinalis* L., *Dianthus caryophyllus* L., *Citrus limetta* Risso
430. *Dianthus caryophyllus* L., *Baccharis salicifolia* (Ruiz & Pav.) Pers., *Peperomia fraseri* C. DC., *Peperomia hartwegiana* Miq., *Hesperoxiphion niveum* (Ravenna) Ravenna, *Ruta graveolens* L., *Rosmarinus officinalis* L., *Salvia rosmarinifolia* G. Don.
431. *Sanguisorba minor* Scop., *Matricaria frigidum* (H.B.K.) Kunth, *Matricaria recutita* L., *Ambrosia peruviana* Willd., *Melissa officinalis* L., Barrojo, *Rosmarinus officinalis* L., *Salvia rosmarinifolia* G. Don., *Desmodium molliculum* (Kunth) DC., *Minthostachys mollis* (Kunth) Griseb.
432. *Lathyrus odoratus* L., *Melissa officinalis* L., *Sanguisorba minor* Scop., *Origanum majorana* L., *Cymbopogon citratus* (DC.) Stapf, *Aloysia triphylla* Royle

433. *Lavandula angustifolia* Mill., *Rosmarinus officinalis* L., *Salvia rosmarinifolia* G. Don., *Dianthus caryophyllus* L., *Tagetes erecta* L., *Sanguisorba minor* Scop.
434. *Melissa officinalis* L., *Sanguisorba minor* Scop., *Cymbopogon citratus* (DC.) Stapf, *Aloysia triphylla* Royle, *Origanum majorana* L., *Tillandsia cacticola* L.B. Sm., *Tillandsia multiflora* var. *decipiens* (André) L.B. Sm., *Echeveria peruviana* Meyen, *Rosmarinus officinalis* L., *Salvia rosmarinifolia* G. Don., *Dianthus caryophyllus* L., *Tagestes erecta* L., *Peperomia inaequalifolia* Ruiz & Pav., *Matricaria frigidum* (H.B.K.) Kunth, *Matricaria recutita* L., *Ambrosia peruviana* Willd., *Citrus sinensis* (L.) Osbeck
435. *Origanum majorana* L., *Alternanthera halimifolia* (Lam.) Standl. Ex Pittier, *Peperomia inaequalifolia* Ruiz & Pav., *Melissa officinalis* L., *Foeniculum vulgare* Mill., *Hyptis sidifolia* (L'Hér.) Briq., *Mentha x piperita* L., *Matricaria frigidum* (H.B.K.) Kunth, *Matricaria recutita* L., *Ambrosia peruviana* Willd.
436. *Rosmarinus officinalis* L., *Eucalyptus globulus* Labill., *Equisetum bogotense* Kunth, *Equisetum giganteum* L., *Ruta graveolens* L.
437. *Cuphea strigulosa* Kunth, *Peperomia inaequalifolia* Ruiz & Pav., *Dianthus caryophyllus* L., *Tagestes erecta* L., *Lonicera japonica* Thunb. Ex Murray, *Urtica magellanica* Juss. ex Poir., *Urtica urens* L., *Alternanthera halimifolia* (Lam.) Standl. Ex Pittier, *Alternanthera porrigens* (Jacq.) Kuntze, *Alternanthera brasiliana* (L.) Kuntze, *Jamesonia rotundifolia* Fée, *Pilea microphylla* (L.) Liebm., *Iresine herbstii* Hook., *Erodium cicutarium* (L.) L'Hér. Ex Aiton, *Desmodium molliculum* (Kunth) DC., *Equisetum bogotense* Kunth, *Equisetum giganteum* L., *Verbena litoralis* Kunth, *Sanguisorba minor* Scop., *Buddleja utilis* Kraenzl., *Cynodon dactylon* (L.) Pers., *Bejaria aestuans* Mutis ex L., *Cenchrus echinatus* L., *Tribulus terrestris* L., *Baccharis genistelloides* (Lam.) Pers., *Bidens pilosa* L.
438. *Malva sylvestris* L., *Melissa officinalis* L., *Sanguisorba minor* Scop., *Origanum majorana* L., *Viola tricolor* L., *Lathyrus odoratus* L., *Cymbopogon citratus* (DC.) Stapf, *Aloysia triphylla* Royle
439. *Oxalis bulbigera* R. Knuth., *Melissa officinalis* L., *Mentha x piperita* L., *Matricaria frigidum* (H.B.K.) Kunth, *Matricaria recutita* L., *Ambrosia peruviana* Willd., *Foeniculum vulgare* Mill., *Rosmarinus officinalis* L., *Salvia rosmarinifolia* G. Don.
440. *Peperomia galioides* Kunth, *Pelargonium odoratissimum* (L.) L'Hér., *Tillandsia cacticola* L.B. Sm., *Tillandsia multiflora* var. *decipiens* (André) L.B. Sm., *Echeveria peruviana* Meyen, *Pilea microphylla* (L.) Liebm.
441. *Peperomia inaequalifolia* Ruiz & Pav., *Melissa officinalis* L., *Sanguisorba minor* Scop., *Origanum majorana* L., *Viola tricolor* L., *Lathryus odoratus* L.
442. *Laccopetalum giganteum* (Wedd.) Ulbr., *Peperomia inaequalifolia* Ruiz & Pav.
443. *Cydonia oblonga* Mill., *Matricaria recutita* L., *Melissa officinalis* L., *Foeniculum vulgare* Mill., *Lonicera japonica* Thunb. Ex Murray, *Rosmarinus officinalis* L., *Salvia rosmarinifolia* G. Don.
444. *Cydonia oblonga* Mill., *Matricaria frigidum* (H.B.K.) Kunth, *Matricaria recutita* L., *Ambrosia peruviana* Willd., *Melissa officinalis* L., *Viola tricolor* L., *Lathryus odoratus* L., *Borago officinalis* L., *Origanum majorana* L., *Rosmarinus officinalis* L., *Salvia rosmarinifolia* G. Don.
445. *Fragaria vesca* L., *Foeniculum vulgare* Mill., *Matricaria frigidum* (H.B.K.) Kunth, *Matricaria recutita* L., *Ambrosia peruviana* Willd., *Melissa officinalis* L, *Viola tricolor* L., *Lathryus odoratus* L.
446. *Sanguisorba minor* Scop., *Bejaria aestuans* Mutis ex L., *Alternanthera porrigens* (Jacq.) Kuntze, *Cuphea strigulosa* Kunth, *Melissa officinalis* L., *Peperomia inaequalifolia* Ruiz & Pav., *Dianthus caryophyllus* L., *Matricaria frigidum* (H.B.K.) Kunth, *Matricaria recutita* L., *Ambrosia peruviana* Willd., *Cestrum nocturnum* L.
447. *Ruta graveolens* L., *Gentianella dianthoides* (Kunth) Fabris ex J.S. Pringle, *Gentianella bicolor* (Wedd.) J.S. Pringle, *Lepechinia meyenii* (Walp.) Epling, *Salvia officinalis* L., *Salvia cuspidata* Ruiz & Pav., *Salvia sagittata* Ruiz. & Pav., *Origanum vulgare* L., *Schinus molle* L., *Eucalyptus globulus* Labill., *Ambrosia peruviana* Willd., *Artemisia absinthium* L., *Adiantum concinnum* Humb. & Bonpl. Ex Willd.
448. *Populus deltoides* W. Bartram ex Marshall, *Matricaria frigidum* (H.B.K.) Kunth, *Matricaria recutita* L., *Ambrosia peruviana* Willd., *Melissa officinalis* L., *Sanguisorba minor* Scop., *Foeniculum vulgare* Mill., *Marrubium vulgare* L., *Desmodium molliculum* (Kunth) DC., *Minthostachys mollis* (Kunth) Griseb., *Cydonia oblonga* Mill.
449. *Smilax medica* Schltdl. & Cham., *Peperomia inaequalifolia* Ruiz & Pav., *Piper aduncum* L., *Equisetum bogotense* Kunth, *Equisetum giganteum* L.
450. *Viola tricolor* L., *Melissa officinalis* L.

Hemorrhages

451. *Eustephia coccinea* Cav., *Buddleja utilis* Kraenzl. *Bejaria aestuans* Mutis ex L., *Pelargonium odoratissimum* (L.) L'Hér.
452. *Acanthoxanthium spinosum* (L.) Fourr., *Senna bicapsularis* (L.) Roxb., *Inga edulis* C. Martius, *Inga feuillei* DC.
453. *Bixa orellana* L., *Uncaria tomentosa* (Willd. ex Roem. & Schult.) DC., *Mimosa nothacacia* Barneby
454. *Equisetum giganteum* L., *Verbena litoralis* Kunth, *Matricaria frigidum* (H.B.K.) Kunth, *Matricaria recutita* L., *Ambrosia peruviana* Willd, *Mauria heterophylla* Kunth, *Eugenia obtusifolia* Cambess., *Zea mays* L., *Iresine diffusa* Humb. & Bonpl. ex Willd., *Scirpus californicus* subsp. *tatora* (Kunth) T. Koyama, *Plantago sericea* subsp. *sericans* (Pilg.) Rahn, *Rorippa nasturtium-aquaticum* (L.) Hayek, *Desmodium molliculum* (Kunth) DC., *Dioscorea trifida* L.f.
455. *Piper aduncum* L., *Lepechinia meyenii* (Walp.) Epling, *Perezia multiflora* (Bonpl.) Less., *Oritrophium peruvianum* (Lam.) Cuatrec., *Senecio canescens* (Bonpl.) Cuatrec., *Borago officinalis* L., *Eupatorium gayanum* Wedd.
456. *Plantago major* L., *Piper aduncum* L., *Artemisia absinthium* L., *Lepechinia meyenii* (Walp.) Epling, *Senna occidentalis* (L.) Link
457. *Uncaria tomentosa* (Willd. ex Roem. & Schult.) DC., *Phyllanthus niruri* L., *Phyllanthus stipulatus* (Raf.) G.L.Webster, *Phyllanthus urinaria* L., *Linum sativum* L., *Linum usitatissimum* L., *Peumus boldus* Molina, *Cordia lutea* Lam., *Capsella bursa-pastoris* (L.) Medik.
458. *Celtis loxensis* C.C. Berg, *Brosimum rubescens* Taub., *Laccopetalum giganteum* (Wedd.) Ulbr., *Heisteria acuminata* (Humb. & Bonpl.) Engl., *Cinchona officinalis* L., *Corynaea crassa* Hook.f.
459. *Urtica magellanica* Juss. ex Poir., *Oritrophium peruvianum* (Lam.) Cuatrec., *Laccopetalum giganteum* (Wedd.) Ulbr., *Senecio tephrosioides* Turcz., *Verbena litoralis* Kunth, *Gentianella bicolor* (Wedd.) J.S. Pringle, *Bidens pilosa* L., *Baccharis genistelloides* (Lam.) Pers., *Juglans neotropica* Diels, *Schinus molle* L., *Ruta graveolens* L., *Piper aduncum* L.

Hemorrhoids

460. *Urtica magellanica* Juss. ex Poir., *Oritrophium peruvianum* (Lam.) Cuatrec., *Laccopetalum giganteum* (Wedd.) Ulbr., *Senecio tephrosioides* Turcz., *Malesherbia ardens* J.F. Macbr., *Alternanthera brasiliana* (L.) Kuntze, *Jamesonia rotundifolia* Fée, *Stachys lanata* Jacq., *Gentianella bicolor* (Wedd.) J.S. Pringle, *Baccharis genistelloides* (Lam.) Pers., *Juglans neotropica* Diels, *Schinus molle* L., *Ruta graveolens* L., *Piper aduncum* L.

Hepatitis

461. *Picrosia longifolia* D. Don, *Verbena litoralis* Kunth, *Schkuhria pinnata* (Lam.) Kuntze ex Thell., *Polygala paniculata* L.
462. *Berberis buceronis* J.F. Macbr., *Bidens pilosa* L., *Equisetum bogotense* Kunth, *Equisetum giganteum* L.
463. *Cordia lutea* Lam., *Plantago major* L., *Plantago linearis* Kunth, *Peumus boldus* Molina,
464. *Spartium junceum* L., *Cordia lutea* Lam.
465. *Piper* cf. *aequale* Vahl, *Cordia lutea* Lam., *Peumus boldus* Molina
466. *Portulaca oleracea* subsp. *tuberculata* Danin & H.G. Baker, *Peumus boldus* Molina, *Tiquilia paronychioides* (Phil.) A.T. Richardson, *Equisetum bogotense* Kunth, *Equisetum giganteum* L.

Hyperactivity

467. *Verbena litoralis* Kunth, *Piper aduncum* L., *Monactis flaverioides* Kunth, *Malva sylvestris* L., *Alcea rosea* L., *Pelargonium odoratissimum* (L.) L'Hér., *Malva parviflora* L.

Indigestion

468. *Mentha spicata* L., *Pimpinella anisum* L.
469. *Rosmarinus officinalis* L., *Eucalyptus globulus* Labill., *Equisetum bogotense* Kunth, *Equisetum giganteum* L., *Ruta graveolens* L.

Infection

470. *Petroselinum crispum* (Mill.) Fuss, *Melissa officinalis* L., *Sanguisorba minor* Scop., *Origanum majorana* L., *Tillandsia cacticola* L.B. Sm., *Tillandsia multiflora* var. *decipiens* (André) L.B. Sm., *Echeveria peruviana* Meyen
471. *Salvia discolor* Kunth, *Achyrocline alata* (Kunth) DC, *Porophyllum ruderale* (Jacq.) Cass., *Cordia alliodora* (Ruiz & Pav.) Oken
472. *Satureja pulchella* (Kunth) Briq., *Otholobium glandulosum* (L.) J.W. Grimes, *Matricaria frigidum* (H.B.K.) Kunth, *Matricaria recutita* L., *Ambrosia peruviana* Willd, *Desmodium molliculum* (Kunth) DC., *Marrubium vulgare* L., *Minthostachys mollis* (Kunth) Griseb.
473. *Cuphea strigulosa* Kunth, *Peperomia inaequalifolia* Ruiz & Pav., *Dianthus caryophyllus* L., *Tagetes erecta* L., *Lonicera japonica* Thunb. Ex Murray, *Urtica magellanica* Juss. ex Poir., *Urtica urens* L., *Alternanthera halimifolia* (Lam.) Standl. Ex Pittier *Alternanthera porrigens* (Jacq.) Kuntze, *Alternanthera brasiliana* (L.) Kuntze, *Jamesonia rotundifolia* Fée, *Pilea microphylla* (L.) Liebm., *Iresine herbstii* Hook., *Erodium cicutarium* (L.) L'Hér. Ex Aiton, *Desmodium molliculum* (Kunth) DC., *Equisetum bogotense* Kunth, *Equisetum giganteum* L., *Verbena litoralis* Kunth *Sanguisorba minor* Scop., *Buddleja utilis* Kraenzl., *Cynodon dactylon* (L.) Pers., *Bejaria aestuans* Mutis ex L., *Cenchrus echinatus* L., *Tribulus terrestris* L., *Baccharis genistelloides* (Lam.) Pers., *Bidens pilosa* L.
474. *Piper aduncum* L., *Lepechinia meyenii* (Walp.) Epling, *Eucalyptus globulus* Labill., *Nerium oleander* L., *Myrica pubescens* Humb. & Bonpl. ex Willd, *Verbena litoralis* Kunth, *Ambrosia peruviana* Willd.
475. *Piper* cf. *aequale* Vahl, *Cordia lutea* Lam., *Peumus boldus* Molina

Inflammation

476. *Eustephia coccinea* Cav., *Buddleja utilis* Kraenzl. *Bejaria aestuans* Mutis ex L., *Pelargonium odoratissimum* (L.) L'Hér.
477. *Mangifera indica* L., *Schinus molle* L., *Eucalyptus globulus* Labill., *Tessaria integrifolia* Ruiz & Pav., *Citrus limon* (L.) Burm. f.
478. *Mauria heterophylla* Kunth, *Equisetum bogotense* Kunth, *Equisetum giganteum* L., *Verbena litoralis* Kunth, *Bidens pilosa* L.
479. *Annona muricata* L., *Bidens pilosa* L., *Ananas comosus* (L.) Merr., *Bixa orellana* L.
480. *Acanthoxanthium spinosum* (L.) Fourr., *Senna bicapsularis* (L.) Roxb. *Inga edulis* C.Martius, *Inga feuillei* DC.
481. *Baccharis genistelloides* (Lam.) Pers., *Schkuhria pinnata* (Lam.) Kuntze ex Thell., *Polygala paniculata* L., *Verbena litoralis* Kunth, *Bidens pilosa* L., *Equisetum bogotense* Kunth, *Equisetum giganteum* L., *Cuphea strigulosa* Kunth, *Ipomoea batatas* (L.) Lam .
482. *Bidens pilosa* L., *Mauria heterophylla* Kunth, *Eugenia obtusifolia* Cambess., *Tiquilia paronychioides* (Phil.) A.T. Richardson, *Zea mays* L., *Equisetum bogotense* Kunth, *Equisetum giganteum* L., *Sanguisorba minor* Scop., *Cestrum nocturnum* L.
483. *Paranephelius uniflorus* Poepp., *uddleja utilis* Kraenzl., *Bejaria aestuans* Mutis ex L., *Tiquilia paronychioides* (Phil.) A.T. Richardson, *Desmodium molliculum* (Kunth) DC., *Sambucus peruviana* Kunth, *Equisetum bogotense* Kunth, *Equisetum giganteum* L.
484. *Senecio chionogeton* Wedd., *Gentianella bicolor* (Wedd.) J.S. Pringle, *Echinopsis pachanoi* (Britton & Rose) Friedrich & G.D. Rowley
485. *Tagetes erecta* L., *Melissa officinalis* L., *Sanguisorba minor* Scop., *Mentha x piperita* L., *Matricaria frigidum* (H.B.K.) Kunth, *Matricaria recutita* L., *Ambrosia peruviana* Willd
486. *Taraxacum officinale* F.H.Wigg., *Mauria heterophylla* Kunth, *Desmodium molliculum* (Kunth) DC., *Equisetum bogotense* Kunth, *Equisetum giganteum* L., *Linum sativum* L., *Linum usitatissimum* L., *Monactis flaverioides* Kunth , *Malva sylvestris* L., *Pelargonium odoratissimum* (L.) L'Hér., *Malva parviflora* L., *Bidens pilosa* L., *Aristolochia ruiziana* (Klotzsch) Duch.
487. *Tessaria integrifolia* Ruiz & Pav., *Equisetum bogotense* Kunth, *Equisetum giganteum* L., *Verbena litoralis* Kunth, *Mauria heterophylla* Kunth, *Iresine diffusa* Humb. & Bonpl. ex Willd., *Plantago sericea* subsp. *sericans* (Pilg.) Rahn, *Zea mays* L.
488. *Tiquilia paronychioides* (Phil.) A.T. Richardson, *Monactis flaverioides* Kunth , *Malva sylvestris* L., *Pelargonium odoratissimum* (L.) L'Hér., *Malva parviflora* L., *Zea mays* L., *Equisetum bogotense* Kunth, *Equisetum giganteum* L., *Pilea microphylla* (L.) Liebm., *Buddleja utilis* Kraenzl., *Bejaria aestuans* Mutis ex L., *Cenchrus echinatus* L., *Tribulus terrestris* L., *Baccharis genistelloides* (Lam.) Pers., *Bidens pilosa* L., *Rorippa nasturtium-aquaticum* (L.) Hayek, *Typha angustifolia* L., *Bixa orrellana* L., *Alternanthera porrigens* (Jacq.) Kuntze, *Cuphea strigulosa* Kunth, *Eustephia coccinea* Cav.

489. *Capsella bursa-pastoris* (L.) Medik., *Mauria heterophylla* Kunth, *Verbena litoralis* Kunth, *Zea mays* L., *Buddleja utilis* Kraenzl., *Tiquilia paronychioides* (Phil.) A.T. Richardson, *Geranium ayavacense* Willd. ex Kunth, *Geranium sessiliflorum* Cav., *Gentianella bicolor* (Wedd.) J.S. Pringle, *Equisetum bogotense* Kunth, *Equisetum giganteum* L.
490. *Rorippa nasturtium-aquaticum* (L.) Hayek, *Monactis flaverioides* Kunth, *Malva sylvestris* L., *Pelargonium odoratissimum* (L.) L'Hér., *Malva* parviflora L., *Desmodium molliculum* (Kunth) DC., *Eugenia obtusifolia* Cambess., *Bidens pilosa* L., *Mauria heterophylla* Kunth, *Iresine diffusa* Humb. & Bonpl. ex Willd., *Plantago sericea* subsp. *sericans* (Pilg.) Rahn, *Tiquilia paronychioides* (Phil.) A.T. Richardson, *Bejaria aestuans* Mutis ex L.
491. *Cyclanthera pedata* (L.) Schrad., *Sechium edule* (Jacq.) Sw., *Daucus carota* L.,
492. *Dioscorea tambillensis* R. Knuth, *Bidens pilosa* L., *Mauria heterophylla* Kunth, *Equisetum bogotense* Kunth, *Equisetum giganteum* L., *Desmodium molliculum* (Kunth) DC., *Verbena litoralis* Kunth, *Linum sativum* L., *Linum usitatissimum* L., *Hordeum vulgare* L.
493. *Dioscorea trifida* L.f., *Buddleja utilis* Kraenzl., *Bejaria aestuans* Mutis ex L., *Paranephelius uniflorus* Poepp., *Rorippa nasturtium-aquaticum* (L.) Hayek
494. *Equisetum bogotense* Kunth, *Desmodium molliculum* (Kunth) DC., *Mauria heterophylla* Kunth
495. *Chamaesyce hypericifolia* (L.) Millsp., *Equisetum bogotense* Kunth, *Equisetum giganteum* L., *Bidens pilosa* L., *Linum sativum* L., *Linum usitatissimum* L., *Mauria heterophylla* Kunth, *Sarcostemma clausum* (Jacq.) Schult.
496. *Phyllanthus niruri* L., *Equisetum bogotense* Kunth, *Equisetum giganteum* L., *Plantago linearis* Kunth, *Plantago major* L., *Peumus boldus* Molina, *Cordia lutea* Lam., *Lycaste gigantea* Lindl., *Buddleja utilis* Kraenzl., *Tiquilia paronychioides* (Phil.) A.T. Richardson
497. *Desmodium molliculum* (Kunth) DC., *Mauria heterophylla* Kunth, *Bidens pilosa* L., *Verbena litoralis* Kunth
498. *Senna occidentalis* (L.) Link, *Bidens pilosa* L., *Equisetum bogotense* Kunth, *Equisetum giganteum* L., *Linum sativum* L., *Linum usitatissimum* L., *Mauria heterophylla* Kunth, *Desmodium molliculum* (Kunth) DC.
499. *Trifolium repens* L., *Alternanthera porrigens* (Jacq.) Kuntze, *Cuphea strigulosa* Kunth, *Smilax medica* Schltdl. & Cham.
500. *Erodium cicutarium* (L.) L'Hér. Ex Aiton, *Scabiosa atropurpurea* L., *Cuphea strigulosa* Kunth, *Alternanthera halimifolia* (Lam.) Standl. Ex Pittier
501. *Geranium ayavacense* Willd. ex Kunth, *Geranium sessiliflorum* Cav., *Mauria heterophylla* Kunth, *Equisetum bogotense* Kunth, *Equisetum giganteum* L., *Verbena litoralis* Kunth, *Eugenia obtusifolia* Cambess., *Bidens pilosa* L., *Cynodon dactylon* (L.) Pers.
502. *Marrubium vulgare* L., *Eucalyptus globulus* Labill., *Achyrocline alata* (Kunth) DC, *Aiouea dubia* (Kunth) Mez., *Nectandra reticulata* (Ruiz & Pav.) Mez., *Polylepis racemosa* Ruiz & Pav.
503. *Salvia rosmarinifolia* G. Don., *Lepechinia meyenii* (Walp.) Epling, *Salvia officinalis* L., *Salvia cuspidata* Ruiz & Pav., *Salvia sagittata* Ruiz. & Pav., *Origanum majorana* L.
504. *Salvia rosmarinifolia* G. Don., *Bursera graveolens* (Kunth) Triana & Planch., *Eucalyptus globulus* Labill.
505. *Cuphea strigulosa* Kunth, *Peperomia inaequalifolia* Ruiz & Pav., *Dianthus caryophyllus* L., *Tagetes erecta* L., *Lonicera japonica* Thunb. Ex Murray, *Urtica magellanica* Juss. ex Poir., *Urtica urens* L., *Alternanthera halimifolia* (Lam.) Standl. Ex Pittier, *Alternanthera porrigens* (Jacq.) Kuntze, *Alternanthera brasiliana* (L.) Kuntze, *Jamesonia rotundifolia* Fée, *Pilea microphylla* (L.) Liebm., *Iresine herbstii* Hook., *Erodium cicutarium* (L.) L'Hér. Ex Aiton, *Desmodium molliculum* (Kunth) DC., *Equisetum bogotense* Kunth, *Equisetum giganteum* L., *Verbena litoralis* Kunth, *Sanguisorba minor* Scop., *Buddleja utilis* Kraenzl., *Cynodon dactylon* (L.) Pers., *Bejaria aestuans* Mutis ex L., *Cenchrus echinatus* L., *Tribulus terrestris* L., *Baccharis genistelloides* (Lam.) Pers., *Bidens pilosa* L.
506. *Malva parviflora* L., *Desmodium molliculum* (Kunth) DC., *Mauria heterophylla* Kunth, *Verbena litoralis* Kunth, *Equisetum bogotense* Kunth, *Equisetum giganteum* L., *Bidens pilosa* L., *Monactis flaverioides* Kunth, *Malva sylvestris* L., *Pelargonium odoratissimum* (L.) L'Hér., *Malva parviflora* L
507. *Mirabilis jalapa* L., *Tiquilia paronychioides* (Phil.) A.T. Richardson
508. *Argemone mexicana* L., *Equisetum bogotense* Kunth, *Equisetum giganteum* L., *Monactis flaverioides* Kunth, *Malva sylvestris* L., *Alcea rosea* L., *Pelargonium odoratissimum* (L.) L'Hér., *Malva parviflora* L., *Plantago linearis* Kunth, *Plantago major* L., *Desmodium molliculum* (Kunth) DC.
509. *Plantago linearis* Kunth, *Piper aduncum* L.
510. *Uncaria tomentosa* (Willd. ex Roem. & Schult.) DC., *Phyllanthus niruri* L., *Phyllanthus stipulatus* (Raf.) G.L. Webster, *Phyllanthus urinaria* L., *Linum sativum* L., *Linum usitatissimum* L., *Peumus boldus* Molina, *Cordia lutea* Lam., *Capsella bursa-pastoris* (L.) Medik.
511. *Calceolaria rugulosa* Edwin, *Verbena litoralis* Kunth, *Equisetum bogotense* Kunth, *Equisetum giganteum* L., *Desmodium molliculum* (Kunth) DC., *Bidens pilosa* L., *Plantago linearis* Kunth, *Plantago major* L.
512. *Smilax medica* Schltdl. & Cham., *Peperomia inaequalifolia* Ruiz & Pav., Chajur, *Piper aduncum* L., *Equisetum bogotense* Kunth, *Equisetum giganteum* L.
513. *Passiflora ligularis* Juss., *Peumus boldus* Molina, *Equisetum bogotense* Kunth, *Equisetum giganteum* L., *Mauria heterophylla* Kunth, *Bidens pilosa* L.
514. *Piper aduncum* L., *Lepechinia meyenii* (Walp.) Epling, *Eucalyptus globulus* Labill., *Nerium oleander* L., *Myrica pubescens* Humb. & Bonpl. ex Willd., *Verbena litoralis* Kunth, *Ambrosia peruviana* Willd.
515. *Cenchrus echinatus* L., *Bidens pilosa* L., *Arctium lappa* L., *Centropogon* cf. *rufus* E. Wimm.
516. *Cynodon dactylon* (L.) Pers., *Equisetum bogotense* Kunth, *Equisetum giganteum* L., *Verbena litoralis* Kunth, *Bidens pilosa* L., *Monactis flaverioides* Kunth, *Malva sylvestris* L., *Alcea rosea* L., *Pelargonium odoratissimum* (L.) L'Hér., *Malva parviflora* L., *Buddleja utilis* Kraenzl., *Smilax medica* Schltdl. & Cham., *Cuphea strigulosa* Kunth

Inflammation of the Bladder

517. *Bejaria aestuans* Mutis ex L., *Buddleja utilis* Kraenzl., *Dioscorea trifida* L. f., *Tiquilia paronychioides* (Phil.) A.T. Richardson, *Ilex guayusa* Loes., *Geranium ayavacense* Willd. ex Kunth, *Geranium sessiliflorum* Cav., *Monactis flaverioides* Kunth, *Malva sylvestris* L., *Pelargonium odoratissimum* (L.) L'Hér., *Malva parviflora* L., *Bidens pilosa* L., *Verbena litoralis* Kunth, *Plantago linearis* Kunth, *Plantago major* L., *Equisetum bogotense* Kunth, *Equisetum giganteum* L., *Muehlenbeckia tamnifolia* (Kunth) Meisn., *Smilax kunthii* Killip & C.V. Morton, *Oreocallis grandiflora* (Lam.) R. Br., *Cinchona officinalis* L.

Inflammation of the Kidneys

518. *Bixa orellana* L., *Uncaria tomentosa* (Willd. ex Roem. & Schult.) DC., *Mimosa nothacacia* Barneby
519. *Cordia lutea* Lam, *Plantago linearis* Kunth, *Plantago major* L., *Peumus boldus* Molina,
520. *Tiquilia paronychioides* (Phil.) A.T. Richardson, *Monactis flaverioides* Kunth, *Malva sylvestris* L., *Pelargonium odoratissimum* (L.) L'Hér., *Malva parviflora* L., *Zea mays* L., *Equisetum bogotense* Kunth, *Equisetum giganteum* L., *Pilea microphylla* (L.) Liebm., *Buddleja utilis* Kraenzl., *Bejaria aestuans* Mutis ex L., *Cenchrus echinatus* L., *Tribulus terrestris* L., *Baccharis genistelloides* (Lam.) Pers., *Bidens pilosa* L., *Rorippa nasturtium-aquaticum* (L.) Hayek, *Typha angustifolia* L., *Bixa orellana* L., *Alternanthera porrigens* (Jacq.) Kuntze, *Cuphea strigulosa* Kunth, *Eustephia coccinea* Cav.
521. *Stellaria media* (L.) Vill., *Monactis flaverioides* Kunth, *Malva sylvestris* L., *Pelargonium odoratissimum* (L.) L'Hér., *Malva parviflora* L., *Bidens pilosa* L., *Mauria heterophylla* Kunth, *Eugenia obtusifolia* Cambess.
522. *Dioscorea tambillensis* R. Knuth, *Bidens pilosa* L., *Mauria heterophylla* Kunth, *Equisetum bogotense* Kunth, *Equisetum giganteum* L., *Desmodium molliculum (*Kunth*)* DC., *Verbena litoralis* Kunth, *Linum sativum* L., *Linum usitatissimum* L., *Hordeum vulgare* L.
523. *Equisetum bogotense* Kunth, *Cordia lutea* Lam, *Alternanthera porrigens* (Jacq.) Kuntze, *Cuphea strigulosa* Kunth, *Typha angustifolia* L., *Bixa orellana* L., *Smilax medica* Schltdl. & Cham.
524. *Bejaria aestuans* Mutis ex L., *Buddleja utilis* Kraenzl., *Dioscorea trifida* L. f., *Tiquilia paronychioides* (Phil.) A.T. Richardson, *Ilex guayusa* Loes., *Geranium ayavacense* Willd. ex Kunth, *Geranium sessiliflorum* Cav., *Monactis flaverioides* Kunth, *Malva sylvestris* L., *Pelargonium odoratissimum* (L.) L'Hér., *Malva parviflora* L., *Bidens pilosa* L., *Verbena litoralis* Kunth, *Plantago linearis* Kunth, *Plantago major* L., *Equisetum bogotense* Kunth, *Equisetum giganteum* L., *Muehlenbeckia tamnifolia* (Kunth) Meisn., *Smilax kunthii* Killip & C.V. Morton, *Oreocallis grandiflora* (Lam.) R. Br., *Cinchona officinalis* L.
525. *Desmodium molliculum* (Kunth) DC., *Mauria heterophylla* Kunth, *Bidens pilosa* L., *Verbena litoralis* Kunth
526. *Trifolium repens* L., *Alternanthera porrigens* (Jacq.) Kuntze, *Cuphea strigulosa* Kunth, *Iresine herbstii* Hook., *Smilax medica* Schltdl. & Cham.
527. *Geranium ayavacense* Willd. ex Kunth, *Geranium sessiliflorum* Cav., *Mauria heterophylla* Kunth, *Equisetum bogotense* Kunth, *Equisetum giganteum* L., *Verbena litoralis* Kunth, *Eugenia obtusifolia* Cambess., *Bidens pilosa* L., *Cynodon dactylon* (L.) Pers.
528. *Linum sativum* L., *Linum usitatissimum* L., *Equisetum bogotense* Kunth, *Equisetum giganteum* L., *Phyllanthus niruri* L., *Phyllanthus stipulatus* (Raf.) G.L.Webster, *Phyllanthus urinaria* L., *Lycaste gigantea* Lindl., *Peumus boldus* Molina, *Cordia lutea* Lam
529. *Peumus boldus* Molina, *Desmodium molliculum (*Kunth*)* DC., *Linum sativum* L., *Linum usitatissimum* L., *Rorippa nasturtium-aquaticum* (L.) Hayek, *Dioscorea trifida* L. f., *Zea mays* L., *Cordia lutea* Lam
530. *Lycaste gigantea* Lindl., *Linum sativum* L., *Linum usitatissimum* L., *Rorippa nasturtium-aquaticum* (L.) Hayek, *Desmodium molliculum (*Kunth*)* DC., *Dioscorea trifida* L. f., *Zea mays* L.
531. *Passiflora ligularis* Juss., *Peumus boldus* Molina, *Equisetum bogotense* Kunth, *Equisetum giganteum* L., *Mauria heterophylla* Kunth, *Bidens pilosa* L.
532. *Plantago linearis* Kunth, *Equisetum bogotense* Kunth, *Equisetum giganteum* L., *Mauria heterophylla* Kunth, *Eugenia obtusifolia* Cambess., *Cynodon dactylon* (L.) Pers., *Buddleja utilis* Kraenzl.
533. *Cynodon dactylon* (L.) Pers., *Equisetum bogotense* Kunth, *Equisetum giganteum* L., *Verbena litoralis* Kunth, *Bidens pilosa* L., *Monactis flaverioides* Kunth, *Malva sylvestris* L., *Pelargonium odoratissimum* (L.) L'Hér., *Malva parviflora* L., *Buddleja utilis* Kraenzl., *Bejaria aestuans* Mutis ex L., *Smilax medica* Schltdl. & Cham., *Cuphea strigulosa* Kunth
534. *Hordeum vulgare* L., *Linum sativum* L., *Linum usitatissimum* L., *Equisetum bogotense* Kunth, *Equisetum giganteum* L., *Bidens pilosa* L., *Monactis flaverioides* Kunth, *Malva sylvestris* L., *Pelargonium odoratissimum* (L.) L'Hér., *Malva parviflora* L.
535. *Saccharum officinarum* L., *Equisetum bogotense* Kunth, *Equisetum giganteum* L., *Linum sativum* L., *Linum usitatissimum* L., *Phyllanthus niruri* L., *Phyllanthus stipulatus* (Raf.) G.L.Webster, *Phyllanthus urinaria* L., *Peumus boldus* Molina, *Desmodium molliculum (*Kunth*)* DC.
536. *Polypodium crassifolium* L., *Desmodium molliculum (*Kunth*)* DC., *Bidens pilosa* L., *Equisetum bogotense* Kunth, *Equisetum giganteum* L.
537. *Oreocallis grandiflora* (Lam.) R. Br., *Buddleja utilis* Kraenzl., *Tiquilia paronychioides* (Phil.) A.T. Richardson
538. *Rubus robustus* C. Presl., *Alternanthera halimifolia* (Lam.) Standl. ex Pittier, *Bejaria aestuans* Mutis ex L., *Typha angustifolia* L.
539. *Smilax medica* Schltdl. & Cham., *Peperomia inaequalifolia* Ruiz & Pav., *Piper aduncum* L., *Equisetum bogotense* Kunth, *Equisetum giganteum* L.
540. *Pilea microphylla* (L.) Liebm., *Equisetum bogotense* Kunth, *Equisetum giganteum* L., *Bixa orellana* L., *Phyllanthus niruri* L., *Phyllanthus stipulatus* (Raf.) G.L.Webster, *Phyllanthus urinaria* L.

Inflammation of the Liver

541. *Bejaria aestuans* Mutis ex L., *Buddleja utilis* Kraenzl., *Dioscorea trifida* L. f., *Tiquilia paronychioides* (Phil.) A.T. Richardson, *Ilex guayusa* Loes., *Geranium ayavacense* Willd. ex Kunth, *Geranium sessiliflorum* Cav., *Monactis flaverioides* Kunth, *Malva sylvestris* L., *Pelargonium odoratissimum* (L.) L'Hér., *Malva parviflora* L., *Bidens pilosa* L., *Verbena litoralis* Kunth, *Plantago linearis* Kunth, *Plantago major* L., *Equisetum bogotense* Kunth, *Equisetum giganteum* L., *Muehlenbeckia tamnifolia* (Kunth) Meisn., *Smilax kunthii* Killip & C.V. Morton, *Oreocallis grandiflora* (Lam.) R. Br., *Cinchona officinalis* L.
542. *Phyllanthus stipulatus* (Raf.) G.L.Webster, *Phyllanthus niruri* L., *Equisetum bogotense* Kunth, *Equisetum giganteum* L., *Plantago linearis* Kunth, *Plantago major* L., *Peumus boldus* Molina, *Cordia lutea* Lam, *Lycaste gigantea* Lindl., *Buddleja utilis* Kraenzl., *Tiquilia paronychioides* (Phil.) A.T. Richardson
543. *Geranium ayavacense* Willd. ex Kunth, *Geranium sessiliflorum* Cav., *Mauria heterophylla* Kunth, *Equisetum bogotense* Kunth, *Equisetum giganteum* L., *Verbena litoralis* Kunth, *Eugenia obtusifolia* Cambess., *Bidens pilosa* L., *Cynodon dactylon* (L.) Pers.
544. *Linum sativum* L., *Linum usitatissimum* L., *Equisetum bogotense* Kunth, *Equisetum giganteum* L., *Phyllanthus niruri* L., *Phyllanthus stipulatus* (Raf.) G.L.Webster, *Phyllanthus urinaria* L., *Lycaste gigantea* Lindl., *Peumus boldus* Molina, *Cordia lutea* Lam
545. *Peumus boldus* Molina, *Desmodium molliculum (*Kunth*)* DC., *Linum sativum* L., *Linum usitatissimum* L., *Rorippa nasturtium-aquaticum* (L.) Hayek, *Dioscorea trifida* L. f., *Zea mays* L., *Cordia lutea* Lam

546. *Passiflora ligularis* Juss., *Peumus boldus* Molina, *Equisetum bogotense* Kunth, *Equisetum giganteum* L., *Mauria heterophylla* Kunth, *Bidens pilosa* L.

Inflammation of the Lungs

547. *Diplostephium gynoxyoides* Cuatrec., *Picrosia longifolia* D. Don

Inflammation of the Ovaries

548. *Iresine diffusa* Humb. & Bonpl. ex Willd., *Scabiosa atropurpurea* L., *Alternanthera porrigens* (Jacq.) Kuntze, *Cuphea strigulosa* Kunth, *Epilobium* sp., *Brosimum rubescens* Taub.
549. *Paranephelius uniflorus* Poepp., *Buddleja utilis* Kraenzl., *Bejaria aestuans* Mutis ex L., *Tiquilia paronychioides* (Phil.) A.T. Richardson, *Sambucus peruviana* Kunth, *Equisetum bogotense* Kunth, *Equisetum giganteum* L., *Desmodium molliculum (*Kunth*)* DC.
550. *Tiquilia paronychioides* (Phil.) A.T. Richardson, *Monactis flaverioides* Kunth, *Malva sylvestris* L., *Pelargonium odoratissimum* (L.) L'Hér., *Malva parviflora* L., *Zea mays* L., *Equisetum bogotense* Kunth, *Equisetum giganteum* L., *Pilea microphylla* (L.) Liebm., *Buddleja utilis* Kraenzl., *Bejaria aestuans* Mutis ex L., *Cenchrus echinatus* L., *Tribulus terrestris* L., *Baccharis genistelloides* (Lam.) Pers., *Bidens pilosa* L., *Rorippa nasturtium-aquaticum* (L.) Hayek, *Typha angustifolia* L., *Bixa orellana* L., *Alternanthera porrigens* (Jacq.) Kuntze, *Eustephia coccinea* Cav.
551. *Dioscorea tambillensis* R. Knuth, *Bidens pilosa* L., *Mauria heterophylla* Kunth, *Equisetum bogotense* Kunth, *Equisetum giganteum* L., *Desmodium molliculum (*Kunth*)* DC., *Verbena litoralis* Kunth, *Linum sativum* L., *Linum usitatissimum* L., *Hordeum vulgare* L.
552. *Bejaria aestuans* Mutis ex L., *Buddleja utilis* Kraenzl., *Dioscorea trifida* L. f., *Tiquilia paronychioides* (Phil.) A.T. Richardson, *Ilex guayusa* Loes., *Geranium ayavacense* Willd. ex Kunth, *Geranium sessiliflorum* Cav., *Monactis flaverioides* Kunth,, *Malva sylvestris* L., *Pelargonium odoratissimum* (L.) L'Hér., *Malva parviflora* L., *Bidens pilosa* L., *Verbena litoralis* Kunth, *Plantago linearis* Kunth, *Plantago major* L., *Equisetum bogotense* Kunth, *Equisetum giganteum* L., *Muehlenbeckia tamnifolia* (Kunth) Meisn., *Smilax medica* Schltdl. & Cham., *Oreocallis grandiflora* (Lam.) R. Br., *Cinchona officinalis* L.
553. *Caesalpinia spinosa* (Molina) Kuntze, *Nerium oleander* L., *Myrica pubescens* Humb. & Bonpl. ex Willd., *Monactis flaverioides* Kunth, *Artemisia absinthium* L., *Achyrocline alata* (Kunth) DC., *Aiouea dubia* (Kunth) Mez., *Nectandra reticulata* (Ruiz & Pav.) Mez.
554. *Desmodium molliculum* (Kunth) DC., *Mauria heterophylla* Kunth, *Bidens pilosa* L., *Verbena litoralis* Kunth
555. *Oreocallis grandiflora* (Lam.) R. Br., *Buddleja utilis* Kraenzl., *Tiquilia paronychioides* (Phil.) A.T. Richardson

Inflammation of the Stomach

556. *Iresine herbstii* Hook., *Pilea microphylla* (L.) Liebm.
557. *Tropaeolum minus* L., *Bidens pilosa* L., *Mauria heterophylla* Kunth, *Equisetum bogotense* Kunth, *Equisetum giganteum* L., *Verbena litoralis* Kunth, *Zea mays* L.

Inflammation of the Tonsils

558. *Caesalpinia spinosa* (Molina) Kuntze, *Rosmarinus officinalis* L., *Salvia rosmarinifolia* G. Don, *Erythroxylum coca* Lam, *Croton draconoides* Muell.-Arg., *Croton lechleri* Muell.-Arg.

Inflammation of the Womb

559. *Bejaria aestuans* Mutis ex L., *Buddleja utilis* Kraenzl., *Dioscorea trifida* L. f., *Tiquilia paronychioides* (Phil.) A.T. Richardson, *Ilex guayusa* Loes., *Geranium ayavacense* Willd. ex Kunth, *Geranium sessiliflorum* Cav., *Monactis flaverioides* Kunth, *Malva sylvestris* L., *Pelargonium odoratissimum* (L.) L'Hér., *Malva parviflora* L., *Bidens pilosa* L., *Verbena litoralis* Kunth, *Plantago linearis* Kunth, *Plantago major* L., *Equisetum bogotense* Kunth, *Equisetum giganteum* L., *Muehlenbeckia tamnifolia* (Kunth) Meisn., *Smilax medica* Schltdl. & Cham., *Oreocallis grandiflora* (Lam.) R. Br., *Cinchona officinalis* L.

Inflammation of urinary tract

560. *Schkuhria pinnata* (Lam.) Kuntze ex Thell., *Urtica magellanica* Juss. ex Poir., *Urtica urens* L., *Alternanthera porrigens* (Jacq.) Kuntze, *Cuphea strigulosa* Kunth, *Adiantum concinnum* Humb. & Bonpl. ex Willd., *Satureja pulchella* (Kunth) Briq., *Bejaria aestuans* Mutis ex L., *Peumus boldus* Molina, *Rorippa nasturtium-aquaticum* (L.) Hayek, *Buddleja utilis* Kraenzl., *Schkuhria pinnata* (Lam.) Kuntze ex Thell., *Polygala paniculata* L.
561. *Trifolium repens* L., *Alternanthera porrigens* (Jacq.) Kuntze, *Cuphea strigulosa* Kunth, *Iresine herbstii* Hook., *Smilax medica* Schltdl. & Cham.

Inflammation of Uterus

562. *Eustephia coccinea* Cav., *Buddleja utilis* Kraenzl. *Bejaria aestuans* Mutis ex L., *Pelargonium odoratissimum* (L.) L'Hér.
563. *Mauria heterophylla* Kunth, *Equisetum bogotense* Kunth, *Equisetum giganteum* L., *Verbena litoralis* Kunth, *Bidens pilosa* L.
564. *Paranephelius uniflorus* Poepp., *Buddleja utilis* Kraenzl., *Bejaria aestuans* Mutis ex L., *Tiquilia paronychioides* (Phil.) A.T. Richardson, *Desmodium molliculum (*Kunth*)* DC.), *Sambucus peruviana* Kunth, *Equisetum bogotense* Kunth, *Equisetum giganteum* L., *Desmodium molliculum (*Kunth*)* DC.
565. *Senecio chionogeton* Wedd., *Brugmansia arborea* (L.) Lagerh., *Brugmansia candida* Pers., *Gaultheria reticulata* Kunth, *Solanum mammosum* L.
566. *Bejaria aestuans* Mutis ex L., *Buddleja utilis* Kraenzl., *Dioscorea trifida* L. f., *Tiquilia paronychioides* (Phil.) A.T. Richardson, *Ilex guayusa* Loes., *Geranium ayavacense* Willd. ex Kunth, *Geranium sessiliflorum* Cav., *Monactis flaverioides* Kunth, *Malva sylvestris* L., *Pelargonium odoratissimum* (L.) L'Hér., *Malva parviflora* L., *Bidens pilosa* L., *Verbena litoralis* Kunth, *Plantago linearis* Kunth, *Plantago major* L., *Equisetum bogotense* Kunth, *Equisetum giganteum* L., *Muehlenbeckia tamnifolia* (Kunth) Meisn., *Smilax Smilax medica* Schltdl. & Cham., *Oreocallis grandiflora* (Lam.) R. Br., *Cinchona officinalis* L.

567. *Caesalpinia spinosa* (Molina) Kuntze, *Nerium oleander* L., *Myrica pubescens* Humb. & Bonpl. ex Willd., *Monactis flaverioides* Kunth, *Artemisia absinthium* L., *Achyrocline alata* (Kunth) DC., *Aiouea dubia* (Kunth) Mez., *Nectandra reticulata (*Ruiz & Pav.*) Mez*
568. *Oreocallis grandiflora* (Lam.) R. Br., *Buddleja utilis* Kraenzl., *Tiquilia paronychioides* (Phil.) A.T. Richardson
569. *Rubus robustus* C. Presl., *Alternanthera halimifolia* (Lam.) Standl. ex Pittier, *Bejaria aestuans* Mutis ex L., *Typha angustifolia* L.

Insomnia

570. *Apium graveolens* L., *Matricaria frigidum* (H.B.K.) Kunth, *Matricaria recutita* L., *Ambrosia peruviana* Willd., *Origanum majorana* L., *Adiantum concinnum* Humb. & Bonpl. ex Willd.
571. *Borago officinalis* L., *Oritrophium peruvianum* (Lam.) Cuatrec., *Senecio canescens* (Bonpl.) Cuatrec.
572. *Sambucus peruviana* Kunth, *Matricaria frigidum* (H.B.K.) Kunth, *Matricaria recutita* L., *Foeniculum vulgare* Mill., *Ambrosia peruviana* Willd., *Melissa officinalis* L., *Sanguisorba minor* Scop., *Dianthus caryophyllus* L., *Tagetes erecta* L.
573. *Dianthus caryophyllus* L., *Baccharis salicifolia* (Ruiz & Pav.) Pers., *Peperomia fraseri* C. DC., *Peperomia hartwegiana* Miq., *Hesperoxiphion niveum* (Ravenna) Ravenna, *Ruta graveolens L.*, *Rosmarinus officinalis* L., *Salvia rosmarinifolia* G. Don
574. *Melissa officinalis* L., *Sanguisorba minor* Scop., *Cymbopogon citratus* (DC.) Stapf., *Aloysia triphylla* (L'Hér.) Britton, *Origanum majorana* L., *Tillandsia cacticola* L.B. Sm., *Tillandsia multiflora* Benth. var. *decipiens* (André) L.B. Sm., *Echeveria peruviana* Meyen, *Salvia rosmarinifolia* G. Don, *Rosmarinus officinalis* L., *Dianthus caryophyllus* L., *Tagetes erecta* L., *Peperomia inaequalifolia* Ruiz & Pav., *Matricaria frigidum* (H.B.K.) Kunth, *Matricaria recutita* L., *Ambrosia peruviana* Willd. *Origanum majorana* L., *Sanguisorba minor* Scop., *Citrus sinensis* (L.) Osbeck
575. *Ocimum basilicum* L., *Eucalyptus globulus* Labill., *Schinus molle* L., *Spartium junceum* L., *Porophyllum ruderale* (Jacq.) Cass., *Ruta graveolens* L., *Ambrosia peruviana* Willd.,
576. *Ocimum basilicum* L., *Tilia platyphyllos* Scop., *Melissa officinalis* L., *Origanum majorana* L., *Cymbopogon citratus* (DC.) Stapf., *Aloysia triphylla* (L'Hér.) Britton
577. *Ocimum basilicum* L., *Rosmarinus officinalis* L., *Salvia rosmarinifolia* G. Don, *Lepechinia meyenii* (Walp.) Epling, *Cestrum auriculatum* L'Hér. *Monactis flaverioides* Kunth, *Malva sylvestris* L., *Pelargonium odoratissimum* (L.) L'Hér., *Malva parviflora* L.
578. *Oxalis bulbigera* R. Knuth, *Melissa officinalis* L., *Mentha x piperita* L., *Foeniculum vulgare* Mill., *Rosmarinus officinalis* L., *Salvia rosmarinifolia* G. Don
579. *Passiflora caerulea* L., *Melissa officinalis* L., *Sanguisorba minor* Scop., *Desmodium molliculum (*Kunth*)* DC., *Minthostachys mollis* (Kunth) Griseb., *Rosmarinus officinalis* L., *Salvia rosmarinifolia* G. Don
580. *Cydonia oblonga* Mill., *Matricaria frigidum* (H.B.K.) Kunth, *Matricaria recutita* L., *Ambrosia peruviana* Willd, *Melissa officinalis* L., *Foeniculum vulgare* Mill., *Lonicera japonica* Thunb. ex Murray, *Rosmarinus officinalis* L., *Salvia rosmarinifolia* G. Don
581. *Cydonia oblonga* Mill., *Matricaria frigidum* (H.B.K.) Kunth, *Matricaria recutita* L., *Ambrosia peruviana* Willd, *Melissa officinalis* L., *Sanguisorba minor* Scop., *Borago officinalis* L., *Viola tricolor* L., *Lathyrus odoratus* L., *Origanum majorana* L., *Rosmarinus officinalis* L., *Salvia rosmarinifolia* G. Don
582. *Fragaria vesca* L., *Foeniculum vulgare* Mill., *Matricaria frigidum* (H.B.K.) Kunth, *Matricaria recutita* L., *Ambrosia peruviana* Willd, *Sanguisorba minor* Scop.
583. *Sanguisorba minor* Scop., *Bejaria aestuans* Mutis ex L., *Cenchrus echinatus* L., *Tribulus terrestris* L., *Baccharis genistelloides* (Lam.) Pers., *Bidens pilosa* L., *Alternanthera porrigens* (Jacq.) Kuntze, *Cuphea strigulosa* Kunth *Melissa officinalis* L., *Peperomia inaequalifolia* Ruiz & Pav., *Dianthus caryophyllus* L., *Matricaria frigidum* (H.B.K.) Kunth, *Matricaria recutita* L., *Ambrosia peruviana* Willd, *Cestrum nocturnum* L.
584. *Citrus sinensis* (L.) Osbeck, *Melissa officinalis* L., *Dianthus caryophyllus* L., *Tagetes erecta* L., *Matricaria frigidum* (H.B.K.) Kunth, *Matricaria recutita* L., *Ambrosia peruviana* Willd, *Origanum majorana* L., *Desmodium molliculum (*Kunth*)* DC., *Minthostachys mollis* (Kunth) Griseb., *Rosmarinus officinalis* L., *Salvia rosmarinifolia* G. Don
585. *Tilia platyphyllos* Scop., *Sambucus peruviana* Kunth, *Matricaria frigidum* (H.B.K.) Kunth, *Matricaria recutita* L., *Ambrosia peruviana* Willd, *Foeniculum vulgare* Mill., *Melissa officinalis* L., *Sanguisorba minor* Scop., *Dianthus caryophyllus* L., *Tagetes erecta* L.
586. *Aloysia triphylla* (L'Hér.) Britton, *Desmodium molliculum (*Kunth*)* DC., *Minthostachys mollis* (Kunth) Griseb., *Marrubium vulgare* L., *Melissa officinalis* L., *Foeniculum vulgare* Mill., *Lonicera japonica* Thunb. ex Murray, *Dianthus caryophyllus* L., *Tagetes erecta* L., *Viola tricolor* L., *Lathyrus odoratus* L.
587. *Viola tricolor* L., *Melissa officinalis* L.

Internal Bleeding

588. *Acmella* cf. *ciliata* (Kunth) Cass., *Mentha spicata* L.

Intestine

589. *Arctium lappa* L., *Bejaria aestuans* Mutis ex L., *Cenchrus echinatus* L., *Tribulus terrestris* L., *Baccharis genistelloides* (Lam.) Pers., *Bidens pilosa* L., *Centropogon* cf. *rufus* E. Wimm.
590. *Malva sylvestris* L., *Chuquiraga weberbaueri* Tovar, *Picrosia longifolia* D. Don.
591. *Cenchrus echinatus* L., *Bidens pilosa* L., *Arctium lappa* L., *Centropogon* cf. *rufus* E. Wimm

Judgment

592. *Clethra castaneifolia* Meisn., *Oenothera rosea* L'Hér. ex Aiton, *Cheilanthes myriophylla* Desv., *Hesperoxiphion niveum* (Ravenna) Ravenna

Kidneys

593. *Iresine diffusa* Humb. & Bonpl. ex Willd., *Scabiosa atropurpurea* L., *Alternanthera porrigens* (Jacq.) Kuntze, *Cuphea strigulosa* Kunth, *Epilobium* sp., *Brosimum rubescens* Taub.
594. *Iresine herbstii* Hook., *Pilea microphylla* (L.) Liebm.
595. *Mauria heterophylla* Kunth, *Equisetum bogotense* Kunth, *Equisetum giganteum* L., *Verbena litoralis* Kunth, *Bidens pilosa* L.

596. *Annona muricata* L., *Bidens pilosa* L., *Ananas comosus* (L.) Merr., *Bixa orellana* L.
597. *Baccharis genistelloides* (Lam.) Pers., *Schkuhria pinnata* (Lam.) Kuntze ex Thell., *Polygala paniculata* L., *Verbena litoralis* Kunth, *Bidens pilosa* L., *Equisetum bogotense* Kunth, *Equisetum giganteum* L., *Cuphea strigulosa* Kunth, *Ipomoea batatas* (L.) Lam.
598. *Bidens pilosa* L., *Mauria heterophylla* Kunth, *Eugenia obtusifolia* Cambess., *Tiquilia paronychioides* (Phil.) A.T. Richardson, *Zea mays* L., *Equisetum bogotense* Kunth, *Equisetum giganteum* L., *Sanguisorba minor* Scop., *Cestrum nocturnum* L.
599. *Tessaria integrifolia* Ruiz & Pav., *Equisetum bogotense* Kunth, *Equisetum giganteum* L., *Verbena litoralis* Kunth, *Mauria heterophylla* Kunth, *Iresine diffusa* Humb. & Bonpl. ex Willd., *Plantago sericea* Ruiz & Pav. subsp. *sericans* (Pilg.) Rahn, *Zea mays* L.
600. *Capsella bursa-pastoris* (L.) Medik., *Mauria heterophylla* Kunth, *Verbena litoralis* Kunth, *Zea mays* L., *Buddleja utilis* Kraenzl., *Tiquilia paronychioides* (Phil.) A.T. Richardson, *Geranium ayavacense* Willd. ex Kunth, *Geranium sessiliflorum* Cav., *Gentianella bicolor* (Wedd.) J.S. Pringle, *Equisetum bogotense* Kunth, *Equisetum giganteum* L.
601. *Rorippa nasturtium-aquaticum* (L.) Hayek, *Monactis flaverioides* Kunth, *Malva sylvestris* L., *Pelargonium odoratissimum* (L.) L'Hér., *Malva parviflora* L., *Bidens pilosa* L., *Desmodium molliculum (*Kunth*)* DC., *Eugenia obtusifolia* Cambess., *Mauria heterophylla* Kunth, *Iresine diffusa* Humb. & Bonpl. ex Willd., *Plantago sericea* Ruiz & Pav. subsp. *sericans* (Pilg.) Rahn, *Tiquilia paronychioides* (Phil.) A.T. Richardson, *Bejaria aestuans* Mutis ex L.
602. *Equisetum bogotense* Kunth, *Cordia lutea* Lam, *Alternanthera porrigens* (Jacq.) Kuntze, *Cuphea strigulosa* Kunth, *Typha angustifolia* L., *Bixa orellana* L., *Smilax medica* Schltdl. & Cham.
603. *Equisetum bogotense* Kunth, *Desmodium molliculum (*Kunth*)* DC., *Mauria heterophylla* Kunth
604. *Equisetum giganteum* L., *Verbena litoralis* Kunth, *Matricaria frigidum* (H.B.K.) Kunth, *Matricaria recutita* L., *Ambrosia peruviana* Willd, *Mauria heterophylla* Kunth, *Eugenia obtusifolia* Cambess., *Zea mays* L., *Iresine diffusa* Humb. & Bonpl. ex Willd., *Plantago sericea* Ruiz & Pav. subsp. *sericans* (Pilg.) Rahn, *Rorippa nasturtium-aquaticum* (L.) Hayek, *Desmodium molliculum (*Kunth*)* DC., *Dioscorea trifida* L. f.
605. *Phyllanthus stipulatus* (Raf.) G.L.Webster, *Phyllanthus niruri* L., *Equisetum bogotense* Kunth, *Equisetum giganteum* L., *Plantago linearis* Kunth, *Plantago major* L., *Peumus boldus* Molina, *Cordia lutea* Lam, *Lycaste gigantea* Lindl., *Buddleja utilis* Kraenzl., *Tiquilia paronychioides* (Phil.) A.T. Richardson
606. *Desmodium molliculum* (Kunth) DC., *Mauria heterophylla* Kunth, *Bidens pilosa* L., *Verbena litoralis* Kunth
607. *Trifolium repens* L., *Iresine herbstii* Hook., *Smilax medica* Schltdl. & Cham.
608. *Geranium sessiliflorum* Cav., *Geranium ayavacense* Willd. ex Kunth, *Mauria heterophylla* Kunth, *Equisetum bogotense* Kunth, *Equisetum giganteum* L., *Verbena litoralis* Kunth, *Eugenia obtusifolia* Cambess., *Bidens pilosa* L., *Cynodon dactylon* (L.) Pers.
609. *Persea americana* Mill., *Linum sativum* L., *Linum usitatissimum* L.
610. *Linum sativum* L., *Linum usitatissimum* L., *Equisetum bogotense* Kunth, *Equisetum giganteum* L., *Phyllanthus stipulatus* (Raf.) G.L.Webster, *Phyllanthus niruri* L., *Phyllanthus urinaria* L., *Lycaste gigantea* Lindl., *Peumus boldus* Molina, *Cordia lutea* Lam
611. *Cuphea strigulosa* Kunth, *Peperomia inaequalifolia* Ruiz & Pav., *Dianthus caryophyllus* L., *Tagetes erecta* L., *Lonicera japonica* Thunb. ex Murray, *Urtica magellanica* Juss. ex Poir., *Urtica urens* L., *Alternanthera porrigens* (Jacq.) Kuntze, *Alternanthera halimifolia* (Lam.) Standl. ex Pittier, *Pilea microphylla* (L.) Liebm., *Iresine herbstii* Hook., *Erodium cicutarium* (L.) L'Hér. ex Aiton, *Desmodium molliculum (*Kunth*)* DC., *Equisetum bogotense* Kunth, *Equisetum giganteum* L., *Verbena litoralis* Kunth, *Sanguisorba minor* Scop., *Buddleja utilis* Kraenzl., *Cynodon dactylon* (L.) Pers., *Bejaria aestuans* Mutis ex L., *Cenchrus echinatus* L., *Tribulus terrestris* L., *Baccharis genistelloides* (Lam.) Pers., *Bidens pilosa* L.
612. *Mirabilis jalapa* L., *Tiquilia paronychioides* (Phil.) A.T. Richardson
613. *Polypodium crassifolium* L., *Desmodium molliculum (*Kunth*)* DC., *Bidens pilosa* L., *Equisetum bogotense* Kunth, *Equisetum giganteum* L.
614. *Portulaca oleracea* L. subsp. *tuberculata* Danin & H.G. Baker, *Peumus boldus* Molina, *Tiquilia paronychioides* (Phil.) A.T. Richardson, *Equisetum bogotense* Kunth, *Equisetum giganteum* L.
615. *Rubus robustus* C. Presl., *Alternanthera halimifolia* (Lam.) Standl. ex Pittier, *Bejaria aestuans* Mutis ex L., *Typha angustifolia* L.
616. *Uncaria tomentosa* (Willd. ex Roem. & Schult.) DC., *Phyllanthus stipulatus* (Raf.) G.L.Webster, *Phyllanthus niruri* L., *Phyllanthus urinaria* L., *Linum sativum* L., *Linum usitatissimum* L., *Peumus boldus* Molina, *Cordia lutea* Lam, *Capsella bursa-pastoris* (L.) Medik.

Laxative

617. *Inga edulis* Mart., *Acanthoxanthium spinosum* (L.) Fourr., *Senna bicapsularis* (L.) Roxb.
618. *Huperzia* cf. *columnaris* B. Øllg., *Echinopsis pachanoi* (Britton & Rose) Friedrich & G.D. Rowley

Liver

619. *Iresine herbstii* Hook., *Pilea microphylla* (L.) Liebm.
620. *Cocos nucifera* L., *Cymbopogon citratus* (DC.) Stapf., *Otholobium glandulosum* (L.) J.W. Grimes, *Foeniculum vulgare* Mill., *Mentha x piperita* L.
621. *Arctium lappa* L., *Bejaria aestuans* Mutis ex L., *Cenchrus echinatus* L., *Tribulus terrestris* L., *Baccharis genistelloides* (Lam.) Pers., *Bidens pilosa* L., *Centropogon* cf. *rufus* E. Wimm.
622. *Baccharis genistelloides* (Lam.) Pers., *Schkuhria pinnata* (Lam.) Kuntze ex Thell., *Polygala paniculata* L., *Verbena litoralis* Kunth, *Bidens pilosa* L., *Equisetum bogotense* Kunth, *Equisetum giganteum* L., *Cuphea strigulosa* Kunth, *Ipomoea batatas* (L.) Lam.
623. *Bidens pilosa* L., *Mauria heterophylla* Kunth, *Eugenia obtusifolia* Cambess., *Tiquilia paronychioides* (Phil.) A.T. Richardson, *Zea mays* L., *Equisetum bogotense* Kunth, *Equisetum giganteum* L., *Psidium guajava* L., *Sanguisorba minor* Scop., *Cestrum nocturnum* L.
624. *Chuquiraga weberbaueri* Tovar, *Eucalyptus globulus* Labill., *Piper aduncum* L., *Gaultheria erecta* Vent., *Desmodium molliculum (*Kunth*)* DC., *Minthostachys mollis* (Kunth) Griseb., *Cordia lutea* Lam
625. *Schkuhria pinnata* (Lam.) Kuntze ex Thell., *Urtica magellanica* Juss. ex Poir., *Urtica urens* L., *Alternanthera porrigens* (Jacq.) Kuntze, *Cuphea strigulosa* Kunth, *Adiantum concinnum* Humb. & Bonpl. ex Willd., *Satureja pulchella* (Kunth) Briq., *Bejaria aestuans* Mutis ex L., *Peumus boldus* Molina, *Rorippa nasturtium-aquaticum* (L.) Hayek, *Buddleja utilis* Kraenzl., *Schkuhria pinnata* (Lam.) Kuntze ex Thell., *Polygala paniculata* L.

626. *Taraxacum officinale* F.H. Wigg., *Mauria heterophylla* Kunth, *Desmodium molliculum (*Kunth*)* DC., *Equisetum bogotense* Kunth, *Equisetum giganteum* L., *Linum sativum* L., *Linum usitatissimum* L., *Monactis flaverioides* Kunth, *Malva sylvestris* L., *Pelargonium odoratissimum* (L.) L'Hér., *Malva parviflora* L., *Bidens pilosa* L., *Aristolochia ruiziana* (Klotzsch) Duch.
627. *Tessaria integrifolia* Ruiz & Pav., *Equisetum bogotense* Kunth, *Equisetum giganteum* L., *Verbena litoralis* Kunth, *Mauria heterophylla* Kunth, *Iresine diffusa* Humb. & Bonpl. ex Willd., *Plantago sericea* Ruiz & Pav. subsp. *sericans* (Pilg.) Rahn, *Zea mays* L.
628. *Berberis buceronis* J.F. Macbr., *Bidens pilosa* L., *Equisetum bogotense* Kunth, *Equisetum giganteum* L.
629. *Cordia lutea* Lam, *Plantago linearis* Kunth, *Plantago major* L., *Peumus boldus* Molina,
630. *Iresine diffusa* Humb. & Bonpl. ex Willd., *Scabiosa atropurpurea* L., *Alternanthera porrigens* (Jacq.) Kuntze, *Cuphea strigulosa* Kunth, *Epilobium* sp., *Brosimum rubescens* Taub.
631. *Mauria heterophylla* Kunth, *Equisetum bogotense* Kunth, *Equisetum giganteum* L., *Verbena litoralis* Kunth, *Bidens pilosa* L.
632. *Picrosia longifolia* D. Don, *Verbena litoralis* Kunth, *Schkuhria pinnata* (Lam.) Kuntze ex Thell., *Polygala paniculata* L.
633. *Rorippa nasturtium-aquaticum* (L.) Hayek, *Monactis flaverioides* Kunth, *Malva sylvestris* L., *Pelargonium odoratissimum* (L.) L'Hér., *Malva parviflora* L., *Desmodium molliculum (*Kunth*)* DC., *Eugenia obtusifolia* Cambess., *Bidens pilosa* L., *Mauria heterophylla* Kunth, *Iresine diffusa* Humb. & Bonpl. ex Willd., *Plantago sericea* Ruiz & Pav. subsp. *sericans* (Pilg.) Rahn, *Tiquilia paronychioides* (Phil.) A.T. Richardson, *Bejaria aestuans* Mutis ex L.,
634. *Centropogon* cf. *rufus* E. Wimm., *Bejaria aestuans* Mutis ex L., *Cenchrus echinatus* L., *Tribulus terrestris* L., *Baccharis genistelloides* (Lam.) Pers., *Bidens pilosa* L., *Arctium lappa* L.
635. *Dioscorea tambillensis* R. Knuth, *Bidens pilosa* L., *Mauria heterophylla* Kunth, *Equisetum bogotense* Kunth, *Equisetum giganteum* L., *Desmodium molliculum (*Kunth*)* DC., *Verbena litoralis* Kunth, *Linum sativum* L., *Linum usitatissimum* L., *Hordeum vulgare* L.
636. *Bejaria aestuans* Mutis ex L., *Buddleja utilis* Kraenzl., *Dioscorea trifida* L. f., *Tiquilia paronychioides* (Phil.) A.T. Richardson, *Ilex guayusa* Loes., *Geranium ayavacense* Willd. ex Kunth, *Geranium sessiliflorum* Cav., *Monactis flaverioides* Kunth, *Malva sylvestris* L., *Pelargonium odoratissimum* (L.) L'Hér., *Malva parviflora* L., *Bidens pilosa* L., *Verbena litoralis* Kunth, *Plantago linearis* Kunth, *Plantago major* L., *Equisetum bogotense* Kunth, *Equisetum giganteum* L., *Muehlenbeckia tamnifolia* (Kunth) Meisn., *Smilax medica* Schltdl. & Cham., *Oreocallis grandiflora* (Lam.) R. Br., *Cinchona officinalis* L.
637. *Phyllanthus stipulatus* (Raf.) G.L.Webster, *Phyllanthus niruri* L., *Equisetum bogotense* Kunth, *Equisetum giganteum* L., *Plantago linearis* Kunth, *Plantago major* L., *Peumus boldus* Molina, *Cordia lutea* Lam, *Lycaste gigantea* Lindl., *Buddleja utilis* Kraenzl., *Tiquilia paronychioides* (Phil.) A.T. Richardson
638. *Spartium junceum* L., *Cordia lutea* Lam
639. *Geranium sessiliflorum* Cav., *Mauria heterophylla* Kunth, *Equisetum bogotense* Kunth, *Equisetum giganteum* L., *Verbena litoralis* Kunth, *Eugenia obtusifolia* Cambess., *Bidens pilosa* L., *Cynodon dactylon* (L.) Pers.
640. *Satureja pulchella* (Kunth) Briq., *Otholobium glandulosum* (L.) J.W. Grimes, *Matricaria frigidum* (H.B.K.) Kunth, *Matricaria recutita* L., *Ambrosia peruviana* Willd., *Desmodium molliculum (*Kunth*)* DC., *Minthostachys mollis* (Kunth) Griseb.
641. *Malva parviflora* L., *Desmodium molliculum (*Kunth*)* DC., *Mauria heterophylla* Kunth, *Verbena litoralis* Kunth, *Equisetum bogotense* Kunth, *Equisetum giganteum* L., *Bidens pilosa* L., *Monactis flaverioides* Kunth, *Malva sylvestris* L., *Pelargonium odoratissimum* (L.) L'Hér.
642. *Passiflora ligularis* Juss., *Peumus boldus* Molina, *Equisetum bogotense* Kunth, *Equisetum giganteum* L., *Mauria heterophylla* Kunth, *Bidens pilosa* L.
643. *Piper* cf. *aequale* Vahl, *Cordia lutea* Lam, *Peumus boldus* Molina
644. *Plantago linearis* Kunth, *Equisetum bogotense* Kunth, *Equisetum giganteum* L., *Mauria heterophylla* Kunth, *Eugenia obtusifolia* Cambess., *Cynodon dactylon* (L.) Pers., *Buddleja utilis* Kraenzl.
645. *Cenchrus echinatus* L., *Bidens pilosa* L., *Arctium lappa* L., *Centropogon* cf. *rufus* E. Wimm.
646. *Polypodium crassifolium* L., *Desmodium molliculum (*Kunth*)* DC., *Bidens pilosa* L., *Equisetum bogotense* Kunth, *Equisetum giganteum* L.
647. *Portulaca oleracea* L. subsp. *tuberculata* Danin & H.G. Baker, *Peumus boldus* Molina, *Tiquilia paronychioides* (Phil.) A.T. Richardson, *Equisetum bogotense* Kunth, *Equisetum giganteum* L.
648. *Tribulus terrestris* L., *Bidens pilosa* L., *Arctium lappa* L., *Centropogon* cf. *rufus* E. Wimm.

Luck

649. *Tillandsia cacticola* L.B. Sm., *Rosmarinus officinalis* L., *Salvia rosmarinifolia* G. Don, *Bursera graveolens* (Kunth) Triana & Planch., *Lavandula angustifolia* Mill.
650. *Dianthus caryophyllus* L., *Baccharis salicifolia* (Ruiz & Pav.) Pers., *Peperomia fraseri* C. DC., (Rav.) Rav., *Ruta graveolens* L., *Rosmarinus officinalis* L., *Salvia rosmarinifolia* G. Don
651. *Gentianella bicolor* (Wedd.) J.S. Pringle, *Baccharis salicifolia* (Ruiz & Pav.) Pers., *Peperomia fraseri* C. DC., *Peperomia hartwegiana* Miq., *Hypericum laricifolium* Juss., *Hesperoxiphion niveum* (Ravenna) Ravenna, *Werneria villosa* A. Gray, *Stelis eublepharis* Rchb. f., *Jamesonia goudotii* (Hieron.) C. Chr., *Tetragonia crystallina* L'Hér.
652. *Ocimum basilicum* L., *Eucalyptus globulus* Labill., *Schinus molle* L., *Spartium junceum* L., *Porophyllum ruderale* (Jacq.) Cass., *Ruta graveolens* L., *Ambrosia peruviana* Willd.,
653. *Ocimum basilicum* L., *Tilia platyphyllos* Scop., *Melissa officinalis* L., *Origanum majorana* L., *Cymbopogon citratus* (DC.) Stapf., *Aloysia triphylla* (L'Hér.) Britton
654. *Ocimum basilicum* L., *Rosmarinus officinalis* L., *Salvia rosmarinifolia* G. Don, *Lepechinia meyenii* (Walp.) Epling, *Cestrum auriculatum* L'Hér., *Monactis flaverioides* Kunth, *Malva sylvestris* L., *Pelargonium odoratissimum* (L.) L'Hér., *Malva parviflora* L.
655. *Stelis eublepharis* Rchb. f., *Baccharis salicifolia* (Ruiz & Pav.) Pers., *Peperomia fraseri* C. DC., *Peperomia hartwegiana* Miq., *Hesperoxiphion niveum* (Ravenna) Ravenna, *Oenothera rosea* L'Hér. ex Aiton, *Cheilanthes myriophylla* Desv., *Baccharis vaccinioides* Kunth
656. *Peperomia fraseri* C. DC., *Valeriana plantaginea* Kunth, *Huperzia* cf. *columnaris* B. Øllg., *Phyllactis rigida* (Ruiz & Pav.) Pers., *Gentianella bicolor* (Wedd.) J.S. Pringle, *Senecio chionogeton* Wedd., *Solanum* sp., *Niphogeton dissecta* (Benth.) J.F. Macbr., *Huperzia kuesteri* (Nessel) B. Øllg., *Werneria villosa* A. Gray, *Stelis eublepharis* Rchb. f., *Hypericum laricifolium* Juss.

657. *Peperomia quadrifolia* (L.) Kunth, *Bejaria aestuans* Mutis ex L., *Loricaria ferruginea* (Ruiz & Pav.) Wedd., *Lycopodium clavatum* L., *Alternanthera porrigens* (Jacq.) Kuntze, *Oreobolus goeppingeri* Suess., *Jamesonia goudotii* (Hieron.) C. Chr., *Desmodium triflorum* (L.) DC., *Boerhavia coccinea* Mill., *Tillandsia cacticola* L.B. Sm., *Tillandsia multiflora* Benth. var. *decipiens* (André) L.B. Sm., *Echeveria peruviana* Meyen, *Hypericum laricifolium* Juss., *Peperomia hartwegiana* Miq., *Baccharis salicifolia* (Ruiz & Pav.) Pers., *Peperomia fraseri* C. DC., *Hypericum laricifolium* Juss., *Pachyphyllum pastii* Kraenzl. ex Weberb., *Tetragonia crystallina* L'Hér., *Xyris subulata* Ruiz & Pav., *Hesperoxiphion niveum* (Ravenna) Ravenna
658. *Peperomia quadrifolia* (L.) Kunth, *Hypericum laricifolium* Juss., *Peperomia fraseri* C. DC., *Baccharis salicifolia* (Ruiz & Pav.) Pers., *Peperomia hartwegiana* Miq., *Peperomia fraseri* C. DC., *Xyris subulata* Ruiz & Pav., *Werneria pygmaea* Gillies ex Hook. & Arn.
659. *Citrus limon* (L.) Burm. f., *Equisetum bogotense* Kunth, *Equisetum giganteum* L., *Desmodium molliculum (*Kunth*)* DC., *Mauria heterophylla* Kunth, *Bidens pilosa* L., *Verbena litoralis* Kunth
660. *Ruta graveolens* L., *Gentianella dianthoides* (Kunth) Fabris ex J.S. Pringle, *Gentianella bicolor* (Wedd.) J.S. Pringle, *Salvia officinalis* L., *Salvia cuspidata* Ruiz & Pav., *Salvia sagittata* Ruiz. & Pav., *Lepechinia meyenii* (Walp.) Epling, *Origanum vulgare* L., *Schinus molle* L., *Eucalyptus globulus* Labill., *Ambrosia peruviana* Willd., *Artemisia absinthium* L., *Adiantum concinnum* Humb. & Bonpl. ex Willd.
661. *Jaltomata* sp., *Diplostephium sagasteguii* Cuatrec., *Alternanthera brasiliana* (L.) Kuntze, *Epidendrum calanthum* Rchb. f. & Warsz., *Solanum mammosum* L., *Brugmannsia* sp.
662. *Urtica urens* L., *Artemisia absinthium* L., *Salvia discolor* Kunth, *Ambrosia peruviana* Willd., *Miconia salicifolia* (Bonpl. ex Naudin) Naudin
663. *Belonanthus* aff. *hispidus* (Wedd.) Graebn., *Hesperoxiphion niveum* (Ravenna) Ravenna, *Werneria pygmaea* Gillies ex Hook. & Arn., *Peperomia fraseri* C. DC., *Baccharis salicifolia* (Ruiz & Pav.) Pers., *Peperomia hartwegiana* Miq., *Hypericum laricifolium* Juss., *Baccharis vaccinioides* Kunth

Lungs

664. *Borago officinalis* L., *Oritrophium peruvianum* (Lam.) Cuatrec., *Senecio canescens* (Bonpl.) Cuatrec.

Mal Aire

665. *Daucus montanus* Humb. & Bonpl. ex Spreng., *Achyrocline alata* (Kunth) DC., *Siphocampylus cutervensis* Zahlbr., *Alternanthera brasiliana* (L.) Kuntze, *Phytolacca bogotensis* Kunth
666. *Diplostephium gynoxyoides* Cuatrec., *Tagetes erecta* L., *Tagetes patula* L., *Dolichos lablab* L., *Allium sativum* L.
667. *Diplostephium sagasteguii* Cuatrec., *Alternanthera brasiliana* (L.) Kuntze, *Epidendrum calanthum* Rchb. f. & Warsz., *Solanum mammosum* L., *Brugmansia candida* Pers.
668. *Monactis flaverioides* Kunth, *Artemisia absinthium* L., *Ambrosia peruviana* Willd., *Miconia salicifolia* (Bonpl. ex Naudin) Naudin
669. *Munnozia lyrata* (A. Gray) H. Rob. & Brettell, *Monactis flaverioides* Kunth, *Siparuna muricata* (Ruiz & Pav.) A. DC., *Trixis cacalioides* (Kunth) D. Don, *Salvia tubiflora* Ruiz & Pav., *Achyrocline alata* (Kunth) DC., *Aiouea dubia* (Kunth) Mez., *Nectandra reticulata* (Ruiz & Pav.) Mez.
670. *Pseudogynoxys cordifolia* (Cass.) Cabrera, *Marrubium vulgare* L., *Rosmarinus officinalis* L., *Salvia rosmarinifolia* G. Don
671. *Senecio chionogeton* Wedd., *Gentianella bicolor* (Wedd.) J.S. Pringle, *Echinopsis pachanoi* (Britton & Rose) Friedrich & G.D. Rowley
672. *Senecio chionogeton* Wedd., *Brugmansia arborea* (L.) Lagerh., *Brugmansia candida* Pers., *Gaultheria reticulata* Kunth, *Solanum mammosum* L.
673. *Gaultheria reticulata* Kunth, *Brugmansia arborea* (L.) Lagerh., *Brugmansia sanguinea* (Ruiz & Pav.) D. Don, *Brugmansia sanguinea* (Ruiz & Pav.) D. Don, *Brugmansia candida* Pers.,
674. *Cuphea strigulosa* Kunth, *Peperomia inaequalifolia* Ruiz & Pav., *Dianthus caryophyllus* L., *Lonicera japonica* Thunb. ex Murray, *Urtica magellanica* Juss. ex Poir., *Urtica urens* L., *Alternanthera halimifolia* (Lam.) Standl. ex Pittier, *Alternanthera porrigens* (Jacq.) Kuntze, *Pilea microphylla* (L.) Liebm., *Iresine herbstii* Hook., *Erodium cicutarium* (L.) L'Hér. ex Aiton, *Desmodium molliculum (*Kunth*)* DC., *Equisetum bogotense* Kunth, *Equisetum giganteum* L., *Verbena litoralis* Kunth, *Sanguisorba minor* Scop., *Buddleja utilis* Kraenzl., *Cynodon dactylon* (L.) Pers., *Bejaria aestuans* Mutis ex L., *Cenchrus echinatus* L., *Tribulus terrestris* L., *Baccharis genistelloides* (Lam.) Pers., *Bidens pilosa* L.
675. *Digitaria ciliaris* (Retz.) Koeler, *Salvia tubiflora* Ruiz & Pav. *Achyrocline alata* (Kunth) DC., *Aiouea dubia* (Kunth) Mez., *Nectandra reticulata* (Ruiz & Pav.) Mez., *Strychnos* sp., *Oenothera rosea* L'Hér. ex Aiton, *Cheilanthes myriophylla* Desv.
676. *Jaltomata* sp., *Diplostephium sagasteguii* Cuatrec., *Alternanthera brasiliana* (L.) Kuntze, *Epidendrum calanthum* Rchb. f. & Warsz., *Solanum mammosum* L.

Malaria

677. *Phytolacca bogotensis* Kunth, *Siparuna muricata* (Ruiz & Pav.) A. DC., *Trixis cacalioides* (Kunth) D. Don, *Artemisia absinthium* L., *Sambucus peruviana* Kunth, *Mauria heterophylla* Kunth, *Equisetum giganteum* L.
678. *Salix chilensis* Molina, *Prunus serotina* Ehrh. subsp. *capuli* (Cav.) McVaugh

Menstrual regulation

679. *Adiantum concinnum* Humb. & Bonpl. ex Willd., *Bejaria aestuans* Mutis ex L., *Alternanthera porrigens* (Jacq.) Kuntze, *Cuphea strigulosa* Kunth, *Alternanthera halimifolia* (Lam.) Standl. ex Pittier, *Alternanthera brasiliana* (L.) Kuntze, *Origanum vulgare* L.
680. *Petroselinum crispum* (Mill.) Fuss, *Melissa officinalis* L., *Sanguisorba minor* Scop., *Origanum majorana* L., *Tillandsia cacticola* L.B. Sm., *Tillandsia multiflora* Benth. var. *decipiens* (André) L.B. Sm., *Echeveria peruviana* Meyen
681. *Schkuhria pinnata* (Lam.) Kuntze ex Thell., *Urtica magellanica* Juss. ex Poir., *Urtica urens* L., *Alternanthera porrigens* (Jacq.) Kuntze, *Cuphea strigulosa* Kunth, *Adiantum concinnum* Humb. & Bonpl. ex Willd., *Satureja pulchella* (Kunth) Briq., *Bejaria aestuans* Mutis ex L., *Peumus boldus* Molina, *Rorippa nasturtium-aquaticum* (L.) Hayek, *Buddleja utilis* Kraenzl., *Polygala paniculata* L.
682. *Equisetum giganteum* L., *Verbena litoralis* Kunth, *Matricaria frigidum* (H.B.K.) Kunth, *Matricaria recutita* L., *Ambrosia peruviana* Willd., *Mauria heterophylla* Kunth, *Eugenia obtusifolia* Cambess., *Zea mays* L., *Iresine diffusa* Humb. & Bonpl. ex

Willd., *Plantago sericea* Ruiz & Pav. subsp. *sericans* (Pilg.) Rahn, *Rorippa nasturtium-aquaticum* (L.) Hayek, *Desmodium molliculum (*Kunth*)* DC., *Dioscorea trifida* L. f.

683. *Bejaria aestuans* Mutis ex L., *Buddleja utilis* Kraenzl., *Dioscorea trifida* L. f., *Tiquilia paronychioides* (Phil.) A.T. Richardson, *Ilex guayusa* Loes., *Geranium ayavacense* Willd. ex Kunth, *Geranium sessiliflorum* Cav., *Monactis flaverioides* Kunth, *Alcea rosea* L., *Pelargonium odoratissimum* (L.) L'Hér., *Malva parviflora* L., *Bidens pilosa* L., *Verbena litoralis* Kunth, *Plantago linearis* Kunth, *Plantago major* L., *Equisetum bogotense* Kunth, *Equisetum giganteum* L., *Muehlenbeckia tamnifolia* (Kunth) Meisn., *Smilax medica* Schltdl. & Cham., *Oreocallis grandiflora* (Lam.) R. Br., *Cinchona officinalis* L.
684. *Origanum majorana* L., *Alternanthera halimifolia* (Lam.) Standl. ex Pittier, *Peperomia inaequalifolia* Ruiz & Pav., *Melissa officinalis* L., *Matricaria frigidum* (H.B.K.) Kunth, *Matricaria recutita* L., *Ambrosia peruviana* Willd., *Foeniculum vulgare* Mill., *Hyptis sidifolia* (L'Hér.) Briq., *Mentha x piperita* L.
685. *Salvia officinalis* L., *Piper aduncum* L., *Juglans neotropica* Diels, *Eucalyptus globulus* Labill.
686. *Satureja pulchella* (Kunth) Briq., *Otholobium glandulosum* (L.) J.W. Grimes, *Matricaria frigidum* (H.B.K.) Kunth, *Matricaria recutita* L., *Ambrosia peruviana* Willd., *Desmodium molliculum (*Kunth*)* DC., *Minthostachys mollis* (Kunth) Griseb.
687. *Ximenia americana* L., *Citrus limon* (L.) Burm. f., *Satureja pulchella* (Kunth) Briq., *Foeniculum vulgare* Mill., *Ambrosia peruviana* Willd., *Melissa officinalis* L., *Sanguisorba minor* Scop.
688. *Ruta graveolens* L., *Gentianella dianthoides* (Kunth) Fabris ex J.S. Pringle, *Gentianella bicolor* (Wedd.) J.S. Pringle, *Lepechinia meyenii* (Walp.) Epling, *Salvia officinalis* L., *Salvia cuspidata* Ruiz & Pav., *Salvia sagittata* Ruiz. & Pav., *Origanum vulgare* L., *Schinus molle* L., *Eucalyptus globulus* Labill., *Ambrosia peruviana* Willd., *Artemisia absinthium* L., *Adiantum concinnum* Humb. & Bonpl. ex Willd.
689. *Cestrum undulatum* Ruiz & Pav., *Cestrum strigilatum* Ruiz & Pav., *Ruta graveolens* L., *Origanum vulgare* L.
690. *Iresine diffusa* Humb. & Bonpl. ex Willd., *Scabiosa atropurpurea* L., *Alternanthera porrigens* (Jacq.) Kuntze, *Cuphea strigulosa* Kunth, *Epilobium* sp., *Brosimum rubescens* Taub.
691. *Lantana scabiosiflora* Kunth, *Schkuhria pinnata* (Lam.) Kuntze ex Thell., *Polygala paniculata* L., *Adiantum concinnum* Humb. & Bonpl. ex Willd., *Bejaria aestuans* Mutis ex L., *Satureja pulchella* (Kunth) Briq., *Lepechinia meyenii* (Walp.) Epling

Nausea

692. *Ruta graveolens* L., *Gentianella dianthoides* (Kunth) Fabris ex J.S. Pringle, *Gentianella bicolor* (Wedd.) J.S. Pringle, *Lepechinia meyenii* (Walp.) Epling, *Salvia officinalis* L., *Salvia cuspidata* Ruiz & Pav, *Salvia sagittata* Ruiz. & Pav., *Origanum vulgare* L., *Schinus molle* L., *Eucalyptus globulus* Labill., *Ambrosia peruviana* Willd., *Artemisia absinthium* L., *Adiantum concinnum* Humb. & Bonpl. ex Willd.

Nerves

693. *Alternanthera halimifolia* (Lam.) Standl. ex Pittier, *Melissa officinalis* L., *Matricaria frigidum* (H.B.K.) Kunth, *Matricaria recutita* L., *Ambrosia peruviana* Willd., *Rosmarinus officinalis* L., *Salvia rosmarinifolia* G. Don, *Foeniculum vulgare* Mill., *Marrubium vulgare* L., *Desmodium molliculum (*Kunth*)* DC., *Minthostachys mollis* (Kunth) Griseb., *Cydonia oblonga* Mill.
694. *Apium graveolens* L., *Matricaria frigidum* (H.B.K.) Kunth, *Matricaria recutita* L., *Ambrosia peruviana* Willd., *Origanum majorana* L., *Adiantum concinnum* Humb. & Bonpl. ex Willd.
695. *Ambrosia peruviana* Willd., *Matricaria frigidum* (H.B.K.) Kunth, *Matricaria recutita* L., *Borago officinalis* L., *Lonicera japonica* Thunb. ex Murray, *Melissa officinalis* L., *Foeniculum vulgare* Mill., *Marrubium vulgare* L., *Desmodium molliculum (*Kunth*)* DC.), *Minthostachys mollis* (Kunth) Griseb.
696. *Perezia multiflora* (Bonpl.) Less., *Piper aduncum* L., *Eucalyptus globulus* Labill., *Malesherbia ardens* J.F. Macbr., *Alternanthera brasiliana* (L.) Kuntze, *Stachys lanata* Jacq., *Senecio canescens* (Bonpl.) Cuatrec., *Juglans neotropica* Diels, *Oritrophium peruvianum* (Lam.) Cuatrec., *Laccopetalum giganteum* (Wedd.) Ulbr., *Senecio tephrosioides* Turcz., *Tilia platyphyllos* Scop., *Rubus robustus* C.Presl.
697. *Senecio canescens* (Bonpl.) Cuatrec., *Borago officinalis* L., *Eucalyptus globulus* Labill., *Perezia multiflora* (Bonpl.) Less., *Sonchus oleraceus* L., *Matricaria frigidum* (H.B.K.) Kunth, *Matricaria recutita* L., *Ambrosia peruviana* Willd, *Melissa officinalis* L., *Peperomia inaequalifolia* Ruiz & Pav., *Mentha* x *piperita* L., *Dianthus caryophyllus* L., *Tagetes erecta* L., *Acanthoxanthium spinosum* (L.) Fourr., *Aphelandra cirsioides* Lindau
698. *Tagetes erecta* L., *Melissa officinalis* L., *Sanguisorba minor* Scop., *Mentha x piperita* L., *Matricaria frigidum* (H.B.K.) Kunth, *Matricaria recutita* L., *Ambrosia peruviana* Willd
699. *Borago officinalis* L., *Oritrophium peruvianum* (Lam.) Cuatrec., *Senecio canescens* (Bonpl.) Cuatrec.
700. *Tillandsia cacticola* L.B. Sm., *Rosmarinus officinalis* L., *Salvia rosmarinifolia* G. Don, *Bursera graveolens* (Kunth) Triana & Planch., *Lavandula angustifolia* Mill.
701. *Tillandsia multiflora* Benth. var. *decipiens* (André) L.B. Sm., *Melissa officinalis* L., *Dianthus caryophyllus* L., *Citrus limetta* Risso
702. *Sambucus peruviana* Kunth, *Matricaria frigidum* (H.B.K.) Kunth, *Matricaria recutita* L., *Foeniculum vulgare* Mill., *Ambrosia peruviana* Willd., *Melissa officinalis* L., *Sanguisorba minor* Scop., *Dianthus caryophyllus* L., *Tagetes erecta* L.
703. *Dianthus caryophyllus* L., *Baccharis salicifolia* (Ruiz & Pav.) Pers., *Peperomia fraseri* C. DC., *Peperomia hartwegiana* Miq., *Hesperoxiphion niveum* (Ravenna) Ravenna, *Ruta graveolens* L., *Rosmarinus officinalis* L., *Salvia rosmarinifolia* G. Don
704. *Sanguisorba minor* Scop., *Matricaria frigidum* (H.B.K.) Kunth, *Matricaria recutita* L., *Ambrosia peruviana* Willd, *Melissa officinalis* L., *Borago officinalis* L., *Rosmarinus officinalis* L., *Salvia rosmarinifolia* G. Don, *Marrubium vulgare* L., *Desmodium molliculum (*Kunth*)* DC.), *Minthostachys mollis* (Kunth) Griseb.
705. *Lathyrus odoratus* L., *Melissa officinalis* L., *Sanguisorba minor* Scop., *Origanum majorana* L., *Cymbopogon citratus* (DC.) Stapf., *Aloysia triphylla* (L'Hér.) Britton
706. *Lavandula angustifolia* Mill., *Rosmarinus officinalis* L., *Salvia rosmarinifolia* G. Don, *Dianthus caryophyllus* L., *Tagetes erecta* L., *Foeniculum vulgare* Mill., *Melissa officinalis* L., *Matricaria frigidum* (H.B.K.) Kunth, *Matricaria recutita* L., *Ambrosia peruviana* Willd, *Sanguisorba minor* Scop.
707. *Origanum majorana* L., *Alternanthera halimifolia* (Lam.) Standl. ex Pittier, *Peperomia inaequalifolia* Ruiz & Pav., *Melissa officinalis* L., *Matricaria frigidum* (H.B.K.) Kunth, *Matricaria recutita* L., *Ambrosia peruviana* Willd, *Foeniculum vulgare* Mill., *Hyptis sidifolia* (L'Hér.) Briq., *Mentha x piperita* L.

708. *Rosmarinus officinalis* L., *Eucalyptus globulus* Labill., *Equisetum bogotense* Kunth, *Equisetum giganteum* L., *Ruta graveolens* L.
709. *Satureja pulchella* (Kunth) Briq., *Otholobium glandulosum* (L.) J.W. Grimes, *Matricaria frigidum* (H.B.K.) Kunth, *Matricaria recutita* L., *Ambrosia peruviana* Willd, *Marrubium vulgare* L., *Desmodium molliculum (*Kunth*)* DC., *Minthostachys mollis* (Kunth) Griseb.
710. *Malva sylvestris* L., *Melissa officinalis* L., *Sanguisorba minor* Scop., *Origanum majorana* L., *Viola tricolor* L., *Lathyrus odoratus* L., *Cymbopogon citratus* (DC.) Stapf., *Aloysia triphylla* (L'Hér.) Britton
711. *Ximenia americana* L., *Citrus limon* (L.) Burm. f., *Satureja pulchella* (Kunth) Briq., *Foeniculum vulgare* Mill., *Ambrosia peruviana* Willd., *Melissa officinalis* L., *Sanguisorba minor* Scop.
712. *Stelis eublepharis* Rchb. f., *Baccharis salicifolia* (Ruiz & Pav.) Pers., *Peperomia fraseri* C. DC., *Peperomia hartwegiana* Miq., *Hesperoxiphion niveum* (Ravenna) Ravenna, *Oenothera rosea* L'Hér. ex Aiton, *Cheilanthes myriophylla* Desv., *Baccharis vaccinioides* Kunth
713. *Oxalis bulbigera* R. Knuth, *Melissa officinalis* L., *Mentha x piperita* L., *Matricaria frigidum* (H.B.K.) Kunth, *Matricaria recutita* L., *Ambrosia peruviana* Willd, *Foeniculum vulgare* Mill., *Rosmarinus officinalis* L., *Salvia rosmarinifolia* G. Don
714. *Passiflora caerulea* L., *Melissa officinalis* L., *Sanguisorba minor* Scop., *Desmodium molliculum (*Kunth*)* DC., *Minthostachys mollis* (Kunth) Griseb., *Rosmarinus officinalis* L., *Salvia rosmarinifolia* G. Don
715. *Peperomia fraseri* C. DC., *Werneria villosa* A. Gray, *Stelis eublepharis* Rchb. f., *Hypericum laricifolium* Juss., *Phyllactis rigida* (Ruiz & Pav.) Pers.
716. *Peperomia fraseri* C. DC., *Tillandsia cacticola* L.B. Sm., *Tillandsia multiflora* Benth. var. *decipiens* (André) L.B. Sm., *Echeveria peruviana* Meyen., *Melissa officinalis* L., *Sanguisorba minor* Scop., *Rosmarinus officinalis* L., *Salvia rosmarinifolia* G. Don, *Origanum majorana* L., *Viola tricolor* L., *Lathyrus odoratus* L.
717. *Peperomia galioides* Kunth, *Pelargonium odoratissimum* (L.) L'Hér., *Tillandsia cacticola* L.B. Sm., *Tillandsia multiflora* Benth. var. *decipiens* (André) L.B. Sm., *Echeveria peruviana* Meyen., *Pilea microphylla* (L.) Liebm.
718. *Cydonia oblonga* Mill., *Matricaria frigidum* (H.B.K.) Kunth, *Matricaria recutita* L., *Ambrosia peruviana* Willd, *Melissa officinalis* L., *Foeniculum vulgare* Mill., *Lonicera japonica* Thunb. ex Murray, *Rosmarinus officinalis* L., *Salvia rosmarinifolia* G. Don
719. *Cydonia oblonga* Mill., *Matricaria frigidum* (H.B.K.) Kunth, *Matricaria recutita* L., *Ambrosia peruviana* Willd, *Melissa officinalis* L., *Sanguisorba minor* Scop., *Borago officinalis* L., *Viola tricolor* L., *Lathyrus odoratus* L., *Origanum majorana* L., *Rosmarinus officinalis* L., *Salvia rosmarinifolia* G. Don
720. *Fragaria vesca* L., *Foeniculum vulgare* Mill., *Matricaria frigidum* (H.B.K.) Kunth, *Matricaria recutita* L., *Ambrosia peruviana* Willd, *Sanguisorba minor* Scop.
721. *Sanguisorba minor* Scop., *Bejaria aestuans* Mutis ex L., *Cenchrus echinatus* L., *Tribulus terrestris* L., *Baccharis genistelloides* (Lam.) Pers., *Bidens pilosa* L., *Alternanthera porrigens* (Jacq.) Kuntze, *Cuphea strigulosa* Kunth, Melissa *officinalis* L., *Peperomia inaequalifolia* Ruiz & Pav., *Dianthus caryophyllus* L., *Matricaria frigidum* (H.B.K.) Kunth, *Matricaria recutita* L., *Ambrosia peruviana* Willd., *Cestrum nocturnum* L.
722. *Citrus aurantium* L., *Mentha x piperita* L., *Pimpinella anisum* L.
723. *Citrus limetta* Risso, *Matricaria frigidum* (H.B.K.) Kunth, *Matricaria recutita* L., *Ambrosia peruviana* Willd, *Foeniculum vulgare* Mill., *Melissa officinalis* L., *Rosmarinus officinalis* L., *Salvia rosmarinifolia* G. Don, *Borago officinalis* L., *Lonicera japonica* Thunb. ex Murray, *Gentianella crassicaulis* J.S. Pringle
724. *Citrus limon* (L.) Burm. f., *Matricaria frigidum* (H.B.K.) Kunth, *Matricaria recutita* L., *Ambrosia peruviana* Willd, *Melissa officinalis* L., *Sanguisorba minor* Scop., *Gentianella crassicaulis* J.S. Pringle, *Dianthus caryophyllus* L., *Tagetes erecta* L.
725. *Citrus reticulata* Blanco, *Origanum majorana* L., *Melissa officinalis* L., *Sanguisorba minor* Scop., *Borago officinalis* L., *Matricaria frigidum* (H.B.K.) Kunth, *Matricaria recutita* L., *Ambrosia peruviana* Willd
726. *Citrus sinensis* (L.) Osbeck, *Melissa officinalis* L, *Dianthus caryophyllus* L., *Tagetes erecta* L., *Matricaria frigidum* (H.B.K.) Kunth, *Matricaria recutita* L., *Ambrosia peruviana* Willd, *Origanum majorana* L., *Marrubium vulgare* L., *Desmodium molliculum (*Kunth*)* DC., *Minthostachys mollis* (Kunth) Griseb., *Rosmarinus officinalis* L., *Salvia rosmarinifolia* G. Don
727. *Ruta graveolens* L., *Gentianella dianthoides* (Kunth) Fabris ex J.S. Pringle, *Gentianella bicolor* (Wedd.) J.S. Pringle, *Minthostachys mollis* (Kunth) Griseb., *Origanum vulgare* L., *Schinus molle* L., *Eucalyptus globulus* Labill., *Ambrosia peruviana* Willd., *Artemisia absinthium* L., *Adiantum concinnum* Humb. & Bonpl. ex Willd.
728. *Populus deltoides* W. Bartram ex Marshall, *Matricaria frigidum* (H.B.K.) Kunth, *Matricaria recutita* L., *Ambrosia peruviana* Willd, *Melissa officinalis* L., *Sanguisorba minor* Scop., *Foeniculum vulgare* Mill., *Marrubium vulgare* L., *Desmodium molliculum (*Kunth*)* DC.), *Minthostachys mollis* (Kunth) Griseb., *Cydonia oblonga* Mill.
729. *Tilia platyphyllos* Scop., *Sambucus peruviana* Kunth, *Matricaria frigidum* (H.B.K.) Kunth, *Matricaria recutita* L., *Ambrosia peruviana* Willd, *Foeniculum vulgare* Mill., *Melissa officinalis* L., *Sanguisorba minor* Scop., *Dianthus caryophyllus* L., *Tagetes erecta* L.,
730. *Aloysia triphylla* (L'Hér.) Britton, *Marrubium vulgare* L., *Desmodium molliculum (*Kunth*)* DC.), *Minthostachys mollis* (Kunth) Griseb., *Melissa officinalis* L., *Foeniculum vulgare* Mill., *Lonicera japonica* Thunb. ex Murray, *Dianthus caryophyllus* L., *Tagetes erecta* L., *Viola tricolor* L., *Lathyrus odoratus* L.
731. *Viola tricolor* L., *Melissa officinalis* L.
732. *Matricaria frigidum* (H.B.K.) Kunth, *Matricaria recutita* L., *Ambrosia peruviana* Willd, *Melissa officinalis* L., *Sanguisorba minor* Scop., *Foeniculum vulgare* Mill.
733. *Iresine herbstii* Hook., *Pilea microphylla* (L.) Liebm.
734. *Petroselinum crispum* (Mill.) Fuss, *Melissa officinalis* L., *Sanguisorba minor* Scop., *Origanum majorana* L., *Tillandsia cacticola* L.B. Sm., *Tillandsia multiflora* Benth. var. *decipiens* (André) L.B. Sm., *Echeveria peruviana* Meyen
735. *Hedyosmum racemosum* (Ruiz & Pav.) G. Don, *Heisteria acuminata* (Humb. & Bonpl.) Engl., *Cinchona officinalis* L.
736. *Myroxylon balsamum* (L.) Harms, *Couepia* sp., *Trichilia* sp., *Strychnos* sp., *Achyrocline alata* (Kunth) DC., *Aiouea dubia* (Kunth) Mez., *Nectandra reticulata* (Ruiz & Pav.) Mez., *Myristica fragrans* Houtt., *Thevetia peruviana* (Pers.) K. Schum.
737. *Melissa officinalis* L., *Sanguisorba minor* Scop., *Cymbopogon citratus* (DC.) Stapf., *Aloysia triphylla* (L'Hér.) Britton, *Origanum majorana* L., *Tillandsia cacticola* L.B. Sm., *Tillandsia multiflora* Benth. var. *decipiens* (André) L.B. Sm., *Echeveria peruviana* Meyen, *Rosmarinus officinalis* L., *Salvia rosmarinifolia* G. Don, *Dianthus caryophyllus* L., *Peperomia inaequalifolia* Ruiz &

Pav.*Matricaria frigidum* (H.B.K.) Kunth, *Matricaria recutita* L., *Ambrosia peruviana* Willd., *Sanguisorba minor* Scop., *Citrus aurantium* L.

738. *Nectandra reticulata* (Ruiz & Pav.) Mez., *Marrubium vulgare* L., *Dianthus caryophyllus* L., *Tagetes erecta* L., *Eucalyptus globulus* Labill.
739. *Nectandra reticulata* (Ruiz & Pav.) Mez., *Cymbopogon citratus* (DC.) Stapf., *Aloysia triphylla* (L'Hér.) Britton, *Strychnos* sp., *Myroxylon balsamum* (L.) Harms
740. *Cuphea strigulosa* Kunth, *Peperomia inaequalifolia* Ruiz & Pav., *Dianthus caryophyllus* L., *Tagetes erecta* L., *Lonicera japonica* Thunb. ex Murray, *Urtica magellanica* Juss. ex Poir., *Urtica urens* L., *Alternanthera halimifolia* (Lam.) Standl. ex Pittier, *Alternanthera porrigens* (Jacq.) Kuntze, *Alternanthera brasiliana* (L.) Kuntze, *Pilea microphylla* (L.) Liebm., *Iresine herbstii* Hook., *Erodium cicutarium* (L.) L'Hér. ex Aiton, *Desmodium molliculum (*Kunth*)* DC., *Equisetum bogotense* Kunth, *Equisetum giganteum* L., *Verbena litoralis* Kunth, *Sanguisorba minor* Scop., *Buddleja utilis* Kraenzl., *Cynodon dactylon* (L.) Pers., *Bejaria aestuans* Mutis ex L., *Cenchrus echinatus* L., *Tribulus terrestris* L., *Baccharis genistelloides* (Lam.) Pers., *Bidens pilosa* L.
741. *Sanguisorba minor* Scop., *Bejaria aestuans* Mutis ex L., *Cenchrus echinatus* L., *Tribulus terrestris* L., *Baccharis genistelloides* (Lam.) Pers., *Bidens pilosa* L., *Alternanthera porrigens* (Jacq.) Kuntze, *Cuphea strigulosa* Kunth, *Melissa officinalis* L., *Peperomia inaequalifolia* Ruiz & Pav., *Dianthus caryophyllus* L., *Matricaria frigidum* (H.B.K.) Kunth, *Matricaria recutita* L., *Ambrosia peruviana* Willd, *Cestrum nocturnum* L.

Nervousness

742. *Zornia reticulata* Sm., *Schkuhria pinnata* (Lam.) Kuntze ex Thell., *Polygala paniculata* L., *Iresine herbstii* Hook.
743. *Peperomia galioides* Kunth, *Pelargonium odoratissimum* (L.) L'Hér., *Tillandsia cacticola* L.B. Sm., *Tillandsia multiflora* Benth. var. *decipiens* (André) L.B. Sm. *Echeveria peruviana* Meyen, *Pilea microphylla* (L.) Liebm.

Nostalgic Anxiety/Emotional Trauma

744. *Peperomia galioides* Kunth, *Pelargonium odoratissimum* (L.) L'Hér., *Tillandsia cacticola* L.B. Sm., *Tillandsia multiflora* Benth. var. *decipiens* (André) L.B. Sm. *Echeveria peruviana* Meyen, *Pilea microphylla* (L.) Liebm.
745. *Peperomia inaequalifolia* Ruiz & Pav., *Melissa officinalis* L., *Sanguisorba minor* Scop., *Origanum majorana* L., *Viola tricolor* L., *Lathyrus odoratus* L.

Ovaries

746. *Taraxacum officinale* F.H. Wigg., *Mauria heterophylla* Kunth, *Desmodium molliculum (*Kunth*)* DC., *Equisetum bogotense* Kunth, *Equisetum giganteum* L., *Linum sativum* L., *Linum usitatissimum* L., *Monactis flaverioides* Kunth, *Malva sylvestris* L., *Alcea rosea* L., *Pelargonium odoratissimum* (L.) L'Hér., *Malva parviflora* L., *Bidens pilosa* L., *Aristolochia ruiziana* (Klotzsch) Duch.
747. *Lantana scabiosiflora* Kunth, *Schkuhria pinnata* (Lam.) Kuntze ex Thell., *Polygala paniculata* L., *Adiantum concinnum* Humb. & Bonpl. ex Willd., *Bejaria aestuans* Mutis ex L., *Satureja pulchella* (Kunth) Briq., *Lepechinia meyenii* (Walp.) Epling\

Pain

748. *Aloysia triphylla* (L'Hér.) Britton, *Lavandula angustifolia* Mill., *Origanum vulgare* L., *Piper nigrum* L.
749. *Tribulus terrestris* L., *Bidens pilosa* L., *Arctium lappa* L., *Centropogon* cf. *rufus* E. Wimm.

Pain of Love

750. *Dianthus caryophyllus* L., *Baccharis salicifolia* (Ruiz & Pav.) Pers., *Peperomia fraseri* C. DC., *Peperomia hartwegiana* Miq., *Hesperoxiphion niveum* (Ravenna) Ravenna, *Ruta graveolens* L., *Rosmarinus officinalis* L., *Salvia rosmarinifolia* G. Don
751. *Melissa officinalis* L., *Sanguisorba minor* Scop., *Cymbopogon citratus* (DC.) Stapf., *Aloysia triphylla* (L'Hér.) Britton, *Origanum majorana* L., *Tillandsia cacticola* L.B. Sm., *Rosmarinus officinalis* L., *Salvia rosmarinifolia* G. Don, *Dianthus caryophyllus* L., *Tagetes erecta* L., *Peperomia inaequalifolia* Ruiz & Pav., *Matricaria frigidum* (H.B.K.) Kunth, *Matricaria recutita* L., *Ambrosia peruviana* Willd., *Origanum majorana* L., *Sanguisorba minor* Scop., *Citrus sinensis* (L.) Osbeck
752. *Origanum majorana* L., *Alternanthera halimifolia* (Lam.) Standl. ex Pittier, *Peperomia inaequalifolia* Ruiz & Pav., *Melissa officinalis* L., *Foeniculum vulgare* Mill., *Hyptis sidifolia* (L'Hér.) Briq., *Mentha x piperita* L., *Matricaria frigidum* (H.B.K.) Kunth, *Matricaria recutita* L., *Ambrosia peruviana* Willd.
753. *Rosmarinus officinalis* L., *Eucalyptus globulus* Labill., *Equisetum bogotense* Kunth, *Equisetum giganteum* L., *Ruta graveolens* L.
754. *Sanguisorba minor* Scop., *Bejaria aestuans* Mutis ex L., *Cenchrus echinatus* L., *Tribulus terrestris* L., *Baccharis genistelloides* (Lam.) Pers., *Bidens pilosa* L., *Alternanthera porrigens* (Jacq.) Kuntze, *Cuphea strigulosa* Kunth, *Melissa officinalis* L., *Peperomia inaequalifolia* Ruiz & Pav., *Dianthus carthusianorum* L., *Matricaria frigidum* (H.B.K.) Kunth, *Matricaria recutita* L., *Ambrosia peruviana* Willd., *Cestrum nocturnum* L.
755. *Viola tricolor* L., *Melissa officinalis* L.

Parasites

756. *Cocos nucifera* L., *Cymbopogon citratus* (DC.) Stapf., *Otholobium glandulosum* (L.) J.W. Grimes, *Foeniculum vulgare* Mill., *Mentha x piperita* L.
757. *Mentha spicata* L., *Pimpinella anisum* L.

Pharyngitis

758. *Caesalpinia spinosa* (Molina) Kuntze, *Rosmarinus officinalis* L., *Salvia rosmarinifolia* G. Don, *Erythroxylum coca* Lam, *Croton draconoides* Muell.-Arg., *Croton lechleri* Muell.-Arg.

Pimples

759. *Sarcostemma clausum* (Jacq.) Schult., *Spartium junceum* L., *Polylepis racemosa* Ruiz & Pav., *Solanum tuberosum* L., *Eucalyptus globulus* Labill.

Pneumonia

760. *Picrosia longifolia* D. Don, *Verbena litoralis* Kunth, *Schkuhria pinnata* (Lam.) Kuntze ex Thell., *Polygala paniculata* L.
761. *Oritrophium peruvianum* (Lam.) Cuatrec., *Laccopetalum giganteum* (Wedd.) Ulbr., *Senecio tephrosioides* Turcz., *Verbena litoralis* Kunth, *Senecio canescens* (Bonpl.) Cuatrec., *Clerodendron* sp.,

Promoting Lactation

762. *Chamaesyce hypericifolia* (L.) Millsp., *Equisetum bogotense* Kunth, *Equisetum giganteum* L., *Bidens pilosa* L., *Linum sativum* L., *Linum usitatissimum* L., *Mauria heterophylla* Kunth, *Sarcostemma clausum* (Jacq.) Schult.

Prostate

763. *Bidens pilosa* L., *Mauria heterophylla* Kunth, *Eugenia obtusifolia* Cambess., *Tiquilia paronychioides* (Phil.) A.T. Richardson, *Zea mays* L., *Equisetum bogotense* Kunth, *Equisetum giganteum* L., *Psidium guajava* L., *Sanguisorba minor* Scop., *Cestrum nocturnum* L.
764. *Monactis flaverioides* Kunth, *Artemisia absinthium* L., *Salvia discolor* Kunth, *Ambrosia peruviana* Willd., *Miconia salicifolia* (Bonpl. ex Naudin) Naudin
765. *Bixa orellana* L., *Uncaria tomentosa* (Willd. ex Roem. & Schult.) DC., *Mimosa nothacacia* Barneby
766. *Cordia lutea* Lam, *Plantago linearis* Kunth, *Plantago major* L., *Peumus boldus* Molina,
767. *Tiquilia paronychioides* (Phil.) A.T. Richardson, *Monactis flaverioides* Kunth, *Malva sylvestris* L., *Alcea rosea* L., *Pelargonium odoratissimum* (L.) L'Hér., *Malva parviflora* L., *Zea mays* L., *Equisetum bogotense* Kunth, *Equisetum giganteum* L., *Pilea microphylla* (L.) Liebm., *Buddleja utilis* Kraenzl., *Bejaria aestuans* Mutis ex L., *Cenchrus echinatus* L., *Tribulus terrestris* L., *Baccharis genistelloides* (Lam.) Pers., *Bidens pilosa* L., *Rorippa nasturtium-aquaticum* (L.) Hayek, *Typha angustifolia* L., *Bixa orellana* L., *Alternanthera porrigens* (Jacq.) Kuntze, *Cuphea strigulosa* Kunth, *Eustephia coccinea* Cav.
768. *Capsella bursa-pastoris* (L.) Medik., *Mauria heterophylla* Kunth, *Verbena litoralis* L., *Zea mays* L., *Buddleja utilis* Kraenzl., *Tiquilia paronychioides* (Phil.) A.T. Richardson, *Geranium ayavacense* Willd. ex Kunth, *Geranium sessiliflorum* Cav., *Gentianella bicolor* (Wedd.) J.S. Pringle, *Equisetum bogotense* Kunth, *Equisetum giganteum* L.,
769. *Sambucus peruviana* Kunth, *Ricinus communis* L.
770. *Equisetum giganteum* L., *Verbena litoralis* Kunth, *Matricaria frigidum* (H.B.K.) Kunth, *Matricaria recutita* L., *Ambrosia peruviana* Willd., *Mauria heterophylla* Kunth, *Eugenia obtusifolia* Cambess., *Zea mays* L., *Iresine diffusa* Humb. & Bonpl. ex Willd., *Plantago sericea* Ruiz & Pav. subsp. *sericans* (Pilg.) Rahn, *Rorippa nasturtium-aquaticum* (L.) Hayek, *Desmodium molliculum (*Kunth*)* DC., *Dioscorea trifida* L. f.
771. *Bejaria aestuans* Mutis ex L., *Buddleja utilis* Kraenzl., *Dioscorea trifida* L. f., *Tiquilia paronychioides* (Phil.) A.T. Richardson, *Ilex guayusa* Loes., *Geranium ayavacense* Willd. ex Kunth, *Geranium sessiliflorum* Cav., *Monactis flaverioides* Kunth, *Malva sylvestris* L., *Alcea rosea* L., *Pelargonium odoratissimum* (L.) L'Hér., *Malva parviflora* L., *Bidens pilosa* L., *Verbena litoralis* Kunth, *Plantago linearis* Kunth, *Plantago major* L., *Equisetum bogotense* Kunth, *Equisetum giganteum* L., *Muehlenbeckia tamnifolia* (Kunth) Meisn., *Smilax medica* Schltdl. & Cham., *Oreocallis grandiflora* (Lam.) R. Br., *Cinchona officinalis* L.
772. *Linum usitatissimum* L., *Linum sativum* L., *Equisetum bogotense* Kunth, *Equisetum giganteum* L., *Phyllanthus niruri* L., *Phyllanthus stipulatus* (Raf.) G.L.Webster, *Phyllanthus urinaria* L., *Lycaste gigantea* Lindl., *Peumus boldus* Molina, *Cordia lutea* Lam
773. *Mirabilis jalapa* L., *Tiquilia paronychioides* (Phil.) A.T. Richardson
774. *Saccharum officinarum* L., *Equisetum bogotense* Kunth, *Equisetum giganteum* L., *Linum sativum* L., *Linum usitatissimum* L., *Phyllanthus niruri* L., *Phyllanthus stipulatus* (Raf.) G.L.Webster, *Phyllanthus urinaria* L., *Peumus boldus* Molina, *Desmodium molliculum (*Kunth*)* DC.
775. *Uncaria tomentosa* (Willd. ex Roem. & Schult.) DC., *Phyllanthus niruri* L., *Phyllanthus stipulatus* (Raf.) G.L.Webster, *Phyllanthus urinaria* L., *Linum sativum* L., *Linum usitatissimum* L., *Peumus boldus* Molina, *Cordia lutea* Lam, *Capsella bursa-pastoris* (L.) Medik.
776. *Typha angustifolia* L., *Erodium cicutarium* (L.) L'Hér. ex Aiton, *Bixa orellana* L.
777. *Pilea microphylla* (L.) Liebm., *Equisetum bogotense* Kunth, *Equisetum giganteum* L., *Bixa orellana* L., *Phyllanthus niruri* L., *Phyllanthus stipulatus* (Raf.) G.L.Webster, *Phyllanthus urinaria* L.
778. *Urtica urens* L., *Artemisia absinthium* L., *Salvia discolor* Kunth, *Ambrosia peruviana* Willd., *Miconia salicifolia* (Bonpl. ex Naudin) Naudin

Protection

779. *Digitaria ciliaris* (Retz.) Koeler, *Phyllactis rigida* (Ruiz & Pav.) Pers., *Tetragonia crystallina* L'Hér., *Jamesonia goudotii* (Hieron.) C. Chr., *Xyris subulata* Ruiz & Pav.
780. *Cyperus articulatus* L., *Baccharis salicifolia* (Ruiz & Pav.) Pers., *Peperomia fraseri* C. DC., *Peperomia hartwegiana* Miq., *Hesperoxiphion niveum* (Ravenna) Ravenna, *Jamesonia goudotii* (Hieron.) C. Chr., *Xyris subulata* Ruiz & Pav., *Tetragonia crystallina* L'Hér., *Werneria villosa* A. Gray, *Stelis eublepharis* Rchb. f., *Valeriana bonplandiana* Wedd., *Sambucus peruviana* Kunth, *Citrus limetta* Risso
781. *Gentianella bicolor* (Wedd.) J.S. Pringle, *Baccharis salicifolia* (Ruiz & Pav.) Pers., *Peperomia fraseri* C. DC., *Peperomia hartwegiana* Miq., *Hypericum laricifolium* Juss., *Hesperoxiphion niveum* (Ravenna) Ravenna, *Werneria villosa* A. Gray, *Stelis eublepharis* Rchb. f., *Jamesonia goudotii* (Hieron.) C. Chr., *Tetragonia crystallina* L'Hér.
782. *Valeriana plantaginea* Kunth, *Brugmansia candida* Pers., *Brugmansia arborea* (L.) Lagerh., *Brugmansia sanguinea* (Ruiz & Pav.) D. Don, *Gaultheria reticulata* Kunth

Pulmonary Disease

783. *Cronquistianthus lavandulifolius* (DC.) R.M. King & H. Rob., *Piper aduncum* L., *Rubus robustus* C.Presl., *Juglans neotropica* Diels, *Lepechinia meyenii* (Walp.) Epling, *Salvia officinalis* L., *Salvia cuspidata* Ruiz & Pav., *Salvia sagittata* Ruiz. & Pav., *Borago*

officinalis L., *Salvia discolor* Kunth, *Ambrosia peruviana* Willd., *Miconia salicifolia* (Bonpl. ex Naudin) Naudin, *Oritrophium peruvianum* (Lam.) Cuatrec., *Senecio canescens* (Bonpl.) Cuatrec.
784. *Bixa orellana* L., *Uncaria tomentosa* (Willd. ex Roem. & Schult.) DC., *Mimosa nothacacia* Barneby

Rashes
785. *Sarcostemma clausum* (Jacq.) Schult., *Spartium junceum* L., *Polylepis racemosa* Ruiz & Pav., *Solanum tuberosum* L., *Eucalyptus globulus* Labill.
786. *Eupatorium triplinerve* Vahl, *Nerium oleander* L., *Myrica pubescens* Humb. & Bonpl. ex Willd.

Recovering
787. *Cinnamomum verum* J. Presl., *Piper aduncum* L., *Verbena litoralis* Kunth, *Clerodendron* sp., *Oritrophium peruvianum* (Lam.) Cuatrec., *Senecio canescens* (Bonpl.) Cuatrec.

Rehabilitation of Drug Addicts
788. *Inga edulis* Mart., *Acanthoxanthium spinosum* (L.) Fourr., *Senna bicapsularis* (L.) Roxb.

Relaxation
789. *Alternanthera halimifolia* (Lam.) Standl. ex Pittier, *Melissa officinalis* L., *Matricaria frigidum* (H.B.K.) Kunth, *Matricaria recutita* L., *Ambrosia peruviana* Willd., *Rosmarinus officinalis* L., *Salvia rosmarinifolia* G. Don, *Foeniculum vulgare* Mill., *Marrubium vulgare* L., *Desmodium molliculum (*Kunth*)* DC., *Minthostachys mollis* (Kunth) Griseb., *Cydonia oblonga* Mill.
790. *Lavandula angustifolia* Mill., *Rosmarinus officinalis* L., *Salvia rosmarinifolia* G. Don, *Eucalyptus globulus* Labill.
791. *Cestrum auriculatum* L'Hér., *Polylepis racemosa* Ruiz & Pav., *Eucalyptus globulus* Labill., *Salvia rosmarinifolia* G. Don

Renal Bleeding
792. *Acmella* cf. *ciliata* (Kunth) Cass., *Mentha spicata* L.

Renal Disease
793. *Stellaria media* (L.) Vill., *Monactis flaverioides* Kunth, *Malva sylvestris* L., *Alcea rosea* L., *Pelargonium odoratissimum* (L.) L'Hér., *Malva parviflora* L., *Bidens pilosa* L., *Mauria heterophylla* Kunth, *Eugenia obtusifolia* Cambess.
794. *Cyclanthera pedata* (L.) Schrad., *Sechium edule* (Jacq.) Sw., *Daucus carota* L.
795. *Dioscorea trifida* L. f., *Buddleja utilis* Kraenzl., *Bejaria aestuans* Mutis ex L., *Paranephelius uniflorus* Poepp., *Rorippa nasturtium-aquaticum* (L.) Hayek
796. *Mirabilis jalapa* L., *Tiquilia paronychioides* (Phil.) A.T. Richardson

Rheumatism
797. *Baccharis latifolia* (Ruiz & Pav.) Pers., *Matricaria recutita* L., *Monactis flaverioides* Kunth, *Nerium oleander* L., *Myrica pubescens* Humb. & Bonpl. ex Willd.
798. *Hedyosmum racemosum* (Ruiz & Pav.) G. Don, *Heisteria acuminata* (Humb. & Bonpl.) Engl., *Cinchona officinalis* L.
799. *Allium sativum* L., *Eucalyptus globulus* Labill., *Laccopetalum giganteum* (Wedd.) Ulbr., *Heisteria acuminata* (Humb. & Bonpl.) Engl., *Zingiber officinale* Roscoe
800. *Siparuna muricata* (Ruiz & Pav.) A. DC., *Monactis flaverioides* Kunth, *Achyrocline alata* (Kunth) DC., *Aiouea dubia* (Kunth) Mez., *Nectandra reticulata* (Ruiz & Pav.) Mez., *Rosmarinus officinalis* L., *Salvia rosmarinifolia* G. Don, *Ruta graveolens* L., *Escallonia pendula* (Ruiz & Pav.) Pers., *Bursera graveolens* (Kunth) Triana & Planch.
801. *Muehlenbeckia tamnifolia* (Kunth) Meisn., *Bejaria aestuans* Mutis ex L.
802. *Uncaria tomentosa* (Willd. ex Roem. & Schult.) DC., *Phyllanthus niruri* L., *Phyllanthus urinaria* L., *Phyllanthus stipulatus* (Raf.) G.L.Webster, *Linum sativum* L., *Linum usitatissimum* L., *Peumus boldus* Molina, *Cordia lutea* Lam, *Capsella bursa-pastoris* (L.) Medik.
803. *Escallonia pendula* (Ruiz & Pav.) Pers., *Oreocallis grandiflora* (Lam.) R. Br., *Vallea stipularis* L. f., *Achyrocline alata* (Kunth) DC., *Artemisia absinthium* L.
804. *Urtica magellanica* Juss. ex Poir., *Oritrophium peruvianum* (Lam.) Cuatrec., *Laccopetalum giganteum* (Wedd.) Ulbr., *Senecio tephrosioides* Turcz., *Verbena litoralis* Kunth, *Gentianella bicolor* (Wedd.) J.S. Pringle, *Sonchus oleraceus* L., *Juglans neotropica* Diels, *Schinus molle* L., *Ruta graveolens* L., *Piper aduncum* L.

Scars
805. *Desmodium molliculum* (Kunth) DC., *Plantago linearis* Kunth, *Plantago major* L., *Piper aduncum* L.

Sexual Potency
806. *Ipomoea pauciflora* M. Martens & Galeotti, *Corynaea crassa* Hook. f., *Laccopetalum giganteum* (Wedd.) Ulbr., *Brosimum rubescens* Taub., *Celtis loxensis* C.C. Berg, *Heisteria acuminata* (Humb. & Bonpl.) Engl., *Cinchona officinalis* L., *Eustephia coccinea* Cav.
807. *Brosimum rubescens* Taub., *Celtis loxensis* C.C. Berg, *Cinchona officinalis* L., *Heisteria acuminata* (Humb. & Bonpl.) Engl., *Laccopetalum giganteum* (Wedd.) Ulbr., *Ipomoea pauciflora* M. Martens & Galeotti, *Corynaea crassa* Hook. f.
808. *Myristica fragrans* Houtt., *Brosimum rubescens* Taub., *Celtis loxensis* C.C. Berg, *Laccopetalum giganteum* (Wedd.) Ulbr., *Corynaea crassa* Hook. f., *Eustephia coccinea* Cav.
809. *Cinchona officinalis* L., *Brosimum rubescens* Taub., *Celtis loxensis* C.C. Berg, *Laccopetalum giganteum* (Wedd.) Ulbr., *Isoetes andina* Spruce ex Hook., *Peperomia quadrifolia* (L.) Kunth, *Corynaea crassa* Hook. f.
810. *Celtis loxensis* C.C. Berg, *Brosimum rubescens* Taub., *Heisteria acuminata* (Humb. & Bonpl.) Engl., *Laccopetalum giganteum* (Wedd.) Ulbr., *Cinchona officinalis* L., *Corynaea crassa* Hook. f.

Sharp Pain (internal)

811. *Perezia multiflora* (Bonpl.) Less., *Piper aduncum* L., *Eucalyptus globulus* Labill., *Verbena litoralis* Kunth, *Senecio canescens* (Bonpl.) Cuatrec., *Juglans neotropica* Diels, *Oritrophium peruvianum* (Lam.) Cuatrec., *Laccopetalum giganteum* (Wedd.) Ulbr., *Senecio tephrosioides* Turcz., *Tilia platyphyllos* Scop., *Rubus robustus* C.Presl.

Sinusitis

812. *Salvia rosmarinifolia* G. Don, *Lepechinia meyenii* (Walp.) Epling, *Salvia officinalis* L., *Salvia cuspidata* Ruiz & Pav., *Salvia sagittata* Ruiz. & Pav., *Origanum majorana* L.
813. *Salvia rosmarinifolia* G. Don, *Bursera graveolens* (Kunth) Triana & Planch., *Eucalyptus globulus* Labill.
814. *Eucalyptus globulus* Labill., *Matricaria frigidum* (H.B.K.) Kunth, *Matricaria recutita* L., *Ambrosia peruviana* Willd., *Piper aduncum* L., *Juglans neotropica* Diels, *Cordia alliodora* (Ruiz & Pav.) Oken
815. *Spartium junceum* L., *Gaultheria reticulata* Kunth
816. *Salvia rosmarinifolia* G. Don, *Schkuhria pinnata* (Lam.) Kuntze ex Thell., *Polygala paniculata* L., *Equisetum bogotense* Kunth, *Equisetum giganteum* L.

Skin

817. *Arctium lappa* L., *Bejaria aestuans* Mutis ex L., *Cenchrus echinatus* L., *Tribulus terrestris* L., *Baccharis genistelloides* (Lam.) Pers., *Bidens pilosa* L., *Centropogon* cf. *rufus* E. Wimm.
818. *Caesalpinia spinosa* (Molina) Kuntze, *Rosmarinus officinalis* L., *Salvia rosmarinifolia* G. Don, *Erythroxylum coca* Lam, *Croton draconoides* Muell.-Arg., *Croton lechleri* Muell.-Arg.
819. *Caesalpinia spinosa* (Molina) Kuntze, *Nerium oleander* L., *Myrica pubescens* Humb. & Bonpl. ex Willd., *Monactis flaverioides* Kunth, *Artemisia absinthium* L., *Achyrocline alata* (Kunth) DC., *Aiouea dubia* (Kunth) Mez., *Nectandra reticulata* (Ruiz & Pav.) Mez.
820. *Salvia ayavacensis* Kunth, *Senecio genisianus* Cuatrec., *Phytolacca bogotensis* Kunth, *Siphocampylus cutervensis* Zahlbr., *Daucus montanus* Humb. & Bonpl. ex Spreng., *Salvia rosmarinifolia* G. Don
821. *Cenchrus echinatus* L., *Bidens pilosa* L., *Arctium lappa* L., *Centropogon* cf. *rufus* E. Wimm.
822. *Tribulus terrestris* L., *Bidens pilosa* L., *Arctium lappa* L., *Centropogon* cf. *rufus* E. Wimm.

Skin Marks

823. *Sarcostemma clausum* (Jacq.) Schult., *Spartium junceum* L., *Polylepis racemosa* Ruiz & Pav., *Solanum tuberosum* L., *Eucalyptus globulus* Labill.

Sleep Aid

824. *Muehlenbeckia tamnifolia* (Kunth) Meisn., *Bejaria aestuans* Mutis ex L.

Snake Bite

825. *Sicyos baderoa* Hook. & Arn., *Vallesia glabra* (Cav.) Link

Sorcery

826. *Hydrocotyle bonariensis* Lam, *Cydista aequinoctialis* (L.) Miers, *Celtis loxensis* C.C. Berg
827. *Mandevilla* cf. *trianae* Woodson, *Daucus montanus* Humb. & Bonpl. ex Spreng., *Baccharis latifolia* (Ruiz & Pav.) Pers., *Acalypha mandonii* Müll. Arg., *Trixis cacalioides* (Kunth) D. Don, *Achyrocline alata* (Kunth) DC., *Centropogon reticulatus* Drake, *Fuchsia ayavacensis* Kunth, *Siphocampylus cutervensis* Zahlbr., *Monactis flaverioides* Kunth
828. *Artemisia absinthium* L., *Siparuna muricata* (Ruiz & Pav.) A. DC., *Trixis cacalioides* (Kunth) D. Don, *Tagetes erecta* L., *Tagetes patula* L., *Monactis flaverioides* Kunth
829. *Artemisia absinthium* L., *Siparuna muricata* (Ruiz & Pav.) A. DC., *Ruta graveolens* L.
830. *Ferreyranthus verbascifolius* (Kunth) H. Rob. & Brettell, *Cantua quercifolia* Juss., *Trixis cacalioides* (Kunth) D. Don, *Ruta graveolens* L. *Vallea stipularis* L. f., *Centropogon reticulatus* Drake, *Fuchsia ayavacensis* Kunth, *Siphocampylus cutervensis* Zahlbr., *Salvia ayavacensis* Kunth, *Matricaria frigidum* (H.B.K.) Kunth, *Matricaria recutita* L., *Ambrosia peruviana* Willd.
831. *Hypericum aciculare* Kunth, *Ricinus communis* L., *Cestrum auriculatum* L'Hér.
832. *Juglans neotropica* Diels, *Trixis cacalioides* (Kunth) D. Don, *Oreocallis grandiflora* (Lam.) R. Br., *Achyrocline alata* (Kunth) DC., *Sambucus peruviana* Kunth,
833. *Salvia ayavacensis* Kunth, *Senecio genisianus* Cuatrec., *Phytolacca bogotensis* Kunth, *Centropogon reticulatus* Drake, *Fuchsia ayavacensis* Kunth, *Siphocampylus cutervensis* Zahlbr., *Daucus montanus* Humb. & Bonpl. ex Spreng., *Salvia rosmarinifolia* G. Don
834. *Huperzia kuesteri* (Nessel) B. Øllg., *Brugmansia sanguinea* (Ruiz & Pav.) D. Don
835. *Escallonia pendula* (Ruiz & Pav.) Pers., *Oreocallis grandiflora* (Lam.) R. Br., *Vallea stipularis* L. f., *Achyrocline alata* (Kunth) DC., *Artemisia absinthium* L.
836. *Brugmansia arborea* (L.) Lagerh., *Gaultheria reticulata* Kunth

Sores

837. *Gaultheria reticulata* Kunth, *Brugmansia candida* Pers., *Brugmansia arborea* (L.) Lagerh., *Brugmansia sanguinea* (Ruiz & Pav.) D. Don

Speech Impediment

838. *Cantua buxifolia* Juss. ex Lam., *Eucalyptus globulus* Labill., *Marrubium vulgare* L., *Zea mays* L.

Spiritual Flowering

839. *Baccharis vaccinioides* Kunth, *Oritrophium peruvianum* (Lam.) Cuatrec., *Tetragonia crystallina* L'Hér., *Xyris subulata* Ruiz & Pav., *Desmodium triflorum* (L.) DC., *Boerhavia coccinea* Mill., *Tillandsia cacticola* L.B. Sm., *Tillandsia multiflora* Benth. var. *decipiens* (André) L.B. Sm., *Echeveria peruviana* Meyen, *Oreobolus goeppingeri* Suess., *Jamesonia goudotii* (Hieron.) C. Chr., *Peperomia quadrifolia* (L.) Kunth, *Bejaria aestuans* Mutis ex L., *Werneria villosa* A. Gray, *Stelis eublepharis* Rchb. f., *Baccharis salicifolia* (Ruiz & Pav.) Pers., *Peperomia fraseri* C. DC., *Peperomia hartwegiana* Miq., *Werneria pygmaea* Gillies ex Hook. & Arn., *Hypericum laricifolium* Juss.

Sterilization for Women

840. *Persea americana* Mill., *Linum sativum* L., *Linum usitatissimum* L.
841. *Aa paleacea* (Kunth) Rchb. f., *Mimosa albida* Humb. & Bonpl. ex Willd., *Sicana odorifera* (Vell.) Naudin

Stomach

842. *Taraxacum officinale* F.H. Wigg., *Mauria heterophylla* Kunth, *Desmodium molliculum (*Kunth*)* DC., *Equisetum bogotense* Kunth, *Equisetum giganteum* L., *Linum sativum* L., *Linum usitatissimum* L., *Monactis flaverioides* Kunth, *Malva sylvestris* L., *Alcea rosea* L., *Pelargonium odoratissimum* (L.) L'Hér., *Malva parviflora* L., *Bidens pilosa* L., *Aristolochia ruiziana* (Klotzsch) Duch.
843. *Capsella bursa-pastoris* (L.) Medik., *Mauria heterophylla* Kunth, *Verbena litoralis* Kunth, *Zea mays* L., *Buddleja utilis* Kraenzl., *Tiquilia paronychioides* (Phil.) A.T. Richardson, *Geranium ayavacense* Willd. ex Kunth, *Geranium sessiliflorum* Cav., *Gentianella bicolor* (Wedd.) J.S. Pringle, *Equisetum bogotense* Kunth, *Equisetum giganteum* L.,
844. *Equisetum bogotense* Kunth, *Cordia lutea* Lam, *Alternanthera porrigens* (Jacq.) Kuntze, *Cuphea strigulosa* Kunth, *Typha angustifolia* L., *Bixa orellana* L., *Smilax medica* Schltdl. & Cham.
845. *Equisetum bogotense* Kunth, *Desmodium molliculum (*Kunth*)* DC., *Mauria heterophylla* Kunth
846. *Desmodium molliculum* (Kunth) DC., *Mauria heterophylla* Kunth, *Bidens pilosa* L., *Verbena litoralis* Kunth
847. *Trifolium repens* L., *Alternanthera porrigens* (Jacq.) Kuntze, *Cuphea strigulosa* Kunth, *Iresine herbstii* Hook., *Smilax medica* Schltdl. & Cham.
848. *Rosmarinus officinalis* L., *Eucalyptus globulus* Labill., *Equisetum bogotense* Kunth, *Equisetum giganteum* L., *Ruta graveolens* L.
849. *Ximenia americana* L., *Citrus limon* (L.) Burm. f., *Satureja pulchella* (Kunth) Briq., *Foeniculum vulgare* Mill., *Ambrosia peruviana* Willd., *Melissa officinalis* L., *Sanguisorba minor* Scop.
850. *Argemone mexicana* L., *Equisetum bogotense* Kunth, *Equisetum giganteum* L., *Monactis flaverioides* Kunth,, *Malva sylvestris* L., *Alcea rosea* L., *Pelargonium odoratissimum* (L.) L'Hér., *Malva parviflora* L., *Plantago linearis* Kunth, *Plantago major* L., *Desmodium molliculum (*Kunth*)* DC.
851. *Citrus aurantium* L., *Mentha* x *piperita* L., *Pimpinella anisum* L.
852. *Aloysia triphylla* (L'Hér.) Britton, *Desmodium molliculum (*Kunth*)* DC., *Minthostachys mollis* (Kunth) Griseb., *Melissa officinalis* L., *Foeniculum vulgare* Mill., *Lonicera japonica* Thunb. ex Murray, *Dianthus caryophyllus* L., *Tagetes erecta* L., *Viola tricolor* L., *Lathyrus odoratus* L.

Stomach Pain

853. *Foeniculum vulgare* Mill., *Matricaria frigidum* (H.B.K.) Kunth, *Matricaria recutita* L., *Ambrosia peruviana* Willd., *Mentha x piperita* L., *Melissa officinalis* L., *Sanguisorba minor* Scop., *Dianthus carthusianorum* L., *Borago officinalis* L.
854. *Sanguisorba minor* Scop. *Pimpinella anisum* L., *Mentha spicata* L.
855. *Trifolium repens* L., *Alternanthera porrigens* (Jacq.) Kuntze, *Cuphea strigulosa* Kunth, *Iresine herbstii* Hook., *Smilax medica* Schltdl. & Cham.

Stress

856. *Lavandula angustifolia* Mill., *Rosmarinus officinalis* L., *Salvia rosmarinifolia* G. Don, *Eucalyptus globulus* Labill.

Susto

857. *Mauria heterophylla* Kunth, *Phytolacca bogotensis* Kunth, *Juglans neotropica* Diels, *Artemisia absinthium* L., *Equisetum giganteum* L.
858. *Apium graveolens* L., *Petroselinum crispum* (Mill.) Fuss
859. *Arracacia xanthorrhiza* Bancr., *Zea mays* L., *Eucalyptus globulus* Labill.
860. *Daucus montanus* Humb. & Bonpl. ex Spreng., *Achyrocline alata* (Kunth) DC., *Siphocampylus cutervensis* Zahlbr., *Alternanthera brasiliana* (L.) Kuntze, *Phytolacca bogotensis* Kunth
861. *Mandevilla* cf. *trianae* Woodson, *Daucus montanus* Humb. & Bonpl. ex Spreng., *Baccharis latifolia* (Ruiz & Pav.) Pers., *Acalypha mandonii* Müll. Arg., *Trixis cacalioides* (Kunth) D. Don, *Achyrocline alata* (Kunth) DC., *Centropogon reticulatus* Drake*, Fuchsia ayavacensis* Kunth, *Siphocampylus cutervensis* Zahlbr., *Monactis flaverioides* Kunth
862. *Oreopanax eriocephalus* Harms, *Nerium oleander* L., *Myrica pubescens* Humb. & Bonpl. ex Willd., *Oreocallis grandiflora* (Lam.) R. Br., *Salvia rosmarinifolia* G. Don, *Matricaria recutita* L., *Rosmarinus officinalis* L., *Juglans neotropica* Diels.
863. *Sarcostemma clausum* (Jacq.) Schult., *Spartium junceum* L., *Polylepis racemosa* Ruiz & Pav., *Solanum tuberosum* L., *Eucalyptus globulus* Labill.
864. *Achyrocline alata* (Kunth) DC., *Siparuna muricata* (Ruiz & Pav.) A. DC., *Artemisia absinthium* L., *Mauria heterophylla* Kunth
865. *Artemisia absinthium* L., *Siparuna muricata* (Ruiz & Pav.) A. DC., *Trixis cacalioides* (Kunth) D. Don, *Tagetes erecta* L., *Monactis flaverioides* Kunth
866. *Artemisia absinthium* L., *Siparuna muricata* (Ruiz & Pav.) A. DC., *Ruta graveolens* L.
867. *Ferreyranthus verbascifolius* (Kunth) H. Rob. & Brettell*, Cantua quercifolia* Juss., *Matricaria recutita* L.,*Trixis cacalioides* (Kunth) D. Don, *Ruta graveolens* L. *Vallea stipularis* L. f., *Centropogon reticulatus* Drake*, Fuchsia ayavacensis* Kunth, *Siphocampylus cutervensis* Zahlbr., *Salvia ayavacensis* Kunth*, Matricaria frigidum* (H.B.K.) Kunth, *Ambrosia peruviana* Willd.
868. *Monactis flaverioides* Kunth, *Artemisia absinthium* L., *Salvia discolor* Kunth, *Ambrosia peruviana* Willd., *Miconia salicifolia* (Bonpl. ex Naudin) Naudin

869. *Munnozia lyrata* (A. Gray) H. Rob. & Brettell, *Monactis flaverioides* Kunth, *Siparuna muricata* (Ruiz & Pav.) A. DC., *Trixis cacalioides* (Kunth) D. Don, *Salvia tubiflora* Ruiz & Pav., *Achyrocline alata* (Kunth) DC., *Aiouea dubia* (Kunth) Mez., *Nectandra reticulata* (Ruiz & Pav.) Mez.
870. *Plantago linearis* Kunth, *Plantago major* L., *Cordia alliodora* (Ruiz & Pav.) Okens, *Porophyllum ruderale* (Jacq.) Cass., *Rosmarinus officinalis* L., *Eucalyptus globulus* Labill., *Brugmansia arborea* (L.) Lagerh., *Brugmansia sanguinea* (Ruiz & Pav.) D. Don, *Spartium junceum* L., *Trixis cacalioides* (Kunth) D. Don
871. *Pseudogynoxys cordifolia* (Cass.) Cabrera, *Marrubium vulgare* L., *Rosmarinus officinalis* L., *Salvia rosmarinifolia* G. Don
872. *Tagetes erecta* L., *Origanum vulgare* L., *Zea mays* L., *Spartium junceum* L., *Ruta graveolens* L.
873. *Tagetes patula* L., *Artemisia absinthium* L., *Ruta graveolens* L., *Monactis flaverioides* Kunth, *Matricaria recutita* L.
874. *Trixis cacalioides* (Kunth) D. Don, *Siparuna muricata* (Ruiz & Pav.) A. DC., *Ruta graveolens* L., *Artemisia absinthium* L., *Achyrocline alata* (Kunth) DC., *Escallonia pendula* (Ruiz & Pav.) Pers.
875. *Salvia discolor* Kunth, *Ambrosia peruviana* Willd., *Miconia salicifolia* (Bonpl. ex Naudin) Naudin, *Cordia alliodora* (Ruiz & Pav.) Okens, *Siparuna muricata* (Ruiz & Pav.) A. DC., *Porophyllum ruderale* (Jacq.) Cass., *Ruta graveolens* L.
876. *Tillandsia cacticola* L.B. Sm., *Rosmarinus officinalis* L., *Salvia rosmarinifolia* G. Don, *Bursera graveolens* (Kunth) Triana & Planch., *Lavandula angustifolia* Mill.
877. *Bursera graveolens* (Kunth) Triana & Planch., *Salvia rosmarinifolia* G. Don, *Rosmarinus officinalis* L.
878. *Siphocampylus cutervensis* Zahlbr., *Fuchsia ayavacensis* Kunth, *Huperzia hohenackeri* (Herter) Holub
879. *Sambucus peruviana* Kunth, *Juglans neotropica* Diels, *Monactis flaverioides* Kunth, *Matricaria recutita* L. .
880. *Myroxylon balsamum* (L.) Harms, *Achyrocline alata* (Kunth) DC., *Aiouea dubia* (Kunth) Mez., *Nectandra reticulata* (Ruiz & Pav.) Mez., *Couepia* sp., *Trichilia* sp., *Strychnos* sp., *Myristica fragrans* Houtt., *Cymbopogon citratus* (DC.) Stapf., *Aloysia triphylla* (L'Hér.) Britton, *Nicotiana tabacum* L., *Allium sativum* L, *Laccopetalum giganteum* (Wedd.) Ulbr., *Eucalyptus globulus* Labill.
881. *Pelargonium odoratissimum* (L.) L'Hér., *Achyrocline alata* (Kunth) DC., *Aiouea dubia* (Kunth) Mez., *Nectandra reticulata* (Ruiz & Pav.) Mez., *Eucalyptus globulus* Labill., *Marrubium vulgare* L., *Zea mays* L., *Spartium junceum* L.
882. *Lepechinia meyenii* (Walp.) Epling, *Trixis cacalioides* (Kunth) D. Don, *Artemisia absinthium* L., *Juglans neotropica* Diels
883. *Marrubium vulgare* L., *Eucalyptus globulus* Labill., *Achyrocline alata* (Kunth) DC., *Aiouea dubia* (Kunth) Mez., *Nectandra reticulata* (Ruiz & Pav.) Mez., *Cajanus cajan* (L.) Huth, *Polylepis racemosa* Ruiz & Pav.,
884. *Salvia ayavacensis* Kunth, *Senecio genisianus* Cuatrec., *Phytolacca bogotensis* Kunth, *Siphocampylus cutervensis* Zahlbr., *Daucus montanus* Humb. & Bonpl. ex Spreng., *Salvia rosmarinifolia* G. Don
885. *Salvia discolor* Kunth, *Achyrocline alata* (Kunth) DC., *Porophyllum ruderale* (Jacq.) Cass., *Cordia alliodora* (Ruiz & Pav.) Oken
886. *Salvia rosmarinifolia* G. Don, *Bursera graveolens* (Kunth) Triana & Planch., *Eucalyptus globulus* Labill.
887. *Salvia rosmarinifolia* G. Don, *Polygala paniculata* L., *Equisetum bogotense* Kunth, *Equisetum giganteum* L.
888. *Aiouea dubia* (Kunth) Mez., *Achyrocline alata* (Kunth) DC., *Nectandra reticulata* (Ruiz & Pav.) Mez., *Trichilia* sp., *Strychnos* sp.
889. *Malva sylvestris* L., *Melissa officinalis* L., *Sanguisorba minor* Scop., *Origanum majorana* L., *Viola tricolor* L., *Lathyrus odoratus* L., *Cymbopogon citratus* (DC.) Stapf., *Aloysia triphylla* (L'Hér.) Britton
890. *Siparuna muricata* (Ruiz & Pav.) A. DC., *Monactis flaverioides* Kunth, *Achyrocline alata* (Kunth) DC., *Aiouea dubia* (Kunth) Mez., *Nectandra reticulata* (Ruiz & Pav.) Mez., *Rosmarinus officinalis* L., *Salvia rosmarinifolia* G. Don, *Ruta graveolens* L., *Escallonia pendula* (Ruiz & Pav.) Pers., *Bursera graveolens* (Kunth) Triana & Planch.
891. *Myrica pubescens* Humb. & Bonpl. ex Willd., *Sambucus peruviana* Kunth, *Juglans neotropica* Diels,
892. *Cestrum auriculatum* L'Hér., *Polylepis racemosa* Ruiz & Pav., *Eucalyptus globulus* Labill., *Salvia rosmarinifolia* G. Don
893. *Fuchsia ayavacensis* Kunth, *Sambucus peruviana* Kunth, *Juglans neotropica* Diels, *Lepechinia meyenii* (Walp.) Epling, *Salvia officinalis* L., *Salvia cuspidata* Ruiz & Pav., *Salvia sagittata* Ruiz. & Pav., *Siparuna muricata* (Ruiz & Pav.) A. DC.
894. *Phytolacca bogotensis* Kunth, *Siparuna muricata* (Ruiz & Pav.) A. DC., *Artemisia absinthium* L., *Sambucus peruviana* Kunth, *Mauria heterophylla* Kunth, *Equisetum giganteum* L.
895. *Plantago sericea* Ruiz & Pav., *Melissa officinalis* L., *Origanum majorana* L.
896. *Cantua buxifolia* Juss. ex Lam., *Eucalyptus globulus* Labill., *Marrubium vulgare* L., *Zea mays* L.
897. *Oreocallis grandiflora* (Lam.) R. Br., *Vallea stipularis* L. f., *Daucus montanus* Humb. & Bonpl. ex Spreng., *Siphocampylus cutervensis* Zahlbr., *Bejaria aestuans* Mutis ex L., *Oxalis bulbigera* R. Knuth
898. *Ruta graveolens* L., *Gentianella bicolor* (Wedd.) J.S. Pringle, *Lepechinia meyenii* (Walp.) Epling, *Salvia officinalis* L., *Salvia cuspidata* Ruiz & Pav., *Salvia sagittata* Ruiz. & Pav., *Origanum vulgare* L., *Schinus molle* L., *Eucalyptus globulus* Labill., *Ambrosia peruviana* Willd., *Artemisia absinthium* L., *Adiantum concinnum* Humb. & Bonpl. ex Willd.
899. *Escallonia pendula* (Ruiz & Pav.) Pers., *Oreocallis grandiflora* (Lam.) R. Br., *Vallea stipularis* L. f., *Achyrocline alata* (Kunth) DC., *Artemisia absinthium* L.
900. *Brugmansia candida* Pers., *Brugmansia arborea* (L.) Lagerh., *Brugmansia sanguinea* (Ruiz & Pav.) D. Don, *Porophyllum ruderale* (Jacq.) Cass., *Zea mays* L., *Gaultheria reticulata* Kunth
901. *Cestrum auriculatum* L'Hér., *Polylepis racemosa* Ruiz & Pav., *Eucalyptus globulus* Labill., *Salvia rosmarinifolia* G. Don
902. *Lycopersicon hirsutum* Dunal, *Tagetes erecta* L., *Daucus montanus* Humb. & Bonpl. ex Spreng., *Mentha x piperita* L., *Equisetum giganteum* L.,
903. *Solanum americanum* Mill., *Melissa officinalis* L., *Origanum majorana* L.
904. *Solanum americanum* Mill., *Monactis flaverioides* Kunth, *Porophyllum ruderale* (Jacq.) Cass., *Rosmarinus officinalis* L., *Salvia rosmarinifolia* G. Don, *Salvia tubiflora* Ruiz & Pav., *Couepia* sp., *Achyrocline alata* (Kunth) DC., *Aiouea dubia* (Kunth) Mez., *Nectandra reticulata* (Ruiz & Pav.) Mez., *Strychnos* sp., *Tagetes erecta* L., *Ruta graveolens* L.
905. *Solanum* sp., *Senecio genisianus* Cuatrec., *Daucus montanus* Humb. & Bonpl. ex Spreng.
906. *Celtis loxensis* C.C. Berg, *Siphocampylus cutervensis* Zahlbr., *Hydrocotyle bonariensis* Lam
907. *Urtica urens* L., *Artemisia absinthium* L., *Salvia discolor* Kunth

Swelling

908. *Fuchsia ayavacensis* Kunth, *Sambucus peruviana* Kunth, *Juglans neotropica* Diels, *Salvia officinalis* L., *Lepechinia meyenii* (Walp.) Epling, *Salvia cuspidata* Ruiz & Pav., *Salvia sagittata* Ruiz. & Pav., *Siparuna muricata* (Ruiz & Pav.) A. DC.

Tachycardia

909. *Melissa officinalis* L., *Sanguisorba minor* Scop., *Cymbopogon citratus* (DC.) Stapf., *Aloysia triphylla* (L'Hér.) Britton, *Origanum majorana* L., *Tillandsia cacticola* L.B. Sm., *Tillandsia multiflora* Benth. var. *decipiens* (André) L.B. Sm., *Echeveria peruviana* Meyen, *Rosmarinus officinalis* L., *Salvia rosmarinifolia* G. Don, *Dianthus caryophyllus* L., *Tagetes erecta* L., *Peperomia inaequalifolia* Ruiz & Pav., *Matricaria frigidum* (H.B.K.) Kunth, *Matricaria recutita* L., *Ambrosia peruviana* Willd., *Origanum majorana* L., *Sanguisorba minor* Scop., *Citrus sinensis* (L.) Osbeck
910. *Malva sylvestris* L., *Melissa officinalis* L., *Sanguisorba minor* Scop., *Origanum majorana* L., *Viola tricolor* L., *Lathyrus odoratus* L., *Cymbopogon citratus* (DC.) Stapf., *Aloysia triphylla* (L'Hér.) Britton

Tapeworm

911. *Mentha spicata* L., *Pimpinella anisum* L.

Tension

912. *Ipomoea pauciflora* M. Martens & Galeotti, *Corynaea crassa* Hook. f., *Laccopetalum giganteum* (Wedd.) Ulbr., *Brosimum rubescens* Taub., *Celtis loxensis* C.C. Berg, *Heisteria acuminata* (Humb. & Bonpl.) Engl., *Cinchona officinalis* L., *Eustephia coccinea* Cav.

Throat

913. *Caesalpinia spinosa* (Molina) Kuntze, *Rosmarinus officinalis* L., *Salvia rosmarinifolia* G. Don, *Erythroxylum coca* Lam, *Croton draconoides* Muell.-Arg., *Croton lechleri* Müll. Arg.
914. *Rubus robustus* C. Presl., *Salvia discolor* Kunth, *Ambrosia peruviana* Willd., *Miconia salicifolia* (Bonpl. ex Naudin) Naudin

Tranquility

915. *Verbena litoralis* Kunth, *Piper aduncum* L., *Monactis flaverioides* Kunth, *Malva sylvestris* L., *Alcea rosea* L., *Pelargonium odoratissimum* (L.) L'Hér., *Malva parviflora* L., *Matricaria frigidum* (H.B.K.) Kunth, *Matricaria recutita* L., *Ambrosia peruviana* Willd.

Tuberculosis

916. *Schinus molle* L., *Eucalyptus globulus* Labill., *Ruta graveolens* L., *Dodonaea viscosa Jacq.*, *Tilia platyphyllos* Scop.
917. *Piper aduncum* L., *Lepechinia meyenii* (Walp.) Epling, *Perezia multiflora* (Bonpl.) Less., *Oritrophium peruvianum* (Lam.) Cuatrec., *Senecio canescens* (Bonpl.) Cuatrec., *Borago officinalis* L., *Eupatorium gayanum* Wedd.

Tumors

918. *Arctium lappa* L., *Bejaria aestuans* Mutis ex L., *Cenchrus echinatus* L., *Tribulus terrestris* L., *Baccharis genistelloides* (Lam.) Pers., *Bidens pilosa* L., *Centropogon* cf. *rufus* E. Wimm.
919. *Plantago major* L., *Piper aduncum* L., *Artemisia absinthium* L., *Lepechinia meyenii* (Walp.) Epling, *Senna occidentalis* (L.) Link
920. *Cenchrus echinatus* L., *Bidens pilosa* L., *Arctium lappa* L., *Centropogon* cf. *rufus* E. Wimm.
921. *Tribulus terrestris* L., *Bidens pilosa* L., *Arctium lappa* L., *Centropogon* cf. *rufus* E. Wimm.

Twisted Bones

922. *Alternanthera brasiliana* (L.) Kuntze, *Gaultheria erecta* Vent., *Oritrophium peruvianum* (Lam.) Cuatrec., *Laccopetalum giganteum* (Wedd.) Ulbr., *Senecio tephrosioides* Turcz., *Clerodendron* sp.

Typhoid

923. *Cestrum auriculatum* L'Hér., *Polylepis racemosa* Ruiz & Pav., *Eucalyptus globulus* Labill., *Salvia rosmarinifolia* G. Don

Ulcers

924. *Eustephia coccinea* Cav., *Buddleja utilis* Kraenzl. *Bejaria aestuans* Mutis ex L., *Pelargonium odoratissimum* (L.) L'Hér.
925. *Gaultheria reticulata* Kunth, *Brugmansia candida* Pers., *Brugmansia arborea* (L.) Lagerh., *Brugmansia sanguinea* (Ruiz & Pav.) D. Don, *Brugmansia candida* Pers.
926. *Trifolium repens* L., *Alternanthera porrigens* (Jacq.) Kuntze, *Iresine herbstii* Hook., *Smilax medica* Schltdl. & Cham.
927. *Polypodium crassifolium* L., *Desmodium molliculum (*Kunth*)* DC., *Bidens pilosa* L., *Equisetum bogotense* Kunth, *Equisetum giganteum* L.
928. *Citrus limon* (L.) Burm. f., *Matricaria frigidum* (H.B.K.) Kunth, *Matricaria recutita* L., *Ambrosia peruviana* Willd., *Melissa officinalis* L., *Sanguisorba minor* Scop., *Gentianella crassicaulis* J.S. Pringle, *Dianthus caryophyllus* L., *Tagetes erecta* L.
929. *Mauria heterophylla* Kunth, *Equisetum bogotense* Kunth, *Equisetum giganteum* L., *Verbena litoralis* Kunth, *Bidens pilosa* L.,

Urinary Infections

930. *Bixa orellana* L., *Uncaria tomentosa* (Willd. ex Roem. & Schult.) DC., *Mimosa nothacacia* Barneby
931. *Tiquilia paronychioides* (Phil.) A.T. Richardson, *Monactis flaverioides* Kunth, *Malva sylvestris* L., *Pelargonium odoratissimum* (L.) L'Hér., *Malva parviflora* L., *Zea mays* L., *Equisetum bogotense* Kunth, *Equisetum giganteum* L., *Pilea microphylla* (L.) Liebm., *Buddleja utilis* Kraenzl., *Bejaria aestuans* Mutis ex L., *Cenchrus echinatus* L., *Tribulus terrestris* L., *Baccharis genistelloides* (Lam.) Pers., *Bidens pilosa* L., *Rorippa nasturtium-aquaticum* (L.) Hayek, *Typha angustifolia* L., *Bixa orellana* L., *Alternanthera porrigens* (Jacq.) Kuntze, *Cuphea strigulosa* Kunth, *Eustephia coccinea* Cav.

Urinary Problems

932. *Arctium lappa* L., *Bejaria aestuans* Mutis ex L., *Cenchrus echinatus* L., *Tribulus terrestris* L., *Baccharis genistelloides* (Lam.) Pers., *Bidens pilosa* L., *Centropogon* cf. *rufus* E. Wimm.

933. *Capsella bursa-pastoris* (L.) Medik., *Mauria heterophylla* Kunth, *Verbena litoralis* Kunth, *Zea mays* L., *Buddleja utilis* Kraenzl., *Tiquilia paronychioides* (Phil.) A.T. Richardson, *Geranium ayavacense* Willd. ex Kunth, *Geranium sessiliflorum* Cav., *Gentianella bicolor* (Wedd.) J.S. Pringle, *Equisetum bogotense* Kunth, *Equisetum giganteum* L.,
934. *Rorippa nasturtium-aquaticum* (L.) Hayek, *Monactis flaverioides* Kunth, *Malva sylvestris* L., *Alcea rosea* L., *Pelargonium odoratissimum* (L.) L'Hér., *Malva parviflora* L., *Desmodium molliculum (*Kunth*)* DC., *Eugenia obtusifolia* Cambess., *Bidens pilosa* L., *Mauria heterophylla* Kunth, *Iresine diffusa* Humb. & Bonpl. ex Willd., *Plantago sericea* Ruiz & Pav. subsp. *sericans* (Pilg.) Rahn, *Tiquilia paronychioides* (Phil.) A.T. Richardson, *Bejaria aestuans* Mutis ex L.,
935. *Centropogon* cf. *rufus* E. Wimm., *Bejaria aestuans* Mutis ex L., *Cenchrus echinatus* L., *Tribulus terrestris* L., *Baccharis genistelloides* (Lam.) Pers., *Bidens pilosa* L., *Arctium lappa* L.
936. *Equisetum bogotense* Kunth, *Cordia lutea* Lam, *Alternanthera porrigens* (Jacq.) Kuntze, *Cuphea strigulosa* Kunth, *Typha angustifolia* L., *Bixa orellana* L., *Smilax medica* Schltdl. & Cham.
937. *Equisetum bogotense* Kunth, *Desmodium molliculum (*Kunth*)* DC., *Mauria heterophylla* Kunth
938. *Geranium ayavacense* Willd. ex Kunth, *Geranium sessiliflorum* Cav., *Mauria heterophylla* Kunth, *Equisetum bogotense* Kunth, *Equisetum giganteum* L., *Verbena litoralis* Kunth, *Eugenia obtusifolia* Cambess., *Bidens pilosa* L., *Cynodon dactylon* (L.) Pers.

Uterus

939. *Bejaria aestuans* Mutis ex L., *Buddleja utilis* Kraenzl., *Dioscorea trifida* L. f., *Tiquilia paronychioides* (Phil.) A.T. Richardson, *Ilex guayusa* Loes., *Geranium ayavacense* Willd. ex Kunth, *Geranium sessiliflorum* Cav., *Monactis flaverioides* Kunth, *Malva sylvestris* L., *Pelargonium odoratissimum* (L.) L'Hér., *Malva parviflora* L., *Bidens pilosa* L., *Verbena litoralis* Kunth, *Plantago linearis* Kunth, *Plantago major* L., *Equisetum bogotense* Kunth, *Equisetum giganteum* L., *Muehlenbeckia tamnifolia* (Kunth) Meisn., *Smilax medica* Schltdl. & Cham., *Oreocallis grandiflora* (Lam.) R. Br., *Cinchona officinalis* L.
940. *Gentianella brunneotincta* (Gilg) Pringle, *Phyllactis rigida* (Ruiz & Pav.) Pers.
941. *Cynodon dactylon* (L.) Pers., *Equisetum bogotense* Kunth, *Equisetum giganteum* L., *Verbena litoralis* Kunth, *Bidens pilosa* L., *Monactis flaverioides* Kunth, *Malva sylvestris* L., *Pelargonium odoratissimum* (L.) L'Hér., *Malva parviflora* L., *Buddleja utilis* Kraenzl., *Smilax medica* Schltdl. & Cham., *Cuphea strigulosa* Kunth

Uterus (cancer)

942. *Dioscorea trifida* L. f., *Buddleja utilis* Kraenzl., *Bejaria aestuans* Mutis ex L., *Paranephelius uniflorus* Poepp., *Rorippa nasturtium-aquaticum* (L.) Hayek
943. *Dioscorea trifida* L. f., *Piper aduncum* L., *Monactis flaverioides* Kunth, *Malva sylvestris* L., *Pelargonium odoratissimum* (L.) L'Hér., *Malva parviflora* L., *Caesalpinia spinosa* (Molina) Kuntze

Vaginal cleansing

944. *Monactis flaverioides* Kunth, *Artemisia absinthium* L., *Salvia discolor* Kunth, *Ambrosia peruviana* Willd., *Miconia salicifolia* (Bonpl. ex Naudin) Naudin
945. *Dioscorea trifida* L. f., *Piper aduncum* L., *Monactis flaverioides* Kunth, *Malva sylvestris* L., *Pelargonium odoratissimum* (L.) L'Hér., *Malva parviflora* L., *Caesalpinia spinosa* (Molina) Kuntze
946. *Plantago major* L., *Piper aduncum* L., *Artemisia absinthium* L., *Lepechinia meyenii* (Walp.) Epling, *Senna occidentalis* (L.) Link
947. *Urtica urens* L., *Artemisia absinthium* L., *Salvia discolor* Kunth, *Ambrosia peruviana* Willd., *Miconia salicifolia* (Bonpl. ex Naudin) Naudin

Vaginal Discharge

948. *Tristerix longebracteatus* (Desr.) Barlow & Wiens, *Uncaria tomentosa* (Willd. ex Roem. & Schult.) DC., *Mimosa nothacacia* Barneby, *Ephedra americana* Humb. & Bonpl. ex Willd.
949. *Plantago sericea* Ruiz & Pav. var. *lanuginosa* Griseb., *Gentianella brunneotincta* (Gilg) Pringle

Vomiting

950. *Ruta graveolens* L., *Gentianella dianthoides* (Kunth) Fabris ex J.S. Pringle, *Gentianella bicolor* (Wedd.) J.S. Pringle, *Lepechinia meyenii* (Walp.) Epling, *Salvia officinalis* L., *Salvia cuspidata* Ruiz & Pav., *Salvia sagittata* Ruiz. & Pav., *Origanum vulgare* L., *Schinus molle* L., *Eucalyptus globulus* Labill., *Ambrosia peruviana* Willd., *Artemisia absinthium* L., *Adiantum concinnum* Humb. & Bonpl. ex Willd.

Warts

951. *Alternanthera porrigens* (Jacq.) Kuntze, *Schkuhria pinnata* (Lam.) Kuntze ex Thell., *Polygala paniculata* L., *Alternanthera halimifolia* (Lam.) Standl. ex Pittier, *Alternanthera brasiliana* (L.) Kuntze, *Iresine herbstii* Hook., *Cuphea strigulosa* Kunth, *Adiantum concinnum* Humb. & Bonpl. ex Willd., *Cuphea strigulosa* Kunth, *Smilax medica* Schltdl. & Cham., *Rubus robustus* C. Presl.

Wounds

952. *Mauria heterophylla* Kunth, *Equisetum bogotense* Kunth, *Equisetum giganteum* L., *Verbena litoralis* Kunth, *Bidens pilosa* L.,
953. *Senecio genisianus* Cuatrec., *Escallonia pendula* (Ruiz & Pav.) Pers., *Oreocallis grandiflora* (Lam.) R. Br., *Alternanthera brasiliana* (L.) Kuntze
954. *Salvia ayavacensis* Kunth*, Cestrum auriculatum* L'Hér.
955. *Equisetum bogotense* Kunth, *Cordia lutea* Lam, *Alternanthera porrigens* (Jacq.) Kuntze, *Cuphea strigulosa* Kunth, *Typha angustifolia* L., *Bixa orellana* L., *Smilax medica* Schltdl. & Cham.
956. *Equisetum bogotense* Kunth, *Desmodium molliculum (*Kunth*)* DC., *Mauria heterophylla* Kunth
957. *Equisetum giganteum* L., *Mauria heterophylla* Kunth, *Verbena litoralis* Kunth, *Cestrum auriculatum* L'Hér., *Plantago linearis* Kunth, *Plantago major* L.

958. *Gaultheria reticulata* Kunth, *Brugmansia candida* Pers., *Brugmansia arborea* (L.) Lagerh., *Brugmansia sanguinea* (Ruiz & Pav.) D. Don
959. *Caesalpinia spinosa* (Molina) Kuntze, *Nerium oleander* L., *Myrica pubescens* Humb. & Bonpl. ex Willd., *Monactis flaverioides* Kunth, *Artemisia absinthium* L., *Achyrocline alata* (Kunth) DC., *Aiouea dubia* (Kunth) Mez., *Nectandra reticulata* (Ruiz & Pav.) Mez.
960. *Desmodium molliculum* (Kunth) DC., *Plantago linearis* Kunth, *Plantago major* L., *Piper aduncum* L.
961. *Juglans neotropica* Diels, *Siparuna muricata* (Ruiz & Pav.) A. DC., *Oreocallis grandiflora* (Lam.) R. Br., *Achyrocline alata* (Kunth) DC., *Sambucus peruviana* Kunth
962. *Lepechinia meyenii* (Walp.) Epling, *Rosmarinus officinalis* L., *Salvia rosmarinifolia* G. Don, *Plantago linearis* Kunth, *Plantago major* L.
963. *Piper aduncum* L., *Lepechinia meyenii* (Walp.) Epling, *Eucalyptus globulus* Labill., *Nerium oleander* L., *Myrica pubescens* Humb. & Bonpl. ex Willd., *Verbena litoralis* Kunth, *Ambrosia peruviana* Willd.
964. *Plantago linearis* Kunth, *Piper aduncum* L.
965. *Plantago linearis* Kunth, *Equisetum bogotense* Kunth, *Equisetum giganteum* L., *Mauria heterophylla* Kunth, *Eugenia obtusifolia* Cambess., *Cynodon dactylon* (L.) Pers., *Buddleja utilis* Kraenzl.
966. *Plantago major* L., *Piper aduncum* L., *Lepechinia meyenii* (Walp.) Epling, *Senna occidentalis* (L.) Link
967. *Prunus serotina* Ehrh. subsp. *capuli* (Cav.) McVaugh, *Verbena litoralis* Kunth, *Equisetum bogotense* Kunth, *Equisetum giganteum* L.
968. *Uncaria tomentosa* (Willd. ex Roem. & Schult.) DC., *Phyllanthus niruri* L., *Phyllanthus stipulatus* (Raf.) G.L.Webster, *Linum sativum* L., *Linum usitatissimum* L., *Peumus boldus* Molina, *Cordia lutea* Lam, *Capsella bursa-pastoris* (L.) Medik.
969. *Cestrum auriculatum* L'Hér., *Equisetum bogotense* Kunth, *Equisetum giganteum* L.
970. *Verbena litoralis* Kunth, *Sonchus oleraceus* L., *Alternanthera halimifolia* (Lam.) Standl. ex Pittier, *Alternanthera porrigens* (Jacq.) Kuntze, *Alternanthera brasiliana* (L.) Kuntze, *Portulaca oleracea* L. subsp. *tuberculata* Danin & H.G. Baker, *Portulaca villosa* Cham.
971. *Matricaria recutita* L., *Nerium oleander* L., *Myrica pubescens* Humb. & Bonpl. ex Willd., *Plantago linearis* Kunth, *Plantago major* L.

Yellow Fever

972. *Sambucus peruviana* Kunth, *Ricinus communis* L.
973. *Sambucus peruviana* Kunth, *Juglans neotropica* Diels, *Monactis flaverioides* Kunth, *Matricaria recutita* L.
974. *Phytolacca bogotensis* Kunth, *Siparuna muricata* (Ruiz & Pav.) A. DC., *Trixis cacalioides* (Kunth) D. Don, *Artemisia absinthium* L., *Sambucus peruviana* Kunth, *Mauria heterophylla* Kunth, *Equisetum giganteum* L.
