# Supplementary material for: Herbal mixtures in traditional medicine in Northern Peru
Source: J Ethnobiol Ethnomed. 2010 Mar 14;6:10. doi: 10.1186/1746-4269-6-10 (PMC2848642; doi:10.1186/1746-4269-6-10)
Supplement: Additional file 2 — Scientific plant names and collection numbers. [file 1746-4269-6-10-S2.PDF]

Additional file 2. Scientific plant names and voucher information

Scientific name	Family	Voucher #
Aphelandra cirsioides Lindau	Acanthaceae	ISA40
Adiantum concinnum Humb. & Bonpl. Ex Willd.	Adiantaceae	VFCHL29, TRUBH17, RBU/PL265, JULS149, ACR91
Tetragonia crystallina L'Hér.	Aizoaceae	ISA133, RBU/PL360, ISA26, GER131
Alternanthera brasiliana (L.) Kuntze	Amaranthaceae	RBU/PL275, JULS11, EHCHL78, ISA83
Alternanthera halimifolia (Lam.) Standl. Ex Pittier	Amaranthaceae	JULS85, JULS243, GER23
Alternanthera porrigens (Jacq.) Kuntze	Amaranthaceae	EHCHL142, ISA56, RBU/PL301, RBU/PL324, EHCHL93, GER117, ACR149, ACR395
Alternanthera villosa Kunth	Amaranthaceae	GER63, ACR103
Iresine diffusa Humb. & Bonpl. ex Willd.	Amaranthaceae	JULS75, ISA62
Iresine herbstii Hook.	Amaranthaceae	ISA42, EHCHL114, ISA121
Eustephia coccinea Cav.	Amaryllidaceae	RBU/PL313, GER71, EHCHL68
Loxopterygium huasango Spruce ex Engl.	Anacardiaceae	GER109
Mangifera indica L.	Anacardiaceae	GER49
Mauria heterophylla Kunth	Anacardiaceae	ISA24, JULS17, EHCHL83
Schinus molle L.	Anacardiaceae	EHCHL123, JULS196, GER13
Annona muricata L.	Annonaceae	GER2, EHCHL81, ACR81
Apium graveolens L.	Apiaceae	JULS21, ISA79, ISA116, EHCHL106, ACR22, KMM439
Arracacia xanthorrhiza Bancr.	Apiaceae	JULS278
Daucus carota L.	Apiaceae	
Daucus montanus Humb. & Bonpl. ex Spreng.	Apiaceae	ISA33, ISA38, ISA68, JULS271, GER172
Foeniculum vulgare Mill.	Apiaceae	EHCHL23, JULS101, JULS166, KMM409, ACR82
Hydrocotyle bonariensis Lam.	Apiaceae	ISA8
Niphogeton dissecta (Benth.) J.F. Macbr.	Apiaceae	EHCHL166, RBU/PL338, AKT1196
Petroselinum crispum (Mill.) Fuss	Apiaceae	ISA80, EHCHL31, ISA117, RBU/PL278, JULS225
Pimpinella anisum L.	Apiaceae	EHCHL137, TRUBH21, GER213
Mandevilla cf. *trianae* Woodson	Apocynaceae	ISA14, ISA13
Nerium oleander L.	Apocynaceae	JULS65, GER45, ACR34
Thevetia peruviana (Pers.) K. Schum.	Apocynaceae	EHCHL162, TRUVan/Erica19, JULS187, EHCHL174, GER225, KMM416
Vallesia glabra (Cav.) Link.	Apocynaceae	GER26
Ilex guayusa Loes.	Aquifoliaceae	EHCHL130, JULS160, AKT1179, KMM513
Oreopanax eriocephalus Harms	Araliaceae	EHCHL36, JULS39, RBU/PL270, ISA71, GER221, ACR59, KMM445
Cocos nucifera L.	Arecaceae	JULS145
Aristolochia ruiziana (Klotzsch) Duch.	Aristolochiaceae	GER107
Sarcostemma clausum (Jacq.) Schult.	Asclepiadaceae	JULS121, GER43
Acanthoxanthium spinosum (L.) Fourr.	Asteraceae	JULS176, EHCHL32, AKT1127
Achyrocline alata (Kunth) DC.	Asteraceae	ISA85, ISA109, AKT1199, ACR2
Acmella cf. *ciliata* (Kunth) Cass.	Asteraceae	GER7
Ambrosia peruviana Willd.	Asteraceae	JULS108, TRUBH18, RBU/PL370, TRUBH15, JULS90, GER9, GER110, ACR11
Arctium lappa L.	Asteraceae	GER227
Arnica montana L.	Asteraceae	JULS106
Artemisia absinthium L.	Asteraceae	ISA66, RBU/PL363, GER146, AKT1099, KMM432
Baccharis genistelloides (Lam.) Pers.	Asteraceae	EHCHL101, TRUBH10, RBU/PL255, JULS34, VFCHL36, EHCHL92, AKT1144, ACR78, KMM421
Baccharis inidca L.	Asteraceae	JULS220
Baccharis latifolia (Ruiz & Pav.) Pers.	Asteraceae	ISA86, ISA115
Baccharis salicifolia (Ruiz & Pav.) Pers.	Asteraceae	TRUVan/Erica5, GER125, GER84
Baccharis vaccinioides Kunth	Asteraceae	GER188, TRU BH 28
Bidens pilosa L.	Asteraceae	JULS74, VFCHL25, EHCHL18, ISA127, GER1, ACR177, ACR58, KMM427, KMM552, KMM469
Chuquiraga weberbaueri Tovar	Asteraceae	JULS99, EHCHL131, ACR85
Cronquistianthus lavandulifolius (DC.) R.M. King & H. Rob.	Asteraceae	ISA5, JULS233, GER163

Diplostephium gynoxyoides Cuatrec.	Asteraceae	GER5
Diplostephium sagasteguii Cuat.	Asteraceae	GER61, RBU/PL336, ISA139, TRUBH24, GER58, KMM478, AKT1145, AKT1192, ACR124, ACR153
Eupatorium gayanum Wedd.	Asteraceae	RBU/PL276, EHCHL164
Eupatorium triplinerve Vahl.	Asteraceae	JULS134
Ferryranthus verbascifolius (Kunth) H. Rob. & Brettell	Asteraceae	ISA36, ISA9, ISA11
Loricaria ferruginea (Ruiz & Pav.) Wedd.	Asteraceae	GER108, RBU/PL339, TRUBH20, ISA136, TRU Van/Erica23, TRU Van/Erica10, ISA148(105a), ACR63, AKT1174
Matricaria frigidum (Kunth) Kunth	Asteraceae	JULS22, EHCHL1, TRUBH7
Matricaria recutita L.	Asteraceae	JULS192, RBU/PL306, ISA120, ISA76, GER145, AKT1175
Mikania leiostachya Benth.	Asteraceae	ISA12, ACR6
Monactis flaverioides Kunth	Asteraceae	EHCHL19, RBU/PL274, TRUVan/Erica7, ISA104, ISA72
Munnozia lyrata (A. Gray.) Rob. & Brett.	Asteraceae	EHCHL138, KMM519
Oritrophium peruvianum (Lam.) Cautrec.	Asteraceae	JULS58, EHCHL126, TRUBH29, TRUBH26, ISA96, TRUVan/Erica2, GER166
Paranephelius uniflorus Poepp.	Asteraceae	EHCHL133, JULS125
Perezia multiflora (Bonpl.) Less.	Asteraceae	RBU/PL323, JULS16, EHCHL52, GER160, AKT1153, ACR54, KMM535
Picrosia longifolia D.Don.	Asteraceae	EHCHL116, JULS6, GER21, ACR29, ACR39, KMM436, KMM540
Porophyllum ruderale (Jacq.) Cass.	Asteraceae	ISA73, GER89, JULS180, ACR49, KMM515
Pseudogynoxys cordifolia (Cass.) Cabrera	Asteraceae	JULS294, AKT1168
Schkuhria pinnata (Lam.) Kuntze ex Thell.	Asteraceae	RBU/PL266, JULS42, VFCHL27, GER228, ACR17
Senecio canescens (Bonpl.) Cuatrec.	Asteraceae	TRUBH8, RBU/PL322, EHCHL104, EHCHL24, ISA108, TRUVan/Erica12, JULS14, GER158, AKT1098, AKT1156, ACR28, ACR44
Senecio chionogeton Wedd.	Asteraceae	GER60
Senecio genisianus Cuatrec.	Asteraceae	ISA16
Senecio pseudotites Griseb.	Asteraceae	GER217
Senecio tephrosioides Turcz.	Asteraceae	JULS12
Sonchus oleraceus L.	Asteraceae	RBU/PL368, EHCHL54, JULS64, VFCHL48, JULS248, ACR197, ACR40
Tagetes erecta L.	Asteraceae	EHCHL141, JULS156, GER112, ACT1104, KMM413
Tagetes filifolia Lag.	Asteraceae	RBU/PL283, JULS8, AKT1167, KMM524
Tagetes patula L.	Asteraceae	ISA89
Taraxacum officinale F.H. Wigg.	Asteraceae	RBU/PL252, JULS150, GER62, GER189
Tessaria integrifolia Ruiz & Pav.	Asteraceae	JULS71, GER12, kmm465
Trixis cacalioides (Kunth) D. Don	Asteraceae	ISA65, RBU/PL295
Werneria pygmaea Gillies ex Hook. & Arn.	Asteraceae	GER120
Werneria villosa A.Gray	Asteraceae	ISA101, GER124
Corynaea crassa Hook.f.	Balanophoraceae	JULS171, VFCHL52, AKT1169, KMM474, ACR130
Berberis buceronis J.F. Macbr.	Berberidaceae	JULS285, KMM573
Cydista aequinoctialis (L.) Miers	Bignoniaceae	ISA6
Jacaranda acutifolia Bonpl.	Bignoniaceae	RBU/PL326, ACR89
Bixa orellana L.	Bixaceae	ISA126, RBU/PL264, JULS9, EHCHL20, ISA44, JULS293, KMM429
Borago officinalis L.	Boraginaceae	ISA112, JULS24, RBU/PL300, EHCHL58, ACR9
Cordia alliodora (Ruiz & Pav.) Oken	Boraginaceae	ISA74, JULS281, KMM489
Cordia lutea Lam.	Boraginaceae	ISA125, EHCHL77, JULS62, GER10, AKT1114
Tiquilia paronychioides (Phil.) A.T. Richardson	Boraginaceae	JULS154, EHCHL107, ISA58, GER20, KMM406
Capsella bursa-pastoris (L.) Medik.	Brassicaceae	JULS7, VFCHL42, VFCHL12, RBU/PL257, EHCHL6, KMM451
Rorippa nasturtium-aquaticum (L.) Hayek	Brassicaceae	RBU/PL367, EHCHL25, JULS113, ACR94, AKT1163
Ananas comosus (L.) Merr.	Bromeliaceae	JULS230
Puya weberbaueri Mez.	Bromeliaceae	JULS290
Tillandsia cacticola L.B.Sm.	Bromeliaceae	VFCHL17, RBU/PL375, RBU/PL289, GER123, JULS307, ACR183

Tillandsia multiflora Benth. var. *decipiens* (André) L.B. Sm.	Bromeliaceae	EHCHL15, RBU/PL376
Bursera graveolens (Kunth) Triana & Planch.	Burseraceae	ISA143, JULS210, GER34
Echinopsis pachanoi (Britton & Rose) Friedrich & G.D. Rowley	Cactaceae	TRUBH36, JULS242, GER73, ACR95
Capparis crotonoides Kunth	Capparaceae	GER4, JULS250, KMM586
Lonicera japonica Thunb. ex Murray	Caprifoliaceae	JULS28
Sambucus peruviana Kunth	Caprifoliaceae	EHCHL140, RBU/PL291, VFCHL44, ISA131, ISA87, JULS246, EHCHL110, AKT1103, ACR66
Dianthus carthusianorum L.	Caryophyllaceae	
Dianthus caryophyllus L.	Caryophyllaceae	JULS18, JULS37, GER214, AKT1125, KMM580
Stellaria media (L.) Vill.	Caryophyllaceae	JULS262
Chenopodium quinoa Willd.	Chenopodiaceae	JULS236
Hedyosmum racemosum (Ruiz & Pav.) G. Don	Chloranthaceae	EHCHL147, RBU/PL377, KMM505
Couepia sp.	Chrysobalanaceae	EHCHL157, RBU/PL381, GER68, VFCHL54
Clethra castaneifolia Meisn.	Clethraceae	GER115, KMM549
Hypericum laricifolium Juss.	Clusiaceae	RBU/PL344, TRUBH1, TRUVan/Erica22, EHCHL145, GER128, GER126, AKT1172, KMM533
Hypericum silenoides Juss.	Clusiaceae	EHCHL85, AKT1154, ACR152, KMM387
Ipomoea batatas (L.) Lam.	Convolvulaceae	JULS120
Ipomoea pauciflora M.Martens & Galeotti	Convolvulaceae	GER222
Echeveria peruviana Meyen	Crassulaceae	EHCHL118, VFCHL33, JULS249, ACR169, AKT1113, AKT1165, KMM537
Cyclanthera pedata (L.) Schrad.	Cucurbitaceae	GER150
Sechium edule (Jacq.) Sw.	Cucurbitaceae	JULS119
Sicana odorifera (Vell.) Naudin	Cucurbitaceae	JULS247, ACR96
Sicyos baderoa Hook. & Arn.	Cucurbitaceae	GER99
Scirpus californicus Steud. subsp. *tatora* (Kunth) T. Koyama	Cyperacea	JULS111, GER169
Cyperus articulatus L.	Cyperaceae	JULS267
Oreobolus goeppingeri Suess.	Cyperaceae	EHCHL149, TRUVan/Erica17, EHCHL67, GER119, KMM493, AKT1184, ACR126
Dioscorea tambillensis R. Kunth	Dioscoreaceae	JULS283, GER140, KMM583
Dioscorea trifida L.f.	Dioscoreaceae	JULS214, EHCHL40, JULS212, GER142, JULS213, KMM503
Scabiosa atropurpurea L.	Dipsacaceae	JULS100, EHCHL111, RBU/PL372, ISA50, KMM512, ACR158
Vallea stipularis L.f.	Elaeocarpaceae	ISA32
Ephedra americana Humb. & Bonpl. ex Willd.	Ephedraceae	EHCHL150, JULS38, GER75, AKT1159, KMM511
Equisetum bogotense Kunth.	Equisetaceae	ISA52, TRUVan/Erica6, ISA107, ACR1
Equitsetum giganteum L.	Equisetaceae	VFCHL1, JULS5, TRUBH19, GER149
Bejaria aestuans Mutis ex L.	Ericaceae	VFCHL22, JULS50, EHCHL39, ISA114, ISA43, JULS234, GER121, AKT1109
Gaultheria erecta Vent.	Ericaceae	JULS288, JULS198, KMM472
Gaultheria reticulata Kunth	Ericaceae	EHCHL57, JULS259, RBU/PL293, EHCHL171, EHCHL51, GER81, GER241, GER57, KMM531
Erythroxylum coca Lam.	Erythroxylaceae	JULS144, GER201
Acalypha mandonii Müll. Arg.	Euphorbiaceae	RBU/PL294
Chamaesyce hypericifolia (L.) Millsp.	Euphorbiaceae	JULS67, GER41
Croton draconoides Müll. Arg.	Euphorbiaceae	JULS244a, GER100b
Croton lechleri Müll. Arg.	Euphorbiaceae	JULS244b, GER100a, KMM546
Phyllanthus niruri L.	Euphorbiaceae	JULS133c, EHCHL167b, GER152c, KMM517, AKT1151
Phyllanthus stipulatus (Raf.) G.L.Webster	Euphorbiaceae	JULS133b, EHCHL167a, GER152b
Ricinus communis L.	Euphorbiaceae	JULS83, GER19
Phyllanthus urinaria L.	Euphorbuiaceae	JULS133a, EHCHL167c, GER152a

Caesalpinia spinosa (Molina) Kuntze	Fabaceae	ISA55, EHCHL27, VFCHL21, JULS255, GER143, ACR111
Cajanus cajan (L.) Huth	Fabaceae	JULS136, ACR10
Desmodium molliculum (Kunth) D.C.	Fabaceae	JULS41, RBU/PL268, GER135, JULS44, EHCHL109, RBU/PL256, AKT1162, KMM392, ACR201
Desmodium triflorum (L.) DC	Fabaceae	GER122, RBU/PL347
Dolichos lablab L.	Fabaceae	GER235
Inga edulis Mart.	Fabaceae	JULS168, JULS304
Inga feuillei DC.	Fabaceae	GER17
Lathyrus odoratus L.	Fabaceae	VFCHL43
Leucaena leucocephala (Lam.) de Wit	Fabaceae	JULS104
Mimosa albida Humb. & Bonpl. ex Willd.	Fabaceae	JULS265b, AKT1118, ACR190, KMM560
Mimosa nothacacia Barneby	Fabaceae	JULS265, GER199
Myroxylon balsamum (L.) Harms.	Fabaceae	JULS287, RBU/PL382, EHCHL151, VFCHL46, GER91
Senna bicapsularis (L.) Roxb.	Fabaceae	JULS95
Senna occidentalis (L.) Link.	Fabaceae	JULS152
Spartium junceum L.	Fabaceae	EHCHL60, EHCHL 146, RBU/PL279, JULS239, KMM407
Trifolium repens L.	Fabaceae	ISA47, RBU/PL330, EHCHL30, AKT1194, KMM577
Zornia reticulata Sm.	Fabaceae	EHCHL122
Gentianella bicolor (Wedd.) J.S. Pringle	Gentianaceae	EHCHL14, VFCHL5, RBU/PL304, JULS167, AKT1147, AKT1207, ACR30, KMM526, ACR156
Gentianella brunneotincta (Gilg) Pringle	Gentianaceae	JULS282, KMM403
Gentianella crassicaulis J.S. Pringle	Gentianaceae	VFCHL7, ACR20, KMM4322
Gentianella dianthoides (Kunth) Fabris ex J.S. Pringle	Gentianaceae	RBU/PL253, RBU/PL320, JULS56, TRUVan/Erica21, EHCHL136, EHCHL61, ACR155, KMM576
Erodium cicutarium L'Hér. ex Aiton	Geraniaceae	ISA110, ISA54, AKT1171, ACR142, KMM578
Geranium ayavacense Willd. ex Kunth	Geraniaceae	JULS48, EHCHL63a, VFCHL6
Geranium sessiliflorum Cav.	Geraniaceae	JULS48a, EHCHL63 VFCHL6a, ACR38, KMM400
Pelargonium odoratissimum (L.) L'Hér.	Geraniaceae	TRUVan/Erica14, TRUBH6, EHCHL89, JULS188
Escallonia pendula (Ruiz & Pav.) Pers.	Saxifragaceae	ISA23, ISA63
Illicium verum Hook.f.	Magnoliaceae	JULS102
Isoetes andina Spruce ex Hook.	Isoetaceae	ISA100
Juglans neotropica Diels.	Juglandaceae	RBU/PL273, ISA67, EHCHL4, ISA123, AKT1111, KMM435
Hyptis sidifolia (L'Hér.) Briq.	Lamiaceae	EHCHL21, RBU/PL254, JULS222, JULS4, GER76, ACR69
Lavandula angustifolia Mill.	Lamiaceae	GER113, JULS177
Lepechinia meyenii (Walp.) Epling	Lamiaceae	RBU/PL303, VFCHL17, ISA91
Marrubium vulgare L.	Lamiaceae	JULS132, ACR25, ACR33
Melissa officinalis L.	Lamiaceae	JULS26, EHCHL2, RBU/PL260, VFCHL14
Mentha spicata L.	Lamiaceae	RBU/PL308, EHCHL74, RBU/PL267, JULS72, VFCHL3, JULS20, GER15, GER134, JULS20, AKT1101, AKT1166, KMM453, KMM500
Mentha x piperita L.	Lamiaceae	JULS29, ACR68
Minthostachys mollis (Kunth) Griseb.	Lamiaceae	EHCHL84, JULS200, AKT1142, ACR7, KMM417, KMM456
Ocimum basilicum L.	Lamiaceae	JULS54, EHCHL48, VFCHL13, RBU/PL284, TRUVan/Erica8, GER191, ACR32, KMM437, KMM428
Origanum majorana L.	Lamiaceae	EHCHL88, JULS19, RBU/PL317, GER165, ACR24, KMM471
Origanum vulgare L.	Lamiaceae	JULS205, GER114, KMM509
Otholobium glandulosum (L.) J.W. Grimes	Lamiaceae	EHCHL5, JULS40, KMM419, AKT1134
Rosmarinus officinalis L.	Lamiaceae	RBU/PL329, ISA78, TRUBH11, EHCHL3, JULS27, VFCHL2, ISA105, AKT1129, ACR16
Salvia ayavacensis Kunth	Lamiaceae	ISA37, ISA150(92a), RBU/PL290
Salvia cuspidata Ruiz & Pav.	Lamiaceae	RBU/PL315
Salvia discolor Kunth	Lamiaceae	SA93, ISA151(93a), ISA25
Salvia officinalis L.	Lamiaceae	JULS241
Salvia rosmarinifolia G. Don.	Lamiaceae	JULS49, ISA118, ISA77, EHCHL108, GER111
Salvia sagittata Ruiz. & Pav.,	Lamiaceae	RBU/PL318
Salvia tubiflora Ruiz & Pav.	Lamiaceae	EHCHL148, RBU/PL286, EHCHL49, GER70, GER175
Satureja pulchella (Kunth) Briq.	Lamiaceae	GER148, JULS43, KMM543
Stachys lanata Crantz	Lamiaceae	JULS13
Aiouea dubia (Kunth) Mez	Lauraceae	EHCHL152
Cinnamomum verum J. Presl	Lauraceae	JULS122, GER101, KMM575

Nectandra reticulata (Ruiz & Pav.) Mez	Lauraceae	RBU/PL379, JULS151, GER67
Persea americana Mill.	Lauraceae	JULS211, GER18
Allium sativum L.	Liliaceae	JULS92, GER37
Hesperoxiphion niveum (Ravenna) Ravenna	Iridaceae	JULS269, TRUVan/Erica9, TRUBH27, RBU/PL325, JULS87, EHCHL79, GER93, AKT1100, ACR45
Linum savitum L.	Linaceae	EHCHL159, JULS185, GER139
Linum usitatissimum L.	Linaceae	EHCHL159a, JULS185a, GER139a
Centropogon reticulatus Drake	Lobeliaceae	EHCHL119
Centropogon cf.*rufus* E. Wimm.	Lobeliaceae	GER210
Siphocampylus cutervensis Zahlbr.	Campanulaceae	GER102
Buddleja utilis Kraenzl.	Loganiaceae	RBU/PL333, EHCHL38, ISA60, JULS155, GER136, AKT1131
Strychnos sp.	Loganiaceae	RBU/PL378, EHCHL158
Psittacanthus chanduyensis Eichler	Loranthaceae	RBU/PL269
Tristerix longebracteatus (Des.) Barlow & Wiens	Loranthaceae	JULS296, GER74
Huperzia cf. *columnaris* B. Øellg.	Lycopodiaceae	GER106
Huperzia hohenackeri (Herter) Holub	Lycopodiaceae	TRUVan/Erica4
Huperzia kuesteri (Nessel) B. Øllg.	Lycopodiaceae	RBU/PL357, TRUVan/Erica3, TRUVan/Erica1, GER59, TRUVan/Erica15
Huperzia sp.	Lycopodiaceae	RBU/PL356(a)
Lycopodium clavatum L.	Lycopodiaceae	RBU/PL348, TRUBH 4, GER154
Huperzia reflexa (Lam.) Trevis.	Lycopodiaceea	RBU/PL359, EHCHL113
Cuphea strigulosa Kunth	Lythraceae	GER104, EHCHL35, VFCHL34, JULS33, ISA51, RBU/PL259, EHCHL43, JULS59, ISA53, GER147
Malesherbia ardens J.F. Macbr.	Malesherbiaceae	EHCHL139
Alcea rosea L.	Malvaceae	JULS78, JULS79
Malva parviflora L.	Malvaceae	JULS189
Malva sylvestris L.	Malvaceae	VFCHL49, EHCHL29, ACR8
Miconia salicifolia (Bonpl. ex Naud.) Naud.	Melastomataceae	GER83, AKT1204, KMM544
Trichilia sp.	Meliaceae	RBU/PL380
Peumus boldus Molina	Monimiaceae	JULS114, GER157
Siparuna muricata (Ruiz & Pav.) A. DC.	Monimiaceae	GER88, EHCHL129, ISA113, ISA64
Brosimum rubescens Taub.	Moraceae	JULS209, ISA49, EHCHL64, RBU/PL311, GER86, EHCHL62, KMM570
Myrica pubescens Humb. & Bonpl. ex Willd.	Myricaceae	ISA84, ISA128
Myristica fragrans Houtt.	Myristicaceae	RBU/PL385, EHCHL155, JULS292, GER197
Eucalyptus citriodora Hook.	Myrtaceae	JULS60, KMM454
Eucalyptus globulus Labill.	Myrtaceae	ISA130, JULS61, VFCHL35, JULS153, GER14, EHCHL12, AKT1110, KMM408, ACR74
Eugenia obtusifolia Cambess.	Myrtaceae	JULS32, ACR19, ACR76, ACR180
Psidium guayava L.	Myrtaceae	VFCHL24, KMM399
Boerhavia coccinea Mill.	Nyctaginaceae	GER122, RBU/PL347
Mirabilis jalapa L.	Nyctaginaceae	JULS116, GER185
Heisteria acuminata (Humb. & Bonpl.) Engl.	Olacaceae	RBU/PL287, JULS138, GER164, KMM507
Ximenia americana L.	Olacaceae	JULS184
Epilobium sp.	Onagraceae	ISA46
Fuchsia ayavacensis Kunth	Onagraceae	ISA82, ISA1
Oenothera rosea L'Hér. ex Aiton	Onagraceae	RBU/PL366
Aa paleacea (Kunth) Rchb. f.	Orchidaceae	ISA141, EHCHL75, AKT1185, ACR144, KMM530
Epidendrum calanthum Rchb. f. & Warsz.	Orchidaceae	GER79
Lycaste gigantea Lindl.	Orchidaceae	GER156
Pachyphyllum pastii Kraenzl. ex Weberb.	Orchidaceae	EHCHL97
Stelis eublepharis Rchb.f.	Orchidaceae	RBU/PL342, EHCHL9, VFCHL40
Oxalis bulbigera R. Knuth	Oxalidaceae	JULS261
Argemone mexicana L.	Papaveraceae	JULS126, GER176, KMM433, AKT1135, ACR61
Passiflora caerulea L.	Passifloraceae	JULS217
Passiflora ligularis Juss.	Passifloraceae	EHCHL47, JULS163, KMM420, AKT1112
Phytolacca bogotensis Kunth	Phytolaccaceae	ISA81, ISA111, JULS218, KMM457, AKT1180

Peperomia fraseri C.DC.	Piperaceae	EHCHL7, RBU/PL341, VFCHL32, TRUVan/Erica16, AKT1133, AKT1146, KMM476
Peperomia galioides Kunth	Piperaceae	EHCHL95, VFCHL38, RBU/PL298
Peperomia hartwegiana Miq.	Piperaceae	ISA134, ISA92, GER127
Peperomia inaequalifolia Ruiz & Pav.	Piperaceae	TRUBH12, JULS30, VFCHL39, EHCHL8, RBU/PL297, GER80, AKT1148, KMM534
Peperomia quadrifolia (L.) Kunth	Piperaceae	EHCHL66, GER130, JULS306, KMM491, KMM532
Piper aduncum L.	Piperaceae	VFCHL26, RBU/PL277, TRUVan/Erica24, JULS15, GER141, JULS199, AKT1150, ACR12
Piper cf. *aequale* Vahl.	Piperaceae	EHCHL82, RBU/PL272
Piper nigrum L.	Piperaceae	JULS227
Plantago linearis Kunth	Plantaginaceae	JULS35, JULS86, GER133
Plantago major L.	Plantaginaceae	VFCHL50, EHCHL11, TRUVan/Erica13, KMM411
Plantago sericea Ruiz & Pav.	Plantaginaceae	EHCHL98, KMM499, AKT1182, ACR186
Plantago sericea Ruiz & Pav. subsp. *sericans* (Pilg.) Rahn	Plantaginaceae	RBU/PL335, EHCHL96
Plantago sericea Ruiz & Pav. var. *lanuginosa* Griseb.	Plantaginaceae	JULS207
Cenchrus echinatus L.	Poaceae	JULS89
Cynodon dactylon (L.) Pers.	Poaceae	ISA61, JULS73, ISA106, GER151, ACR62, KMM450
Digitaria ciliaris (Retz.) Koeler	Poaceae	GER69, JULS220
Hordeum vulgare L.	Poaceae	JULS128, GER183
Saccharum officinarum L.	Poaceae	VFCHL4, JULS123, GER208, AKT1216, KMM502
Zea mays L.	Poaceae	JULS69, JULS139, GER31, GER186
Cantua buxifolia Juss. ex Lam.	Polemoniaceae	JULS297, ACR86
Cantua quercifolia Juss.	Polemoniaceae	RBU/PL362, EHCHL100, GER144. ISA10, AKT1161, KMM495
Polygala paniculata L.	Polygalaceae	EHCHL59
Muehlenbeckia tamnifolia (Kunth) Meisn.	Polygonaceae	RBU/PL309, ISA30
Cheilanthes myriophylla Desv.	Pteridaceae	GER94, EHCHL37, KMM431, ACR145
Jamesonia goudotii (Hieron.) C. Chr.	Pteridaceae	ISA146(107a)
Jamesonia rotundifolia Fée	Polypodiaceae	RBU/PL343, ISA132, EHCHL26, TRUVan/Erica11, TRUBH22
Polypodium crassifolium L.	Polypodiaceae	EHCHL71, TRUBH38, RBU/PL331, RBU/PL332, JULS52, JULS303, AKT1137
Portulaca oleracea L. subsp. *tuberculata* Danin & H.G. Baker	Portulacaceae	JULS268
Portulaca villosa Cham.	Portulacaceae	GER171
Oreocallis grandiflora (Lam.) R.Br.	Proteaceae	EHCHL127, JULS31, ISA28, ISA70, AKT1137, AKT1173, KMM514
Punica granatum L.	Punicaceae	JULS159
Laccopetalum giganteum (Wedd.) Ulbr.	Ranunculaceae	VFCHL53, RBU/PL321, EHCHL42, JULS284, GER162, AKT1119, KMM410
Alchemilla nivalis Kunth	Rosaceae	ISA97
Cydonia oblonga Mill.	Rosaceae	JULS194
Fragaria vesca L.	Rosaceae	JULS158
Polylepis racemosa Ruiz & Pav.	Rosaceae	JULS2, ACR3
Prunus serotina Ehrh.	Rosaceae	EHCHL94, ACR172
Prunus serotina Ehrh. subsp.*capuli* (Cav.) McVaugh	Rosaceae	JULS51
Rubus robustus C.Presl.	Rosaceae	EHCHL132(a), ISA41, ISA48, JULS47, EHCHL132(b), AKT1155, ACR70
Sanguisorba minor Scop.	Rosaceae	EHCHL117, TRUBH35, RBU/PL262, ISA57, JULS25, ISA147(103a), VFCHL20, GER170, ACR23
Cinchona officinalis L.	Rubiaceae	RBU/PL314, JULS127, ISA19, GER167, ACR123, KMM525
Cinchona sp.	Rubiaceae	JULS127a
Uncaria tomentosa (Willd. ex Roem. & Schult.) DC.	Rubiaceae	VFCHL11, RBU/PL263, EHCHL103, JULS275, GER230, ACR198
Citrus aurantium L.	Rutaceae	EHCHL105
Citrus limetta Risso	Rutaceae	JULS182, GER177, KMM425
Citrus limon (L.) Burm.f.	Rutaceae	JULS183, GER11, KMM424

Citrus reticulata Blanco	Rutaceae	JULS191
Citrus sinensis (L.) Osbeck	Rutaceae	JULS202, GER178
Ruta graveolens L.	Rutaceae	ISA152, JULS1, TRUVan/Erica20, EHCHL128, VFCHL16, ISA145(108a), GER24, AKT1097, AKT1105, KMM430
Populus deltoides W. Bartram ex Marshall	Salicaceae	JULS93
Salix chilensis Molina	Salicaceae	TRUBH25, JULS82, GER39, AKT1106, ACR42
Dodonaea viscosa (L.) Jacq.	Santalaceae	
Calceolaria rugulosa Edwin	Scrophulariaceae	JULS232
Scutellaria scutellariodes (Kunth) Harley	Lamiaceae	
Smilax medica Schltdl. & Cham.	Smilacaceae	GER218, JULS273
Brugmansia arborea (L.) Lagerh.	Solanaceae	GER64, VFCHL18, GER50, JULS157, GER52
Brugmansia candida Pers.	Solanaceae	GER54, RBU/PL316, RBU/PL327, RBU/PL328, GER51, GER77, KMM564
Brugmansia sanguinea (Ruiz & Pav.) D. Don	Solanaceae	GER103, EHCHL10, VFCHL23, RBU/PL250, GER53, KMM528
Brugmansia sp.	Solanaceae	Ger64a
Cestrum auriculatum L'Hér.	Solanaceae	JULS166, RBU/PL281, EHCHL172, ISA122, GER174, EHCHL102, ACR36
Cestrum nocturnum L.	Solanaceae	ISA142
Cestrum strigilatum Ruiz & Pav.	Solanaceae	JULS245
Cestrum undulatum Ruiz & Pav.	Solanaceae	JULS245a
Jaltomata sp.	Solanaceae	GER58
Lycopersicon hirsutum Dunal	Solanaceae	ISA31
Nicotiana tabacum L.	Solanaceae	JULS251, GER92, ACR53, KMM388, KMM557
Solanum americanum Mill.	Solanaceae	EHCHL125, JULS76, EHCHL87, GER85, GER159, ACR37, ACR99
Solanum mammosum L.	Solanaceae	VFCHL45, GER56, GER55, GER153
Solanum sp.	Solanaceae	ISA3
Solanum tuberosum L.	Solanaceae	JULS140, JULS141
Thelypteris cf. *scalaris* (Christ.) Alston	Thelypteridaceae	JULS291
Daphnopsis weberbaueri Domke	Thymelaeaceae	EHCHL153, JULS137, GER216
Tilia platyphyllos Scop.	Tiliaceae	JULS257
Tropaeolum minus L.	Tropaeolaceae	JULS81
Typha angustifolia L.	Typhaceae	ISA45
Celtis loxensis C.C.Berg	Ulmaceae	JULS208, EHCHL65, GER87, ISA7, KMM561
Pilea microphylla (L.) Lieberm.	Urticaceae	RBU/PL282, EHCHL33
Urtica magellanica Juss. ex Poir.	Urticaceae	RBU/PL251, ISA119, JULS11, EHCHL50, VFCHL9, GER161
Urtica urens L.	Urticaceae	RBU/PL251a, ISA119a, ACR199
Belonanthus aff. *hispidus* (Wedd.) Graebn.	Valerianaceae	JULS299, AKT1186
Phyllactis rigida (Ruiz & Pav.) Pers.	Valerianaceae	EHCHL163, TRUBH30, JULS57, EHCHL44, JULS46, ISA137, RBU/PL365 , RBU/PL355, GER187, AKT1205, KMM481, AKT1117
Valeriana bonplandiana Wedd.	Valerianaceae	RBU/PL350
Valeriana plantaginea Kunth	Valerianaceae	GER193, EHCHL91, EHCHL120, ACR120
Aloysia triphylla Royle	Verbenaceae	JULS130, RBU/PL384, EHCHL161, RBU/PL305, GER90, KMM398
Clerodendron sp.	Verbenaceae	JULS115
Lantana scabiosiflora Kunth	Verbenaceae	VFCHL51, GER6, AKT1123, ACR50
Lippia integrifolia (Griseb.) Hieron.	Verbenaceae	EHCHL76
Verbena litoralis Kunth	Verbenaceae	RBU/PL369, JULS77, EHCHL69, VFCHL28, GER138, ACR13
Viola tricolor L.	Violaceae	JULS36, VFCHL19, AKT1203, ACR195
Xyris subulata Ruiz & Pav.	Xyridaceae	ISA103, RBU/PL349, JULS300, GER132, JULS306
Zingiber officinale Roscoe	Zingiberaceae	ULS237, GER206
Tribulus terrestris L.	Zygophyllaceae	GER137
