# Supplementary material for: Herbal mixtures in traditional medicine in Northern Peru
Source: J Ethnobiol Ethnomed. 2010 Mar 14;6:10. doi: 10.1186/1746-4269-6-10 (PMC2848642; doi:10.1186/1746-4269-6-10)

Additional file 3. Dendrograms

Hierarchical Clustering Report

Page/Date/Time 1 2/8/2010 3:28:51 PM
Database C:\Documents and Settings\rb ... NCSS 2007\Junk\Mixtures4.S0N
Variables Aa_paleacea__H_B_K___Rchb__f_ Aa_paleacea__H_B_K___Rchb__f_ to Pseudogynoxis_cordifolia Pseudogynoxis_cordifolia
Clustering Method Group Average (Unweighted Pair-Group)
Distance Type Euclidean
Scale Type Standard Deviation

Dendrogram Section

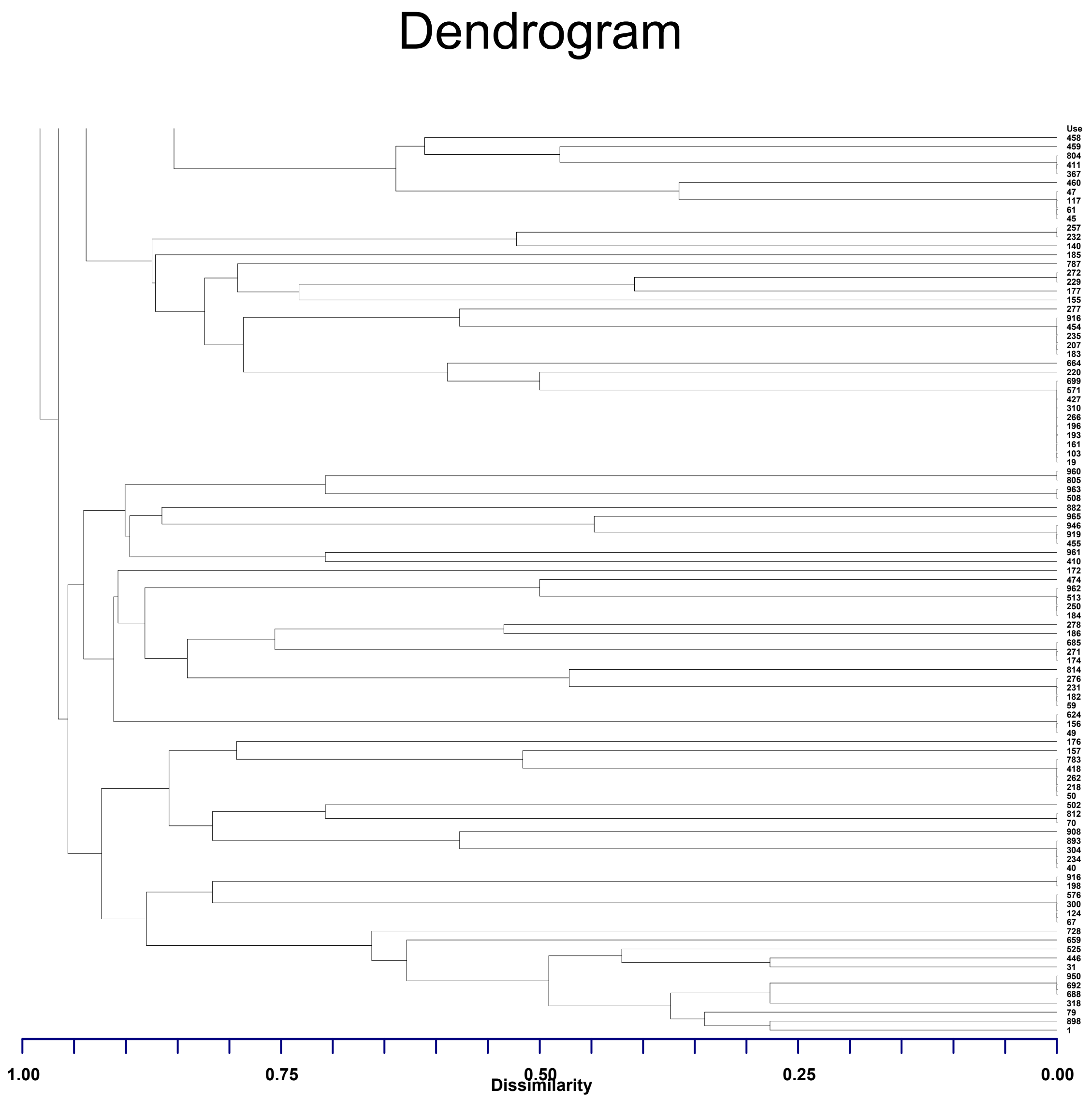

a

Hierarchical Clustering Report

Page/Date/Time	2 2/8/2010 3:28:51 PM
Database	C:\Documents and Settings\rb ... NCSS 2007\Junk\Mixtures4.S0N
Variables	Aa_paleacea__H_B_K___Rchb__f_ Aa_paleacea__H_B_K___Rchb__f_ to Pseudogynoxis_cordifolia Pseudogynoxis_cordifolia
Clustering Method	Group Average (Unweighted Pair-Group)
Distance Type	Euclidean
Scale Type	Standard Deviation

Dendrogram

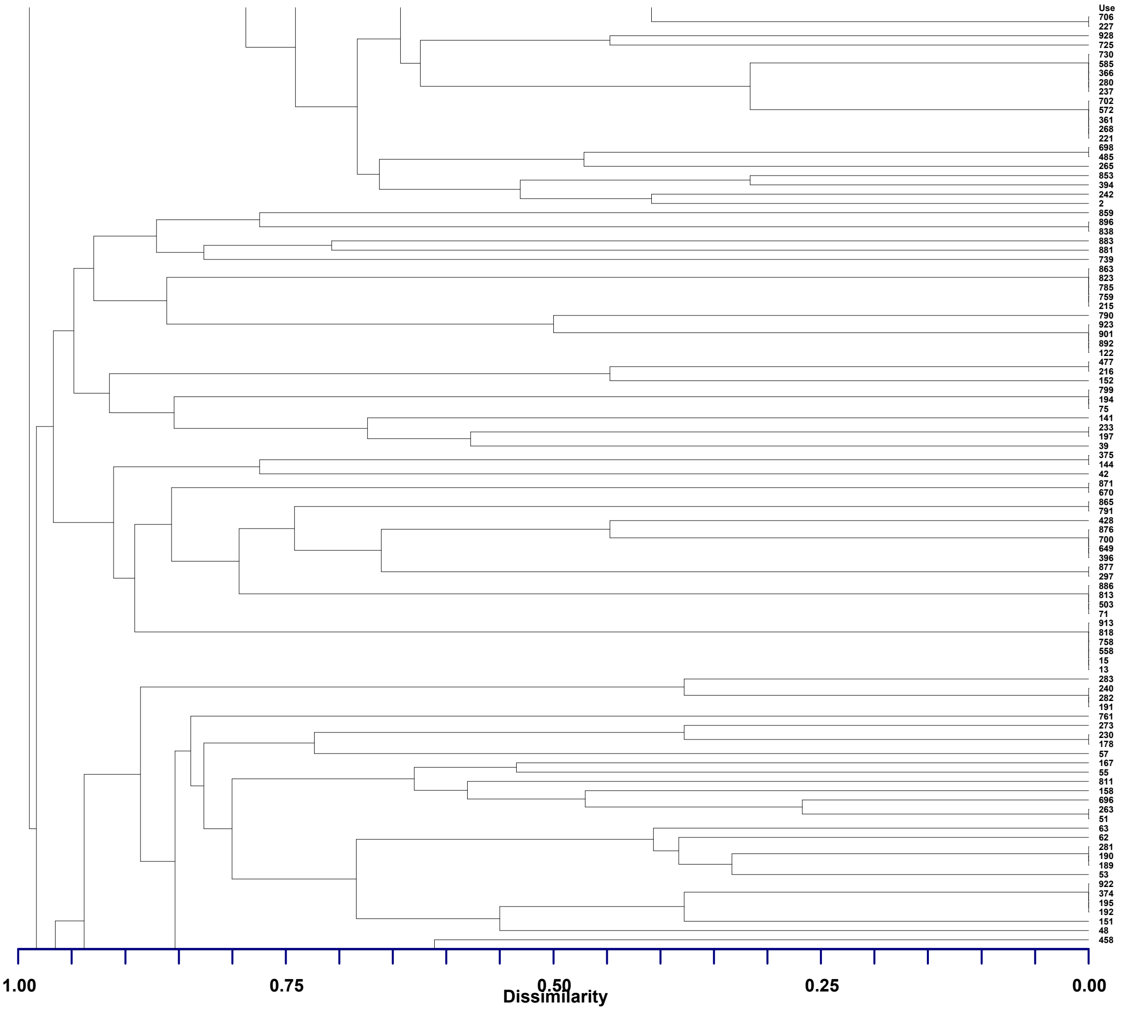

b

Hierarchical Clustering Report

Page/Date/Time 3 2/8/2010 3:28:51 PM
Database C:\Documents and Settings\rb ... NCSS 2007\Junk\Mixtures4.S0N
Variables Aa_paleacea__H_B_K___Rchb__f_ Aa_paleacea__H_B_K___Rchb__f_ to Pseudogynoxis_cordifolia Pseudogynoxis_cordifolia
Clustering Method Group Average (Unweighted Pair-Group)
Distance Type Euclidean
Scale Type Standard Deviation

Dendrogram

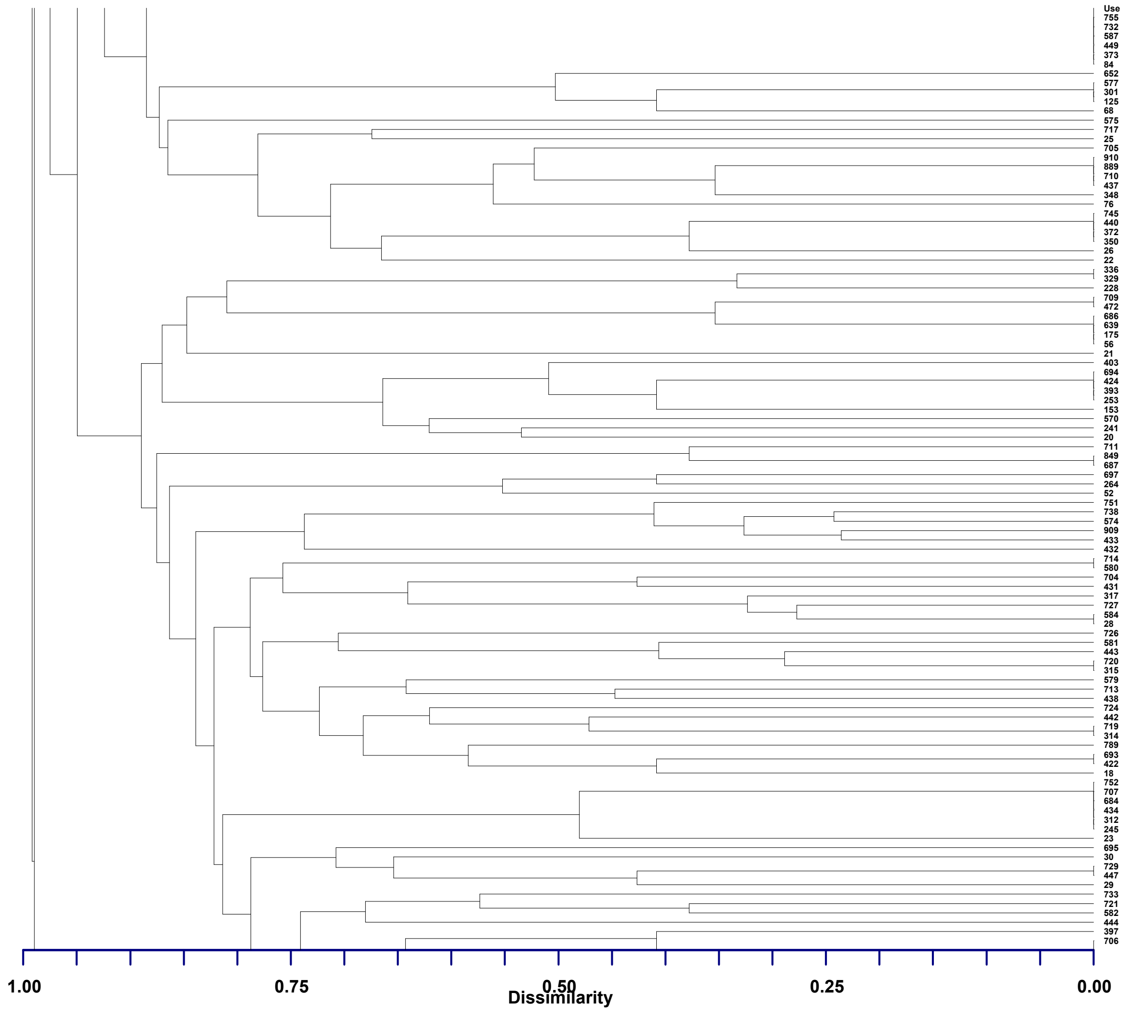

c

Hierarchical Clustering Report

Page/Date/Time	4 2/8/2010 3:28:51 PM
Database	C:\Documents and Settings\rb ... NCSS 2007\Junk\Mixtures4.S0N
Variables	Aa_paleacea__H_B_K___Rchb__f_ Aa_paleacea__H_B_K___Rchb__f_ to Pseudogynoxis_cordifolia Pseudogynoxis_cordifolia
Clustering Method	Group Average (Unweighted Pair-Group)
Distance Type	Euclidean
Scale Type	Standard Deviation

Dendrogram

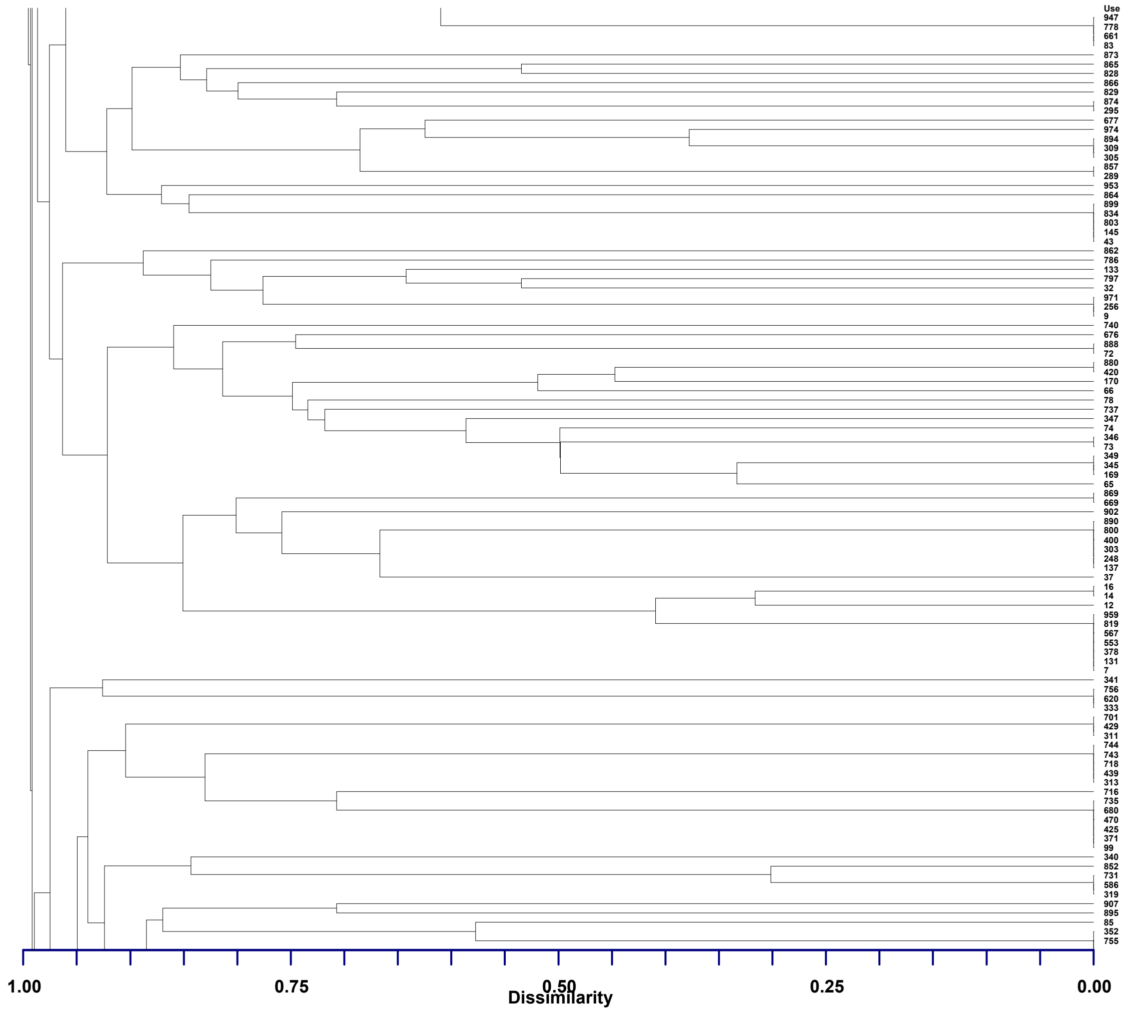

d

Hierarchical Clustering Report

Page/Date/Time 5 2/8/2010 3:28:51 PM
Database C:\Documents and Settings\rb ... NCSS 2007\Junk\Mixtures4.S0N
Variables Aa_paleacea__H_B_K___Rchb__f_ Aa_paleacea__H_B_K___Rchb__f_ to Pseudogynoxis_cordifolia Pseudogynoxis_cordifolia
Clustering Method Group Average (Unweighted Pair-Group)
Distance Type Euclidean
Scale Type Standard Deviation

Dendrogram

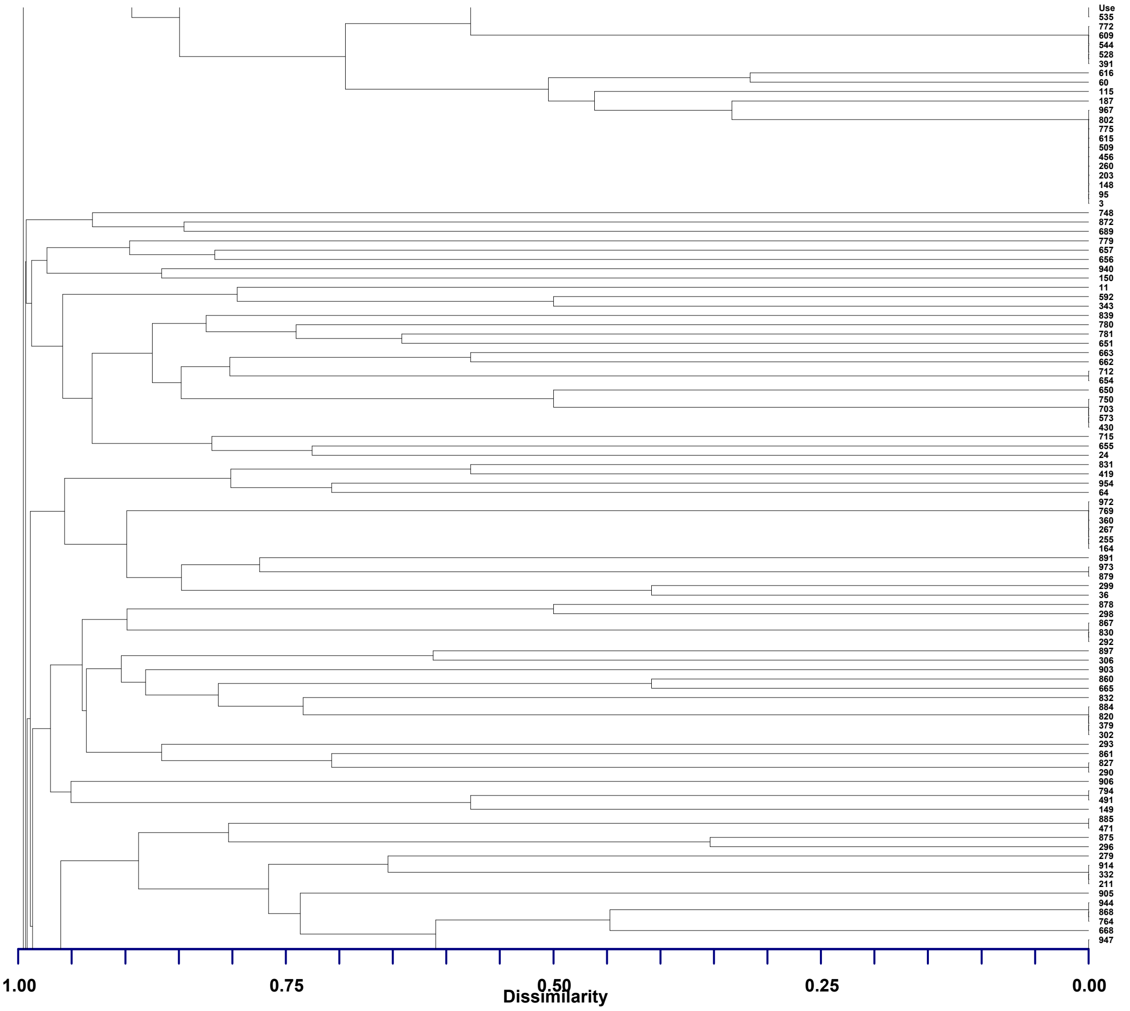

e

Hierarchical Clustering Report

Page/Date/Time	6 2/8/2010 3:28:51 PM
Database	C:\Documents and Settings\rb ... NCSS 2007\Junk\Mixtures4.S0N
Variables	Aa_paleacea__H_B_K___Rchb__f_ Aa_paleacea__H_B_K___Rchb__f_ to Pseudogynoxis_cordifolia Pseudogynoxis_cordifolia
Clustering Method	Group Average (Unweighted Pair-Group)
Distance Type	Euclidean
Scale Type	Standard Deviation

Dendrogram

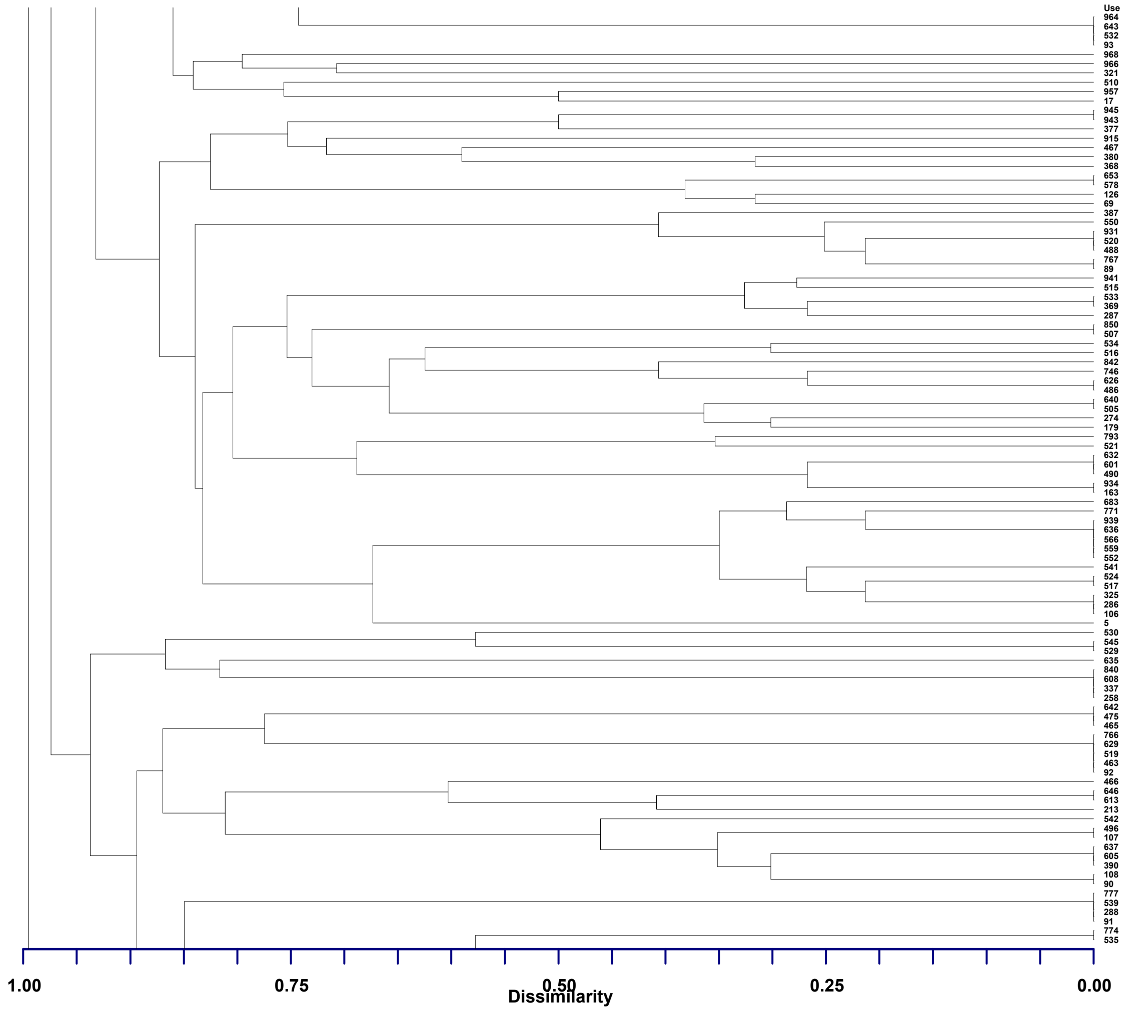

Hierarchical Clustering Report

Page/Date/Time 7 2/8/2010 3:28:51 PM
Database C:\Documents and Settings\rb ... NCSS 2007\Junk\Mixtures4.S0N
Variables Aa_paleacea__H_B_K___Rchb__f_ Aa_paleacea__H_B_K___Rchb__f_ to Pseudogynoxis_cordifolia Pseudogynoxis_cordifolia
Clustering Method Group Average (Unweighted Pair-Group)
Distance Type Euclidean
Scale Type Standard Deviation

Dendrogram

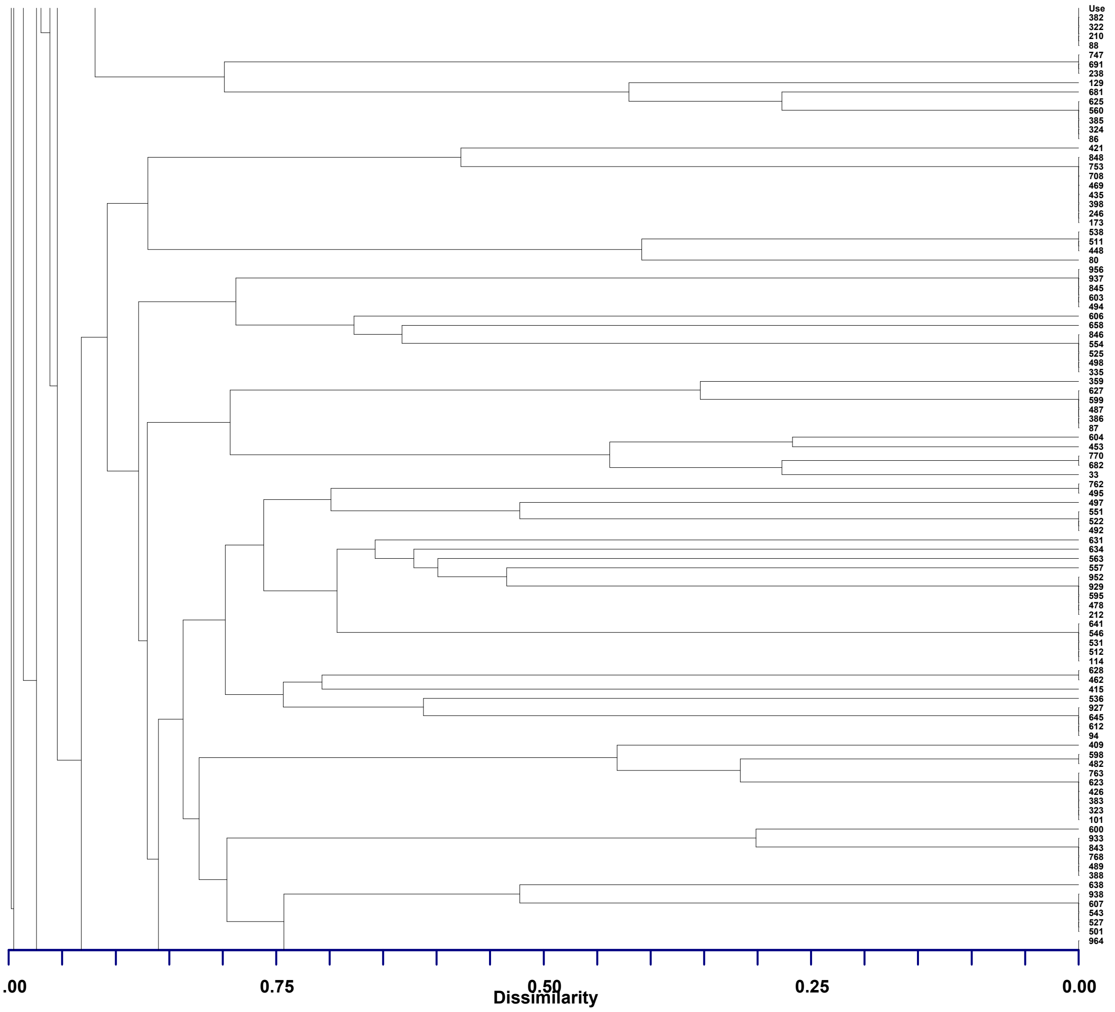

Hierarchical Clustering Report

Page/Date/Time 8 2/8/2010 3:28:51 PM
Database C:\Documents and Settings\rb ... NCSS 2007\Junk\Mixtures4.S0N
Variables Aa_paleacea__H_B_K___Rchb__f_ Aa_paleacea__H_B_K___Rchb__f_ to Pseudogynoxis_cordifolia Pseudogynoxis_cordifolia
Clustering Method Group Average (Unweighted Pair-Group)
Distance Type Euclidean
Scale Type Standard Deviation

Dendrogram

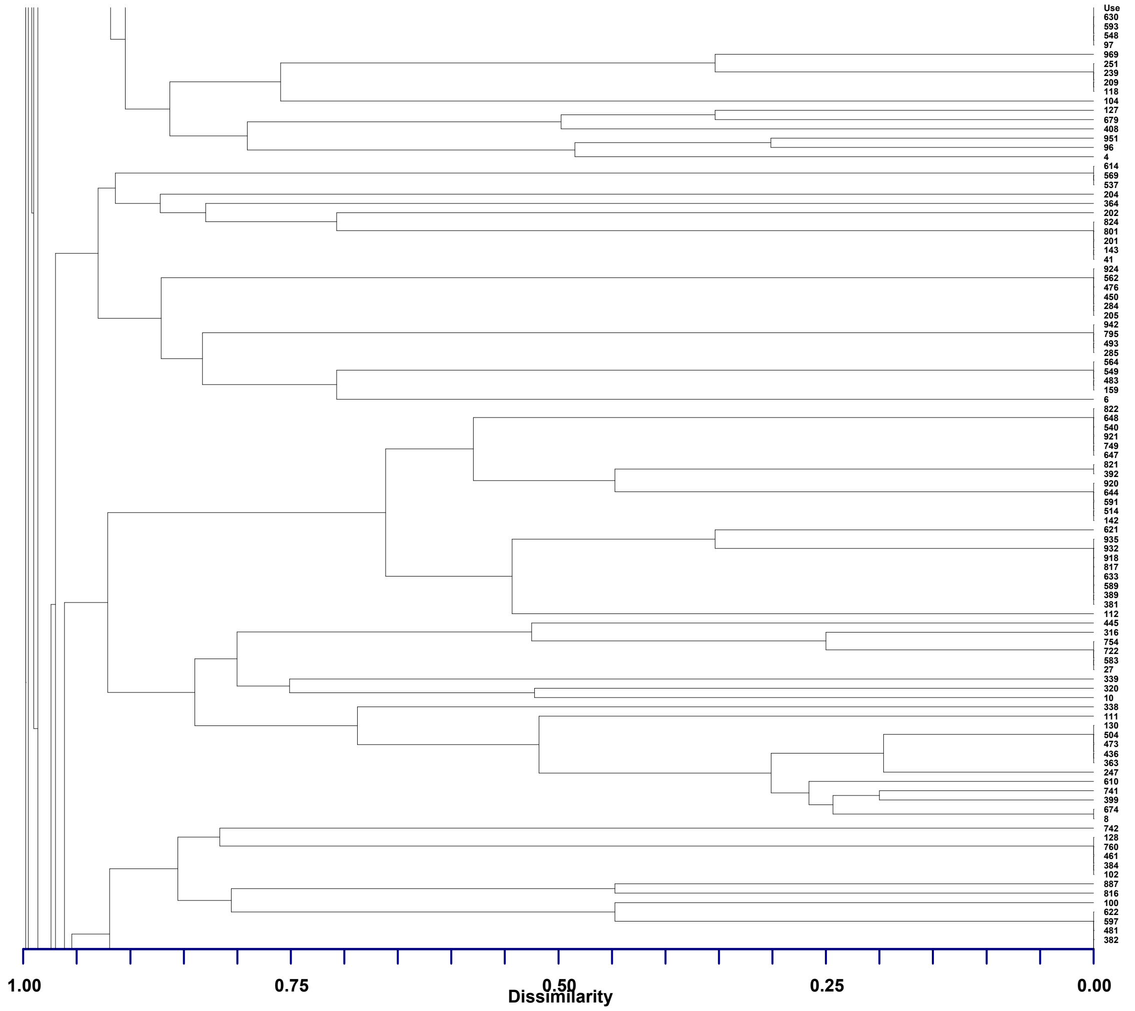

h

Hierarchical Clustering Report

Page/Date/Time 9 2/8/2010 3:28:51 PM
Database C:\Documents and Settings\rb ... NCSS 2007\Junk\Mixtures4.S0N
Variables Aa_paleacea__H_B_K___Rchb__f_ Aa_paleacea__H_B_K___Rchb__f_ to Pseudogynoxis_cordifolia Pseudogynoxis_cordifolia
Clustering Method Group Average (Unweighted Pair-Group)
Distance Type Euclidean
Scale Type Standard Deviation

Dendrogram

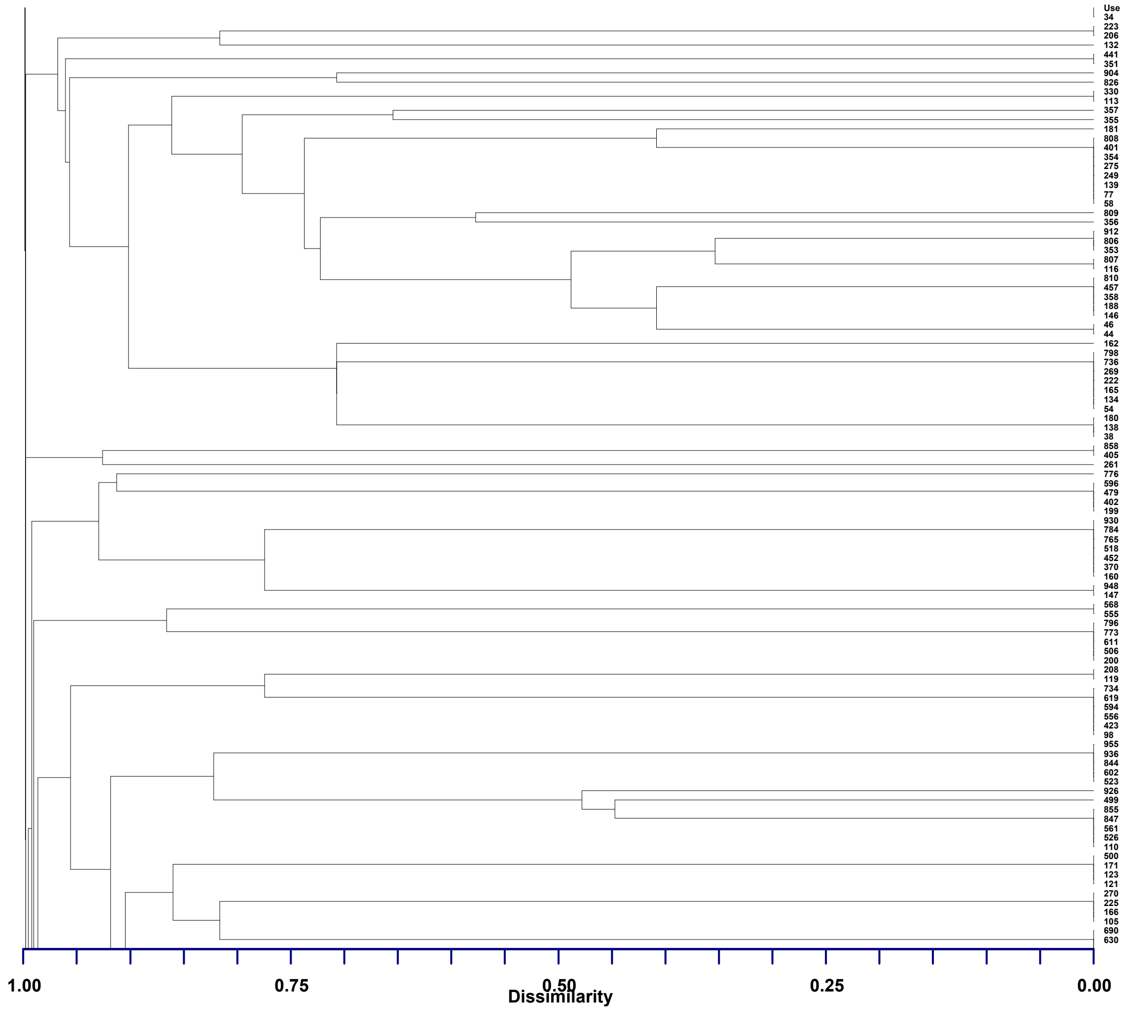

Hierarchical Clustering Report

Page/Date/Time 10 2/8/2010 3:28:51 PM
Database C:\Documents and Settings\rb ... NCSS 2007\Junk\Mixtures4.S0N
Variables Aa_paleacea__H_B_K___Rchb__f_ Aa_paleacea__H_B_K___Rchb__f_ to Pseudogynoxis_cordifolia Pseudogynoxis_cordifolia
Clustering Method Group Average (Unweighted Pair-Group)
Distance Type Euclidean
Scale Type Standard Deviation

Dendrogram

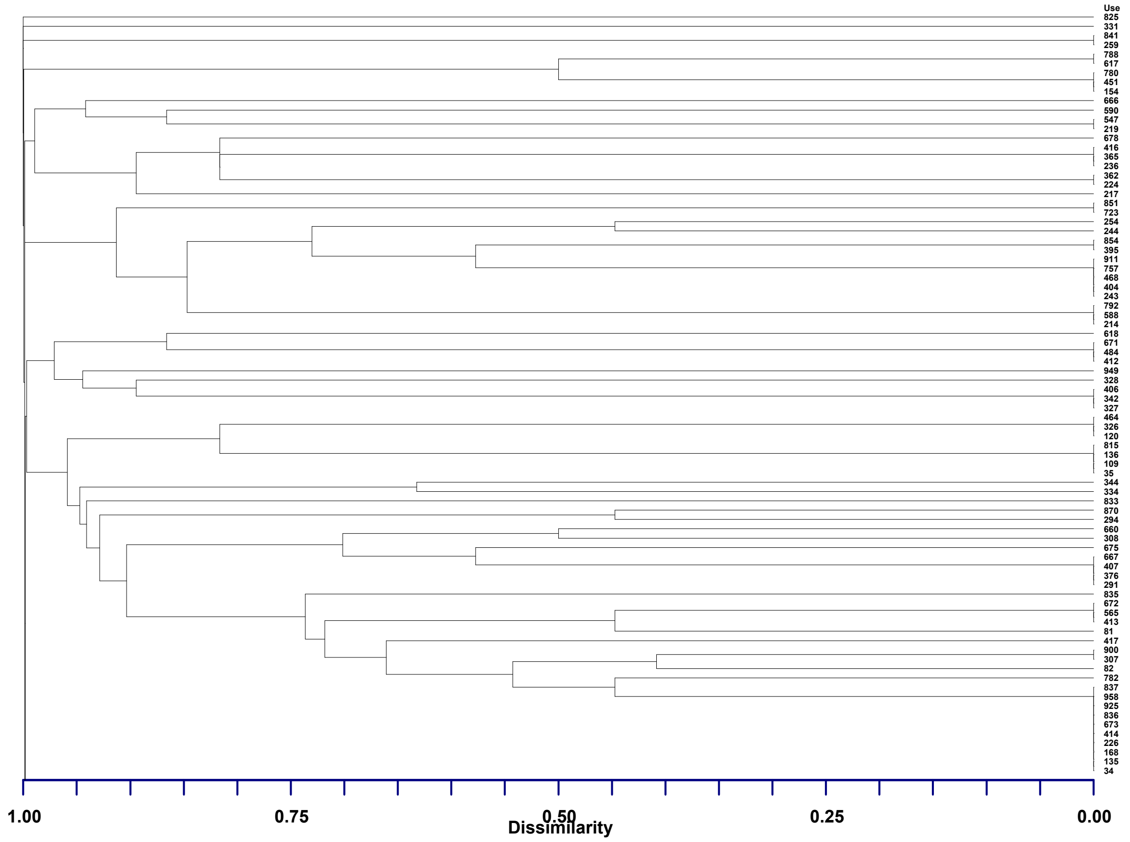

Supplement: Additional file 3 — Dendrograms. [file 1746-4269-6-10-S3.PDF]
