# Supplementary material for: Herbal mixtures in traditional medicine in Northern Peru
Source: J Ethnobiol Ethnomed. 2010 Mar 14;6:10. doi: 10.1186/1746-4269-6-10 (PMC2848642; doi:10.1186/1746-4269-6-10)
Supplement: Additional file 4 — Most important plant species. [file 1746-4269-6-10-S4.PDF]

Additional file 4. Most important plant species 64 species = 19.39% introduced; 1727 mentions = 23.91% introduced

# applications	*Scientific name*	Family	%	# applications	*Scientific name*	Family	%
**182**	*Equisetum bogotense* Kunth.	Equisetaceae	18.82	**40**	*Nectandra retuculata* (R.P.) Mez.	Lauraceae	4.14
**177**	*Equisetum giganteum* (Wedd.) Ulbrich.	Equisetaceae	18.30	**38**	*Aiouea dubia* (Kunth) Mez.	Lauraceae	3.93
**159**	*Ambrosia peruviana* Willd.	Asteraceae	16.44	**38**	*Iresine herbstii* Hook.	Amaranthaceae	3.93
**158**	*Bidens pilosa* L.	Asteraceae	16.34	**38**	*Pilea microphylla* (L.) Liebm.	Urticaceae	3.93
**125**	*Melissa officinalis* L.	Lamiaceae	12.92	**38**	*Sambucus peruviana* Kunth	Caprifoliaceae	3.93
**123**	*Matricaria recutita* L.	Asteraceae	12.72	**37**	*Adiantum concinnum* Humb. & Bonpl. ex Willd.	Adiantaceae	3.83
**119**	*Verbena litoralis* Kunth	Verbenaceae	12.31	**37**	*Jamesonia rotundifolia* Fée	Polypodiaceae	3.83
**113**	*Sanguisorba minor* Scop.	Rosaceae	11.69	**36**	*Gentianella bicolor* (Wedd.) J.S. Pringle	Gentianaceae	3.72
**109**	*Matricaria frigidum* (H.B.K.) Kunth.	Asteraceae	11.27	**34**	*Heisteria acuminata* (Humb. & Bonpl.) Engl.	Olacaceae	3.52
**106**	*Bejaria aestuans* Mutis ex L.	Ericaceae	10.96	**34**	*Schinus molle* L.	Anacardiaceae	3.52
**103**	*Eucalyptus globulus* Labill.	Myrtaceae	10.65	**33**	*Brosimum rubescens* Taub.	Moraceae	3.41
**101**	*Desmodium molliculum*(H.B.K.)D.C.	Fabaceae	10.44	**33**	*Geranium ayavacense* Willd. ex Kunth	Geraniaceae	3.41
**97**	*Salvia rosmarinifolia* G. Don	Lamiaceae	10.03	**33**	*Phyllanthus stipulatus* (Raf.) G.L.Webster	Euphorbiaceae	3.41
**94**	*Mauria heterophylla* Kunth	Anacardiaceae	9.72	**33**	*Schkuhria pinnata* (Lam.) Kuntze ex Thell.	Asteraceae	3.41
**91**	*Rosmarinus officinalis* L.	Lamiaceae	9.41	**32**	*Geranium sessiliflorum* Cav.	Geraniaceae	3.31
**88**	*Moncatis flaverioides* H.B.K.	Asteraceae	9.1	**32**	*Oreocallis grandiflora* (Lam.) R. Br.	Proteaceae	3.31
**86**	*Buddleja utilis* Kraenzl.	Loganiaceae	8.89	**32**	*Phyllanthus niruri* L.	Euphorbiaceae	3.31
**84**	*Cuphea strigulosa* Kunth	Lythraceae	8.69	**32**	*Senecio tephrosioides* Turcz.	Asteraceae	3.31
**77**	*Piper aduncum* L.	Piperaceae	7.96	**31**	*Eugenia obtusifolia* Cambess.	Myrtaceae	3.21
**74**	*Alternanthera porrigens* (Jacq.) Kuntze	Amaranthaceae	7.65	**31**	*Siparuna muricata* R. & P.	Monimiaceae	3.21
**71**	*Pelargonium odoratissimum* (L.) L'Hér. .	Geraniaceae	7.34	**30**	*Dioscorea trifida* L. f.	Dioscoreaceae	3.10
**68**	*Oritrophium peruvianum*(LAm.) Cautrec.	Asteraceae	7.03	**30**	*Polygala paniculata* L.	Polygalaceae	3.10
**68**	*Tiquilia paronychioides* (Phil.) A.T. Richardson	Borabinaceae	7.03	**30**	*Salvia officinalis* L.	Lamiaceae	3.10
**66**	*Origanum majorana* L.	Lamiaceae	6.83	**30**	*Urtica magellanica* A. Jussieu ex DC	Urticaceae	3.10
**64**	*Malva sylvestris* L.	Malvaceae	6.62	**29**	*Bixa orellana* L.	Bixaceae	3.00
**64**	*Ruta graveolens* L.	Rutaceae	6.62	**29**	*Minthostachys mollis* (Kunth) Griseb.	Lamiaceae	3.00
**63**	*Dianthus caryophyllus* L.	Caryophyllaceae	6.51	**29**	*Rorippa nasturtium-aquaticum* (L.) Hayek	Brassicaceae	3.00
**62**	*Alternanthera brasilians* (L.) Kuntze	Amaranthaceae	6.41	**28**	*Alcea rosea* L.	Malvaceae	2.89
**60**	*Artemisia absinthium* L.	Asteraceae	6.20	**28**	*Aloysia triphylla* (L'Her.) Britt.	Verbenaceae	2.89
**60**	*Laccopetalum giganteum* (Wedd.) Ulbr.	Ranunculaceae	6.20	**28**	*Gaultheria reticulata* Kunth	Ericaceae	2.89
**60**	*Malva parviflora* L.	Malvaceae	6.20	**28**	*Viola tricolor* L.	Violaceae	2.89
**60**	*Peumus boldus* Molina	Monimiaceae	6.20	**27**	*Brugmansia candida* Pers.	Solanaceae	2.79
**59**	*Achyroline alata* (H.B.K.) DC.	Asteraceae	6.10	**27**	*Salvia cuspidata* Ruiz & Pav.	Lamiaceae	2.79
**57**	*Foeniculum vulgare* Mill.	Apiaceae	5.89	**27**	*Tillandsia cacticola* L.B. Sm.	Bromeliaceae	2.79
**54**	*Cordia lutea* Lam	Boraginaceae	5.58	**26**	*Celtis loxensis* C.C. Berg	Ulmaceae	2.69
**54**	*Cynodon dactylon* (L.) Pers.	Poaceae	5.58	**26**	*Eustephia coccinea* Cav.	Amaryllidaceae	2.69
**54**	*Plantago linearis* Kunth	Plantaginaceae	5.58	**26**	*Salvia sagittata* Ruiz. & Pav.,	Lamiaceae	2.69
**52**	*Tagetes erecta* L.	Asteraceae	5.37	**25**	*Brugmansia arborea* (L.) Lagerh.	Solanaceae	2.59
**51**	*Baccharis genisteloides* (Lam.) Pers.	Asteraceae	5.27	**25**	*Malesherbia ardens* Macbr.	Malesherbiaceae	2.59
**51**	*Juglans neotropica* Diels.	Juglandaceae	5.27	**25**	*Marrubium vulgare* L.	Lamiaceae	2.59
**51**	*Lepechinia meyenii* (Walp.) Epling	Brassicaceae	5.27	**25**	*Mentha x piperita* L.	Lamiaceae	2.59
**49**	*Alternanthera halimifolia* (Lam.) Standl. ex Pittier	Amaranthaceae	5.07	**25**	*Tilia platyphyllos* Scop.	Tiliaceae	2.59
**48**	*Plantago major* L.	Plantaginaceae	4.96	**24**	*Corynaea crassa* Hook. f.	Balanophoraceae	2.48
**46**	*Borago officinalis* L.	Boraginaceae	4.76	**24**	*Lathyrus odoratus* L.	Fabaceae	2.48
**45**	*Cenchrus echinatus* L.	Poaceae	4.65	**24**	*Myrica pubescens* Humb. & Bonpl. ex Willd.	Myricaceae	2.48
**45**	*Senecio canescens* (Bonpl.) Cuatrec. Cuatrecasas	Asteraceae	4.65	**24**	*Phyllanthus urinaria* L.	Ericaceae	2.48
**45**	*Zea mays* L.	Poaceae	4.65	**24**	*Rubus robustus* C.Presl.	Rosaceae	2.48
**44**	*Peperomia inaequalifolia* Ruiz & Pav.	Piperaceae	4.55	**24**	*Stachys lanata* Jacq.	Lamiaceae	2.48
**43**	*Tribulus terrestris* L.	Zygophyllaceae	4.45	**24**	*Uncaria tomentosa* (Willd. ex Roem. & Schult.) DC.	Rubiaceae	2.48
**42**	*Cinchona officinalis* L.	Rubiaceae	4.34	**24**	*Urtica urens* L.	Urticaceae	2.48
**42**	*Smilax medica* Schltdl. & Cham.	Smilacaceae	4.34	**23**	*Brugmansia sanguinea*(R. &P.)D.	Solanaceae	2.38

**40**	*Linum sativum* L.	Linaceae	4.14	**23**	*Lonicera japonica* Thunberg	Caprifoliaceae	2.38
**40**	*Linum usitatissimum* L.	Linaceae	4.14	**23**	*Nerium oleander* L.	Apocynaceae	2.38
**23**	*Tillandsia multiflora* Benth. var. *decipiens* (André) L.B. Sm.	Bromeliaceae	2.38	**10**	*Portulaca oleracea* L. subsp. *tuberculata* Danin & H.G. Baker	Portulaccaceae	1.03
**22**	*Arctium lappa* L.	Asteraceae	2.28	**10**	*Tessaria integrifolia* Ruiz & Pav.	Asteraceae	1.03
**22**	*Centropogon* cf.*rufus* Wimm.	Lobeliaceae	2.28	**10**	*Thevetia peruviana* (Pers.) K. Schum.	Apocynaceae	1.03
**22**	*Muehlenbeckia tamnifolia* (Kunth) Meisn. Meisner	Polygonaceae	2.28	**9**	*Acanthoxanthium spinosum* L.	Asteraceae	0.93
**21**	*Peperomia fraseri* C. DC.	Piperaceae	2.17	**9**	*Mimosa nothacacia* Barneby	Fabaceae	0.93
**21**	*Salvia discolor* Kunth	Lamiaceae	2.17	**9**	*Petroselium crispum* (Miller) A.W. Hill	Apiaceae	0.93
**21**	*Trixis cacalioides* (Kunth) D. Don	Asteraceae	2.17	**9**	*Trifolium repens* L.	Lamiaceae	0.93
**20**	*Echeveria peruviana* Meyen.	Crassulaceae	2.07	**8**	*Acalypha mandonii* Müll. Arg.	Euphorbiaceae	0.84
**20**	*Iresine diffusa* Humb. & Bonpl. ex Willd.	Amaranthaceae	2.07	**8**	*Citrus sinesis* (L.) Osbeck	Rutaceae	0.84
**19**	*Caesalpinia spinosa* (Molina) Kuntze	Fabaceae	1.96	**8**	*Paranephelius uniflorus* Poepp.	Asteraceae	0.84
**19**	*Miconia salicifolia* (Bonpl. ex Naudin) Naudin	Melastomataceae	1.96	**8**	*Phyllactis rigida* (R.P.) Persoon	Valerianaceae	0.84
**19**	*Myroxylon balsamum* (L.) Harms	Fabaceae	1.96	**8**	*Ricinus communis* L.	Euphorbiaceae	0.84
**19**	*Satureja pulchella* (Kunth) Briq.	Lamiaceae	1.96	**8**	*Senecio chionogeton* Wedd.	Asteraceae	0.84
**19**	*Sonchus oleraceus* L.	Asteraceae	1.96	**8**	*Stelis eublepharis* Rchb. f.	Orchidaceae	0.84
**19**	*Strychnos* sp.	Loganiaceae	1.96	**7**	*Centropogon reticulatus* Drake	Lobeliaceae	0.72
**18**	*Erodium cicutarium* (L.) L'Hér. ex Aiton	Geraniaceae	1.86	**7**	*Diplostephium sagasteguii* Cuatrecasas	Asteraceae	0.72
**18**	*Lycaste gigantea* Lindl.	Orchidaceae	1.86	**7**	*Epidendrum calanthum* Rchb. f. & Warsz.	Orchidaceae	0.72
**18**	*Origanum vulgare* L.	Lamiaceae	1.86	**7**	*Eupatorium gayanum* Wedd.	Asteraceae	0.72
**18**	*Siphocampylus cutervensis* Zahlbr.	Lobeliaceae	1.86	**7**	*Gentianella crassicaulis* J.S. Pringle	Gentianaceae	0.72
**17**	*Cestrum nocturnum* L.	Solanaceae	1.76	**7**	*Hedyosmum racemosum* (Ruiz & Pav.) G. Don	Chloranthaceae	0.72
**17**	*Perezia multifora* (H. & B.) Lessing	Asteraceae	1.76	**7**	*Hyptis sidifolia*(L'Her.) Briq.	Lamiaceae	0.72
**17**	*Typha angustifolia* L.	Typhaceae	1.76	**7**	*Ipomoea batatas* (L.) Lam.	Convolvulaceae	0.72
**16**	*Ilex guayusa* Loes.	Aquifoliaceae	1.65	**7**	*Ipomoea pauciflora* M. Martens & Galeotti	Convolvulaceae	0.72
**16**	*Mentha spicata* L.	Lamiaceae	1.65	**7**	*Lippia integrifolia* (Grieseb.) Hieron	Verbenaceae	0.72
**16**	*Otholobium glandulosum* (L.) Grimes	Lamiaceae	1.65	**7**	*Psidium guajava* L.	Myrtaceae	0.72
**16**	*Plantago sericea* Ruiz & Pav. subsp. *sericans* (Pilg.) Rahn	Plantaginaceae	1.65	**7**	*Salix chilensis* Molina	Salicaceae	0.72
**16**	*Porophyllum ruderale* (Jacq.) Cass.	Asteraceae	1.65	**7**	*Sarcostemma clausum* (Jacq.) Schult.	Asclepiaedaceae	0.72
**16**	*Trichillia* sp.	Meliaceae	1.65	**7**	*Senecio genisianus* Cuatrec.	Asteraceae	0.72
**15**	*Cestrum auriculatum* L'Hér.	Solanaceae	1.55	**7**	*Solanum americanum* Mill.	Solanaceae	0.72
**15**	*Clerodendron* sp.	Verbenaceae	1.55	**7**	*Taraxacum officinale* F.H. Wigg.	Asteraceae	0.72
**15**	*Couepia* sp.	Chrysobalanaceae	1.55	**7**	*Werneria villosa* A. Gray	Asteraceae	0.72
**15**	*Cydonia oblonga* Mill.	Rosaceae	1.55	**7**	*Zingiber officinale* Roscoe	Zingiberaceae	0.72
**15**	*Escallonia pendula* (Ruiz & Pav.) Pers.	Grossulariaceae	1.55	**6**	*Cheilanthes myriophylla* Desv.	Polypodiaceae	0.62
**15**	*Lavandula angustifolia* Mill.	Lamiaceae	1.55	**6**	*Cronquistianthus lavandulifolius* (DC.) R.M. King & H. Rob.	Asteraceae	0.62
**15**	*Ocimum basilicum* L.	Lamiaceae	1.55	**6**	*Croton draconoides* Muell.-Arg.	Euphorbiaceae	0.62
**15**	*Scabiosa atropurpurea* L.	Dipsacaceae	1.55	**6**	*Croton lechleri* Muell.Arg.	Euphorbiaceae	0.62
**14**	*Allium sativum* L.	Liliaceae	1.45	**6**	*Erythroxylum coca* Lam	Erythroxylaceae	0.62
**14**	*Daucus montanus* Humb. & Bonpl. ex Spreng.	Apiaceae	1.45	**6**	*Hordeum vulgare* L.	Poaceae	0.62
**14**	*Fuchsia ayavacensis* Kunth	Onagraceae	1.45	**6**	*Inga edulis* C.Martius	Fabaceae	0.62
**14**	*Hesperoxiphion niveum* (Ravenna) Ravenna	Liliaceae	1.45	**6**	*Jamesonia goudotii* (Hieron.) C. Chr.	Polypodiaceae	0.62
**14**	*Phytolacca bogotensis* Kunth	Phytolaccaceae	1.45	**6**	*Passiflora ligularis* Juss.	Passifloraceae	0.62
**14**	*Pimpinella anisum* L.	Apiaceae	1.45	**6**	*Peperomia galioides* Kunth	Piperaceae	0.62
**13**	*Cordia alliodora* (Ruiz & Pav.) Okens	Boraginaceae	1.34	**6**	*Peperomia quadrifolia* (L.) Kunth	Piperaceae	0.62
**13**	*Peperomia hartwegiana* Miq.	Piperaceae	1.34	**6**	*Polypodium crassifolium* L.	Polypodiaceae	0.62
**12**	*Apium graveolens* L.	Apiaceae	1.24	**6**	*Portulaca villosa* Cham.	Portulacaceae	0.62
**12**	*Gaultheria erecta* Vent.	Ericaceae	1.24	**6**	*Senna bicapsularis* (L.) Roxb.	Fabaceae	0.62
**12**	*Hypericum laricifolium* Juss.	Clusiaceae	1.24	**6**	*Tetragonia crystallina* L'Hér.	Aizoaceae	0.62
**12**	*Polylepis racemosa* Ruiz & Pav.	Rosaceae	1.24	**5**	*Baccharis vaccinioides* Kunth	Asteraceae	0.52
**12**	*Salvia ayavacensis* Kunth	Lamiaceae	1.24	**5**	*Citrus limetta* Risso	Rutaceae	0.52
**11**	*Baccharis latifolia* (Ruiz & Pav.) Pers.	Asteraceae	1.14	**5**	*Echinopsis pachanoi* (Britton & Rose) Friedrich & G.D. Rowley	Cactaceae	0.52
**11**	*Epilobium* sp.	Onagraceae	1.14	**5**	*Ephedra americana* Humb. & Bonpl. ex Willd.	Ephedraceae	0.52
**11**	*Gentianella dianthoides* (Kunth) Fabris ex J.S. Pringle	Gentianaceae	1.14	**5**	*Leucaena leucocephala* (Lam.) Dewit	Fabaceae	0.52
**11**	*Solanum mammosum* L.	Solanaceae	1.14	**5**	*Mirabilis jalapa* L.	Nyctagynaceae	0.52

**11**	*Vallea stipularis* L. f.	Elaeocarpaceae	1.14
**10**	*Citrus limon* (L.) Burm. f.	Rutaceae	1.03
**10**	*Picrosia longifolia* D. Don.	Asteraceae	1.03
**5**	*Prunus serotina* Ehrh. subsp. *capuli* (Cav.) McVaugh	Rosaceae	0.52
**5**	*Scutellatia scutellariodes* Kunth	Scrophylariaceae	0.52
**4**	*Annona muricata* L.	Annonaceae	0.41
**4**	*Aristolochia ruiziana* (Klotzsch) Duch.	Aristolochiaceae	0.41
**4**	*Capparis crotonoides* H.B.K.	Capparidaceae	0.41
**4**	*Chuquiraga weberbaueri* Tovar	Asteraceae	0.41
**4**	*Cyclanthera pedata* (L.) Schrad.	Cucurbitaceae	0.41
**4**	*Dioscorea tambillensis* R. Knuth	Dioscoreaceae	0.41
**4**	*Inga feuilli* DC.	Fabaceae	0.41
**4**	*Nicotiana tabacum* L.	Solanaceae	0.41
**4**	*Oenothera rosea* L'Hér. ex Aiton	Onagraceae	0.41
**4**	*Salvia tubiflora* Ruiz & Pav.	Lamiaceae	0.41
**4**	*Sechium edule* (Jacq.) Sw.	Cucurbitaceae	0.41
**4**	*Xyris subulata* Ruiz & Pav.	Xyridaceae	0.41
**3**	*Acmella* cf. *ciliata* (Kunth) Cass.	Asteraceae	0.31
**3**	*Aphelandra cirsioides* Lindau	Acanthaceae	0.31
**3**	*Cantua quercifolia* Juss.	Polemoniaceae	0.31
**3**	*Cinnamomum verum* J. Presl.	Lauraceae	0.31
**3**	*Citrus aurantium* L.	Rutaceae	0.31
**3**	*Clethra castaneifolia* Meisn.	Clethraceae	0.31
**3**	*Cocos nucifera* L.	Arecaceae	0.31
**3**	*Dianthus carthusianorum* L.	Caryophyllaceae	0.31
**3**	*Diplostephium gynoxyoides* Cuatrec.	Asteraceae	0.31
**3**	*Ferreyranthus verbascifolius* (Kunth) H. Rob. & Brettell	Asteraceae	0.31
**3**	*Fragaria vesca* L.	Rosaceae	0.31
**3**	*Huperzia* cf. *columnaris* B. Øllg.	Lycopodiaceae	0.31
**3**	*Illicium verum* Hook.f.	Illiciaceae	0.31
**3**	*Jacaranda acutifolia* H. & B.	Bignoniaceae	0.31
**3**	*Jaltomata* sp.	Solanaceae	0.31
**3**	*Lantana scabiosiflora* Kunth	Verbenaceae	0.31
**3**	*Loxopterygium huasango* Spruce ex Engl.	Anacardiaceae	0.31
**3**	*Mandevilla* cf. *trianae* Woodson	Apocynaceae	0.31
**3**	*Mangifera indica* L.	Anacardiaceae	0.31
**3**	*Passiflora caerulea* L.	Passifloraceae	0.31
**3**	*Peperomia inaequalifolia* Ruiz & Pav.	Piperaceae	0.31
**3**	*Piper* cf. *aequale* Vahl	Piperacea	0.31
**3**	*Populus deltoides* W. Bartram ex Marshall	Salicaceae	0.31
**3**	*Prunus serotina* Ehrh.	Rosaceae	0.31
**3**	*Scirpus californicus* (C.A. Meyer) Steudel subsp. *tatora* (Kunth) T. Koyama	Juncaceae	0.31
**3**	*Solanum* sp.	Solanaceae	0.31
**3**	*Tagetes patula* L.	Asteraceae	0.31
**3**	*Tristerix longebracteatus* (Desr.) Barlow & Wiens	Loranthaceae	0.31
**3**	*Valeriana plantaginea* Kunth	Valerianaceae	0.31
**3**	*Werneria pygmaea* Gillies ex Hook. & Arn.	Asteraceae	0.31
**3**	*Ximenia americana* L.	Olacaceae	0.31
**2**	*Aa paleacea* (Kunth) Rchb. f.	Orchidaceae	0.21
**2**	*Argemone mexicana* L.	Papaveraceae	0.21
**2**	*Digitaria ciliaris* (Retz.) Koeler	Asteraceae	0.21
**2**	*Berberis buceronis* J.F. Macbr.	Berberidaceae	0.21
**2**	*Boerhavia coccinea* Mill.	Nyctaginaceae	0.21
**2**	*Cantua buxifolia* Juss. ex Lam.	Polemoniaceae	0.21
**2**	*Chamaesyce hypericifolia* (L.) Millsp.	Euphorbiaceae	0.21
**2**	*Daphnopsis weberbaueri* Domke	Thymeleaceae	0.21
**5**	*Oxalis bulbigera* R. Knuth	Oxalidaceae	0.52
**5**	*Persea americana* Mill.	Lauraceae	0.52
**5**	*Plantago sericea* Ruiz & Pav.	Plantaginaceae	0.52
**2**	*Daucus carota* L.	Apiaceae	0.21
**2**	*Desmodium triflorum* (L.) DC.	Fabaceae	0.21
**2**	*Gentianella brunneotincta* (Gilg) Pringle	Gentianaceae	0.21
**2**	*Huperzia hohenackeri* Herter	Lycopodiaceae	0.21
**2**	*Huperzia kuesteri* (Nessel) B. Øllg.	Lycopodiaceae	0.21
**2**	*Hydrocotyle bonariensis* Lam	Apiaceae	0.21
**2**	*Hypericum silenioides* Jus.	Clusiaceae	0.21
**2**	*Isoetes andina* Spruce ex Hook.	Isoetaceae	0.21
**2**	*Loricaria ferruginea* (Ruiz & Pav.) Wedd.	Asteraceae	0.21
**2**	*Mikania leiostachya* Benth.	Asteraceae	0.21
**2**	*Mimosa albida* Humb. & Bonpl. ex Willd.	Fabaceae	0.21
**2**	*Munnozia lyrata* (A. Gray) H. Rob. & Brettell	Asteraceae	0.21
**2**	*Niphogeton dissecta* (Benth.) J.F. Macbr.	Apiaceae	0.21
**2**	*Oreobolus goeppingeri* Suess.	Cyperaceae	0.21
**2**	*Pachyphyllum pastii* Kraenzl. ex Weberb.	Orchidaceae	0.21
**2**	*Piper nigrum* L.	Piperaceae	0.21
**2**	*Pseudogynoxys cordifolia* (Cass.) Cabrera	Asteraceae	0.21
**2**	*Saccharum officinarum* L.	Poaceae	0.21
**2**	*Sicana odorifera* (Vell.) Naudin	Cucurbitaceae	0.21
**2**	*Stellaria media* (L.) Vill.	Caryophyllaceae	0.21
**1**	*Alchemilla nivalis* H.B.K.	Rosaceae	0.1
**1**	*Alternanthera villosa* H.B.K.	Amaranthaceae	0.1
**1**	*Arnica montana* L.	Asteraceae	0.1
**1**	*Arracacia xanthorrhiza* Bancr.	Apiaceae	0.1
**1**	*Baccharis inidica* (L.) Gaert	Asteraceae	0.1
**1**	*Baccharis salicifolia* (Ruiz & Pav.) Pers.	Asteraceae	0.1
**1**	*Belonanthus* aff. *hispidus* (Wedd.) Graebn.	Valerianaceae	0.1
**1**	*Brugmannsia* sp.	Solanaceae	0.1
**1**	*Cajanus cajan* (L.) Huth	Fabaceae	0.1
**1**	*Caceolaria rugulosa* Edwin	Scrophulariaceae	0.1
**1**	*Cestrum strigilatum* Ruiz & Pav.	Solanaceae	0.1
**1**	*Cestrum undulatum* Ruiz & Pav.	Solanaceae	0.1
**1**	*Chenopodium quinoa* Willd.	Chenopodiaceae	0.1
**1**	*Cinchona* sp.	Rubiaceae	0.1
**1**	*Citrus reticulata* Blanco	Rutaceae	0.1
**1**	*Cydista aequinoctialis* (L.) Miers	Bignoniaceae	0.1
**1**	*Cyperus articulatus* L.	Cyperaceae	0.1
**1**	*Dolichos lablab* L.	Fabaceae	0.1
**1**	*Eucalyptus citriodora* Hooker	Myrtaceae	0.1
**1**	*Eupatorium triplinerve* Vahl	Asteraceae	0.1
**1**	*Huperzia reflexa* (Lam.) Trevis.	Lycopodiaceea	0.1
**1**	*Huperzia* sp.	Lycopodiaceae	0.1
**1**	*Ipomoea batatas* (L.) Lam.	Convolvulaceae	0.1
**1**	*Lycopersicon hirsutum* Dunal	Solanaceae	0.1
**1**	*Lycopodium clavatum* L.	Lycopodiaceae	0.1
**1**	*Oreopanax eriocephalus* Harms	Araliaceae	0.1
**1**	*Peperomia hartwegiana* Miq.	Piperaceae	0.1
**1**	*Perperomia galiodes* H.B.K.	Piperaceaea	0.1
**1**	*Plantago sericea* Ruiz & Pav. var. *lanuginosa* Grieseb.	Plantaginaceae	0.1
**1**	*Portula villosa* H.B.K.	Portulacaceae	0.1
**1**	*Psittacantus chanduyensis* Eichler	Loranthaceae	0.1
**1**	*Punica granatum* L.	Punicaceae	0.1
**1**	*Puya weberbaueri* Mez.	Bromeliaceae	0.1

**1**	*Tropaeolum minus* L.	Tropaeolaceae	0.1	**1**	*Senecio pseudotites* Grieseb.	Asteraceae	0.1
**1**	*Valeriana bonplandiana* Wedd.	Valerianaceae	0.1	**1**	*Sicyos baderoa* Hook. & Arn.	Cucurbitaceae	0.1
**1**	*Vallesia glabra* (Cav.) Link	Apocynaceae	0.1	**1**	*Thelypteris* cf. *scalaris* (Christ.) Alton	Thelypteridaceae	0.1
**1**	*Zornia reticulata* Sm.	Fabaceae	0.1				
